# Elevation-associated variation in bumble bee venom composition is not retained under common-garden conditions

**DOI:** 10.1098/rspb.2026.2191

**Published:** 2026-11-11

**Authors:** N. Pinar Barkan, Morgan E. Christman, Kelton M. Verble, Sam D. Heraghty, Claire Campion, Ellen C. Keaveny, Sarah A. Waybright, Gwendolyn M. Stamper, Jeffrey D. Lozier, Michael E. Dillon, James P. Strange

**Affiliations:** ^1^ Department of Entomology, The Ohio State University, Columbus, OH, USA; ^2^ Department of Entomology, Louisiana State University, Baton Rouge, LA, USA; ^3^ Department of Biological Sciences, The University of Alabama, Tuscaloosa, AL, USA; ^4^ USDA-ARS Invasive Insect Biocontrol & Behaviour Laboratory, Beltsville, MD, USA; ^5^ Department of Zoology & Physiology, University of Wyoming, Laramie, WY, USA; ^6^ Department of Biology, The University of North Carolina at Chapel Hill, Chapel Hill, NC, USA; ^7^ Department of Neurobiology Physiology and Behaviour, University of California Davis, Davis, CA, USA

**Keywords:** venomics, proteomics, liquid chromatography–tandem mass spectrometry, elevation, common garden

## Abstract

Bumble bees (*Bombus*) are vital pollinators across diverse habitats. Female bumble bees have a defensive, venomous sting used against predators. Little is known about the factors associated with intraspecific variation in bumble bee venom. We studied the venom composition of wild-caught *Bombus vosnesenskii* and tested for elevation-associated differences along an elevation gradient in Oregon, USA. Using a common garden approach with laboratory-reared colonies sourced from wild populations, we also tested the influence of captivity on venom response. Proteomic analysis using liquid chromatography–tandem mass spectrometry revealed clear elevation-associated variation in venom composition among wild populations, particularly in the abundance of the bombolitin-like antimicrobial peptide. By contrast, comparable elevation-associated differences were not evident under common-garden conditions when colonies were reared under shared laboratory conditions. More broadly, our findings demonstrate substantial intraspecific variation in bumble bee venom composition, and captive populations may not fully reflect wild variability, impacting the interpretation of venom studies relying on captive species.

## 1. Introduction

Venoms function as biochemical tools mediating interactions between organisms, including predation and defence [1,2]. Differences in venom composition are associated with these functional roles [3,4], producing paralysing effects in predatory venoms [5–7] and pain-inducing effects in defensive venoms [8–11]. Venom systems are widespread across the animal kingdom, occurring in taxa, such as cnidarians, molluscs, arthropods, amphibians, fish and mammals [12,13]. While many lineages primarily use venom for prey capture [12,14–16], others rely on venom for antipredator defence [4,17,18] and some, particularly arthropods, use venom in both contexts [19]. Despite this functional and taxonomic breadth, the drivers of venom composition in systems where venom functions primarily in defence, rather than prey capture, remain incompletely understood.

Predator–prey interactions impose strong selective pressures that can drive the evolution of complex venom phenotypes in venomous animals [20]. Among the key selective drivers of venom evolution are diet composition [21,22] and predator pressure [23], both of which can shape venom traits through natural selection. In addition, other factors, such as age [24], geography [25,26] and seasonality [27] are known to correlate with venom variation. Such variation is observed both within and between species [28] and across different lineages including snakes, scorpions [29,30], spiders [31,32] and *Conus* snails [9,33]. Despite the increasing recognition of this complexity, the role of non-genetic sources of venom variation remains largely underexplored. In Hymenoptera, factors influencing venom composition are limited to a few studies in bees. For instance, seasonal changes cause fluctuations in two major toxins, phospholipase A_2_ and melittin, of the Africanized honeybee [34]. Caste-specific differences in queen and winter honeybee workers’ venom proteomes have also been reported [35]. Further, interspecific [36], population-level and elevation-mediated environmental changes are believed to cause variation in wild bumble bee venom toxins, leading to decreased levels of toxins at higher elevations [37]. Within this context of limited evidence for environmentally driven venom variation, stinging insects of the order Hymenoptera provide a particularly informative system, occurring across diverse ecological niches and exhibiting complex social behaviours and effective defensive mechanisms [38].

The ancestral Hymenopteran venom most likely served a parasitic function, used by parasitoid ancestors to manipulate the physiology, metabolism and immune systems of their hosts [39]. Over evolutionary time, this venom diversified and gained new specialized activities as Hymenoptera radiated into new ecological roles and social lifestyles [40]. These shifts are closely linked to the evolution of the ovipositor–stinger apparatus: originally adapted for egg laying in parasitic wasps, it was later modified into a defensive stinger in social species [41]. Such transitions mark major steps in venom evolution within the group. For instance, most solitary wasps have retained predatory venoms [12,42], whereas parasitoid wasps use venom for host manipulation [43,44]. On the other hand, social Hymenoptera have evolved primarily defensive venoms, coinciding with the loss of predatory behaviours against prey [17,40]. Among these, ants use their venom for both defence and predation [45], while honey bees, bumble bees (genus *Bombus*) and social wasps use venom primarily for defence [8]. *Bombus* species, in particular, exemplify this transition, having lost the parasitic or predatory functions of venom and instead evolved a potent defensive system, including unique antimicrobial peptides known as bombolitins [46–48]. Venoms of social Hymenoptera have thus become external immune defence systems, combining antimicrobial and defensive roles [49–51], with cytolytic peptides, such as melittin (in honeybees), bombolitins (in bumble bees), mastoparans and crabrolins (in social wasps) and pilosulins (in *Myrmecia pilosula*) [52].

Bumble bees provide an excellent system to investigate possible pressures that drive venom variation, as they are widely distributed across temperate and cold habitats [53,54], including high-elevation mountain ranges [55–57]. Mountain ranges are diverse habitats that vary in elevational and environmental gradients as well as abiotic factors [58]. Therefore, they provide unique systems to study plant–pollinator and biotic interactions [59,60], community composition [61], animal and plant defences [37], physiological responses to thermal extremes [62–64] and climate change effects at the species and population levels [65,66]. As a habitat and elevational generalist inhabiting the mountain ranges of western North America, the yellow-faced bumble bee (*Bombus vosnesenskii*; Radoszkowski 1862) has recently attracted attention to study variation in local and adaptive responses to climate across different altitudes and latitudes [67–70]. *Bombus vosnesenskii* populations collected along an elevation gradient had weak population genetic structure [71,72]. Common-garden experiments using laboratory-reared colonies have also suggested evidence for local adaptation based on discrepancies in cold tolerance at the transcriptomic level [73], chill coma recovery times [74] and energy and toxin metabolomes [75]. Understanding how such environmental and genetic factors shape venom variation requires reliable methods for venom collection and comparison across populations. For large-scale venom collection, venom is extracted in specialized facilities from venomous animals kept in captivity [76,77]. One concern with long-term captivity is that proteomic differences between wild and captive venoms may lead to mismatches in antibody composition in antivenom production, which is essential for broad protection against envenomations [78]. However, most studies indicate that captivity does not significantly alter venom composition, resulting in only minor variations in snakes [76,79–82] and scorpions [78]. This suggests that genetic differences play a larger role in intraspecific venom variation than environmental factors in snakes. Importantly, how captivity studies reflect wild variability in bumble bee venom remains elusive.

Environmentally associated variation in primarily defensive venoms is largely unknown. Given the knowledge gaps, we sought to evaluate whether any potential venom divergence of yellow-faced bumble bees collected along an elevation gradient is influenced by captivity. We initially compared venom composition between workers collected from low- and high-elevation sites, which were previously described to have low genetic structure [71,72]. Here, we hypothesized that population-level differences would be observed in their venom composition. We also expected a decrease in the abundance of certain venom toxins in high-elevation sites, as previously reported in bumble bees [37]. We then tested whether population-level venom phenotypes observed in wild populations were maintained or erased once wild-caught low- and high-elevation gynes were reared in captivity using a common garden approach. In this experimental set-up, venom composition converging after laboratory rearing of wild-derived colonies would suggest acclimatization and phenotypic plasticity. By contrast, persistent differentiation would indicate the potential for genetically determined venom composition. Both experiments allowed us to examine the dynamics of environmentally induced compositional differences in a primarily defensive venom. Further, to our knowledge, this is the first study where a common-garden approach was used in Hymenoptera to test venom responses across an elevation gradient.

## 2. Material and methods

### (a) Collection of low- and high-elevation wild *Bombus vosnesenskii* populations

We sampled wild *B. vosnesenskii* populations in late July and early August in 2022 from the same or nearby low- and high-elevation sites as the gynes. Each collected worker was immediately frozen on dry ice to reduce worker bee stress and maximize potential venom collection. Thirty foraging workers per site were collected from four low-elevation (9, 51, 62, and 122 m; annual mean of 10–12°C) and four high-elevation sites (1311, 1463, 1618, and 1633 m; annual mean of approx. 4°C) in Oregon, USA, representing varying diet, age, temperature and humidity conditions (figure 1; supplementary material, table S1) [69,73]. In total, 240 workers were collected with 120 sampled in late July and 120 sampled in early August (30 workers per site). Frozen bees were shipped to the Ohio State University (OSU) for venom collection.

**Figure 1.**
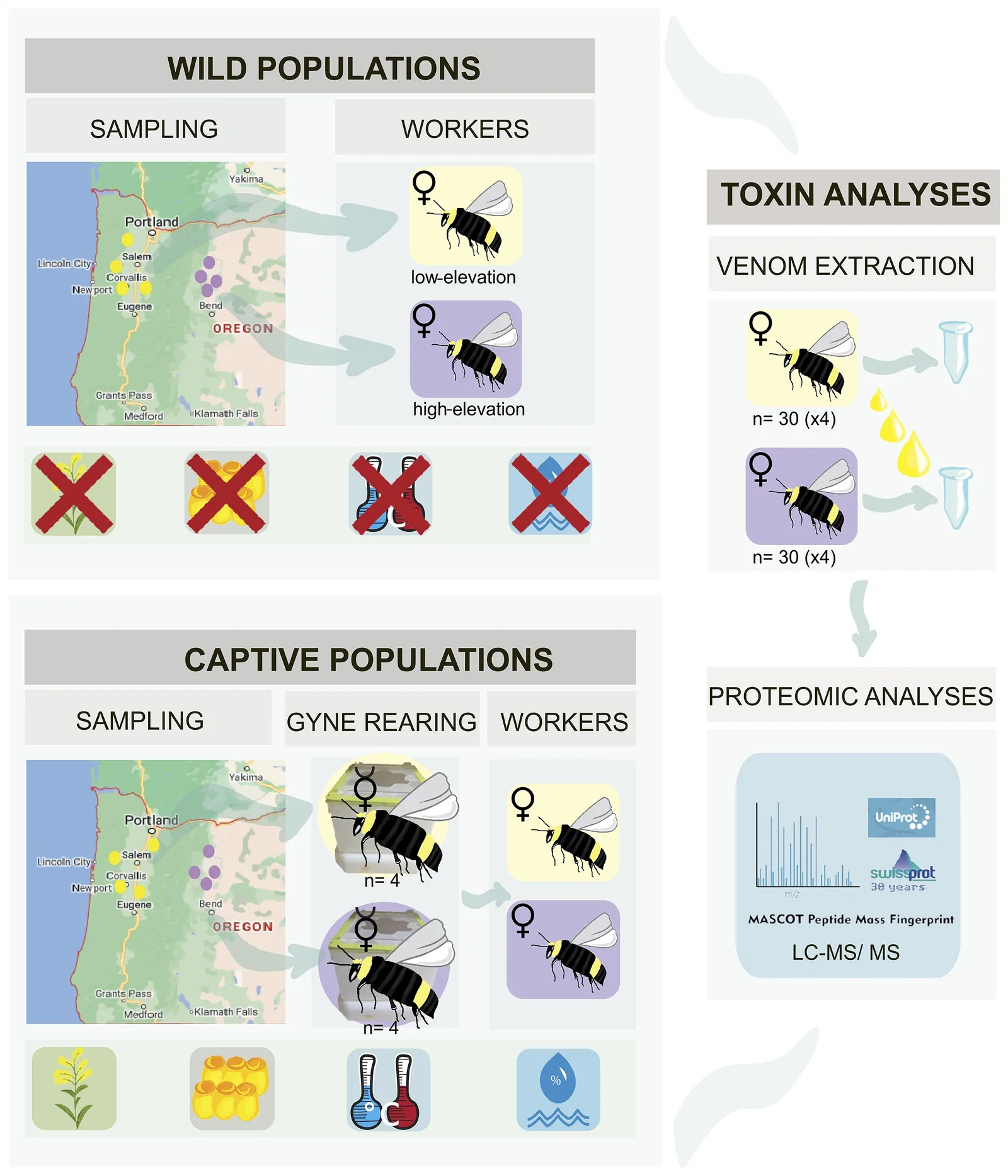
Workflow of the study design and experimental procedures. Wild low- and high-elevation populations were collected from four sites each with variable diet, age and environmental conditions, including temperature and humidity. Captive populations were reared from four wild-caught gynes in a laboratory setting with constant diet, age and environmental conditions. Venom was extracted from 30 workers of both low- and high-elevation populations and proteomically analysed using liquid chromatography–tandem mass spectrometry.

### (b) Rearing *Bombus vosnesenskii* colonies from low- and high-elevation gynes

A total of 175 nest-searching yellow-faced bumble bee gynes were net-collected from low- (37, 52, 58, and 76 m) and high-elevation sites (1311, 1466, 1531, and 1687 m) in April and June of 2023, respectively (figure 1; supplementary material, table S1). Each gyne was placed in a separate vial, which included small breathing holes and a piece of cardboard to reduce bee stress to the gyne. Collected gynes were shipped to our laboratory. Gynes were reared in a captive laboratory setting as described in Christman *et al.* [83]. Briefly, colonies were reared under constant temperature and relative humidity levels (28 ± 2°C and 65 ± 2%, respectively) and provided with the same pollen provisions and artificial nectar ad libitum.

### (c) Venom collection

Venom was extracted from each specimen by surgically removing the venom reservoir from the stinger under sterile conditions, followed by mechanical disruption of the reservoir membrane to release crude liquid venom, as described in [36]. Owing to limited sample volumes, crude liquid venom from 30 workers was pooled for both wild and captive populations. For wild populations, venom was extracted and pooled per site, resulting in four biological replicates at the low and high elevation. For each captive colony, once it exceeded 100 workers, we removed 30 workers from the single gyne associated with that colony. This was done for all eight colonies (one gyne per site for the four low-elevation and four high-elevation sites). Venom from the 30 workers of each colony was extracted and pooled. Each pooled sample, consisting of venom reservoirs from 30 workers, was placed in a single Eppendorf LoBind^®^ tube (Eppendorf AG, Hamburg, Germany), was disrupted and stored at −40°C until further use.

### (d) Proteomic analysis

Proteomic analysis was generated at the Campus Chemical Instrument Centre Mass Spectrometry and Proteomics Facility at the OSU. For each venom sample, quantitative and qualitative proteomic analyses were conducted, with proteins and peptides analysed separately. Briefly, proteins and peptides were digested using trypsin and loaded onto an S-Trap (C02-micro-80, Protifi). Protein identification was performed using nano-liquid chromatography coupled with nanospray tandem mass spectrometry on an Orbitrap Fusion mass spectrometer (Thermo Scientific), equipped with a nanospray FAIMS Pro™ source and operated in positive ion mode. Protein and peptide concentrations were estimated by NanoDrop at 280 nm for loading normalization. Protein data were searched using Mascot Daemon by Matrix Science version 2.7.0 (Boston, MA), Proteome Discoverer (version 2.4, Thermo Scientific) and UniProt against the *B. vosnesenskii* genome available in the National Centre for Biotechnology Information. Label-free quantification was performed using spectral counts, in which relative protein quantification is calculated based on the number of MS/MS spectra identified from the same protein found in eight liquid chromatography–tandem mass spectrometry datasets. Up to four missed cleavages were allowed for the enzyme. A decoy database was included in the search to estimate the false discovery rate (FDR) and peptides were filtered using a 1% peptide-level FDR threshold. Modified peptides were manually validated. Proteins with ≥10 mean spectral counts in at least one group were retained for downstream toxin-focused analyses and this threshold was applied as an analytical filter rather than a detection cut-off. Candidate venom toxins were curated from the literature and cross-referenced against the UniProt ToxProt database using DIAMOND blastp (significance threshold: *e*-value < 1 × 10⁻³) following standard Proteome Discoverer workflows [84]. Signal peptides were identified using SignalP v5.0 (Eukarya model) with default decision thresholds [85]. A protein was considered to contain a signal peptide if the predicted class was SP(Sec/SPI) with a secretion probability of >0.5 and a defined cleavage-site probability.

### (e) Data analysis

Venom composition in captive and wild populations was compared using differential protein abundance analyses between low- and high-elevation venom samples. All statistical and visual analyses were performed in R v4.4.0 [86]. Protein spectral counts were filtered to retain proteins with a mean count of at least 10 in at least one elevation group. Spectral counts were normalized using *edgeR*. Differential abundance analysis on the whole venom proteome was performed with limma-voom (*limma*), which uses empirical Bayes moderation and a moderated *t*-test with Benjamini–Hochberg adjusted FDR values. Data were scaled to z-scores (i.e. standard scores indicating how many s.d. each value is above or below the mean; *pheatmap*). Principal component analysis (PCA) was performed on the voom-transformed abundance matrix of the full venom proteome using the prcomp function to visualize multivariate differences. The statistical significance of differences was assessed by permutational multivariate analysis of variance (PERMANOVA) using *vegan* (adonis2 function) based on 9999 permutations. Data were visualized using *dplyr*, *tidyr*, *ggplot2* and associated R packages.

## 3. Results

### (a) Elevation-associated differences are observed in wild populations but not in common-garden colonies

Elevation-associated patterns in venom composition differed between wild-collected workers and common-garden colonies. In wild workers, we identified 909 proteins and peptides in both low- and high-elevation samples (supplementary material, table S2). Of these, 316 proteins and peptides exhibited an abundance exceeding five spectral counts. For captive workers, 583 proteins and peptides were characterized in low- and high-elevation samples, with 258 displaying an abundance above five spectral counts (supplementary material, table S3). In total, 40 proteins were considered venom toxins across wild and captive populations. Among them, 22 showed significant homology to curated venom toxins (*e*-value < 1 × 10⁻³) (supplementary material, table S4). An additional 18 proteins were included based on prior evidence supporting their venom or venom-gland origin [36,37,87–90], five of which also possessed a predicted signal peptide, providing independent support for their secretion into venom (supplementary material, table S4). These included 37 shared and three unique toxins (two in wild and one in captive populations). The unique toxins exclusive to wild populations include plancitoxin-1 isoform X1 and catalase (supplementary material, table S5). Meanwhile, CLIP domain-containing serine protease-2 was only identified in captive populations (supplementary material, table S5). Among the common proteins, we identified major bumble bee venom toxins, such as phospholipase A_2_ (PLA_2_), transmembrane protease serine 9-like, hyaluronidase, venom protease-like and melittin-like (supplementary material, table S5). Melittin-like, identified in both wild and captive venom samples, exhibits up to 78.6% amino acid sequence similarity to other bombolitins characterized from the venoms of *B. ignitus*, *B. lapidarius*, *B. pensylvanicus* and *B. terrestris* [48,87,91]. Given this high degree of amino acid sequence similarity and melittin not being a bumble bee venom toxin component, we will consider this a misidentification and refer to this peptide as ‘bombolitin-like’ (supplementary material, figure S1).

### (b) Elevation-associated differences in the proportion of toxin spectral counts were evident in wild populations but were not apparent in common-garden colonies

Venom toxin abundance in wild populations of *B. vosnesenskii* varied across elevation. In low-elevation wild populations, venom toxins accounted for 64.8% of the total proteomic abundance (8334/12 864 mean spectral counts), whereas in high-elevation wild populations they accounted for 53.4% (6871/12 878 mean spectral counts), indicating a marked reduction in the relative abundance of venom toxins at high elevation (figure 2A). By contrast, common-garden colonies showed only a modest shift in overall venom toxin abundance between elevations. Venom toxins comprised 63.9% of total proteomic abundance in low-elevation common-garden colonies (10 736/16 807 mean spectral counts) and 66.3% in high-elevation common-garden colonies (11 114/16 774 mean spectral counts) (figure 2B).

**Figure 2.**
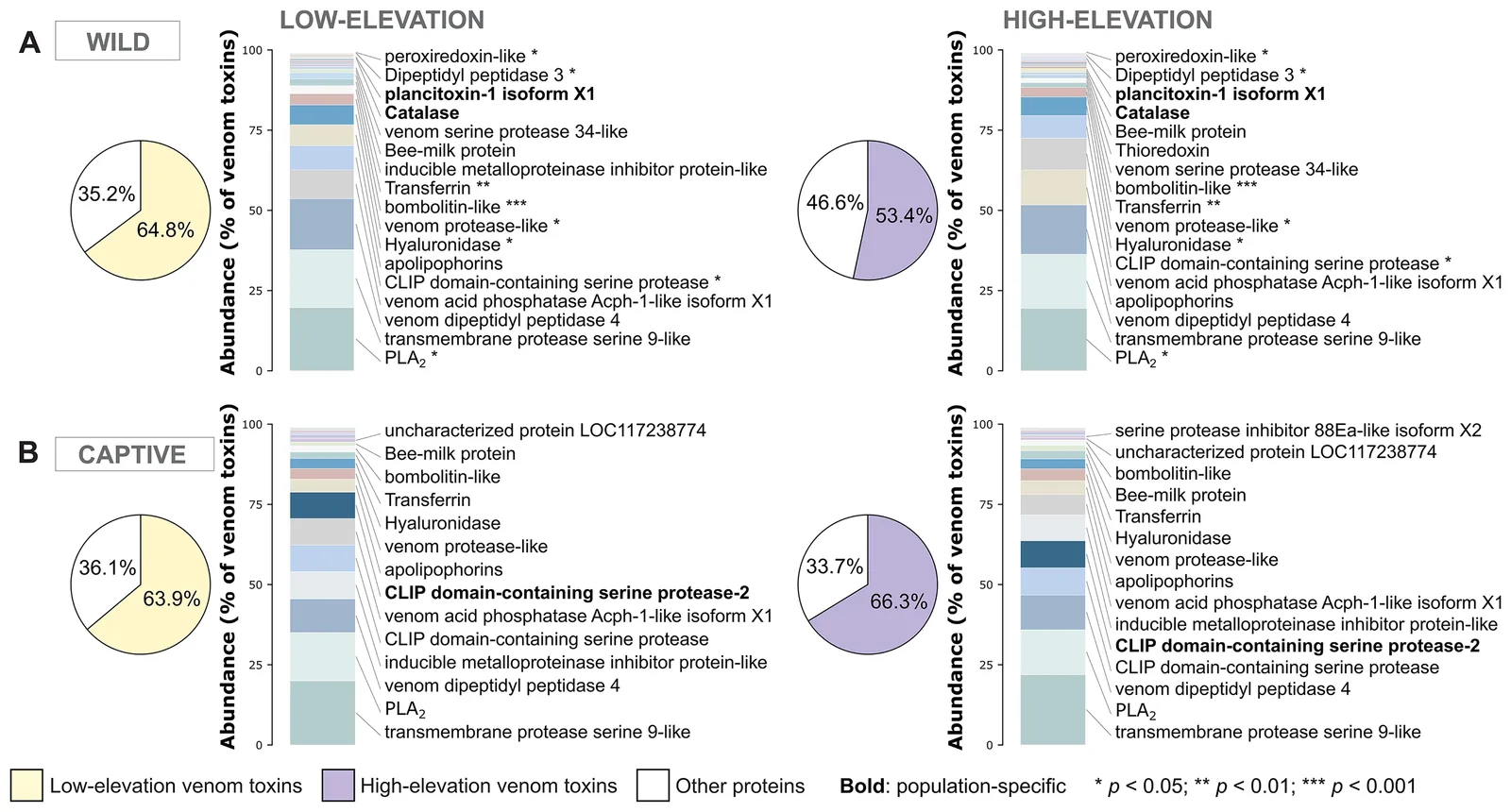
Pie charts and stacked bar plots depicting differences in major and unique venom toxins, as well as toxins with significant abundances in low and high elevation. (A) Wild populations. (B) Captive populations. Not all common or exclusive venom toxins are annotated on the stacked bar plot owing to low abundance. All other venom-related toxins are summarized in table S2. Yellow: low-elevation venom toxins; purple: high-elevation venom toxins; white: other proteins. Bold toxins represent unique proteins. Asterisks indicate proteins with significant differences at low- and high-elevation populations (*: *p* < 0.05, **: *p* < 0.01, ***: *p* < 0.001, limma-voom).

After observing overall venom variation between high- and low-elevation bumble bee populations, we set out to identify the particular toxins accountable for such differences. To this end, we performed a differential abundance analysis using limma-voom on the full proteome, including both toxin and non-toxin venom components. Our primary significance cut-off was a nominal *p*-value < 0.05 and an absolute log2 fold change of >0.5 (|log2FC| > 0.5). Among downregulated toxins, the decrease in canonical *Bombus* toxin bombolitin-like peptide also met the strict statistical cut-off of FDR < 0.05 (log2FC = –1.498, *p* = 5.32e-05, FDR = 0.010) (figure 3A,C). By sharp contrast, in captive populations, the abundance of none of the venom toxins significantly changed (nominal *p* < 0.05 & |log2FC| > 0.5) between elevations (figure 3B,C).

**Figure 3.**
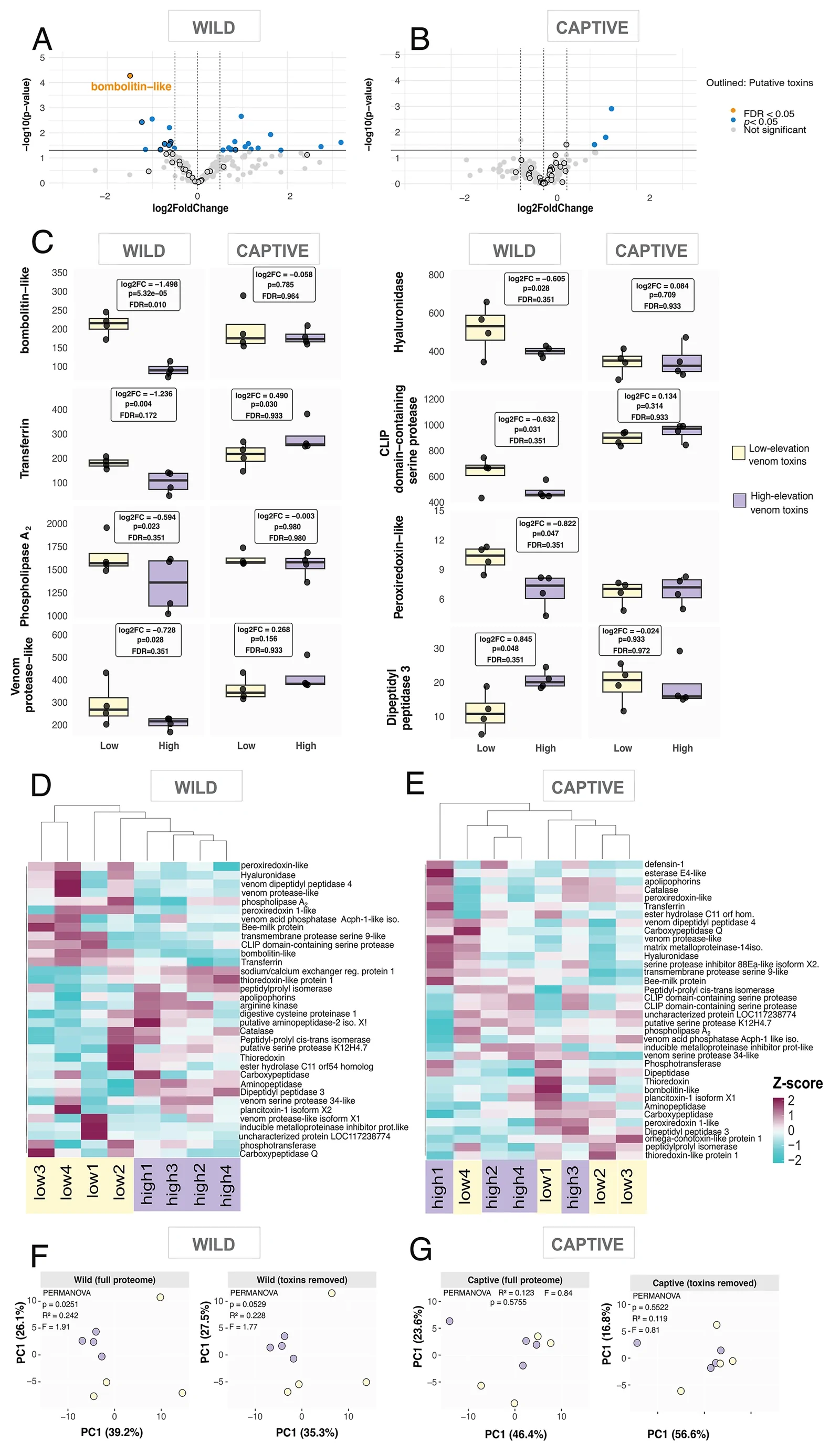
Venom toxins with significant differences in abundance in wild populations at low and high elevations. (A,B) Volcano plots depicting fold-change differences of the 41 venom toxins across low and high elevations. FDR-adjusted *p*-values are included along with their unadjusted *p-*values. The *p-*values and FDR are calculated using a moderated *t*-statistic (limma-voom). Orange: FDR < 0.05, blue: *p* < 0.05, grey: no significant difference in abundance between populations. Putative toxins are outlined. (A) Wild populations. (B) Captive populations. (C) Box plots showing the spectral counts (*y*-axis) of venom toxins across elevation in wild and captive samples. For each toxin, the corresponding |log2FC| (FC: fold change), *p*-value and FDR-adjusted *p*-value are shown. Four biological replicates were used for each group. Light yellow: low-elevation; light purple: high-elevation venom toxins. (D,E) Heat maps of relative abundance in wild and captive populations. (D) Thirty-four toxins shown for wild populations reveal population-level structure in the heat map dendrogram and PCA clustering. (E) Thirty-six toxins shown for common-garden colonies lack the population-level structure observed in plot (D). Protein abundances for low- and high-elevation groups are measured in four biological replicates. The z-scores are shown using a colour gradient. Light yellow: low-elevation venom toxins; light purple: high-elevation venom toxins. (F,G) PCA on the full proteome and with toxins removed for (F) wild populations and (G) captive populations. Statistical significance was tested using PERMANOVA.

These population-specific patterns in venom toxins were visualized using a heat map of standardized relative toxin abundances (row-wise z-scores). In the wild populations, unsupervised hierarchical clustering separated the high- and low-elevation samples (figure 3D). By contrast, common-garden colonies showed a heterogeneous clustering, with no clear separation according to elevation (figure 3E). To statistically test this hypothesis, we performed PCA on the proteomic output followed by PERMANOVA. PCA clustering was performed on the full venom proteome (178–185 proteins above a mean spectral count threshold of >10). Restricting the analysis to toxins alone (29–32 proteins) resulted in insufficient power for multivariate inference. Using the full proteome, PERMANOVA revealed a significant difference between high- and low-elevation samples in the wild populations (figure 3F), whereas no such difference is observed in the captive populations (figure 3G). Importantly, the contribution of toxins to venom plasticity is supported by the observation that removal of toxin proteins reduces the strength and statistical significance of the elevation effect in wild populations (PERMANOVA *p*-value increasing from 0.025 to 0.053, *R*^2^ decreasing from 0.242 to 0.228, *F* decreasing from 1.91 to 1.77) (figure 3F). Overall, our results support that wild populations display an elevation-associated venom signature which is at least partly attributable to venom toxins, and that this signature is completely lost under captivity.

## 4. Discussion

To our knowledge, this study provides the first comprehensive analysis of the venom profile of the yellow-faced bumble bee, *B. vosnesenskii*. Although the genetic basis of venom variation is well documented across many species, venom response to immediate environmental conditions is rare and has received comparatively little attention. This study emphasizes this phenomenon, showing that such differences are present in wild populations but are not consistently retained under common-garden conditions, suggesting that environmentally associated variation may play a more important role in shaping venom composition than previously appreciated.

In our proteomic analysis, we were able to detect major bumble bee venom toxins identified in previous studies [36,37,47,87,88,92], mostly belonging to hydrolases, transferases, lyases, oxidoreductases and other allergens. Among these toxins, PLA_2_, transmembrane protease serine 9-like, venom dipeptidyl peptidase 4 and venom acid phosphatase Acph-like isoform X1 were the most abundant venom components (figure 2). These toxins are responsible for inducing instant local pain, allergic reactions and inflammation [92–95]. Moreover, we also identified the bombolitin-like peptide, a highly potent venom toxin in bumble bees known for its haemolytic, antimicrobial, histamine-releasing and PLA_2_-stimulating activities [46,48,91].

Notably, we observed that the relative abundance of venom toxins decreased from 64.8% of the venom at low elevation to 53.4% at high elevation in wild populations. Elevation-dependent discrepancies were also previously observed at the population level for the common carder bee, *B. pascuorum* [37]. By contrast, this was not retained under common-garden conditions, suggesting that the broad shift in venom composition is not detected under the environmental filter (i.e. diet, microbiome, thermal variance and predation regime) of captivity [81,96–98]. To our knowledge, this is the first study to evaluate the effects of captivity on intraspecific venom variation in Hymenoptera. Our results are contrary to some studies on snake venoms [76,78–80,82], which suggest evidence for ontogenetic or phylogenetic relatedness rather than the environment as drivers of venom plasticity. These differing results may suggest significant differences in venom use between bees and snakes, as well as the greater exposure of snake venoms to natural selection [4,99]. However, other studies in snakes indicate that venom variation may result from both natural population-level differences and adaptation to captive environments, which can occur simultaneously and independently [22,81,100,101].

In wild populations, the abundance of seven venom toxins decreased at high elevations (log2FC < –0.5, nominal *p* < 0.05). These include venom enzymes responsible for inducing inflammation and allergic reactions (PLA_2_, hyaluronidase and venom protease-like), antimicrobial protein (bombolitin-like), potential enhancers of antimicrobial activity (transferrin [102]) and other venom functions (peroxiredoxin-like). Although six of these toxins were significant only at the nominal level, their collective decrease may indicate a shift in venom composition at high elevation, the functional consequences of which remain to be determined. This hypothesis needs to be tested in future studies. Among the changing proteins, the decrease in bombolitin-like was the most pronounced, both in terms of fold change and *p*-value, and it remained statistically significant even after implementing a strict significance threshold (log2FC > 0.5, FDR < 0.05, figure 3). These results imply that bombolitin-like, the signature *Bombus* venom component, may be particularly responsive to environmental context. Importantly, neither bombolitin-like nor the other six toxins decrease at high elevation in common-garden colonies, suggesting that the shift in individual venom components is also unique to wild populations.

At the multivariate level, PCA and hierarchical clustering (heat maps) similarly indicated separation between low- and high-elevation wild populations, whereas this pattern was absent under common-garden colonies. Because PCA and heat maps are descriptive, we formally tested overall proteomic differences using PERMANOVA on Euclidean distances calculated from voom-transformed log2-CPM data. In wild populations, the elevation-associated difference was significant for the full proteome but no longer met the significance threshold after venom toxins were removed from the analysis. This shift suggests that venom toxins contributed importantly to the multivariate separation between elevations. By contrast, common-garden colonies showed no comparable evidence of elevation-associated separation at the multivariate level. Taken together, the broad and individual shifts in venom toxins and the multivariate separation of low- and high-elevation proteomes provide convergent evidence that venom composition differs across elevation in wild populations, whereas these differences were not evident under common-garden conditions.

In this study, we provide evidence that bumble bee venom is primarily influenced by elevation. Low- and high-elevation *B. vosnesenskii* populations exhibit weak genetic structure [71,72] and show few signs of local adaptation in their genomes [67,68], yet they possess distinct methylation patterns [70]. By means of thermal adaptation, *B. vosnesenskii* populations have shown evidence of local adaptation or at least population-specific responses for cold tolerance in laboratory-reared colonies [73–75]. However, the role of biotic factors, such as diet, cannot be ruled out as a driver of population-level differences. Previous research has demonstrated the impact of dietary pressures on predatory venomous organisms [21,22,103] and on species capable of shifting from offensive to defensive venom, such as scorpions and cone snails [23,9,104]. For example, Gangur *et al.* [23] found evidence of adaptive phenotypic plasticity in venom composition in response to predation pressure in scorpions, with the changes occurring in the direction predicted by life history theory and towards more defensive toxins in high-predation environments.

An important limitation of this study is that wild populations were sampled in 2022, whereas common-garden colonies were established and sampled in 2023. Consequently, year- or season-specific effects cannot be fully separated from the effects of captivity or standardized rearing conditions. The attenuation of elevation-associated differences observed in captive-derived workers is therefore consistent with, but does not uniquely demonstrate, environmentally induced plasticity owing to captivity. Nonetheless, the persistence of elevation-associated differences in wild populations, coupled with their reduction under common-garden conditions, indicates that venom composition in *B. vosnesenskii* is responsive to elevational context. Although a fully crossed, multi-year design would be required to disentangle year and captivity effects conclusively, such designs are logistically challenging in bumble bees owing to colony phenology and the difficulty of repeatedly sampling the same populations across years. The present approach therefore provides a tractable first step towards evaluating whether venom phenotypes primarily reflect fixed population differences or environmentally responsive traits.

Importantly, the recent availability of a high-quality *B. vosnesenskii* genome [105] provides a valuable framework for interpreting these ecological and physiological patterns at the molecular level. This genomic resource enables more accurate annotation of toxin-related genes and facilitates linking environmental and epigenetic variation to specific genomic loci associated with venom composition. Future integrative analyses combining genomic, epigenomic and ecological data will help clarify how both intrinsic and extrinsic factors shape venom diversity and elevation-associated phenotypic variation across *B. vosnesenskii* populations. In summary, while elevation-associated factors are believed to play the primary role in shaping venom phenotypes in the wild, a combination of environment, diet and, to a lesser extent, age may contribute to population-level variation. In this study, both low- and high-elevation workers were collected during peak season, but we cannot rule out any influence of the exact developmental stage on the venom composition. Some of the elevation-associated differences could therefore reflect demographic or phenological differences among sampled workers/colonies.

The observed composition of the venom reservoir likely reflects the balance among venom synthesis, previous venom expenditure and replenishment. Lower toxin abundance could therefore partly reflect slower replenishment following venom use rather than reduced toxin production alone. However, because venom from 30 workers was pooled within each biological replicate, stochastic differences in recent venom expenditure by individual workers are unlikely to fully explain the consistent elevation-associated differences observed across pools. One possible explanation is that high-elevation populations experience different predation pressures or frequencies of biotic interactions [37,106], which may influence patterns of venom use or the relative abundance of venom components [107–110]. Whether these compositional differences reflect altered venom production or translate into changes in venom potency or defensive function remains to be tested directly.

Venom evolution in predatory organisms is highly influenced by molecular mechanisms, including gene regulation, duplication, positive selection and protein neofunctionalization [4,99,111,112]. Expressional and translational differences in venom gland genes are known to play major roles in the diversification of multiple venom phenotypes [1,113]. Therefore, we may hypothesize that the environmental pressures created by the common-garden settings may induce changes in venom gland genes encoding these toxins, resulting in adaptive responses to environmental factors [81,100]. Future studies implementing venom gland transcriptomics as well as functional assays, such as toxicity, haemolysis, antimicrobial activity and pain/defensive effectiveness tests are needed to further elucidate possible causes of the observed elevation-dependent population-level differences in venom composition at the gene expression level.

## 5. Conclusion

In this study, we present the first comprehensive characterization of the venom of *B. vosnesenskii*. To our knowledge, this is the first proteomic presentation showing that venom differences associated with elevation in wild populations are not consistently retained under captive, common-garden conditions. Collectively, our findings are suggestive of an elevation-associated phenotypic variation in venom composition among *B. vosnesenskii* populations. Together, these findings highlight venom as a labile ecological trait and caution against assuming that venom profiles measured in captivity fully capture the diversity and compositional variation present in natural populations.

## Supplementary Material



## Data Availability

The data and R code for replicating the analyses are freely available via Zenodo [114]. Supplementary material is available online [115].
