# Supplementary material for: Elevation-associated variation in bumble bee venom composition is not retained under common-garden conditions
Source: Proc Biol Sci. 2026 Nov 11;293(2082):20262191. doi: 10.1098/rspb.2026.2191 (PMC13751118; doi:10.1098/rspb.2026.2191)
Supplement: Supplementary file 1 [file rspb.2026.2191.zip › rspb.2026.2191/rspb20262191_si_001.pdf]

Electronic Supplementary Material for: Elevation-associated variation in bumble bee venom composition is not retained under common-garden conditions

Authors: N. Pinar Barkan*, Morgan E. Christman, Kelton M. Verble, Sam D. Heraghty, Claire Campion, Ellen C. Keaveny, Sarah A. Waybright, Gwendolyn M. Stamper, Jeffrey D. Lozier, Michael E. Dillon, James P. Strange

*Corresponding author: N. Pinar Barkan, **e-mail:** pinarbarkan@gmail.com

Published in: Proceedings of the Royal Society B

DOI: 10.1098/rspb.2026.2191

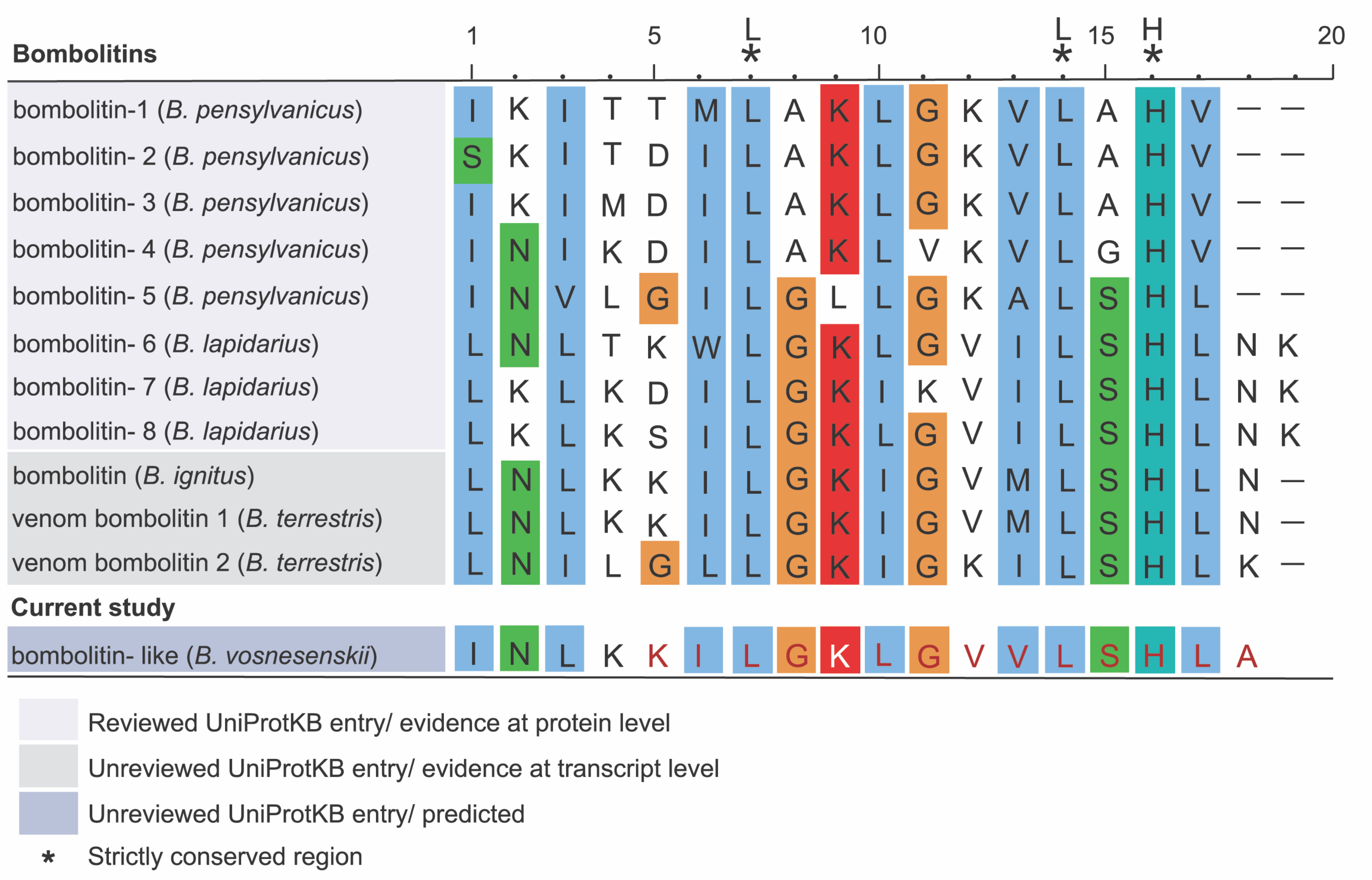

Supplementary Figure S1. Amino acid sequence alignment and similarity to other bombolitins and bombolitin-like identified in *B. vosnesenskii*. L* (leucine) and H* (histidine) are strictly conserved amino acids in all bombolitins and

bombolitin-like which is identified in the current study. The sequences of bombolitin 1-5 (*B. pensylvanicus*), bombolitin 6-8 (*B. lapidarius*), bombolitin (*B. ignitus*), venom bombolitin 1 and 2 (*B. terrestris*), and bombolitin-like (*B. vosnesenskii*) are obtained from Uniprot database (2002-2024 Uniprot consortium). Clustal X color scheme is as follows: blue (hydrophobic), green (polar), orange (glycines), red (positive charge), cyan (aromatic), white (unconserved).

Supplementary Table S1. Low- and high-elevation collection sites for captive and wild populations.

Sites		Location	Latitude	Longitude	Elevation (ft)	Elevation (m)
Low Elevation Site 1	Captive	Mosier- OR	45.684995	-121.392902	170	52
	Wild	Mt Tabor, Portland- OR	45.52142	-122.60432	200	61
Low Elevation Site 2	Captive	Mosier- OR	45.684995	-121.392902	120	37
	Wild	Blue Lake Park, Portland- OR	45.5574002	-122.4451	30	9
Low Elevation Site 3	Captive	Clark Meat Lab parking lot on OSU's campus, Corvallis- OR	44.3357	-123.1716	250	76
	Wild	Silktassel Rd, Corvallis- OR	44.6012948	-123.2904702	400	122
Low Elevation Site 4	Captive	Hood River Water–Front Park	45.4255	-123.3050	190	58
	Wild	Avery Park, Corvallis- OR	44.552925	-123.277159	167	51
High Elevation Site 1	Captive	McKenzie Pass; shooting star meadow-OR	44.22480	-121.87189	4810	1466
	Wild	McKenzie Pass- OR	44.22480	-121.87189	4800	1463
High Elevation Site 2	Captive	Mt Bachelor Edison Sno Park	43.93149	-121.59828	5022	1531
	Wild	Road to Mt Hood Meadows- OR	45.32886	-121.66264	5310	1618
High Elevation Site 3	Captive	Mount Hood Meadows Slopes- OR	45.33499	-121.66278	5536	1687
	Wild	Mt Hood Meadow Slopes- OR	45.33901	-121.65948	5359	1633
High Elevation Site 4	Captive	Tea Cup Sno Park- OR	45.32042	-121.62167	4301.5	1311
	Wild	Tea Cup Sno Park- OR	45.32042	-121.62167	4301.5	1311

Supplementary Table S2. All proteins identified in wild low- and high-elevation venom samples (with four biological replicates each).

<table>
<tr><th>Proteins</th><th>Accession Number</th><th>Molecular Weight</th><th>High elevation Site 1</th><th>High elevation Site 2</th><th>High elevation Site 3</th><th>High elevation Site 4</th><th>Low elevation Site 1</th><th>Low elevation Site 2</th><th>Low elevation Site 3</th><th>Low elevation Site 4</th></tr>
<tr><td>phospholipase A2 OS=Bombus vosnesenskii OX=207650 GN=LOC117231696 PE=4 SV=1</td><td>A0A6J3K1V5</td><td>21 kDa</td><td>1,132.80</td><td>1,586.50</td><td>1,020.70</td><td>1,614.40</td><td>1,556.40</td><td>1,954.00</td><td>1,487.00</td><td>1,580.90</td></tr>
<tr><td>transmembrane protease serine 9-like OS=Bombus vosnesenskii OX=207650 GN=LOC117231933 PE=4 SV=1</td><td>A0A6J3K2U7</td><td>61 kDa</td><td>1,033.50</td><td>1,202.20</td><td>1,128.60</td><td>1,259.30</td><td>1,757.60</td><td>948.74</td><td>1,455.20</td><td>1,830.90</td></tr>
<tr><td>venom dipeptidyl peptidase 4 OS=Bombus vosnesenskii OX=207650 GN=LOC117232985 PE=4 SV=1</td><td>A0A6J3K7R6</td><td>89 kDa</td><td>981.89</td><td>1,172.40</td><td>1,091.30</td><td>986.85</td><td>833.95</td><td>1,325.70</td><td>1,295.80</td><td>1,847.80</td></tr>
<tr><td>apolipophorins OS=Bombus vosnesenskii OX=207650 GN=LOC117238300 PE=4 SV=1</td><td>A0A6J3L183</td><td>375 kDa</td><td>882.65</td><td>582.61</td><td>831.51</td><td>698.53</td><td>678.06</td><td>589.03</td><td>608.05</td><td>312.05</td></tr>
<tr><td>venom acid phosphatase Acph-1-like isoform X1 OS=Bombus vosnesenskii OX=207650 GN=LOC117235967 PE=4 SV=1</td><td>A0A6J3KME8 (+1)</td><td>49 kDa</td><td>521.2</td><td>682.28</td><td>825.7</td><td>697.47</td><td>502.05</td><td>666</td><td>877.75</td><td>956.82</td></tr>
<tr><td>CLIP domain-containing serine protease OS=Bombus vosnesenskii OX=207650 GN=LOC117237098 PE=3 SV=1</td><td>A0A6J3KUF5 (+1)</td><td>40 kDa</td><td>446.97</td><td>464.44</td><td>448.95</td><td>573.45</td><td>744.27</td><td>431.96</td><td>664.44</td><td>667.33</td></tr>
<tr><td>Hyaluronidase OS=Bombus vosnesenskii OX=207650 GN=LOC117242244 PE=3 SV=1</td><td>A0A6J3LGZ0</td><td>88 kDa</td><td>409.86</td><td>390.46</td><td>370.94</td><td>422.93</td><td>339.45</td><td>563.9</td><td>496.49</td><td>656.05</td></tr>
<tr><td>platelet-derived growth factor subunit A isoform X1 OS=Bombus vosnesenskii OX=207650 GN=LOC117236816 PE=3 SV=1</td><td>A0A6J3KSG9</td><td>35 kDa</td><td>203.32</td><td>229.14</td><td>220.74</td><td>249.1</td><td>329.39</td><td>301.58</td><td>192.47</td><td>257.53</td></tr>
<tr><td>sp|TRYP_PIG|</td><td>sp|TRYP_PIG|</td><td>24 kDa</td><td>235.59</td><td>266.13</td><td>285.47</td><td>334.96</td><td>91.357</td><td>409.97</td><td>310.15</td><td>338.36</td></tr>
</table>

venom protease-like OS=Bombus vosnesenskii OX=207650 GN=LOC117231932 PE=4 SV=1	A0A6J3K168	44 kDa	167.82	226.06	226.55	204.58	201.99	282.74	251.31	430.47
Glyceraldehyde-3-phosphate dehydrogenase OS=Bombus vosnesenskii OX=207650 GN=LOC117230333 PE=3 SV=1	A0A6J3JT78	36 kDa	223.49	196.26	232.36	223.66	177.69	246.61	235.37	178.58
inducible metalloproteinase inhibitor protein-like OS=Bombus vosnesenskii OX=207650 GN=LOC117240279 PE=4 SV=1	A0A6J3LE39	11 kDa	15.329	25.688	43.152	33.92	554.85	12.566	18.389	43.235
melittin-like OS=Bombus vosnesenskii OX=207650 GN=LOC117232005 PE=4 SV=1	A0A6J3K401	6 kDa	114.57	84.258	70.537	94.339	221.27	208.91	169.17	244.37
Transferrin OS=Bombus vosnesenskii OX=207650 GN=LOC117230525 PE=3 SV=1	A0A6J3JT60	78 kDa	137.16	47.266	140.24	80.559	171.82	155.5	187.56	206.78
prosaposin OS=Bombus vosnesenskii OX=207650 GN=LOC117235656 PE=4 SV=1	A0A6J3KPJ1	104 kDa	138.77	117.14	105.39	128.26	94.71	105.24	136.08	172.94
Superoxide dismutase [Cu-Zn] OS=Bombus vosnesenskii OX=207650 GN=LOC117238299 PE=3 SV=1	A0A6J3L104	16 kDa	92.783	86.313	91.284	89.039	71.242	102.1	90.717	90.23
sp|K2C1_HUMAN|	sp|K2C1_HUMAN|	66 kDa	98.431	93.505	68.048	122.96	44.421	69.113	150.79	152.26
Aconitate hydratase, mitochondrial OS=Bombus vosnesenskii OX=207650 GN=LOC117243148 PE=3 SV=1	A0A6J3LLK6	86 kDa	83.908	55.487	73.027	76.319	67.051	31.415	61.295	65.793
venom serine protease 34-like OS=Bombus vosnesenskii OX=207650 GN=LOC117238058 PE=4 SV=1	A0A6J3L320	45 kDa	66.158	93.505	92.943	67.839	46.098	75.396	44.133	92.11
transketolase-like protein 2 isoform X2 OS=Bombus vosnesenskii OX=207650 GN=LOC117237681 PE=4 SV=1	A0A6J3KWQ1	67 kDa	74.226	61.652	65.558	72.079	60.346	43.981	74.78	45.115

Pyruvate kinase OS=Bombus vosnesenskii OX=207650 GN=LOC117235801 PE=3 SV=1	A0A6J3KLD6	60 kDa	70.192	69.872	62.239	66.779	71.242	76.967	52.714	43.235
aldo-keto reductase family 1 member B1-like OS=Bombus vosnesenskii OX=207650 GN=LOC117240155 PE=4 SV=1	A0A6J3LBB3	36 kDa	77.454	56.514	63.069	74.199	63.699	70.684	53.94	30.077
sp|K1C10_HUMAN|	sp|K1C10_HUMAN|	60 kDa	63.738	66.79	59.749	74.199	48.612	43.981	85.813	105.27
ATP-citrate synthase OS=Bombus vosnesenskii OX=207650 GN=LOC117243094 PE=3 SV=1	A0A6J3LQA8	120 kDa	57.283	69.872	47.301	78.439	52.803	48.693	35.551	26.317
Fructose-bisphosphate aldolase OS=Bombus vosnesenskii OX=207650 GN=LOC117238722 PE=3 SV=1	A0A6J3L5K3	40 kDa	55.67	58.569	47.301	73.139	66.213	40.84	49.036	41.356
Bee-milk protein OS=Bombus vosnesenskii OX=207650 GN=LOC117233259 PE=3 SV=1	A0A6J3K9U6	96 kDa	33.079	36.991	70.537	32.86	32.688	51.835	154.46	127.83
acetyl-CoA acetyltransferase, cytosolic OS=Bombus vosnesenskii OX=207650 GN=LOC117236714 PE=3 SV=1	A0A6J3KVA4	41 kDa	47.602	63.707	47.301	71.019	53.641	72.255	49.036	54.514
Phosphoglycerate mutase OS=Bombus vosnesenskii OX=207650 GN=LOC117230691 PE=3 SV=1	A0A6J3JVM9	29 kDa	60.511	55.487	68.878	65.719	36.04	31.415	60.069	20.678
Fructose-bisphosphate aldolase OS=Bombus vosnesenskii OX=207650 GN=LOC117238722 PE=3 SV=1	A0A6J3L3C4	39 kDa	53.249	54.459	45.642	69.959	62.861	0	45.359	0
3-ketoacyl-CoA thiolase, mitochondrial OS=Bombus vosnesenskii OX=207650 GN=LOC117234486 PE=3 SV=1	A0A6J3KFI3	42 kDa	51.636	54.459	53.94	65.719	46.936	48.693	46.584	31.957
chitinase-like protein Idgf4 isoform X2 OS=Bombus vosnesenskii OX=207650 GN=LOC117242559 PE=3 SV=1	A0A6J3LI89	48 kDa	50.022	46.239	43.152	58.299	56.155	76.967	55.166	63.913

Peptidyl-prolyl cis-trans isomerase OS=Bombus vosnesenskii OX=207650 GN=LOC117235564 PE=3 SV=1	A0A6J3KKY3	23 kDa	64.545	43.156	54.77	44.519	36.04	86.391	51.488	62.033
venom protease-like OS=Bombus vosnesenskii OX=207650 GN=LOC117238613 PE=4 SV=1	A0A6J3L1M8	33 kDa	30.659	36.991	30.704	39.219	87.167	0	44.133	0
Thioredoxin OS=Bombus vosnesenskii OX=207650 GN=LOC117234344 PE=3 SV=1	A0A6J3KE25	12 kDa	54.056	50.349	53.94	46.639	45.26	75.396	44.133	28.197
Transaldolase OS=Bombus vosnesenskii OX=207650 GN=LOC117234257 PE=3 SV=1	A0A6J3KGW6	37 kDa	48.408	45.211	53.11	54.059	58.67	20.42	36.777	33.836
hydroxyacid-oxoacid transhydrogenase OS=Bombus vosnesenskii OX=207650 GN=LOC117243594 PE=3 SV=1	A0A6J3LNR3	51 kDa	85.522	38.019	53.11	45.579	31.011	36.127	46.584	16.918
heat shock 70 kDa protein cognate 4 OS=Bombus vosnesenskii OX=207650 GN=LOC117238264 PE=3 SV=1	A0A6J3L4B2	71 kDa	52.443	42.129	47.301	45.579	37.716	65.972	41.681	41.356
uncharacterized protein LOC117238774 OS=Bombus vosnesenskii OX=207650 GN=LOC117238774 PE=3 SV=1	A0A6J3L3K9	100 kDa	35.5	39.046	36.513	30.74	96.386	40.84	29.422	31.957
sp|K1C9_HUMAN|	sp|K1C9_HUMAN|	62 kDa	45.988	39.046	39.833	69.959	15.925	31.415	76.006	73.312
antichymotrypsin-2-like isoform X2 OS=Bombus vosnesenskii OX=207650 GN=LOC117231437 PE=3 SV=1	A0A6J3JY50	45 kDa	56.477	26.716	38.173	48.759	43.583	54.976	39.229	31.957
peroxisomal multifunctional enzyme type 2 OS=Bombus vosnesenskii OX=207650 GN=LOC117231189 PE=4 SV=1	A0A6J3JWN4	80 kDa	34.693	55.487	23.236	65.719	38.554	53.406	19.614	16.918
heat shock 70 kDa protein cognate 5 OS=Bombus vosnesenskii OX=207650 GN=LOC117238386 PE=3 SV=1	A0A6J3L1N7	75 kDa	51.636	38.019	37.343	41.339	32.688	47.123	45.359	26.317

D-altritol 5-dehydrogenase-like isoform X1 OS=Bombus vosnesenskii OX=207650 GN=LOC117241762 PE=4 SV=1	A0A6J3LGD9	39 kDa	43.568	42.129	43.152	47.699	26.821	51.835	24.518	15.038
inositol 2-dehydrogenase-like OS=Bombus vosnesenskii OX=207650 GN=LOC117242414 PE=3 SV=1	A0A6J3LI37	43 kDa	48.408	32.881	45.642	37.1	11.734	43.981	38.003	18.798
Glycerol-3-phosphate dehydrogenase [NAD(+)] OS=Bombus vosnesenskii OX=207650 GN=LOC117231866 PE=3 SV=1	A0A6J3K2X5	41 kDa	46.795	33.909	42.322	43.459	29.335	6.283	35.551	24.437
Catalase OS=Bombus vosnesenskii OX=207650 GN=LOC117233114 PE=3 SV=1	A0A6J3K8Z3 (+1)	68 kDa	42.761	33.909	34.854	42.399	27.659	48.693	22.066	28.197
putative serine protease K12H4.7 OS=Bombus vosnesenskii OX=207650 GN=LOC117237063 PE=3 SV=1	A0A6J3KU14	56 kDa	31.466	41.101	39.003	37.1	26.821	50.264	34.325	30.077
probable enoyl-CoA hydratase, mitochondrial OS=Bombus vosnesenskii OX=207650 GN=LOC117242339 PE=3 SV=1	A0A6J3LJE0	32 kDa	52.443	39.046	40.663	36.04	18.439	15.708	26.97	11.279
alpha-L-fucosidase OS=Bombus vosnesenskii OX=207650 GN=LOC117242104 PE=3 SV=1	A0A6J3LKH1	56 kDa	37.113	33.909	36.513	26.5	23.468	42.41	40.455	62.033
sp|K22E_HUMAN|	sp|K22E_HUMAN|	66 kDa	33.886	41.101	29.875	54.059	18.439	18.849	51.488	54.514
Triosephosphate isomerase OS=Bombus vosnesenskii OX=207650 GN=LOC117242845 PE=3 SV=1	A0A6J3LP61	27 kDa	40.34	26.716	37.343	33.92	20.954	39.269	39.229	24.437
enoyl-CoA hydratase OS=Bombus vosnesenskii OX=207650 GN=LOC117232959 PE=3 SV=1	A0A6J3K7P0 (+1)	80 kDa	33.079	39.046	29.045	38.16	25.982	31.415	26.97	20.678
Phosphotransferase OS=Bombus vosnesenskii OX=207650	A0A6J3JUQ3 (+4)	51 kDa	31.466	31.853	34.854	25.44	27.659	42.41	40.455	16.918

GN=LOC117230814 PE=3 SV=1											
alpha-glucosidase OS=Bombus vosnesenskii OX=207650 GN=LOC117235298 PE=4 SV=1	A0A6J3KMJ2	65 kDa	11.295	52.404	34.854	15.9	47.774	7.8538	40.455	0	
Malate dehydrogenase OS=Bombus vosnesenskii OX=207650 GN=LOC117232774 PE=3 SV=1	A0A6J3K5D0	36 kDa	37.113	25.688	34.024	29.68	26.821	47.123	26.97	37.596	
Malic enzyme OS=Bombus vosnesenskii OX=207650 GN=LOC117230634 PE=3 SV=1	A0A6J3JUX4 (+2)	71 kDa	28.238	39.046	29.045	34.98	27.659	34.557	29.422	33.836	
actin-5C OS=Bombus vosnesenskii OX=207650 GN=LOC117242144 PE=3 SV=1	A0A6J3LGG6	42 kDa	28.238	28.771	30.704	28.62	46.098	14.137	28.196	20.678	
peroxiredoxin 1-like OS=Bombus vosnesenskii OX=207650 GN=LOC117238633 PE=3 SV=1	A0A6J3L2X1	22 kDa	24.204	25.688	19.087	25.44	39.393	39.269	15.937	41.356	
Nucleoside diphosphate kinase OS=Bombus vosnesenskii OX=207650 GN=LOC117236192 PE=3 SV=1	A0A6J3KNU4	19 kDa	33.886	29.798	39.003	31.8	18.439	39.269	28.196	20.678	
LOW QUALITY PROTEIN: vitellogenin-like OS=Bombus vosnesenskii OX=207650 GN=LOC117231930 PE=4 SV=1	A0A6J3K186	202 kDa	70.999	11.303	103.73	0	5.0288	6.283	14.711	1.8798	
general odorant-binding protein 56d-like OS=Bombus vosnesenskii OX=207650 GN=LOC117239448 PE=4 SV=1	A0A6J3L6L1	15 kDa	37.113	29.798	40.663	36.04	33.526	20.42	25.744	5.6394	
Aminopeptidase OS=Bombus vosnesenskii OX=207650 GN=LOC117235017 PE=3 SV=1	A0A6J3KHZ5	99 kDa	33.079	30.826	35.684	30.74	22.63	14.137	26.97	28.197	
xanthine dehydrogenase/oxidase-like isoform X2 OS=Bombus vosnesenskii OX=207650 GN=LOC117242522 PE=3 SV=1	A0A6J3LIR7 (+1)	141 kDa	32.272	33.909	43.982	36.04	15.925	25.132	39.229	5.6394	

fatty acid synthase OS=Bombus vosnesenskii OX=207650 GN=LOC117234004 PE=4 SV=1	A0A6J3KC8 2	262 kDa	35.5	48.294	35.684	22.26	6.7051	31.415	9.8072	0
ornithine decarboxylase 2-like OS=Bombus vosnesenskii OX=207650 GN=LOC117238735 PE=3 SV=1	A0A6J3L3E1	47 kDa	18.557	25.688	28.215	25.44	39.393	9.4245	20.84	43.235
plancitoxin-1 isoform X2 OS=Bombus vosnesenskii OX=207650 GN=LOC117233141 PE=3 SV=1	A0A6J3K9T1 (+1)	43 kDa	25.011	29.798	32.364	27.56	22.63	23.561	28.196	39.476
Peptidyl-prolyl cis-trans isomerase OS=Bombus vosnesenskii OX=207650 GN=LOC117235053 PE=3 SV=1	A0A6J3KL98	23 kDa	35.5	29.798	27.385	28.62	23.468	37.698	18.389	22.558
ATP synthase subunit beta OS=Bombus vosnesenskii OX=207650 GN=LOC117243357 PE=3 SV=1	A0A6J3LME 2	55 kDa	33.079	36.991	38.173	11.66	14.248	10.995	9.8072	5.6394
alpha-glucosidase OS=Bombus vosnesenskii OX=207650 GN=LOC117233420 PE=4 SV=1	A0A6J3K922	69 kDa	12.909	30.826	39.003	18.02	22.63	9.4245	51.488	13.159
actin, muscle OS=Bombus vosnesenskii OX=207650 GN=LOC117235961 PE=3 SV=1	A0A6J3KMX 3	42 kDa	25.011	24.661	21.576	27.56	38.554	10.995	25.744	18.798
LOW QUALITY PROTEIN: endoplasmic reticulum chaperone BiP-like OS=Bombus vosnesenskii OX=207650 GN=LOC117240895 PE=3 SV=1	A0A6J3LCD 0	73 kDa	28.238	27.743	28.215	36.04	17.601	18.849	20.84	5.6394
ubiquitin-40S ribosomal protein S27a OS=Bombus vosnesenskii OX=207650 GN=LOC117233173 PE=3 SV=1	A0A6J3K9D 4 (+3)	18 kDa	25.011	21.578	24.896	23.32	27.659	3.1415	17.163	1.8798
Beta-hexosaminidase OS=Bombus vosnesenskii OX=207650 GN=LOC117230346 PE=3 SV=1	A0A6J3JTD9	62 kDa	12.909	31.853	21.576	26.5	20.115	73.825	13.485	9.399
cytochrome P450 315a1, mitochondrial OS=Bombus	A0A6J3L998	61 kDa	14.523	20.551	14.937	26.5	48.612	7.8538	15.937	7.5192

vosnesenskii OX=207650 GN=LOC117239595 PE=3 SV=1										
Isocitrate dehydrogenase [NADP] OS=Bombus vosnesenskii OX=207650 GN=LOC117231466 PE=3 SV=1	A0A6J3K047 (+1)	46 kDa	33.886	21.578	24.066	23.32	13.41	15.708	23.292	18.798
Glutathione peroxidase OS=Bombus vosnesenskii OX=207650 GN=LOC117238361 PE=3 SV=1	A0A6J3L0H1 (+1)	19 kDa	25.818	20.551	22.406	27.56	17.601	28.274	25.744	20.678
LOW QUALITY PROTEIN: fumarylacetoacetate hydrolase domain-containing protein 2A OS=Bombus vosnesenskii OX=207650 GN=LOC117240147 PE=3 SV=1	A0A6J3L8H4	38 kDa	25.011	21.578	24.896	21.2	20.115	34.557	15.937	11.279
translation elongation factor 2 OS=Bombus vosnesenskii OX=207650 GN=LOC117236689 PE=3 SV=1	A0A6J3KV39	95 kDa	25.818	25.688	19.916	21.2	20.954	23.561	13.485	9.399
Pyruvate carboxylase OS=Bombus vosnesenskii OX=207650 GN=LOC117233613 PE=4 SV=1	A0A6J3KA48	132 kDa	24.204	24.661	15.767	22.26	25.982	12.566	12.259	3.7596
glycine--tRNA ligase OS=Bombus vosnesenskii OX=207650 GN=LOC117240358 PE=3 SV=1	A0A6J3L9H6	55 kDa	31.466	13.358	24.896	26.5	13.41	20.42	25.744	20.678
Malate dehydrogenase OS=Bombus vosnesenskii OX=207650 GN=LOC117232345 PE=3 SV=1	A0A6J3K3A1	36 kDa	29.045	21.578	24.896	21.2	17.601	29.844	19.614	18.798
Protein disulfide-isomerase OS=Bombus vosnesenskii OX=207650 GN=LOC117237128 PE=3 SV=1	A0A6J3KUB 9	56 kDa	31.466	23.633	28.215	23.32	16.763	20.42	7.3554	7.5192
trifunctional enzyme subunit beta, mitochondrial OS=Bombus vosnesenskii OX=207650	A0A6J3K330	51 kDa	27.431	19.523	26.555	24.38	15.087	26.703	11.033	13.159

GN=LOC117231974 PE=3 SV=1										
N-acyl-aliphatic-L-amino acid amidohydrolase OS=Bombus vosnesenskii OX=207650 GN=LOC117236511 PE=3 SV=1	A0A6J3KTI8	46 kDa	20.977	19.523	20.746	23.32	17.601	21.991	22.066	22.558
Aspartate aminotransferase OS=Bombus vosnesenskii OX=207650 GN=LOC117230654 PE=3 SV=1	A0A6J3JTX0	47 kDa	18.557	19.523	25.725	21.2	17.601	28.274	20.84	22.558
Protein disulfide-isomerase OS=Bombus vosnesenskii OX=207650 GN=LOC117239181 PE=3 SV=1	A0A6J3L956	56 kDa	23.397	19.523	19.087	23.32	20.954	25.132	15.937	16.918
Transgelin OS=Bombus vosnesenskii OX=207650 GN=LOC117242319 PE=3 SV=1	A0A6J3LHN 2	20 kDa	25.818	20.551	16.597	13.78	25.144	25.132	15.937	11.279
phosphopyruvate hydratase OS=Bombus vosnesenskii OX=207650 GN=LOC117233542 PE=3 SV=1	A0A6J3KBG 5	47 kDa	27.431	12.33	19.916	14.84	22.63	3.1415	38.003	24.437
actin, clone 205-like OS=Bombus vosnesenskii OX=207650 GN=LOC117235959 PE=3 SV=1	A0A6J3KMC 6	42 kDa	16.943	18.496	16.597	24.38	34.364	0	0	11.279
glutamate dehydrogenase [NAD(P)(+)] OS=Bombus vosnesenskii OX=207650 GN=LOC117231037 PE=3 SV=1	A0A6J3JXC8	62 kDa	25.818	20.551	19.087	19.08	17.601	9.4245	13.485	3.7596
cytoplasmic aconitate hydratase-like OS=Bombus vosnesenskii OX=207650 GN=LOC117230685 PE=3 SV=1	A0A6J3JV87	99 kDa	18.557	19.523	24.896	22.26	19.277	17.278	20.84	7.5192
neutral alpha-glucosidase AB isoform X2 OS=Bombus vosnesenskii OX=207650 GN=LOC117239666 PE=3 SV=1	A0A6J3L6Q2 (+3)	107 kDa	33.886	28.771	24.896	12.72	10.058	21.991	11.033	11.279
Hydroxymethylglutaryl-CoA synthase OS=Bombus vosnesenskii OX=207650	A0A6J3K4N 3	51 kDa	20.17	20.551	17.427	29.68	19.277	17.278	18.389	1.8798

GN=LOC117232655 PE=3 SV=1										
uncharacterized protein LOC117232577 isoform X2 OS=Bombus vosnesenskii OX=207650 GN=LOC117232577 PE=4 SV=1	A0A6J3K487 (+1)	48 kDa	18.557	23.633	24.896	18.02	15.087	23.561	8.5813	13.159
alpha-glucosidase OS=Bombus vosnesenskii OX=207650 GN=LOC117239768 PE=4 SV=1	A0A6J3L783	66 kDa	4.034	23.633	20.746	7.4199	28.497	0	20.84	1.8798
Dipeptidase OS=Bombus vosnesenskii OX=207650 GN=LOC117238734 PE=3 SV=1	A0A6J3L5M3	51 kDa	27.431	16.44	19.087	24.38	13.41	18.849	18.389	15.038
Multifunctional fusion protein OS=Bombus vosnesenskii OX=207650 GN=LOC117232948 PE=3 SV=1	A0A6J3K829 (+1)	63 kDa	20.977	15.413	19.087	23.32	9.2196	15.708	8.5813	13.159
Glucose-6-phosphate isomerase OS=Bombus vosnesenskii OX=207650 GN=LOC117234302 PE=3 SV=1	A0A6J3KDN0	63 kDa	17.75	10.275	17.427	22.26	15.087	4.7123	28.196	18.798
uncharacterized protein LOC117230545 OS=Bombus vosnesenskii OX=207650 GN=LOC117230545 PE=4 SV=1	A0A6J3JV84	49 kDa	22.591	14.385	19.916	23.32	11.734	20.42	22.066	13.159
uncharacterized protein LOC117234144 OS=Bombus vosnesenskii OX=207650 GN=LOC117234144 PE=4 SV=1	A0A6J3KF51	27 kDa	17.75	18.496	17.427	18.02	22.63	26.703	17.163	13.159
Rab GDP dissociation inhibitor OS=Bombus vosnesenskii OX=207650 GN=LOC117236932 PE=3 SV=1	A0A6J3KT72	50 kDa	20.17	17.468	20.746	20.14	16.763	21.991	17.163	11.279
antichymotrypsin-2-like OS=Bombus vosnesenskii OX=207650 GN=LOC117231439 PE=3 SV=1	A0A6J3K0H4	44 kDa	20.977	16.44	16.597	14.84	18.439	15.708	19.614	20.678
kynurenine--oxoglutarate transaminase 3 OS=Bombus vosnesenskii OX=207650	A0A6J3KWC1	51 kDa	21.784	17.468	14.937	25.44	13.41	15.708	14.711	13.159

GN=LOC117237489 PE=4 SV=1										
Dipeptidyl peptidase 3 OS=Bombus vosnesenskii OX=207650 GN=LOC117231034 PE=3 SV=1	A0A6J3JVW9	90 kDa	20.977	18.496	19.087	24.38	9.2196	4.7123	12.259	18.798
lysozyme OS=Bombus vosnesenskii OX=207650 GN=LOC117231586 PE=3 SV=1	A0A6J3K197	18 kDa	12.909	20.551	9.9582	12.72	21.792	25.132	22.066	22.558
peptidylglycine monooxygenase OS=Bombus vosnesenskii OX=207650 GN=LOC117231991 PE=4 SV=1	A0A6J3K367	37 kDa	20.977	23.633	19.916	19.08	5.867	23.561	15.937	16.918
aldehyde dehydrogenase, mitochondrial OS=Bombus vosnesenskii OX=207650 GN=LOC117240084 PE=3 SV=1	A0A6J3L942	56 kDa	25.818	22.606	19.916	14.84	9.2196	17.278	9.8072	3.7596
presequence protease, mitochondrial isoform X1 OS=Bombus vosnesenskii OX=207650 GN=LOC117231361 PE=4 SV=1	A0A6J3JZZ9 (+1)	121 kDa	20.17	13.358	11.618	19.08	18.439	17.278	14.711	11.279
protein dj-1beta-like OS=Bombus vosnesenskii OX=207650 GN=LOC117242227 PE=4 SV=1	A0A6J3LGV4	23 kDa	22.591	16.44	19.916	15.9	17.601	7.8538	20.84	7.5192
Annexin OS=Bombus vosnesenskii OX=207650 GN=LOC117239673 PE=3 SV=1	A0A6J3L7R4	36 kDa	17.75	18.496	14.937	13.78	15.925	18.849	9.8072	13.159
aldehyde dehydrogenase (NAD(+)) OS=Bombus vosnesenskii OX=207650 GN=LOC117232657 PE=3 SV=1	A0A6J3K4P4	57 kDa	18.557	18.496	14.107	18.02	13.41	15.708	11.033	5.6394
Glycerol-3-phosphate dehydrogenase OS=Bombus vosnesenskii OX=207650 GN=LOC117236450 PE=3 SV=1	A0A6J3KPT4 (+2)	81 kDa	16.136	14.385	22.406	21.2	12.572	12.566	9.8072	11.279
protein artichoke-like isoform X2 OS=Bombus vosnesenskii OX=207650	A0A6J3L645 (+2)	79 kDa	15.329	15.413	10.788	12.72	18.439	25.132	12.259	13.159

GN=LOC117239542 PE=4 SV=1										
chymotrypsin-1-like OS=Bombus vosnesenskii OX=207650 GN=LOC117231855 PE=4 SV=1	A0A6J3K0H0	28 kDa	5.6477	21.578	22.406	4.2399	20.115	9.4245	34.325	1.8798
hydroxysteroid dehydrogenase-like protein 2 OS=Bombus vosnesenskii OX=207650 GN=LOC117232682 PE=4 SV=1	A0A6J3K6L0	46 kDa	16.136	17.468	12.448	19.08	12.572	17.278	14.711	13.159
Prolyl endopeptidase OS=Bombus vosnesenskii OX=207650 GN=LOC117239543 PE=3 SV=1	A0A6J3L704	86 kDa	16.943	12.33	16.597	16.96	11.734	17.278	12.259	16.918
very long-chain specific acyl-CoA dehydrogenase, mitochondrial OS=Bombus vosnesenskii OX=207650 GN=LOC117238208 PE=3 SV=1	A0A6J3L0E8	69 kDa	17.75	21.578	14.937	23.32	10.896	1.5708	6.1295	5.6394
spermine oxidase-like OS=Bombus vosnesenskii OX=207650 GN=LOC117230418 PE=4 SV=1	A0A6J3JU51	56 kDa	20.17	13.358	18.257	10.6	12.572	20.42	6.1295	7.5192
glutamate synthase (NADH) OS=Bombus vosnesenskii OX=207650 GN=LOC117235398 PE=3 SV=1	A0A6J3KJD9 (+1)	229 kDa	12.102	3.0826	7.4687	15.9	29.335	3.1415	14.711	5.6394
cytochrome P450 6k1-like isoform X1 OS=Bombus vosnesenskii OX=207650 GN=LOC117242889 PE=3 SV=1	A0A6J3LM90	59 kDa	15.329	15.413	18.257	22.26	11.734	10.995	12.259	3.7596
Alpha-mannosidase OS=Bombus vosnesenskii OX=207650 GN=LOC117242330 PE=3 SV=1	A0A6J3LHP8	126 kDa	29.852	29.798	22.406	4.2399	0.83814	28.274	3.6777	0
alpha-glucosidase OS=Bombus vosnesenskii OX=207650 GN=LOC117235313 PE=4 SV=1	A0A6J3KIV4	65 kDa	0	24.661	0	0	22.63	0	18.389	0
ester hydrolase C11orf54 homolog OS=Bombus vosnesenskii OX=207650	A0A6J3L5T9	35 kDa	16.943	11.303	15.767	13.78	10.058	26.703	13.485	13.159

GN=LOC117238785 PE=4 SV=1										
Malic enzyme OS=Bombus vosnesenskii OX=207650 GN=LOC117230727 PE=3 SV=1	A0A6J3JUA2 (+1)	70 kDa	14.523	16.44	14.937	22.26	5.0288	15.708	11.033	11.279
fumarate hydratase OS=Bombus vosnesenskii OX=207650 GN=LOC117232291 PE=3 SV=1	A0A6J3K5M2	55 kDa	14.523	11.303	15.767	18.02	12.572	14.137	12.259	7.5192
protein FAM151B isoform X2 OS=Bombus vosnesenskii OX=207650 GN=LOC117238017 PE=3 SV=1	A0A6J3KZ70 (+1)	32 kDa	13.716	15.413	14.107	18.02	7.5433	9.4245	22.066	18.798
alanine transaminase OS=Bombus vosnesenskii OX=207650 GN=LOC117242549 PE=3 SV=1	A0A6J3LIK0	61 kDa	14.523	11.303	16.597	12.72	10.058	9.4245	13.485	13.159
long-chain-fatty-acid--CoA ligase ACSBG2 isoform X2 OS=Bombus vosnesenskii OX=207650 GN=LOC117235086 PE=4 SV=1	A0A6J3KHT4 (+1)	77 kDa	18.557	21.578	14.107	15.9	2.5144	31.415	4.9036	3.7596
Probable methylmalonate-semialdehyde dehydrogenase [acylating], mitochondrial OS=Bombus vosnesenskii OX=207650 GN=LOC117233535 PE=3 SV=1	A0A6J3K9M5	56 kDa	22.591	18.496	15.767	11.66	6.7051	15.708	6.1295	5.6394
thioredoxin reductase 2, mitochondrial isoform X1 OS=Bombus vosnesenskii OX=207650 GN=LOC117234026 PE=3 SV=1	A0A6J3KC81 (+2)	60 kDa	12.909	14.385	14.937	15.9	8.3814	15.708	15.937	16.918
Calreticulin OS=Bombus vosnesenskii OX=207650 GN=LOC117242401 PE=3 SV=1	A0A6J3LJP3	47 kDa	20.17	13.358	8.2985	7.4199	10.058	14.137	4.9036	1.8798
heat shock protein 60A OS=Bombus vosnesenskii OX=207650 GN=LOC117242836 PE=3 SV=1	A0A6J3LKG2	60 kDa	22.591	16.44	13.278	11.66	9.2196	1.5708	17.163	5.6394

ATP synthase subunit alpha OS=Bombus vosnesenskii OX=207650 GN=LOC117235069 PE=3 SV=1	A0A6J3KHN1	59 kDa	17.75	19.523	14.107	9.5399	3.3526	4.7123	8.5813	0
Aspartate aminotransferase OS=Bombus vosnesenskii OX=207650 GN=LOC117237467 PE=3 SV=1	A0A6J3KYQ6	48 kDa	15.329	10.275	14.937	9.5399	12.572	0	13.485	16.918
Citrate synthase OS=Bombus vosnesenskii OX=207650 GN=LOC117241831 PE=3 SV=1	A0A6J3LGS6	51 kDa	8.8749	8.2202	7.4687	8.4799	32.688	6.283	9.8072	9.399
ribonuclease Oy OS=Bombus vosnesenskii OX=207650 GN=LOC117235716 PE=3 SV=1	A0A6J3KPV6	32 kDa	13.716	10.275	12.448	14.84	13.41	12.566	9.8072	11.279
antichymotrypsin-2-like OS=Bombus vosnesenskii OX=207650 GN=LOC117231436 PE=3 SV=1	A0A6J3K005	45 kDa	8.0681	6.1652	5.809	5.2999	29.335	7.8538	7.3554	0
Dihydrolipoyl dehydrogenase OS=Bombus vosnesenskii OX=207650 GN=LOC117241790 PE=3 SV=1	A0A6J3LGI2	54 kDa	5.6477	20.551	13.278	19.08	14.248	0	14.711	11.279
lambda-crystallin homolog isoform X2 OS=Bombus vosnesenskii OX=207650 GN=LOC117232137 PE=3 SV=1	A0A6J3K4R3	36 kDa	14.523	6.1652	12.448	9.5399	9.2196	3.1415	19.614	3.7596
Phosphoglycerate kinase OS=Bombus vosnesenskii OX=207650 GN=LOC117239799 PE=3 SV=1	A0A6J3L8H2	45 kDa	16.943	12.33	11.618	8.4799	8.3814	9.4245	12.259	3.7596
Adenylosuccinate synthetase OS=Bombus vosnesenskii OX=207650 GN=LOC117243394 PE=3 SV=1	A0A6J3LMV0	49 kDa	8.8749	13.358	13.278	16.96	10.058	14.137	12.259	7.5192
phenoloxidase 1 OS=Bombus vosnesenskii OX=207650 GN=LOC117243143 PE=4 SV=1	A0A6J3LL92	80 kDa	14.523	10.275	10.788	12.72	15.087	10.995	11.033	1.8798
Superoxide dismutase [Cu-Zn] OS=Bombus vosnesenskii OX=207650	A0A6J3K7G9	18 kDa	12.102	10.275	9.9582	18.02	13.41	6.283	7.3554	13.159

GN=LOC117232686 PE=3 SV=1										
neprilysin-2 isoform X2 OS=Bombus vosnesenskii OX=207650 GN=LOC117242822 PE=3 SV=1	A0A6J3LJI2 (+2)	91 kDa	11.295	6.1652	11.618	8.4799	8.3814	12.566	13.485	5.6394
Cytosol aminopeptidase OS=Bombus vosnesenskii OX=207650 GN=LOC117238672 PE=3 SV=1	A0A6J3L338	55 kDa	8.8749	11.303	9.1284	6.3599	8.3814	4.7123	17.163	0
aldehyde dehydrogenase X, mitochondrial isoform X2 OS=Bombus vosnesenskii OX=207650 GN=LOC117242272 PE=3 SV=1	A0A6J3LGM 0 (+1)	53 kDa	18.557	7.1927	14.107	9.5399	10.058	7.8538	13.485	5.6394
probable tRNA (Guanine(26)-N(2))-dimethyltransferase OS=Bombus vosnesenskii OX=207650 GN=LOC117240164 PE=3 SV=1	A0A6J3L9F3	93 kDa	12.909	13.358	9.9582	13.78	18.439	1.5708	8.5813	0
sp|K1C15_SHEEP|	sp|K1C15_S HEEP|	49 kDa	12.102	12.33	11.618	10.6	5.867	0	9.8072	24.437
Aldose 1-epimerase OS=Bombus vosnesenskii OX=207650 GN=LOC117239793 PE=3 SV=1	A0A6J3L7B7 (+2)	41 kDa	8.8749	9.2478	18.257	12.72	5.867	20.42	8.5813	5.6394
arginine kinase OS=Bombus vosnesenskii OX=207650 GN=LOC117230705 PE=3 SV=1	A0A6J3JW3 5	42 kDa	16.136	15.413	14.937	7.4199	5.867	7.8538	8.5813	3.7596
14-3-3 protein zeta isoform X3 OS=Bombus vosnesenskii OX=207650 GN=LOC117234426 PE=3 SV=1	A0A6J3KE91 (+2)	28 kDa	11.295	11.303	9.1284	14.84	10.896	9.4245	7.3554	1.8798
L-fuconate dehydratase OS=Bombus vosnesenskii OX=207650 GN=LOC117238197 PE=4 SV=1	A0A6J3KZI9	51 kDa	12.909	9.2478	11.618	14.84	6.7051	12.566	12.259	13.159
4a-hydroxytetrahydrobiopterin dehydratase OS=Bombus vosnesenskii OX=207650 GN=LOC117242770 PE=3 SV=1	A0A6J3LJ85	16 kDa	10.489	8.2202	14.937	8.4799	5.867	15.708	8.5813	13.159

Diphosphomevalonate decarboxylase OS=Bombus vosnesenskii OX=207650 GN=LOC117236200 PE=3 SV=1	A0A6J3KP45	43 kDa	12.909	13.358	11.618	24.38	6.7051	3.1415	7.3554	5.6394
S-formylglutathione hydrolase OS=Bombus vosnesenskii OX=207650 GN=LOC117231879 PE=3 SV=1	A0A6J3K2Z5	32 kDa	10.489	13.358	12.448	16.96	9.2196	4.7123	8.5813	5.6394
xaa-Pro dipeptidase OS=Bombus vosnesenskii OX=207650 GN=LOC117231884 PE=4 SV=1	A0A6J3K308	55 kDa	12.102	9.2478	13.278	11.66	6.7051	18.849	8.5813	3.7596
N-acetylneuraminate lyase-like isoform X1 OS=Bombus vosnesenskii OX=207650 GN=LOC117235775 PE=3 SV=1	A0A6J3KLW3	33 kDa	16.943	9.2478	15.767	8.4799	8.3814	6.283	12.259	1.8798
protein D2-like isoform X1 OS=Bombus vosnesenskii OX=207650 GN=LOC117230745 PE=4 SV=1	A0A6J3JVN2 (+1)	23 kDa	12.102	7.1927	10.788	11.66	9.2196	14.137	6.1295	13.159
Mevalonate kinase OS=Bombus vosnesenskii OX=207650 GN=LOC117239257 PE=3 SV=1	A0A6J3L5P2	43 kDa	11.295	12.33	10.788	15.9	8.3814	6.283	8.5813	7.5192
uncharacterized protein LOC117235148 OS=Bombus vosnesenskii OX=207650 GN=LOC117235148 PE=4 SV=1	A0A6J3KI42	28 kDa	4.034	21.578	15.767	7.4199	5.0288	0	25.744	3.7596
cytochrome P450 6k1-like isoform X3 OS=Bombus vosnesenskii OX=207650 GN=LOC117242889 PE=3 SV=1	A0A6J3LPF3 (+1)	59 kDa	12.102	7.1927	10.788	13.78	6.7051	6.283	0	0
Superoxide dismutase OS=Bombus vosnesenskii OX=207650 GN=LOC117233036 PE=3 SV=1	A0A6J3K822	25 kDa	6.4545	9.2478	9.1284	11.66	12.572	12.566	6.1295	5.6394
Calponin OS=Bombus vosnesenskii OX=207650 GN=LOC117240277 PE=3 SV=1	A0A6J3LAE3	19 kDa	10.489	9.2478	10.788	11.66	6.7051	15.708	13.485	13.159
Carboxypeptidase OS=Bombus vosnesenskii OX=207650	A0A6J3JZZ8	54 kDa	14.523	10.275	8.2985	11.66	8.3814	9.4245	9.8072	13.159

GN=LOC117231762 PE=3 SV=1										
Vinculin OS=Bombus vosnesenskii OX=207650 GN=LOC117230649 PE=3 SV=1	A0A6J3JTP9 (+18)	118 kDa	14.523	14.385	13.278	13.78	4.1907	6.283	11.033	1.8798
neurotrimin-like OS=Bombus vosnesenskii OX=207650 GN=LOC117234800 PE=4 SV=1	A0A6J3KIX8	55 kDa	8.0681	11.303	12.448	9.5399	8.3814	17.278	7.3554	1.8798
lysosomal Pro-X carboxypeptidase OS=Bombus vosnesenskii OX=207650 GN=LOC117237864 PE=3 SV=1	A0A6J3L151	57 kDa	11.295	14.385	9.9582	12.72	5.867	18.849	7.3554	5.6394
glyoxylate reductase/hydroxypyruvate reductase-like OS=Bombus vosnesenskii OX=207650 GN=LOC117241767 PE=3 SV=1	A0A6J3LIS1	35 kDa	11.295	9.2478	13.278	8.4799	5.867	15.708	8.5813	7.5192
N-sulphoglucosamine sulphohydrolase OS=Bombus vosnesenskii OX=207650 GN=LOC117231385 PE=3 SV=1	A0A6J3JXV0	59 kDa	8.8749	11.303	12.448	12.72	3.3526	7.8538	13.485	16.918
carnitine O-palmitoyltransferase 2, mitochondrial OS=Bombus vosnesenskii OX=207650 GN=LOC117237850 PE=3 SV=1	A0A6J3KYT 6	76 kDa	11.295	10.275	9.9582	12.72	5.867	9.4245	7.3554	3.7596
cytochrome c OS=Bombus vosnesenskii OX=207650 GN=LOC117239742 PE=3 SV=1	A0A6J3L760	12 kDa	8.0681	9.2478	14.937	7.4199	8.3814	9.4245	7.3554	7.5192
probable elongation factor 1-delta isoform X1 OS=Bombus vosnesenskii OX=207650 GN=LOC117230147 PE=3 SV=1	A0A6J3JS28	48 kDa	8.8749	14.385	14.107	12.72	4.1907	6.283	9.8072	16.918
tenascin-like OS=Bombus vosnesenskii OX=207650 GN=LOC117240225 PE=4 SV=1	A0A6J3LBP4	69 kDa	3.2272	3.0826	2.4896	9.5399	29.335	0	0	1.8798
Fumarylacetoacetase OS=Bombus vosnesenskii OX=207650 GN=LOC117231648 PE=3 SV=1	A0A6J3K0Z5	46 kDa	7.2613	8.2202	9.9582	11.66	9.2196	14.137	7.3554	7.5192

twitchin OS=Bombus vosnesenskii OX=207650 GN=LOC117237692 PE=3 SV=1	A0A6J3KY12	1038 kDa	4.8408	8.2202	19.087	8.4799	9.2196	7.8538	7.3554	7.5192
gamma-interferon-inducible lysosomal thiol reductase-like OS=Bombus vosnesenskii OX=207650 GN=LOC117234916 PE=3 SV=1	A0A6J3KGZ4	25 kDa	10.489	9.2478	8.2985	9.5399	7.5433	12.566	13.485	9.399
aldo-keto reductase family 1 member B1-like OS=Bombus vosnesenskii OX=207650 GN=LOC117240154 PE=4 SV=1	A0A6J3L9M0	36 kDa	10.489	7.1927	9.1284	11.66	7.5433	7.8538	13.485	1.8798
Fatty acyl-CoA reductase OS=Bombus vosnesenskii OX=207650 GN=LOC117240703 PE=3 SV=1	A0A6J3LBA6	58 kDa	13.716	21.578	9.1284	12.72	4.1907	9.4245	1.2259	1.8798
H(+)-transporting two-sector ATPase OS=Bombus vosnesenskii OX=207650 GN=LOC117233993 PE=3 SV=1	A0A6J3KC07	68 kDa	11.295	12.33	12.448	1.06	9.2196	1.5708	0	0
Glutathione peroxidase OS=Bombus vosnesenskii OX=207650 GN=LOC117231056 PE=3 SV=1	A0A6J3JW58	23 kDa	12.909	9.2478	10.788	9.5399	5.0288	17.278	9.8072	3.7596
Xylulose kinase OS=Bombus vosnesenskii OX=207650 GN=LOC117230886 PE=3 SV=1	A0A6J3JX60	59 kDa	12.102	9.2478	6.6388	11.66	6.7051	3.1415	9.8072	1.8798
chymotrypsin inhibitor-like OS=Bombus vosnesenskii OX=207650 GN=LOC117240283 PE=4 SV=1	A0A6J3L993	8 kDa	1.6136	4.1101	4.1493	3.18	33.526	0	2.4518	3.7596
adenylate kinase isoenzyme 1 OS=Bombus vosnesenskii OX=207650 GN=LOC117236063 PE=3 SV=1	A0A6J3KN22	21 kDa	8.8749	8.2202	7.4687	10.6	7.5433	17.278	8.5813	11.279
glycerol-3-phosphate phosphatase OS=Bombus vosnesenskii OX=207650 GN=LOC117234387 PE=3 SV=1	A0A6J3KHP6	34 kDa	13.716	6.1652	7.4687	13.78	6.7051	1.5708	6.1295	5.6394

uncharacterized protein LOC117230889 OS=Bombus vosnesenskii OX=207650 GN=LOC117230889 PE=4 SV=1	A0A6J3JX64	53 kDa	4.034	4.1101	3.3194	4.2399	20.115	7.8538	0	0
Mesencephalic astrocyte-derived neurotrophic factor homolog OS=Bombus vosnesenskii OX=207650 GN=LOC117234342 PE=3 SV=1	A0A6J3KDT9	20 kDa	6.4545	6.1652	11.618	11.66	8.3814	4.7123	11.033	11.279
beta-mannosidase OS=Bombus vosnesenskii OX=207650 GN=LOC117239229 PE=3 SV=1	A0A6J3L9G7	184 kDa	7.2613	10.275	19.087	10.6	0.83814	10.995	8.5813	1.8798
Succinate--CoA ligase [ADP-forming] subunit beta, mitochondrial OS=Bombus vosnesenskii OX=207650 GN=LOC117230566 PE=3 SV=1	A0A6J3JTG9	49 kDa	11.295	9.2478	10.788	10.6	5.867	1.5708	7.3554	1.8798
farnesol dehydrogenase-like OS=Bombus vosnesenskii OX=207650 GN=LOC117238743 PE=3 SV=1	A0A6J3L2C4 (+2)	27 kDa	10.489	10.275	10.788	9.5399	6.7051	4.7123	12.259	7.5192
beta-1,3-glucan-binding protein 1-like OS=Bombus vosnesenskii OX=207650 GN=LOC117234110 PE=3 SV=1	A0A6J3KG34	54 kDa	11.295	5.1377	11.618	10.6	7.5433	12.566	9.8072	3.7596
Tubulin beta chain OS=Bombus vosnesenskii OX=207650 GN=LOC117232017 PE=3 SV=1	A0A6J3K442	50 kDa	14.523	16.44	9.9582	2.12	1.6763	1.5708	0	0
hexamerin-like OS=Bombus vosnesenskii OX=207650 GN=LOC117236575 PE=4 SV=1	A0A6J3KR03	81 kDa	11.295	4.1101	10.788	5.2999	14.248	10.995	8.5813	5.6394
antichymotrypsin-2-like OS=Bombus vosnesenskii OX=207650 GN=LOC117231371 PE=3 SV=1	A0A6J3JZC3	59 kDa	9.6817	0	8.2985	0	0	0	0	0
palmitoyl-protein thioesterase 1 OS=Bombus vosnesenskii OX=207650 GN=LOC117239379 PE=3 SV=1	A0A6J3LA43	35 kDa	9.6817	7.1927	7.4687	7.4199	6.7051	14.137	7.3554	13.159

Prostaglandin reductase 1 OS=Bombus vosnesenskii OX=207650 GN=LOC117232106 PE=3 SV=1	A0A6J3K3R2 (+1)	32 kDa	14.523	7.1927	9.9582	6.3599	6.7051	6.283	11.033	3.7596
6-phosphogluconate dehydrogenase, decarboxylating OS=Bombus vosnesenskii OX=207650 GN=LOC117241845 PE=3 SV=1	A0A6J3LEY8	53 kDa	8.0681	7.1927	9.1284	10.6	15.087	1.5708	6.1295	3.7596
filamin-A isoform X1 OS=Bombus vosnesenskii OX=207650 GN=LOC117242997 PE=3 SV=1	A0A6J3LKE3 (+3)	263 kDa	10.489	7.1927	9.1284	11.66	5.867	7.8538	3.6777	0
glyoxalase domain-containing protein 4 OS=Bombus vosnesenskii OX=207650 GN=LOC117232230 PE=3 SV=1	A0A6J3K2N8	32 kDa	6.4545	5.1377	6.6388	10.6	6.7051	14.137	7.3554	13.159
Adenylyl cyclase-associated protein OS=Bombus vosnesenskii OX=207650 GN=LOC117233624 PE=3 SV=1	A0A6J3KA49 (+2)	58 kDa	8.8749	5.1377	9.9582	8.4799	7.5433	12.566	7.3554	3.7596
NADPH--cytochrome P450 reductase OS=Bombus vosnesenskii OX=207650 GN=LOC117236438 PE=3 SV=1	A0A6J3KQE9	79 kDa	12.909	5.1377	9.9582	1.06	8.3814	7.8538	2.4518	1.8798
elongation factor 1-gamma OS=Bombus vosnesenskii OX=207650 GN=LOC117231394 PE=4 SV=1	A0A6J3JXX8	49 kDa	9.6817	9.2478	8.2985	10.6	8.3814	6.283	6.1295	5.6394
Fatty acyl-CoA reductase OS=Bombus vosnesenskii OX=207650 GN=LOC117240706 PE=3 SV=1	A0A6J3LD66	57 kDa	12.909	21.578	10.788	9.5399	1.6763	9.4245	1.2259	0
NADH-cytochrome b5 reductase OS=Bombus vosnesenskii OX=207650 GN=LOC117233094 PE=3 SV=1	A0A6J3K764 (+2)	34 kDa	8.0681	9.2478	8.2985	10.6	5.867	7.8538	3.6777	3.7596
sodium/calcium exchanger regulatory protein 1 isoform X1 OS=Bombus vosnesenskii OX=207650	A0A6J3KUY4	15 kDa	8.0681	11.303	9.9582	10.6	5.0288	9.4245	3.6777	3.7596

GN=LOC117236795 PE=3 SV=1										
Acyl-coenzyme A oxidase OS=Bombus vosnesenskii OX=207650 GN=LOC117230767 PE=3 SV=1	A0A6J3JVS6	76 kDa	12.102	11.303	4.9791	11.66	11.734	1.5708	4.9036	0
peroxiredoxin-like OS=Bombus vosnesenskii OX=207650 GN=LOC117233967 PE=4 SV=1	A0A6J3KBU 5	27 kDa	8.0681	8.2202	6.6388	4.2399	8.3814	10.995	9.8072	11.279
tetratricopeptide repeat protein 27-like isoform X2 OS=Bombus vosnesenskii OX=207650 GN=LOC117233658 PE=3 SV=1	A0A6J3KBE 9 (+1)	111 kDa	9.6817	8.2202	9.1284	7.4199	6.7051	10.995	7.3554	3.7596
Angiotensin-converting enzyme OS=Bombus vosnesenskii OX=207650 GN=LOC117235883 PE=3 SV=1	A0A6J3KQ3 4	74 kDa	12.102	8.2202	10.788	10.6	4.1907	4.7123	13.485	3.7596
clavesin-2 OS=Bombus vosnesenskii OX=207650 GN=LOC117236343 PE=4 SV=1	A0A6J3KPW 1	33 kDa	11.295	11.303	7.4687	10.6	3.3526	3.1415	6.1295	3.7596
slit homolog 3 protein-like OS=Bombus vosnesenskii OX=207650 GN=LOC117235722 PE=4 SV=1	A0A6J3KPW 0	31 kDa	3.2272	0	5.809	0	35.202	3.1415	2.4518	13.159
aldo-keto reductase family 1 member B1-like isoform X2 OS=Bombus vosnesenskii OX=207650 GN=LOC117240156 PE=4 SV=1	A0A6J3L8I9 (+1)	36 kDa	8.0681	0	9.9582	9.5399	0	0	0	0
CD109 antigen-like isoform X2 OS=Bombus vosnesenskii OX=207650 GN=LOC117235688 PE=3 SV=1	A0A6J3KKY 8 (+3)	171 kDa	11.295	6.1652	12.448	4.2399	5.0288	10.995	3.6777	3.7596
Elongation factor 1-alpha OS=Bombus vosnesenskii OX=207650 GN=LOC117239077 PE=3 SV=1	A0A6J3L6U0	50 kDa	10.489	11.303	9.9582	6.3599	5.0288	6.283	6.1295	1.8798
venom protease-like isoform X1 OS=Bombus vosnesenskii OX=207650	A0A6J3KU6 5 (+1)	32 kDa	4.8408	9.2478	3.3194	6.3599	14.248	4.7123	8.5813	1.8798

GN=LOC117237097 PE=4 SV=1										
Adenosylhomocysteinase OS=Bombus vosnesenskii OX=207650 GN=LOC117237425 PE=3 SV=1	A0A6J3KWE5	48 kDa	9.6817	9.2478	10.788	3.18	7.5433	4.7123	7.3554	3.7596
Putative hydroxypyruvate isomerase OS=Bombus vosnesenskii OX=207650 GN=LOC117238498 PE=3 SV=1	A0A6J3L4E6	30 kDa	11.295	7.1927	7.4687	7.4199	6.7051	7.8538	4.9036	1.8798
calexcitin-2 OS=Bombus vosnesenskii OX=207650 GN=LOC117232823 PE=4 SV=1	A0A6J3K5N6	21 kDa	13.716	9.2478	8.2985	11.66	3.3526	3.1415	7.3554	3.7596
testicular acid phosphatase homolog isoform X2 OS=Bombus vosnesenskii OX=207650 GN=LOC117243362 PE=4 SV=1	A0A6J3LRI5 (+3)	45 kDa	9.6817	12.33	7.4687	11.66	2.5144	10.995	6.1295	5.6394
ras-related protein Rac1 OS=Bombus vosnesenskii OX=207650 GN=LOC117239168 PE=4 SV=1	A0A6J3L7B3	21 kDa	5.6477	12.33	5.809	8.4799	7.5433	12.566	4.9036	3.7596
uncharacterized protein LOC117230546 OS=Bombus vosnesenskii OX=207650 GN=LOC117230546 PE=4 SV=1	A0A6J3JUJ9	59 kDa	12.102	5.1377	14.937	8.4799	3.3526	1.5708	11.033	0
Acyl-coenzyme A oxidase OS=Bombus vosnesenskii OX=207650 GN=LOC117230768 PE=3 SV=1	A0A6J3JUC6	77 kDa	13.716	0	4.9791	0	10.896	0	6.1295	0
phosphoribosylformylglycinamidine synthase OS=Bombus vosnesenskii OX=207650 GN=LOC117241660 PE=3 SV=1	A0A6J3LI08	147 kDa	8.0681	5.1377	6.6388	7.4199	5.867	3.1415	2.4518	5.6394
peptidylprolyl isomerase OS=Bombus vosnesenskii OX=207650 GN=LOC117241957 PE=4 SV=1	A0A6J3LHL2	12 kDa	5.6477	6.1652	6.6388	9.5399	10.058	9.4245	6.1295	3.7596
Fatty acyl-CoA reductase OS=Bombus vosnesenskii OX=207650	A0A6J3LBT7	57 kDa	15.329	15.413	10.788	0	0	6.283	0	0

GN=LOC117240705 PE=3 SV=1										
Epoxide hydrolase OS=Bombus vosnesenskii OX=207650 GN=LOC117234154 PE=3 SV=1	A0A6J3KD28	52 kDa	8.0681	9.2478	6.6388	10.6	5.0288	7.8538	7.3554	3.7596
Carboxylic ester hydrolase OS=Bombus vosnesenskii OX=207650 GN=LOC117235060 PE=3 SV=1	A0A6J3KK13	58 kDa	12.909	5.1377	9.9582	7.4199	2.5144	6.283	12.259	3.7596
fasciclin-2 isoform X3 OS=Bombus vosnesenskii OX=207650 GN=LOC117233646 PE=4 SV=1	A0A6J3KA86 (+5)	100 kDa	10.489	9.2478	9.1284	6.3599	1.6763	10.995	3.6777	3.7596
LOW QUALITY PROTEIN: peptidoglycan recognition protein 3-like OS=Bombus vosnesenskii OX=207650 GN=LOC117233578 PE=3 SV=1	A0A6J3K9X4	46 kDa	9.6817	6.1652	7.4687	6.3599	4.1907	12.566	8.5813	7.5192
EH domain-containing protein 3 OS=Bombus vosnesenskii OX=207650 GN=LOC117231106 PE=4 SV=1	A0A6J3JY90	107 kDa	7.2613	6.1652	7.4687	6.3599	5.0288	4.7123	12.259	3.7596
Pyridoxal kinase OS=Bombus vosnesenskii OX=207650 GN=LOC117234550 PE=3 SV=1	A0A6J3KFW6	33 kDa	8.8749	8.2202	9.1284	5.2999	5.0288	7.8538	6.1295	5.6394
peptidylprolyl isomerase OS=Bombus vosnesenskii OX=207650 GN=LOC117239367 PE=4 SV=1	A0A6J3L563 (+1)	45 kDa	9.6817	9.2478	5.809	9.5399	4.1907	3.1415	8.5813	1.8798
guanine nucleotide-binding protein G(I)/G(S)/G(T) subunit beta-1 OS=Bombus vosnesenskii OX=207650 GN=LOC117243391 PE=3 SV=1	A0A6J3LRL8	37 kDa	6.4545	6.1652	7.4687	8.4799	7.5433	1.5708	7.3554	0
Alpha-galactosidase OS=Bombus vosnesenskii OX=207650 GN=LOC117241661 PE=3 SV=1	A0A6J3LD28 (+1)	50 kDa	7.2613	6.1652	7.4687	6.3599	3.3526	6.283	8.5813	5.6394
vacuolar segregation protein 7-like OS=Bombus vosnesenskii OX=207650	A0A6J3K883	26 kDa	7.2613	9.2478	7.4687	6.3599	4.1907	10.995	3.6777	5.6394

GN=LOC117232986 PE=4 SV=1										
vesicle-fusing ATPase OS=Bombus vosnesenskii OX=207650 GN=LOC117239275 PE=4 SV=1	A0A6J3L7V0	89 kDa	10.489	6.1652	5.809	6.3599	5.867	6.283	7.3554	1.8798
regulator of chromosome condensation isoform X2 OS=Bombus vosnesenskii OX=207650 GN=LOC117239731 PE=4 SV=1	A0A6J3L8A3 (+1)	46 kDa	4.8408	6.1652	6.6388	7.4199	10.896	1.5708	1.2259	1.8798
V-type proton ATPase subunit C OS=Bombus vosnesenskii OX=207650 GN=LOC117237209 PE=3 SV=1	A0A6J3KV38	45 kDa	5.6477	7.1927	6.6388	4.2399	7.5433	4.7123	3.6777	5.6394
myrosinase 1 OS=Bombus vosnesenskii OX=207650 GN=LOC117233446 PE=3 SV=1	A0A6J3K967	57 kDa	7.2613	5.1377	9.9582	6.3599	5.867	10.995	3.6777	3.7596
apoptosis-inducing factor 3-like isoform X2 OS=Bombus vosnesenskii OX=207650 GN=LOC117234983 PE=4 SV=1	A0A6J3KKW3 (+1)	64 kDa	11.295	9.2478	5.809	9.5399	1.6763	1.5708	9.8072	5.6394
uncharacterized protein LOC117233786 OS=Bombus vosnesenskii OX=207650 GN=LOC117233786 PE=4 SV=1	A0A6J3KBU7	49 kDa	12.909	13.358	9.9582	2.12	0.83814	9.4245	1.2259	1.8798
Electron transfer flavoprotein subunit alpha OS=Bombus vosnesenskii OX=207650 GN=LOC117237998 PE=3 SV=1	A0A6J3KZ37	35 kDa	8.0681	11.303	8.2985	8.4799	2.5144	1.5708	1.2259	0
10-formyltetrahydrofolate dehydrogenase OS=Bombus vosnesenskii OX=207650 GN=LOC117242354 PE=3 SV=1	A0A6J3LLN7	100 kDa	11.295	5.1377	8.2985	5.2999	4.1907	4.7123	0	0
Carboxypeptidase Q OS=Bombus vosnesenskii OX=207650 GN=LOC117230722 PE=4 SV=1	A0A6J3JU92 (+1)	64 kDa	7.2613	5.1377	6.6388	5.2999	5.0288	9.4245	12.259	7.5192
GDP-L-fucose synthase OS=Bombus vosnesenskii OX=207650	A0A6J3LJ88	37 kDa	11.295	3.0826	6.6388	5.2999	3.3526	7.8538	4.9036	3.7596

GN=LOC117241847 PE=3 SV=1										
pyrimidodiazepine synthase-like OS=Bombus vosnesenskii OX=207650 GN=LOC117236127 PE=3 SV=1	A0A6J3KN94	28 kDa	7.2613	9.2478	7.4687	7.4199	4.1907	9.4245	3.6777	1.8798
Alpha-1,4 glucan phosphorylase OS=Bombus vosnesenskii OX=207650 GN=LOC117234120 PE=3 SV=1	A0A6J3KF07 (+1)	97 kDa	6.4545	4.1101	6.6388	5.2999	9.2196	9.4245	3.6777	1.8798
Acetyl-CoA hydrolase OS=Bombus vosnesenskii OX=207650 GN=LOC117238640 PE=3 SV=1	A0A6J3L6G0 (+1)	47 kDa	9.6817	16.44	5.809	3.18	5.0288	1.5708	4.9036	3.7596
Dolichyl-diphosphooligosaccharide--protein glycosyltransferase subunit 2 OS=Bombus vosnesenskii OX=207650 GN=LOC117234521 PE=3 SV=1	A0A6J3KHD6	73 kDa	11.295	8.2202	9.1284	4.2399	4.1907	4.7123	2.4518	0
nidogen isoform X4 OS=Bombus vosnesenskii OX=207650 GN=LOC117231978 PE=4 SV=1	A0A6J3K1E9 (+1)	144 kDa	7.2613	9.2478	10.788	4.2399	1.6763	7.8538	0	0
Annexin OS=Bombus vosnesenskii OX=207650 GN=LOC117239308 PE=3 SV=1	A0A6J3L4Z2	36 kDa	5.6477	4.1101	3.3194	0	7.5433	4.7123	0	0
Transgelin OS=Bombus vosnesenskii OX=207650 GN=LOC117240061 PE=3 SV=1	A0A6J3L916 (+1)	21 kDa	6.4545	5.1377	6.6388	6.3599	5.0288	15.708	2.4518	3.7596
uncharacterized protein C05D11.1-like OS=Bombus vosnesenskii OX=207650 GN=LOC117242775 PE=4 SV=1	A0A6J3LJ97	117 kDa	9.6817	4.1101	5.809	8.4799	5.0288	9.4245	2.4518	5.6394
Succinate--CoA ligase [GDP-forming] subunit beta, mitochondrial OS=Bombus vosnesenskii OX=207650 GN=LOC117242998 PE=3 SV=1	A0A6J3LKT3 (+1)	185 kDa	7.2613	8.2202	9.9582	6.3599	2.5144	4.7123	4.9036	3.7596
cytochrome P450 4g15 OS=Bombus vosnesenskii	A0A6J3L2K0	63 kDa	9.6817	11.303	7.4687	2.12	3.3526	9.4245	1.2259	1.8798

OX=207650 GN=LOC117238578 PE=3 SV=1										
3-hydroxyisobutyryl-CoA hydrolase, mitochondrial OS=Bombus vosnesenskii OX=207650 GN=LOC117230463 PE=4 SV=1	A0A6J3JSV4 (+2)	51 kDa	10.489	5.1377	11.618	6.3599	4.1907	4.7123	3.6777	0
LOW QUALITY PROTEIN: titin OS=Bombus vosnesenskii OX=207650 GN=LOC117242036 PE=3 SV=1	A0A6J3LFW 1	2183 kDa	0.80681	1.0275	23.236	1.06	9.2196	1.5708	4.9036	0
digestive cysteine proteinase 1 OS=Bombus vosnesenskii OX=207650 GN=LOC117238749 PE=3 SV=1	A0A6J3L714	62 kDa	8.8749	7.1927	6.6388	4.2399	5.867	7.8538	2.4518	3.7596
ras-related protein Rab-1A OS=Bombus vosnesenskii OX=207650 GN=LOC117239216 PE=4 SV=1	A0A6J3L7L5	23 kDa	8.0681	8.2202	4.1493	3.18	9.2196	4.7123	3.6777	0
serine protease inhibitor 88Ea-like isoform X2 OS=Bombus vosnesenskii OX=207650 GN=LOC117233673 PE=3 SV=1	A0A6J3KBG 4 (+1)	50 kDa	3.2272	2.0551	3.3194	2.12	15.925	0	1.2259	0
Cytochrome c oxidase subunit 4 OS=Bombus vosnesenskii OX=207650 GN=LOC117233324 PE=3 SV=1	A0A6J3K8G 3	23 kDa	8.8749	10.275	5.809	1.06	0	12.566	0	0
glutamate decarboxylase 1 OS=Bombus vosnesenskii OX=207650 GN=LOC117230530 PE=3 SV=1	A0A6J3JTC6	55 kDa	9.6817	9.2478	7.4687	7.4199	1.6763	6.283	4.9036	3.7596
Sterol carrier protein 2 OS=Bombus vosnesenskii OX=207650 GN=LOC117236065 PE=3 SV=1	A0A6J3KNF 4	59 kDa	6.4545	14.385	5.809	7.4199	2.5144	7.8538	2.4518	1.8798
isopentenyl-diphosphate Delta-isomerase OS=Bombus vosnesenskii OX=207650 GN=LOC117239147 PE=3 SV=1	A0A6J3L543	28 kDa	10.489	1.0275	0.82985	7.4199	11.734	4.7123	7.3554	1.8798

endoplasmic reticulum junction formation protein lunapark-A isoform X2 OS=Bombus vosnesenskii OX=207650 GN=LOC117237258 PE=4 SV=1	A0A6J3KU64 (+1)	67 kDa	4.8408	5.1377	6.6388	9.5399	5.867	7.8538	4.9036	0
ADP/ATP translocase OS=Bombus vosnesenskii OX=207650 GN=LOC117233403 PE=3 SV=1	A0A6J3KAQ1	33 kDa	5.6477	8.2202	7.4687	2.12	1.6763	6.283	0	0
enoyl-CoA delta isomerase 1, mitochondrial-like isoform X1 OS=Bombus vosnesenskii OX=207650 GN=LOC117233157 PE=4 SV=1	A0A6J3K9A3 (+1)	31 kDa	7.2613	10.275	6.6388	10.6	2.5144	4.7123	3.6777	3.7596
palmitoyl-protein hydrolase OS=Bombus vosnesenskii OX=207650 GN=LOC117234497 PE=3 SV=1	A0A6J3KFL0	20 kDa	3.2272	5.1377	5.809	7.4199	8.3814	1.5708	3.6777	1.8798
Isovaleryl-CoA dehydrogenase, mitochondrial OS=Bombus vosnesenskii OX=207650 GN=LOC117234547 PE=3 SV=1	A0A6J3KF18	47 kDa	9.6817	7.1927	4.9791	8.4799	5.0288	0	4.9036	1.8798
putative aminopeptidase-2 isoform X1 OS=Bombus vosnesenskii OX=207650 GN=LOC117233796 PE=3 SV=1	A0A6J3KAY4 (+1)	313 kDa	12.909	4.1101	6.6388	3.18	3.3526	6.283	2.4518	0
2-iminobutanoate/2-iminopropanoate deaminase OS=Bombus vosnesenskii OX=207650 GN=LOC117233713 PE=3 SV=1	A0A6J3KAM0	15 kDa	7.2613	5.1377	9.1284	6.3599	5.0288	4.7123	4.9036	3.7596
ubiquitin-conjugating enzyme E2-17 kDa OS=Bombus vosnesenskii OX=207650 GN=LOC117233838 PE=3 SV=1	A0A6J3KB77	17 kDa	7.2613	4.1101	6.6388	6.3599	6.7051	1.5708	2.4518	7.5192
Ferritin OS=Bombus vosnesenskii OX=207650 GN=LOC117242978 PE=3 SV=1	A0A6J3LL74	25 kDa	8.8749	5.1377	4.1493	1.06	5.867	7.8538	7.3554	3.7596
phenoloxidase-activating factor 2-like isoform X1 OS=Bombus vosnesenskii OX=207650	A0A6J3KIR2 (+1)	47 kDa	8.0681	5.1377	4.1493	1.06	9.2196	6.283	2.4518	1.8798

GN=LOC117235288 PE=4 SV=1										
actin-interacting protein 1 isoform X1 OS=Bombus vosnesenskii OX=207650 GN=LOC117233927 PE=4 SV=1	A0A6J3KF08 (+1)	66 kDa	8.8749	6.1652	6.6388	7.4199	2.5144	0	4.9036	1.8798
geranylgeranyl pyrophosphate synthase OS=Bombus vosnesenskii OX=207650 GN=LOC117237044 PE=3 SV=1	A0A6J3KXJ7	36 kDa	4.8408	5.1377	0.82985	14.84	6.7051	9.4245	8.5813	0
ras-like protein 2 isoform X1 OS=Bombus vosnesenskii OX=207650 GN=LOC117236480 PE=4 SV=1	A0A6J3KU39	23 kDa	6.4545	6.1652	7.4687	9.5399	2.5144	1.5708	6.1295	0
cytochrome P450 9e2-like OS=Bombus vosnesenskii OX=207650 GN=LOC117243321 PE=3 SV=1	A0A6J3LPB8	61 kDa	7.2613	10.275	5.809	7.4199	3.3526	0	3.6777	0
enoyl-[acyl-carrier-protein] reductase, mitochondrial isoform X1 OS=Bombus vosnesenskii OX=207650 GN=LOC117241787 PE=4 SV=1	A0A6J3LIU8	41 kDa	4.8408	6.1652	4.9791	5.2999	5.0288	9.4245	7.3554	5.6394
tubulin polymerization-promoting protein homolog OS=Bombus vosnesenskii OX=207650 GN=LOC117240160 PE=3 SV=1	A0A6J3L8J3	25 kDa	2.4204	6.1652	4.9791	7.4199	7.5433	9.4245	4.9036	1.8798
cofilin/actin-depolymerizing factor homolog isoform X1 OS=Bombus vosnesenskii OX=207650 GN=LOC117236984 PE=3 SV=1	A0A6J3KX03	17 kDa	5.6477	7.1927	4.1493	3.18	8.3814	7.8538	3.6777	1.8798
delta(3,5)-Delta(2,4)-dienoyl-CoA isomerase, mitochondrial OS=Bombus vosnesenskii OX=207650 GN=LOC117237801 PE=3 SV=1	A0A6J3L0S6	34 kDa	7.2613	6.1652	5.809	3.18	5.867	1.5708	3.6777	1.8798
selenoprotein F OS=Bombus vosnesenskii OX=207650 GN=LOC117233169 PE=3 SV=1	A0A6J3K7M7	18 kDa	2.4204	2.0551	5.809	2.12	12.572	1.5708	7.3554	3.7596

E1 ubiquitin-activating enzyme OS=Bombus vosnesenskii OX=207650 GN=LOC117235496 PE=3 SV=1	A0A6J3KKI9	117 kDa	7.2613	4.1101	7.4687	4.2399	4.1907	12.566	2.4518	3.7596
Sorbitol dehydrogenase OS=Bombus vosnesenskii OX=207650 GN=LOC117233279 PE=3 SV=1	A0A6J3K9X7	38 kDa	8.0681	8.2202	5.809	4.2399	4.1907	6.283	1.2259	1.8798
low-density lipoprotein receptor-related protein 2 OS=Bombus vosnesenskii OX=207650 GN=LOC117242508 PE=4 SV=1	A0A6J3LKB7	515 kDa	4.034	10.275	7.4687	2.12	3.3526	4.7123	1.2259	0
Sulfhydryl oxidase OS=Bombus vosnesenskii OX=207650 GN=LOC117231249 PE=3 SV=1	A0A6J3JZ28	75 kDa	9.6817	9.2478	9.1284	8.4799	0.83814	4.7123	3.6777	0
hypoxia up-regulated protein 1 isoform X2 OS=Bombus vosnesenskii OX=207650 GN=LOC117230237 PE=3 SV=1	A0A6J3JT79	106 kDa	8.0681	10.275	9.1284	3.18	2.5144	3.1415	1.2259	0
mitochondrial-processing peptidase subunit beta OS=Bombus vosnesenskii OX=207650 GN=LOC117238235 PE=3 SV=1	A0A6J3KZV1	53 kDa	5.6477	9.2478	5.809	1.06	4.1907	3.1415	0	0
LOW QUALITY PROTEIN: fasciclin-3 OS=Bombus vosnesenskii OX=207650 GN=LOC117241615 PE=4 SV=1	A0A6J3LDU2	64 kDa	4.8408	6.1652	5.809	8.4799	5.0288	0	3.6777	1.8798
very-long-chain 3-oxoacyl-CoA reductase-like OS=Bombus vosnesenskii OX=207650 GN=LOC117233106 PE=3 SV=1	A0A6J3K7B3	36 kDa	9.6817	10.275	4.9791	5.2999	1.6763	1.5708	2.4518	0
isochorismatase domain-containing protein 2-like OS=Bombus vosnesenskii OX=207650 GN=LOC117231714 PE=3 SV=1	A0A6J3K1Y8	22 kDa	4.034	5.1377	6.6388	6.3599	4.1907	4.7123	4.9036	5.6394
Protein quiver OS=Bombus vosnesenskii OX=207650 GN=LOC117243557 PE=3 SV=1	A0A6J3LPG0	17 kDa	5.6477	4.1101	4.9791	5.2999	4.1907	3.1415	2.4518	5.6394

Alpha-mannosidase OS=Bombus vosnesenskii OX=207650 GN=LOC117235663 PE=3 SV=1	A0A6J3KKT7 (+1)	114 kDa	8.8749	5.1377	9.9582	0	4.1907	4.7123	2.4518	3.7596
succinate-semialdehyde dehydrogenase [NADP(+)] GabD OS=Bombus vosnesenskii OX=207650 GN=LOC117230218 PE=4 SV=1	A0A6J3JR58	56 kDa	7.2613	6.1652	6.6388	5.2999	2.5144	7.8538	3.6777	0
protein CREG1 OS=Bombus vosnesenskii OX=207650 GN=LOC117241680 PE=4 SV=1	A0A6J3LED1	33 kDa	5.6477	3.0826	4.9791	6.3599	6.7051	10.995	2.4518	3.7596
uncharacterized protein LOC117242277 OS=Bombus vosnesenskii OX=207650 GN=LOC117242277 PE=4 SV=1	A0A6J3LHI0	28 kDa	8.0681	5.1377	4.9791	5.2999	5.0288	4.7123	2.4518	3.7596
plastin-2 isoform X2 OS=Bombus vosnesenskii OX=207650 GN=LOC117234674 PE=4 SV=1	A0A6J3KFV5 (+1)	71 kDa	5.6477	4.1101	6.6388	7.4199	1.6763	9.4245	2.4518	7.5192
pyridoxal 5'-phosphate synthase OS=Bombus vosnesenskii OX=207650 GN=LOC117235120 PE=3 SV=1	A0A6J3KKD2	28 kDa	7.2613	3.0826	4.9791	4.2399	5.0288	4.7123	4.9036	0
probable chitinase 2 OS=Bombus vosnesenskii OX=207650 GN=LOC117243229 PE=4 SV=1	A0A6J3LNU9	45 kDa	2.4204	9.2478	4.1493	2.12	10.058	0	4.9036	0
ATP synthase subunit gamma OS=Bombus vosnesenskii OX=207650 GN=LOC117241769 PE=3 SV=1	A0A6J3LDW0	33 kDa	8.8749	9.2478	7.4687	4.2399	0.83814	0	1.2259	0
pyrimidine-specific ribonucleoside hydrolase RihA-like isoform X3 OS=Bombus vosnesenskii OX=207650 GN=LOC117236557 PE=3 SV=1	A0A6J3KQW3 (+2)	37 kDa	8.0681	2.0551	4.9791	3.18	4.1907	4.7123	8.5813	3.7596
lysosomal aspartic protease OS=Bombus vosnesenskii OX=207650	A0A6J3LHR5	42 kDa	4.8408	2.0551	3.3194	5.2999	6.7051	1.5708	11.033	7.5192

GN=LOC117242419 PE=3 SV=1										
uncharacterized protein LOC117231108 OS=Bombus vosnesenskii OX=207650 GN=LOC117231108 PE=3 SV=1	A0A6J3JXU1	92 kDa	8.0681	2.0551	4.9791	3.18	6.7051	3.1415	2.4518	0
Dihydrolipoamide acetyltransferase component of pyruvate dehydrogenase complex OS=Bombus vosnesenskii OX=207650 GN=LOC117233204 PE=3 SV=1	A0A6J3K8Z9	64 kDa	8.8749	7.1927	6.6388	1.06	4.1907	3.1415	1.2259	0
ras-related protein Rap1 OS=Bombus vosnesenskii OX=207650 GN=LOC117232864 PE=4 SV=1	A0A6J3K6Z5	21 kDa	4.034	5.1377	3.3194	6.3599	3.3526	6.283	4.9036	5.6394
esterase E4-like OS=Bombus vosnesenskii OX=207650 GN=LOC117235282 PE=4 SV=1	A0A6J3KLB0	64 kDa	5.6477	4.1101	5.809	4.2399	2.5144	4.7123	9.8072	5.6394
inositol-3-phosphate synthase OS=Bombus vosnesenskii OX=207650 GN=LOC117237148 PE=3 SV=1	A0A6J3KX50 (+1)	57 kDa	4.8408	2.0551	6.6388	4.2399	4.1907	4.7123	6.1295	5.6394
dynein assembly factor 5, axonemal isoform X2 OS=Bombus vosnesenskii OX=207650 GN=LOC117239165 PE=3 SV=1	A0A6J3L583 (+1)	119 kDa	5.6477	4.1101	4.1493	3.18	4.1907	10.995	6.1295	1.8798
phosphatidylinositol transfer protein alpha isoform OS=Bombus vosnesenskii OX=207650 GN=LOC117236864 PE=4 SV=1	A0A6J3KSQ 6	32 kDa	3.2272	4.1101	5.809	6.3599	3.3526	4.7123	4.9036	1.8798
Profilin OS=Bombus vosnesenskii OX=207650 GN=LOC117239432 PE=3 SV=1	A0A6J3L6I3	14 kDa	7.2613	6.1652	4.9791	6.3599	2.5144	4.7123	3.6777	0
guanine nucleotide-binding protein subunit beta-like protein OS=Bombus vosnesenskii OX=207650 GN=LOC117235788 PE=3 SV=1	A0A6J3KLG 9	36 kDa	4.8408	6.1652	5.809	4.2399	2.5144	4.7123	0	1.8798

Bee-milk protein OS=Bombus vosnesenskii OX=207650 GN=LOC117243318 PE=3 SV=1	A0A6J3LM61	48 kDa	4.034	3.0826	4.9791	5.2999	10.058	0	4.9036	0
Proteasome subunit beta OS=Bombus vosnesenskii OX=207650 GN=LOC117242950 PE=3 SV=1	A0A6J3LK81	29 kDa	6.4545	8.2202	6.6388	1.06	0	10.995	0	0
14-3-3 protein epsilon OS=Bombus vosnesenskii OX=207650 GN=LOC117232776 PE=3 SV=1	A0A6J3K834	29 kDa	0	6.1652	2.4896	0	5.867	0	3.6777	0
Glucosylceramidase OS=Bombus vosnesenskii OX=207650 GN=LOC117233260 PE=3 SV=1	A0A6J3K860	58 kDa	6.4545	4.1101	4.9791	3.18	5.867	1.5708	7.3554	1.8798
macro domain-containing protein CT2219-like isoform X1 OS=Bombus vosnesenskii OX=207650 GN=LOC117236147 PE=4 SV=1	A0A6J3KSC0 (+1)	30 kDa	9.6817	3.0826	3.3194	6.3599	2.5144	4.7123	3.6777	0
probable medium-chain specific acyl-CoA dehydrogenase, mitochondrial OS=Bombus vosnesenskii OX=207650 GN=LOC117233161 PE=3 SV=1	A0A6J3K9A8	46 kDa	8.8749	3.0826	4.1493	3.18	1.6763	4.7123	1.2259	0
heparanase-like OS=Bombus vosnesenskii OX=207650 GN=LOC117240191 PE=3 SV=1	A0A6J3L9X2	60 kDa	5.6477	4.1101	2.4896	5.2999	2.5144	14.137	3.6777	5.6394
RNA-binding protein lark isoform X4 OS=Bombus vosnesenskii OX=207650 GN=LOC117241627 PE=4 SV=1	A0A6J3LCX5 (+6)	38 kDa	4.8408	6.1652	3.3194	5.2999	2.5144	7.8538	4.9036	1.8798
dehydrogenase/reductase SDR family member 4 OS=Bombus vosnesenskii OX=207650 GN=LOC117233634 PE=4 SV=1	A0A6J3KA67	29 kDa	4.8408	5.1377	4.1493	5.2999	3.3526	9.4245	2.4518	7.5192
thioredoxin-like protein 1 OS=Bombus vosnesenskii OX=207650 GN=LOC117243430 PE=4 SV=1	A0A6J3LMV1	33 kDa	4.034	6.1652	4.9791	7.4199	3.3526	4.7123	1.2259	1.8798

Bleomycin hydrolase OS=Bombus vosnesenskii OX=207650 GN=LOC117235680 PE=3 SV=1	A0A6J3KNW1	52 kDa	7.2613	7.1927	3.3194	6.3599	1.6763	1.5708	7.3554	1.8798
folate gamma-glutamyl hydrolase OS=Bombus vosnesenskii OX=207650 GN=LOC117231476 PE=3 SV=1	A0A6J3K067	37 kDa	3.2272	6.1652	3.3194	5.2999	5.0288	1.5708	8.5813	1.8798
Electron transfer flavoprotein subunit beta OS=Bombus vosnesenskii OX=207650 GN=LOC117231261 PE=3 SV=1	A0A6J3JYP7	28 kDa	6.4545	6.1652	5.809	4.2399	1.6763	1.5708	6.1295	0
uncharacterized protein LOC117239581 isoform X2 OS=Bombus vosnesenskii OX=207650 GN=LOC117239581 PE=4 SV=1	A0A6J3LB71	75 kDa	11.295	5.1377	8.2985	2.12	0.83814	6.283	3.6777	0
LOW QUALITY PROTEIN: transmembrane protease serine 9-like OS=Bombus vosnesenskii OX=207650 GN=LOC117231737 PE=4 SV=1	A0A6J3K1G1	63 kDa	0	9.2478	6.6388	0	5.867	1.5708	6.1295	0
phosphoserine transaminase OS=Bombus vosnesenskii OX=207650 GN=LOC117232382 PE=3 SV=1	A0A6J3K4Y9	41 kDa	8.0681	6.1652	9.9582	5.2999	1.6763	0	0	0
Apolipoprotein D OS=Bombus vosnesenskii OX=207650 GN=LOC117232811 PE=3 SV=1	A0A6J3K6Q2	30 kDa	4.8408	5.1377	3.3194	6.3599	3.3526	3.1415	6.1295	3.7596
Calcyclin-binding protein OS=Bombus vosnesenskii OX=207650 GN=LOC117230128 PE=4 SV=1	A0A6J3JQX1	26 kDa	5.6477	6.1652	4.1493	4.2399	4.1907	3.1415	2.4518	3.7596
LOW QUALITY PROTEIN: protein Red-like OS=Bombus vosnesenskii OX=207650 GN=LOC117242386 PE=3 SV=1	A0A6J3LH70	102 kDa	5.6477	3.0826	3.3194	3.18	6.7051	0	4.9036	1.8798
Mediator of RNA polymerase II transcription subunit 20 OS=Bombus vosnesenskii OX=207650	A0A6J3L2P0 (+1)	55 kDa	6.4545	9.2478	6.6388	2.12	0.83814	6.283	1.2259	0

GN=LOC117238802 PE=3 SV=1										
uncharacterized protein LOC117243154 isoform X2 OS=Bombus vosnesenskii OX=207650 GN=LOC117243154 PE=4 SV=1	A0A6J3LM77 (+1)	16 kDa	4.034	4.1101	4.1493	5.2999	4.1907	1.5708	7.3554	7.5192
probable salivary secreted peptide OS=Bombus vosnesenskii OX=207650 GN=LOC117234813 PE=4 SV=1	A0A6J3KJ03	15 kDa	0	7.1927	8.2985	5.2999	3.3526	1.5708	11.033	1.8798
Calcium-transporting ATPase OS=Bombus vosnesenskii OX=207650 GN=LOC117241735 PE=3 SV=1	A0A6J3LE93 (+1)	112 kDa	4.8408	7.1927	7.4687	0	0	10.995	1.2259	1.8798
uncharacterized protein LOC117239136 OS=Bombus vosnesenskii OX=207650 GN=LOC117239136 PE=4 SV=1	A0A6J3L525	37 kDa	3.2272	0	0	2.12	20.115	1.5708	1.2259	1.8798
4-hydroxyphenylpyruvate dioxygenase OS=Bombus vosnesenskii OX=207650 GN=LOC117239655 PE=3 SV=1	A0A6J3LBH6	44 kDa	5.6477	3.0826	3.3194	3.18	6.7051	1.5708	4.9036	5.6394
Coronin OS=Bombus vosnesenskii OX=207650 GN=LOC117236453 PE=3 SV=1	A0A6J3KQJ2	55 kDa	4.8408	4.1101	5.809	4.2399	4.1907	3.1415	3.6777	1.8798
Heat shock protein 83 OS=Bombus vosnesenskii OX=207650 GN=LOC117235089 PE=3 SV=1	A0A6J3KHL4	84 kDa	8.8749	6.1652	3.3194	3.18	0	6.283	1.2259	0
40S ribosomal protein S3-like OS=Bombus vosnesenskii OX=207650 GN=LOC117236727 PE=3 SV=1	A0A6J3KRX5	27 kDa	5.6477	9.2478	5.809	1.06	0	4.7123	0	0
Peptidyl-prolyl cis-trans isomerase OS=Bombus vosnesenskii OX=207650 GN=LOC117238473 PE=3 SV=1	A0A6J3L215	18 kDa	8.0681	2.0551	5.809	3.18	1.6763	6.283	3.6777	1.8798
Delta-1-pyrroline-5-carboxylate synthase OS=Bombus vosnesenskii OX=207650	A0A6J3KSI1 (+1)	84 kDa	8.0681	7.1927	9.1284	0	0	1.5708	0	0

GN=LOC117236322 PE=3 SV=1										
ras-related protein Rab-35 OS=Bombus vosnesenskii OX=207650 GN=LOC117239605 PE=4 SV=1	A0A6J3L6G1	23 kDa	0	0	0	0	5.867	0	0	0
uncharacterized oxidoreductase YjmC-like OS=Bombus vosnesenskii OX=207650 GN=LOC117243183 PE=4 SV=1	A0A6J3LMC1	43 kDa	4.034	3.0826	4.9791	2.12	1.6763	4.7123	3.6777	5.6394
haloacid dehalogenase-like hydrolase domain-containing protein 2 isoform X1 OS=Bombus vosnesenskii OX=207650 GN=LOC117232284 PE=3 SV=1	A0A6J3K5K6	28 kDa	7.2613	2.0551	5.809	4.2399	2.5144	4.7123	1.2259	5.6394
dimethylargininase OS=Bombus vosnesenskii OX=207650 GN=LOC117236004 PE=3 SV=1	A0A6J3KN42 (+1)	32 kDa	3.2272	6.1652	4.1493	5.2999	3.3526	1.5708	3.6777	1.8798
ecdysteroid-regulated 16 kDa protein OS=Bombus vosnesenskii OX=207650 GN=LOC117230938 PE=3 SV=1	A0A6J3JXE0	17 kDa	3.2272	1.0275	3.3194	2.12	7.5433	6.283	0	7.5192
glucose-6-phosphate 1-epimerase OS=Bombus vosnesenskii OX=207650 GN=LOC117242678 PE=3 SV=1	A0A6J3LJ93 (+2)	32 kDa	4.034	2.0551	4.1493	5.2999	6.7051	0	3.6777	0
zinc carboxypeptidase-like OS=Bombus vosnesenskii OX=207650 GN=LOC117242130 PE=3 SV=1	A0A6J3LIC7	46 kDa	1.6136	7.1927	6.6388	1.06	5.867	0	6.1295	0
arrestin domain-containing protein 17 isoform X1 OS=Bombus vosnesenskii OX=207650 GN=LOC117234305 PE=3 SV=1	A0A6J3KDV9	44 kDa	4.034	4.1101	4.9791	3.18	3.3526	3.1415	2.4518	5.6394
lysosomal alpha-glucosidase-like OS=Bombus vosnesenskii OX=207650 GN=LOC117237021 PE=3 SV=1	A0A6J3KXG1	115 kDa	4.8408	5.1377	4.1493	6.3599	1.6763	7.8538	3.6777	1.8798

uncharacterized protein LOC117235293 OS=Bombus vosnesenskii OX=207650 GN=LOC117235293 PE=3 SV=1	A0A6J3KJC7	128 kDa	6.4545	1.0275	4.9791	3.18	3.3526	3.1415	7.3554	5.6394
ATP-dependent (S)-NAD(P)H-hydrate dehydratase OS=Bombus vosnesenskii OX=207650 GN=LOC117233515 PE=3 SV=1	A0A6J3KAN5 (+1)	36 kDa	4.8408	4.1101	5.809	5.2999	4.1907	1.5708	1.2259	1.8798
4-coumarate--CoA ligase 1-like OS=Bombus vosnesenskii OX=207650 GN=LOC117231690 PE=3 SV=1	A0A6J3K1U4	65 kDa	7.2613	3.0826	4.9791	3.18	2.5144	0	2.4518	1.8798
ATP synthase subunit b OS=Bombus vosnesenskii OX=207650 GN=LOC117237884 PE=3 SV=1	A0A6J3KXS1	28 kDa	9.6817	5.1377	4.9791	0	0.83814	0	0	0
elongation factor 1-beta' OS=Bombus vosnesenskii OX=207650 GN=LOC117235556 PE=3 SV=1	A0A6J3KKA6	25 kDa	4.8408	6.1652	4.9791	4.2399	3.3526	3.1415	2.4518	1.8798
ras-related protein Rab-5C OS=Bombus vosnesenskii OX=207650 GN=LOC117234098 PE=4 SV=1	A0A6J3KDJ0	23 kDa	3.2272	4.1101	4.1493	4.2399	2.5144	6.283	2.4518	3.7596
Tetraspanin OS=Bombus vosnesenskii OX=207650 GN=LOC117236013 PE=3 SV=1	A0A6J3KQU6 (+1)	26 kDa	4.8408	6.1652	3.3194	4.2399	3.3526	7.8538	1.2259	1.8798
neurexin-4 OS=Bombus vosnesenskii OX=207650 GN=LOC117232677 PE=3 SV=1	A0A6J3K4S8	143 kDa	4.034	4.1101	5.809	3.18	2.5144	3.1415	2.4518	1.8798
carbonic anhydrase 2-like OS=Bombus vosnesenskii OX=207650 GN=LOC117230637 PE=3 SV=1	A0A6J3JUX8	38 kDa	4.8408	1.0275	2.4896	3.18	6.7051	6.283	1.2259	7.5192
circadian clock-controlled protein-like OS=Bombus vosnesenskii OX=207650 GN=LOC117234046 PE=4 SV=1	A0A6J3KEJ0	28 kDa	5.6477	0	5.809	2.12	5.0288	4.7123	6.1295	1.8798

Succinate--CoA ligase [ADP/GDP-forming] subunit alpha, mitochondrial OS=Bombus vosnesenskii OX=207650 GN=LOC117232354 PE=3 SV=1	A0A6J3K3A9 (+1)	36 kDa	4.034	4.1101	4.9791	3.18	4.1907	1.5708	2.4518	0
neuroglian isoform X1 OS=Bombus vosnesenskii OX=207650 GN=LOC117232876 PE=4 SV=1	A0A6J3K5Z4 (+1)	139 kDa	4.8408	3.0826	6.6388	3.18	4.1907	3.1415	0	0
Vacuolar proton pump subunit B OS=Bombus vosnesenskii OX=207650 GN=LOC117238668 PE=3 SV=1	A0A6J3L6L5	55 kDa	4.8408	6.1652	4.9791	0	3.3526	0	1.2259	0
thyrotropin-releasing hormone-degrading ectoenzyme-like isoform X1 OS=Bombus vosnesenskii OX=207650 GN=LOC117242942 PE=3 SV=1	A0A6J3LL15 (+1)	123 kDa	10.489	9.2478	6.6388	0	0	4.7123	0	0
lamin Dm0-like isoform X2 OS=Bombus vosnesenskii OX=207650 GN=LOC117232242 PE=3 SV=1	A0A6J3K2S2 (+1)	69 kDa	2.4204	5.1377	3.3194	4.2399	1.6763	3.1415	7.3554	1.8798
glyoxylate reductase/hydroxypyruvate reductase OS=Bombus vosnesenskii OX=207650 GN=LOC117231482 PE=3 SV=1	A0A6J3JYC3	40 kDa	2.4204	5.1377	3.3194	3.18	4.1907	1.5708	4.9036	1.8798
protein disulfide-isomerase OS=Bombus vosnesenskii OX=207650 GN=LOC117233733 PE=3 SV=1	A0A6J3KAQ 0	47 kDa	4.034	4.1101	2.4896	4.2399	5.867	3.1415	1.2259	0
Acetyltransferase component of pyruvate dehydrogenase complex OS=Bombus vosnesenskii OX=207650 GN=LOC117242230 PE=3 SV=1	A0A6J3LGV 9	53 kDa	4.034	6.1652	4.1493	1.06	3.3526	1.5708	1.2259	0
RNA helicase OS=Bombus vosnesenskii OX=207650 GN=LOC117236593 PE=3 SV=1	A0A6J3KQI8	48 kDa	7.2613	5.1377	4.9791	1.06	0.83814	1.5708	0	0

uncharacterized protein LOC117237750 OS=Bombus vosnesenskii OX=207650 GN=LOC117237750 PE=4 SV=1	A0A6J3L1E8	496 kDa	8.8749	3.0826	9.1284	0	0.83814	3.1415	0	0
phospholipase A1 OS=Bombus vosnesenskii OX=207650 GN=LOC117231169 PE=3 SV=1	A0A6J3JWI4	32 kDa	3.2272	2.0551	1.6597	2.12	15.925	0	0	0
Nicotinate phosphoribosyltransferase OS=Bombus vosnesenskii OX=207650 GN=LOC117230233 PE=3 SV=1	A0A6J3JRB7 (+1)	68 kDa	6.4545	4.1101	4.9791	3.18	1.6763	1.5708	6.1295	1.8798
ADP-ribosylation factor OS=Bombus vosnesenskii OX=207650 GN=LOC117233900 PE=3 SV=1	A0A6J3KDM 2	21 kDa	4.034	3.0826	1.6597	2.12	6.7051	1.5708	1.2259	1.8798
L-lactate dehydrogenase OS=Bombus vosnesenskii OX=207650 GN=LOC117235077 PE=3 SV=1	A0A6J3KHP 1 (+1)	36 kDa	4.034	2.0551	3.3194	4.2399	4.1907	0	6.1295	1.8798
peptidylprolyl isomerase OS=Bombus vosnesenskii OX=207650 GN=LOC117233209 PE=4 SV=1	A0A6J3K7X6 (+1)	26 kDa	3.2272	4.1101	4.1493	3.18	5.867	1.5708	0	1.8798
Guanine deaminase OS=Bombus vosnesenskii OX=207650 GN=LOC117234830 PE=3 SV=1	A0A6J3KJ39	48 kDa	4.8408	4.1101	4.1493	3.18	4.1907	0	4.9036	0
uncharacterized protein LOC117236850 OS=Bombus vosnesenskii OX=207650 GN=LOC117236850 PE=4 SV=1	A0A6J3KW2 6	21 kDa	3.2272	6.1652	2.4896	1.06	4.1907	14.137	0	0
Glutamine synthetase OS=Bombus vosnesenskii OX=207650 GN=LOC117231877 PE=3 SV=1	A0A6J3K0Q 0	41 kDa	7.2613	7.1927	6.6388	1.06	0	4.7123	0	0
cytochrome b-c1 complex subunit 2, mitochondrial OS=Bombus vosnesenskii OX=207650	A0A6J3K1E4	47 kDa	5.6477	5.1377	7.4687	0	0	3.1415	0	0

GN=LOC117231957 PE=4 SV=1										
UV excision repair protein RAD23 OS=Bombus vosnesenskii OX=207650 GN=LOC117235163 PE=3 SV=1	A0A6J3KLV9	40 kDa	3.2272	3.0826	3.3194	3.18	1.6763	4.7123	3.6777	1.8798
venom acid phosphatase Acph-1-like isoform X1 OS=Bombus vosnesenskii OX=207650 GN=LOC117242856 PE=4 SV=1	A0A6J3LJQ5 (+1)	45 kDa	4.034	3.0826	4.1493	4.2399	3.3526	3.1415	4.9036	0
Protein-L-isoaspartate O-methyltransferase OS=Bombus vosnesenskii OX=207650 GN=LOC117238060 PE=3 SV=1	A0A6J3KYR 4 (+2)	25 kDa	4.8408	4.1101	5.809	4.2399	3.3526	1.5708	1.2259	0
Reticulon-like protein OS=Bombus vosnesenskii OX=207650 GN=LOC117234123 PE=4 SV=1	A0A6J3KG6 0 (+1)	51 kDa	5.6477	5.1377	3.3194	2.12	0	7.8538	3.6777	0
Cystathionine beta-synthase OS=Bombus vosnesenskii OX=207650 GN=LOC117231499 PE=3 SV=1	A0A6J3K0B0 (+1)	56 kDa	4.8408	4.1101	4.1493	6.3599	4.1907	0	1.2259	0
glutathione transferase OS=Bombus vosnesenskii OX=207650 GN=LOC117236856 PE=3 SV=1	A0A6J3KVD 7	17 kDa	4.8408	6.1652	4.9791	1.06	0	7.8538	1.2259	0
defensin-1 OS=Bombus vosnesenskii OX=207650 GN=LOC117233325 PE=4 SV=1	A0A6J3K9M 3	18 kDa	2.4204	0	1.6597	0	9.2196	3.1415	0	3.7596
UDP-glucose:glycoprotein glucosyltransferase isoform X4 OS=Bombus vosnesenskii OX=207650 GN=LOC117234455 PE=3 SV=1	A0A6J3KEF 9 (+3)	174 kDa	7.2613	2.0551	7.4687	0	0	6.283	0	0
Ferritin OS=Bombus vosnesenskii OX=207650 GN=LOC117242977 PE=3 SV=1	A0A6J3LPQ 7	26 kDa	4.034	4.1101	4.1493	3.18	2.5144	3.1415	2.4518	1.8798
sulfide:quinone oxidoreductase, mitochondrial OS=Bombus vosnesenskii OX=207650	A0A6J3LLV0	51 kDa	5.6477	4.1101	2.4896	5.2999	2.5144	1.5708	2.4518	3.7596

GN=LOC117243089 PE=4 SV=1										
ATP synthase subunit OS=Bombus vosnesenskii OX=207650 GN=LOC117233395 PE=3 SV=1	A0A6J3KAL2	11 kDa	3.2272	4.1101	3.3194	1.06	0.83814	3.1415	1.2259	1.8798
mitochondrial-processing peptidase subunit alpha OS=Bombus vosnesenskii OX=207650 GN=LOC117235550 PE=3 SV=1	A0A6J3KNY4	62 kDa	6.4545	3.0826	5.809	3.18	0.83814	1.5708	2.4518	0
Mannosyl-oligosaccharide glucosidase OS=Bombus vosnesenskii OX=207650 GN=LOC117238616 PE=3 SV=1	A0A6J3L672 (+1)	95 kDa	4.034	4.1101	4.9791	4.2399	2.5144	1.5708	0	0
Succinate dehydrogenase [ubiquinone] flavoprotein subunit, mitochondrial OS=Bombus vosnesenskii OX=207650 GN=LOC117237512 PE=3 SV=1	A0A6J3KWV8	72 kDa	4.8408	3.0826	1.6597	1.06	7.5433	0	2.4518	0
alpha-tocopherol transfer protein-like isoform X3 OS=Bombus vosnesenskii OX=207650 GN=LOC117236367 PE=4 SV=1	A0A6J3KPD1 (+2)	36 kDa	4.034	7.1927	4.1493	1.06	0.83814	4.7123	0	0
Cell division control protein 42 homolog OS=Bombus vosnesenskii OX=207650 GN=LOC117230940 PE=3 SV=1	A0A6J3JVC5	21 kDa	4.034	3.0826	1.6597	2.12	5.0288	3.1415	2.4518	1.8798
uncharacterized protein LOC117234474 isoform X1 OS=Bombus vosnesenskii OX=207650 GN=LOC117234474 PE=4 SV=1	A0A6J3KEL2 (+5)	214 kDa	2.4204	3.0826	1.6597	4.2399	5.0288	1.5708	1.2259	0
Cytochrome b-c1 complex subunit Rieske, mitochondrial OS=Bombus vosnesenskii OX=207650 GN=LOC117234293 PE=3 SV=1	A0A6J3KDL9 (+1)	30 kDa	4.8408	6.1652	4.1493	2.12	0	1.5708	2.4518	1.8798
dnaJ protein homolog 1-like OS=Bombus vosnesenskii	A0A6J3L778	39 kDa	1.6136	5.1377	1.6597	6.3599	0.83814	1.5708	4.9036	5.6394

OX=207650 GN=LOC117238783 PE=4 SV=1										
cytoglobin-2-like OS=Bombus vosnesenskii OX=207650 GN=LOC117235202 PE=3 SV=1	A0A6J3KIA7	19 kDa	6.4545	4.1101	4.1493	2.12	1.6763	0	2.4518	1.8798
uncharacterized protein LOC117238289 OS=Bombus vosnesenskii OX=207650 GN=LOC117238289 PE=4 SV=1	A0A6J3L041	48 kDa	3.2272	5.1377	4.9791	5.2999	0.83814	1.5708	1.2259	0
basement membrane-specific heparan sulfate proteoglycan core protein-like isoform X1 OS=Bombus vosnesenskii OX=207650 GN=LOC117242530 PE=4 SV=1	A0A6J3LIG2 (+1)	576 kDa	3.2272	2.0551	4.9791	2.12	3.3526	3.1415	0	0
NPC intracellular cholesterol transporter 2-like OS=Bombus vosnesenskii OX=207650 GN=LOC117237184 PE=3 SV=1	A0A6J3KYB 3	16 kDa	2.4204	2.0551	4.1493	0	3.3526	0	11.033	3.7596
uncharacterized protein LOC117234454 OS=Bombus vosnesenskii OX=207650 GN=LOC117234454 PE=4 SV=1	A0A6J3KEF 4	257 kDa	5.6477	4.1101	5.809	3.18	0	3.1415	1.2259	0
proteasome subunit beta type-1 OS=Bombus vosnesenskii OX=207650 GN=LOC117235259 PE=4 SV=1	A0A6J3KL85	26 kDa	5.6477	5.1377	3.3194	0	0.83814	4.7123	0	0
short/branched chain specific acyl-CoA dehydrogenase, mitochondrial OS=Bombus vosnesenskii OX=207650 GN=LOC117230754 PE=3 SV=1	A0A6J3JVZ2	45 kDa	4.8408	6.1652	2.4896	0	0.83814	3.1415	0	0
GTP cyclohydrolase 1 OS=Bombus vosnesenskii OX=207650 GN=LOC117234789 PE=3 SV=1	A0A6J3KIW6	79 kDa	5.6477	6.1652	3.3194	1.06	0	7.8538	0	0
Proteasome subunit alpha type OS=Bombus vosnesenskii OX=207650 GN=LOC117230960 PE=3 SV=1	A0A6J3JXJ9	28 kDa	4.8408	5.1377	2.4896	0	0	6.283	0	0

Proteasome subunit alpha type OS=Bombus vosnesenskii OX=207650 GN=LOC117232596 PE=3 SV=1	A0A6J3K4C3	29 kDa	6.4545	6.1652	4.1493	0	0	3.1415	0	0
UTP--glucose-1-phosphate uridylyltransferase OS=Bombus vosnesenskii OX=207650 GN=LOC117236522 PE=3 SV=1	A0A6J3KQN1 (+2)	58 kDa	8.0681	5.1377	4.9791	0	0	4.7123	0	0
coproporphyrinogen oxidase OS=Bombus vosnesenskii OX=207650 GN=LOC117240248 PE=3 SV=1	A0A6J3LA96	44 kDa	2.4204	2.0551	3.3194	3.18	3.3526	0	2.4518	1.8798
uncharacterized protein LOC117230524 OS=Bombus vosnesenskii OX=207650 GN=LOC117230524 PE=4 SV=1	A0A6J3JUH5	79 kDa	4.8408	2.0551	3.3194	4.2399	4.1907	0	3.6777	1.8798
spectrin alpha chain isoform X2 OS=Bombus vosnesenskii OX=207650 GN=LOC117230437 PE=3 SV=1	A0A6J3JSQ8 (+1)	278 kDa	1.6136	2.0551	2.4896	1.06	4.1907	1.5708	0	0
Histone H4 OS=Bombus vosnesenskii OX=207650 GN=LOC117236733 PE=3 SV=1	A0A6J3KVC8	11 kDa	6.4545	2.0551	2.4896	0	0	14.137	1.2259	1.8798
acetyl-CoA acetyltransferase, mitochondrial isoform X1 OS=Bombus vosnesenskii OX=207650 GN=LOC117237979 PE=3 SV=1	A0A6J3KYZ6 (+1)	55 kDa	7.2613	1.0275	2.4896	0	3.3526	0	6.1295	0
Proteasome subunit alpha type OS=Bombus vosnesenskii OX=207650 GN=LOC117235814 PE=3 SV=1	A0A6J3KPM1	28 kDa	6.4545	5.1377	4.9791	1.06	0	3.1415	0	0
BMP-binding endothelial regulator protein-like OS=Bombus vosnesenskii OX=207650 GN=LOC117239152 PE=4 SV=1	A0A6J3L556	35 kDa	1.6136	1.0275	0	0	11.734	0	0	0
Fatty acyl-CoA reductase OS=Bombus vosnesenskii OX=207650	A0A6J3LFH2	57 kDa	0	10.275	0	0	0	0	0	0

GN=LOC117240704 PE=3 SV=1										
NSFL1 cofactor p47 OS=Bombus vosnesenskii OX=207650 GN=LOC117236632 PE=4 SV=1	A0A6J3KRE3	43 kDa	2.4204	3.0826	3.3194	2.12	3.3526	3.1415	1.2259	1.8798
zinc finger RNA-binding protein isoform X1 OS=Bombus vosnesenskii OX=207650 GN=LOC117241931 PE=4 SV=1	A0A6J3LFX9 (+1)	109 kDa	3.2272	3.0826	3.3194	4.2399	1.6763	3.1415	3.6777	0
uncharacterized protein LOC117235992 isoform X1 OS=Bombus vosnesenskii OX=207650 GN=LOC117235992 PE=3 SV=1	A0A6J3KMN2	96 kDa	2.4204	3.0826	1.6597	3.18	6.7051	1.5708	3.6777	0
serine hydrolase-like protein OS=Bombus vosnesenskii OX=207650 GN=LOC117233019 PE=4 SV=1	A0A6J3K6R3	34 kDa	4.8408	4.1101	1.6597	4.2399	3.3526	0	1.2259	1.8798
trypsin Blo t 3-like OS=Bombus vosnesenskii OX=207650 GN=LOC117230480 PE=4 SV=1	A0A6J3JUX1	28 kDa	0.80681	6.1652	4.1493	1.06	4.1907	0	3.6777	0
glycine--tRNA ligase OS=Bombus vosnesenskii OX=207650 GN=LOC117240882 PE=3 SV=1	A0A6J3LC76	24 kDa	4.8408	2.0551	4.1493	5.2999	0	1.5708	4.9036	0
Tubulin alpha chain OS=Bombus vosnesenskii OX=207650 GN=LOC117241679 PE=3 SV=1	A0A6J3LI83	50 kDa	5.6477	4.1101	4.1493	0	0	7.8538	0	0
T-complex protein 1 subunit beta OS=Bombus vosnesenskii OX=207650 GN=LOC117233093 PE=3 SV=1	A0A6J3KA42	58 kDa	4.034	2.0551	3.3194	2.12	2.5144	3.1415	2.4518	1.8798
Glycine cleavage system H protein OS=Bombus vosnesenskii OX=207650 GN=LOC117233162 PE=3 SV=1	A0A6J3K7L0	19 kDa	3.2272	2.0551	2.4896	3.18	4.1907	3.1415	1.2259	1.8798
Eukaryotic translation initiation factor 2A OS=Bombus vosnesenskii OX=207650	A0A6J3KU02	65 kDa	5.6477	3.0826	3.3194	2.12	2.5144	1.5708	3.6777	0

GN=LOC117237210 PE=3 SV=1										
Phosphotriesterase-related protein OS=Bombus vosnesenskii OX=207650 GN=LOC117234443 PE=3 SV=1	A0A6J3KEL5	40 kDa	4.034	5.1377	4.9791	2.12	0.83814	0	2.4518	0
glucose dehydrogenase [FAD, quinone]-like OS=Bombus vosnesenskii OX=207650 GN=LOC117235983 PE=3 SV=1	A0A6J3KMI4	71 kDa	3.2272	2.0551	3.3194	5.2999	1.6763	3.1415	0	0
Heat shock protein 83 OS=Bombus vosnesenskii OX=207650 GN=LOC117236428 PE=3 SV=1	A0A6J3KPM9	91 kDa	5.6477	5.1377	4.1493	0	0.83814	4.7123	0	0
prenylcysteine oxidase-like isoform X1 OS=Bombus vosnesenskii OX=207650 GN=LOC117232243 PE=3 SV=1	A0A6J3K2R0 (+1)	56 kDa	4.8408	7.1927	5.809	0	0	1.5708	0	0
dihydropteridine reductase OS=Bombus vosnesenskii OX=207650 GN=LOC117240234 PE=4 SV=1	A0A6J3LBR0	26 kDa	4.8408	2.0551	2.4896	4.2399	0.83814	1.5708	3.6777	1.8798
N-acetyl-D-glucosamine kinase OS=Bombus vosnesenskii OX=207650 GN=LOC117240262 PE=3 SV=1	A0A6J3LAB4 (+1)	40 kDa	6.4545	4.1101	2.4896	1.06	1.6763	1.5708	1.2259	1.8798
inorganic diphosphatase OS=Bombus vosnesenskii OX=207650 GN=LOC117240383 PE=3 SV=1	A0A6J3LAT6	38 kDa	6.4545	1.0275	0.82985	1.06	4.1907	1.5708	2.4518	0
FGGY carbohydrate kinase domain-containing protein OS=Bombus vosnesenskii OX=207650 GN=LOC117232543 PE=3 SV=1	A0A6J3K432	60 kDa	6.4545	3.0826	2.4896	1.06	1.6763	0	4.9036	0
ectonucleoside triphosphate diphosphohydrolase 5 isoform X3 OS=Bombus vosnesenskii OX=207650 GN=LOC117240647 PE=3 SV=1	A0A6J3LB33 (+2)	52 kDa	4.034	4.1101	1.6597	4.2399	2.5144	1.5708	0	0

Adenosine kinase OS=Bombus vosnesenskii OX=207650 GN=LOC117241673 PE=3 SV=1	A0A6J3LE00 (+2)	38 kDa	3.2272	1.0275	2.4896	4.2399	3.3526	0	2.4518	0
Na(+)/H(+) exchange regulatory cofactor NHE-RF1 OS=Bombus vosnesenskii OX=207650 GN=LOC117237691 PE=4 SV=1	A0A6J3L077	28 kDa	0.80681	1.0275	0.82985	2.12	5.867	4.7123	0	0
troponin I 2-like isoform X1 OS=Bombus vosnesenskii OX=207650 GN=LOC117236763 PE=4 SV=1	A0A6J3KR96 (+7)	46 kDa	0	0	0	0	8.3814	0	0	0
nucleolysin TIAR OS=Bombus vosnesenskii OX=207650 GN=LOC117238782 PE=4 SV=1	A0A6J3L2J6	43 kDa	0.80681	3.0826	1.6597	3.18	2.5144	3.1415	3.6777	3.7596
nitrilase and fragile histidine triad fusion protein NitFhit isoform X2 OS=Bombus vosnesenskii OX=207650 GN=LOC117238515 PE=4 SV=1	A0A6J3L5P5 (+1)	32 kDa	4.034	2.0551	2.4896	3.18	1.6763	3.1415	3.6777	1.8798
Glucose-6-phosphate 1-dehydrogenase OS=Bombus vosnesenskii OX=207650 GN=LOC117234841 PE=3 SV=1	A0A6J3KJ54 (+2)	60 kDa	4.034	3.0826	1.6597	3.18	3.3526	1.5708	1.2259	0
Acylamino-acid-releasing enzyme OS=Bombus vosnesenskii OX=207650 GN=LOC117242890 PE=3 SV=1	A0A6J3LKW2 (+1)	86 kDa	4.8408	3.0826	3.3194	5.2999	0.83814	1.5708	1.2259	0
uncharacterized protein LOC117239134 isoform X4 OS=Bombus vosnesenskii OX=207650 GN=LOC117239134 PE=4 SV=1	A0A6J3L8S6	146 kDa	3.2272	4.1101	2.4896	3.18	0.83814	3.1415	3.6777	0
3'(2'),5'-bisphosphate nucleotidase 1 OS=Bombus vosnesenskii OX=207650 GN=LOC117232457 PE=3 SV=1	A0A6J3K5B4	33 kDa	4.8408	2.0551	2.4896	4.2399	2.5144	0	4.9036	0
Ferritin OS=Bombus vosnesenskii OX=207650 GN=LOC117236772 PE=3 SV=1	A0A6J3KS72	22 kDa	0.80681	3.0826	2.4896	3.18	0.83814	6.283	0	0

6-phosphogluconolactonase OS=Bombus vosnesenskii OX=207650 GN=LOC117233760 PE=3 SV=1	A0A6J3KDY5	26 kDa	1.6136	1.0275	0.82985	1.06	7.5433	0	2.4518	0
voltage-dependent anion-selective channel-like OS=Bombus vosnesenskii OX=207650 GN=LOC117241771 PE=3 SV=1	A0A6J3LGF4	31 kDa	3.2272	4.1101	4.1493	1.06	0	3.1415	0	0
glucose dehydrogenase [FAD, quinone]-like OS=Bombus vosnesenskii OX=207650 GN=LOC117231081 PE=3 SV=1	A0A6J3JY56	67 kDa	1.6136	2.0551	0	2.12	6.7051	1.5708	0	0
alpha-glucosidase OS=Bombus vosnesenskii OX=207650 GN=LOC117233421 PE=4 SV=1	A0A6J3KC70	66 kDa	8.8749	1.0275	0.82985	0	3.3526	0	4.9036	5.6394
Proteasome subunit alpha type OS=Bombus vosnesenskii OX=207650 GN=LOC117232568 PE=3 SV=1	A0A6J3K460	27 kDa	4.034	5.1377	3.3194	0	0	3.1415	0	0
Proteasome subunit alpha type OS=Bombus vosnesenskii OX=207650 GN=LOC117239914 PE=3 SV=1	A0A6J3L8U3	31 kDa	4.034	4.1101	4.1493	0	0	1.5708	0	0
Proteasome subunit beta OS=Bombus vosnesenskii OX=207650 GN=LOC117232970 PE=3 SV=1	A0A6J3K7Q6	30 kDa	3.2272	5.1377	2.4896	1.06	0	0	0	0
acetyl-CoA carboxylase OS=Bombus vosnesenskii OX=207650 GN=LOC117241816 PE=4 SV=1	A0A6J3LE37 (+8)	273 kDa	3.2272	8.2202	2.4896	0	0.83814	0	0	0
alpha,alpha-trehalose-phosphate synthase [UDP-forming] isoform X1 OS=Bombus vosnesenskii OX=207650 GN=LOC117234349 PE=3 SV=1	A0A6J3KDU9 (+2)	92 kDa	6.4545	4.1101	4.1493	0	0	0	0	0
ADP-ribosylation factor GTPase-activating protein 2 isoform X2 OS=Bombus	A0A6J3KAA4	59 kDa	4.034	2.0551	2.4896	3.18	0.83814	1.5708	3.6777	1.8798

vosnesenskii OX=207650 GN=LOC117233115 PE=4 SV=1										
glutaredoxin-C4-like OS=Bombus vosnesenskii OX=207650 GN=LOC117241705 PE=4 SV=1	A0A6J3LG02	11 kDa	1.6136	2.0551	2.4896	0	5.867	0	1.2259	1.8798
cytochrome b5-like OS=Bombus vosnesenskii OX=207650 GN=LOC117230358 PE=3 SV=1	A0A6J3JU52	14 kDa	3.2272	2.0551	3.3194	2.12	3.3526	0	2.4518	0
3-hydroxyacyl-CoA dehydrogenase type-2 OS=Bombus vosnesenskii OX=207650 GN=LOC117236101 PE=3 SV=1	A0A6J3KN53	27 kDa	6.4545	6.1652	4.1493	1.06	0	1.5708	0	0
neurochondrin homolog OS=Bombus vosnesenskii OX=207650 GN=LOC117231035 PE=3 SV=1	A0A6J3JW26	84 kDa	5.6477	6.1652	4.1493	0	0	3.1415	0	0
myosin heavy chain, muscle isoform X2 OS=Bombus vosnesenskii OX=207650 GN=LOC117237014 PE=3 SV=1	A0A6J3KST3 (+34)	225 kDa	0	1.0275	3.3194	0	3.3526	1.5708	0	0
Nuclear migration protein nudC OS=Bombus vosnesenskii OX=207650 GN=LOC117232109 PE=3 SV=1	A0A6J3K3R6	39 kDa	3.2272	3.0826	0.82985	3.18	2.5144	4.7123	1.2259	1.8798
programmed cell death protein 6 isoform X1 OS=Bombus vosnesenskii OX=207650 GN=LOC117236771 PE=4 SV=1	A0A6J3KRC1 (+1)	22 kDa	4.034	1.0275	3.3194	3.18	1.6763	3.1415	0	1.8798
uncharacterized protein LOC117239118 isoform X1 OS=Bombus vosnesenskii OX=207650 GN=LOC117239118 PE=4 SV=1	A0A6J3L3X2	40 kDa	1.6136	1.0275	0.82985	2.12	5.0288	3.1415	0	5.6394
xanthine dehydrogenase OS=Bombus vosnesenskii OX=207650 GN=LOC117243463 PE=3 SV=1	A0A6J3LRY3	150 kDa	4.034	3.0826	2.4896	1.06	1.6763	1.5708	1.2259	0

MD-2-related lipid-recognition protein-like OS=Bombus vosnesenskii OX=207650 GN=LOC117233559 PE=3 SV=1	A0A6J3K9R7	18 kDa	2.4204	3.0826	3.3194	3.18	3.3526	0	1.2259	0
RNA-binding protein squid isoform X5 OS=Bombus vosnesenskii OX=207650 GN=LOC117242918 PE=4 SV=1	A0A6J3LK38 (+7)	34 kDa	0.80681	4.1101	3.3194	3.18	1.6763	1.5708	0	0
peptidylprolyl isomerase OS=Bombus vosnesenskii OX=207650 GN=LOC117240928 PE=4 SV=1	A0A6J3LG48	40 kDa	2.4204	3.0826	2.4896	4.2399	0.83814	0	1.2259	0
Adenylosuccinate lyase OS=Bombus vosnesenskii OX=207650 GN=LOC117243150 PE=3 SV=1	A0A6J3LQJ7 (+1)	55 kDa	1.6136	2.0551	1.6597	2.12	5.0288	0	0	0
papilin isoform X1 OS=Bombus vosnesenskii OX=207650 GN=LOC117236618 PE=4 SV=1	A0A6J3KQN2 (+9)	320 kDa	2.4204	3.0826	1.6597	0	1.6763	0	1.2259	0
Glycogen debranching enzyme OS=Bombus vosnesenskii OX=207650 GN=LOC117232264 PE=3 SV=1	A0A6J3K5E9	183 kDa	4.034	3.0826	1.6597	1.06	4.1907	0	0	0
V-type proton ATPase subunit S1-like OS=Bombus vosnesenskii OX=207650 GN=LOC117240195 PE=3 SV=1	A0A6J3L8V9	45 kDa	4.8408	5.1377	4.1493	0	0	6.283	0	0
AP complex subunit beta OS=Bombus vosnesenskii OX=207650 GN=LOC117237848 PE=3 SV=1	A0A6J3KY96 (+1)	103 kDa	4.034	2.0551	2.4896	2.12	0.83814	3.1415	2.4518	1.8798
ras-like GTP-binding protein Rho1 isoform X1 OS=Bombus vosnesenskii OX=207650 GN=LOC117239208 PE=4 SV=1	A0A6J3L5I5 (+2)	25 kDa	1.6136	2.0551	1.6597	1.06	4.1907	3.1415	1.2259	1.8798
Regucalcin OS=Bombus vosnesenskii OX=207650 GN=LOC117236559 PE=3 SV=1	A0A6J3KR45	34 kDa	2.4204	3.0826	1.6597	2.12	2.5144	4.7123	3.6777	0
sp|ALBU_HUMAN|	sp|ALBU_HUMAN|	69 kDa	2.4204	4.1101	3.3194	2.12	1.6763	0	2.4518	1.8798

uncharacterized protein LOC117230646 OS=Bombus vosnesenskii OX=207650 GN=LOC117230646 PE=4 SV=1	A0A6J3JUZ3	26 kDa	1.6136	3.0826	4.9791	3.18	1.6763	0	2.4518	0
transmembrane protein 256 homolog OS=Bombus vosnesenskii OX=207650 GN=LOC117236681 PE=3 SV=1	A0A6J3KUA9	18 kDa	3.2272	3.0826	2.4896	3.18	1.6763	3.1415	0	0
transport and Golgi organization 2 homolog OS=Bombus vosnesenskii OX=207650 GN=LOC117242023 PE=4 SV=1	A0A6J3LK30	31 kDa	3.2272	2.0551	3.3194	4.2399	0.83814	0	2.4518	0
Carboxylic ester hydrolase OS=Bombus vosnesenskii OX=207650 GN=LOC117241653 PE=3 SV=1	A0A6J3LD05	65 kDa	2.4204	1.0275	2.4896	3.18	2.5144	0	1.2259	0
Inositol oxygenase OS=Bombus vosnesenskii OX=207650 GN=LOC117237723 PE=3 SV=1	A0A6J3KY58	35 kDa	2.4204	1.0275	0.82985	1.06	5.867	0	2.4518	0
T-cell immunomodulatory protein OS=Bombus vosnesenskii OX=207650 GN=LOC117236404 PE=3 SV=1	A0A6J3KQ87	66 kDa	3.2272	5.1377	4.1493	2.12	0	3.1415	0	0
phosphoglucomutase (alpha-D-glucose-1,6-bisphosphate-dependent) OS=Bombus vosnesenskii OX=207650 GN=LOC117230652 PE=3 SV=1	A0A6J3JVE8	62 kDa	4.034	4.1101	2.4896	1.06	1.6763	0	0	0
threonine--tRNA ligase OS=Bombus vosnesenskii OX=207650 GN=LOC117234964 PE=3 SV=1	A0A6J3KKT2 (+1)	83 kDa	2.4204	1.0275	0.82985	1.06	3.3526	0	0	0
fatty-acid amide hydrolase 2 isoform X2 OS=Bombus vosnesenskii OX=207650 GN=LOC117239684 PE=4 SV=1	A0A6J3L7U3 (+1)	60 kDa	2.4204	3.0826	1.6597	3.18	0	0	1.2259	0
putative tricarboxylate transport protein, mitochondrial OS=Bombus vosnesenskii OX=207650	A0A6J3KYL7	36 kDa	6.4545	4.1101	2.4896	0	0	1.5708	0	0

GN=LOC117237901 PE=3 SV=1										
V-type proton ATPase subunit E OS=Bombus vosnesenskii OX=207650 GN=LOC117232661 PE=3 SV=1	A0A6J3K7C4	26 kDa	0.80681	2.0551	3.3194	0	3.3526	0	0	0
glutamic acid-rich protein-like OS=Bombus vosnesenskii OX=207650 GN=LOC117238541 PE=4 SV=1	A0A6J3L2E4	87 kDa	1.6136	2.0551	2.4896	3.18	2.5144	1.5708	2.4518	1.8798
deaminated glutathione amidase-like isoform X1 OS=Bombus vosnesenskii OX=207650 GN=LOC117238137 PE=4 SV=1	A0A6J3KZ94	64 kDa	3.2272	1.0275	0.82985	3.18	1.6763	1.5708	1.2259	1.8798
glucose dehydrogenase [FAD, quinone]-like isoform X2 OS=Bombus vosnesenskii OX=207650 GN=LOC117231080 PE=3 SV=1	A0A6J3JY52 (+1)	71 kDa	0.80681	3.0826	4.1493	3.18	0.83814	1.5708	1.2259	3.7596
Glucosylceramidase OS=Bombus vosnesenskii OX=207650 GN=LOC117233136 PE=3 SV=1	A0A6J3K7F7 (+1)	58 kDa	3.2272	1.0275	3.3194	0	1.6763	1.5708	2.4518	1.8798
heterogeneous nuclear ribonucleoprotein Q isoform X6 OS=Bombus vosnesenskii OX=207650 GN=LOC117232048 PE=4 SV=1	A0A6J3K1W9 (+5)	74 kDa	3.2272	1.0275	2.4896	1.06	0	4.7123	2.4518	0
Eukaryotic translation initiation factor 2 subunit 1 OS=Bombus vosnesenskii OX=207650 GN=LOC117239492 PE=3 SV=1	A0A6J3L8Q9	38 kDa	3.2272	1.0275	1.6597	1.06	2.5144	3.1415	0	0
Regucalcin OS=Bombus vosnesenskii OX=207650 GN=LOC117236558 PE=3 SV=1	A0A6J3KQD8	37 kDa	1.6136	1.0275	4.1493	0	1.6763	3.1415	4.9036	0
Isocitrate dehydrogenase [NAD] subunit, mitochondrial OS=Bombus vosnesenskii OX=207650 GN=LOC117233292 PE=3 SV=1	A0A6J3KBC2	39 kDa	4.034	3.0826	4.1493	0	0.83814	0	0	0

thioredoxin, mitochondrial-like OS=Bombus vosnesenskii OX=207650 GN=LOC117243081 PE=4 SV=1	A0A6J3LLT4	15 kDa	1.6136	2.0551	0.82985	1.06	2.5144	1.5708	2.4518	3.7596
Transgelin OS=Bombus vosnesenskii OX=207650 GN=LOC117240275 PE=3 SV=1	A0A6J3L983	20 kDa	3.2272	3.0826	3.3194	2.12	0.83814	1.5708	2.4518	1.8798
NPC intracellular cholesterol transporter 2 homolog a-like OS=Bombus vosnesenskii OX=207650 GN=LOC117243477 PE=3 SV=1	A0A6J3LNA 2	17 kDa	2.4204	3.0826	2.4896	2.12	1.6763	1.5708	1.2259	1.8798
Tetraspanin OS=Bombus vosnesenskii OX=207650 GN=LOC117236900 PE=3 SV=1	A0A6J3KS54	26 kDa	1.6136	3.0826	0.82985	2.12	1.6763	1.5708	1.2259	3.7596
histidine triad nucleotide-binding protein 1 OS=Bombus vosnesenskii OX=207650 GN=LOC117231718 PE=4 SV=1	A0A6J3K193	18 kDa	2.4204	0	1.6597	2.12	2.5144	3.1415	1.2259	0
xaa-Pro aminopeptidase ApepP isoform X2 OS=Bombus vosnesenskii OX=207650 GN=LOC117230786 PE=3 SV=1	A0A6J3JVW 5 (+1)	70 kDa	1.6136	0	3.3194	2.12	0.83814	3.1415	6.1295	0
uncharacterized protein LOC117236114 OS=Bombus vosnesenskii OX=207650 GN=LOC117236114 PE=4 SV=1	A0A6J3KNR 7	44 kDa	2.4204	1.0275	3.3194	0	2.5144	3.1415	1.2259	0
lachesin isoform X1 OS=Bombus vosnesenskii OX=207650 GN=LOC117241761 PE=4 SV=1	A0A6J3LDU 5 (+1)	41 kDa	2.4204	4.1101	2.4896	1.06	1.6763	3.1415	0	0
collagen alpha-1(XVIII) chain isoform X4 OS=Bombus vosnesenskii OX=207650 GN=LOC117231243 PE=4 SV=1	A0A6J3JX02 (+9)	123 kDa	3.2272	2.0551	4.9791	3.18	0	1.5708	1.2259	0
talin-1 isoform X3 OS=Bombus vosnesenskii OX=207650 GN=LOC117233591 PE=4 SV=1	A0A6J3KA08 (+4)	264 kDa	0.80681	3.0826	5.809	5.2999	0	0	1.2259	0
S-(hydroxymethyl)glutathione dehydrogenase OS=Bombus	A0A6J3LEG 5	41 kDa	5.6477	3.0826	3.3194	1.06	0	3.1415	0	0

vosnesenskii OX=207650 GN=LOC117240382 PE=3 SV=1										
Endoribonuclease OS=Bombus vosnesenskii OX=207650 GN=LOC117230272 PE=3 SV=1	A0A6J3JRJ6 (+1)	68 kDa	0.80681	0	2.4896	1.06	7.5433	0	0	1.8798
ras-like protein 1 OS=Bombus vosnesenskii OX=207650 GN=LOC117237705 PE=4 SV=1	A0A6J3KXH 7	22 kDa	1.6136	3.0826	1.6597	3.18	0	0	0	0
calsyntenin-1 OS=Bombus vosnesenskii OX=207650 GN=LOC117243057 PE=4 SV=1	A0A6J3LN26	106 kDa	4.8408	5.1377	4.1493	1.06	0	0	0	0
C-factor OS=Bombus vosnesenskii OX=207650 GN=LOC117234027 PE=3 SV=1	A0A6J3KEG 2	27 kDa	1.6136	2.0551	2.4896	3.18	0.83814	0	1.2259	0
Triokinase/FMN cyclase OS=Bombus vosnesenskii OX=207650 GN=LOC117239461 PE=4 SV=1	A0A6J3L5L3	58 kDa	4.034	1.0275	1.6597	1.06	0.83814	3.1415	0	1.8798
Translationally-controlled tumor protein homolog OS=Bombus vosnesenskii OX=207650 GN=LOC117234030 PE=3 SV=1	A0A6J3KEG 8	20 kDa	3.2272	2.0551	2.4896	2.12	1.6763	1.5708	0	0
guanine nucleotide-binding protein G(Q) subunit alpha isoform X5 OS=Bombus vosnesenskii OX=207650 GN=LOC117242948 PE=3 SV=1	A0A6J3LMH 4	47 kDa	3.2272	0	3.3194	1.06	0	1.5708	1.2259	1.8798
enoyl-CoA hydratase domain-containing protein 3, mitochondrial isoform X1 OS=Bombus vosnesenskii OX=207650 GN=LOC117237515 PE=4 SV=1	A0A6J3KW W4 (+1)	33 kDa	3.2272	2.0551	2.4896	4.2399	0.83814	0	1.2259	0
peroxiredoxin-6-like OS=Bombus vosnesenskii OX=207650 GN=LOC117231869 PE=3 SV=1	A0A6J3K2Y0	25 kDa	0.80681	0	1.6597	3.18	6.7051	0	1.2259	1.8798
endoplasmic reticulum resident protein 29 OS=Bombus vosnesenskii OX=207650	A0A6J3KSR 8	27 kDa	3.2272	3.0826	0.82985	3.18	0	3.1415	1.2259	0

GN=LOC117236860 PE=4 SV=1										
Sugar phosphate phosphatase OS=Bombus vosnesenskii OX=207650 GN=LOC117240022 PE=3 SV=1	A0A6J3LAT7	53 kDa	1.6136	1.0275	2.4896	2.12	3.3526	0	1.2259	0
dynein heavy chain, cytoplasmic isoform X2 OS=Bombus vosnesenskii OX=207650 GN=LOC117237873 PE=3 SV=1	A0A6J3L167 (+4)	534 kDa	1.6136	2.0551	0.82985	0	0	1.5708	2.4518	13.159
UDP-glucuronosyltransferase 2B15-like isoform X1 OS=Bombus vosnesenskii OX=207650 GN=LOC117233445 PE=4 SV=1	A0A6J3KAD 6	60 kDa	4.8408	2.0551	2.4896	2.12	0	0	2.4518	0
alpha-actinin, sarcomeric isoform X3 OS=Bombus vosnesenskii OX=207650 GN=LOC117237385 PE=3 SV=1	A0A6J3KVR 3 (+2)	107 kDa	0.80681	3.0826	4.9791	0	0	1.5708	0	0
Proteasome subunit alpha type OS=Bombus vosnesenskii OX=207650 GN=LOC117234178 PE=3 SV=1	A0A6J3KDX 6	28 kDa	1.6136	2.0551	4.1493	0	0	3.1415	0	0
Ras-related protein Rab-14 OS=Bombus vosnesenskii OX=207650 GN=LOC117242778 PE=3 SV=1	A0A6J3LJA3	24 kDa	2.4204	5.1377	1.6597	2.12	0	0	0	0
paramyosin, long form-like OS=Bombus vosnesenskii OX=207650 GN=LOC117233449 PE=4 SV=1	A0A6J3KAE 0	102 kDa	0	0	13.278	0	0	1.5708	0	0
Pyruvate dehydrogenase E1 component subunit beta OS=Bombus vosnesenskii OX=207650 GN=LOC117237770 PE=4 SV=1	A0A6J3KX72	38 kDa	0	0	0	0	5.867	0	0	0
glutathione S-transferase-like OS=Bombus vosnesenskii OX=207650 GN=LOC117231921 PE=4 SV=1	A0A6J3K137	24 kDa	1.6136	2.0551	2.4896	1.06	1.6763	3.1415	1.2259	3.7596

sphingomyelin phosphodiesterase isoform X1 OS=Bombus vosnesenskii OX=207650 GN=LOC117233533 PE=3 SV=1	A0A6J3KCR4 (+2)	83 kDa	3.2272	3.0826	3.3194	1.06	0.83814	0	1.2259	1.8798
NAD(P)H-hydrate epimerase OS=Bombus vosnesenskii OX=207650 GN=LOC117231992 PE=3 SV=1	A0A6J3K1J2	26 kDa	1.6136	1.0275	3.3194	2.12	1.6763	1.5708	1.2259	0
14 kDa phosphohistidine phosphatase-like OS=Bombus vosnesenskii OX=207650 GN=LOC117238069 PE=3 SV=1	A0A6J3L033	18 kDa	1.6136	2.0551	1.6597	3.18	0.83814	3.1415	2.4518	0
T-complex protein 1 subunit epsilon OS=Bombus vosnesenskii OX=207650 GN=LOC117231767 PE=3 SV=1	A0A6J3K1L3	60 kDa	3.2272	2.0551	2.4896	2.12	1.6763	1.5708	1.2259	0
group XV phospholipase A2-like OS=Bombus vosnesenskii OX=207650 GN=LOC117236116 PE=4 SV=1	A0A6J3KNS9	46 kDa	3.2272	2.0551	1.6597	4.2399	0.83814	1.5708	0	0
cytochrome P450 6a2-like OS=Bombus vosnesenskii OX=207650 GN=LOC117233509 PE=3 SV=1	A0A6J3K9I2	58 kDa	1.6136	3.0826	3.3194	2.12	0.83814	3.1415	0	0
Beta-galactosidase OS=Bombus vosnesenskii OX=207650 GN=LOC117242768 PE=3 SV=1	A0A6J3LLJ1	73 kDa	2.4204	1.0275	2.4896	1.06	1.6763	0	4.9036	0
single-stranded DNA-binding protein, mitochondrial OS=Bombus vosnesenskii OX=207650 GN=LOC117239048 PE=4 SV=1	A0A6J3L4L1	16 kDa	1.6136	2.0551	0.82985	2.12	2.5144	1.5708	0	0
trithorax group protein osa-like OS=Bombus vosnesenskii OX=207650 GN=LOC117242406 PE=4 SV=1	A0A6J3LLX3	79 kDa	2.4204	2.0551	0.82985	1.06	4.1907	0	1.2259	0
Histone H2B OS=Bombus vosnesenskii OX=207650	A0A6J3KB65 (+1)	14 kDa	2.4204	3.0826	2.4896	2.12	0	1.5708	0	0

GN=LOC117233847 PE=3 SV=1										
uncharacterized protein LOC117239060 OS=Bombus vosnesenskii OX=207650 GN=LOC117239060 PE=4 SV=1	A0A6J3L3K4	97 kDa	4.8408	4.1101	0.82985	0	0	3.1415	0	0
vacuolar protein sorting-associated protein 35-like isoform X3 OS=Bombus vosnesenskii OX=207650 GN=LOC117231970 PE=3 SV=1	A0A6J3K1F3 (+2)	134 kDa	6.4545	1.0275	1.6597	0	0	0	0	0
leucine-rich repeat-containing protein 15-like OS=Bombus vosnesenskii OX=207650 GN=LOC117235123 PE=4 SV=1	A0A6J3KHZ2	71 kDa	2.4204	0	2.4896	1.06	1.6763	1.5708	2.4518	1.8798
Ribosome maturation protein SBDS OS=Bombus vosnesenskii OX=207650 GN=LOC117238320 PE=3 SV=1	A0A6J3L4N6	29 kDa	2.4204	1.0275	3.3194	2.12	0.83814	0	2.4518	3.7596
muscle LIM protein Mlp84B-like isoform X1 OS=Bombus vosnesenskii OX=207650 GN=LOC117242615 PE=4 SV=1	A0A6J3LKV1 (+1)	54 kDa	0.80681	1.0275	1.6597	2.12	2.5144	1.5708	0	1.8798
Dynein light chain OS=Bombus vosnesenskii OX=207650 GN=LOC117242439 PE=3 SV=1	A0A6J3LIA8	10 kDa	0.80681	3.0826	1.6597	1.06	1.6763	0	1.2259	1.8798
inactive selenide, water dikinase-like protein OS=Bombus vosnesenskii OX=207650 GN=LOC117242254 PE=4 SV=1	A0A6J3LHE5	45 kDa	2.4204	1.0275	1.6597	4.2399	0.83814	3.1415	1.2259	0
uncharacterized protein LOC117238560 OS=Bombus vosnesenskii OX=207650 GN=LOC117238560 PE=4 SV=1	A0A6J3L1G2	72 kDa	2.4204	2.0551	3.3194	1.06	0.83814	1.5708	1.2259	0
protein singed OS=Bombus vosnesenskii OX=207650 GN=LOC117239083 PE=3 SV=1	A0A6J3L4Q7	58 kDa	1.6136	1.0275	1.6597	4.2399	1.6763	1.5708	1.2259	0
SUMO-conjugating enzyme UBC9-B OS=Bombus vosnesenskii OX=207650	A0A6J3KZ08	18 kDa	2.4204	2.0551	2.4896	2.12	2.5144	1.5708	0	0

GN=LOC117237318 PE=3 SV=1										
S-methyl-5'-thioadenosine phosphorylase OS=Bombus vosnesenskii OX=207650 GN=LOC117237214 PE=3 SV=1	A0A6J3KU08	31 kDa	0.80681	2.0551	1.6597	3.18	3.3526	0	1.2259	0
unc-112-related protein-like isoform X1 OS=Bombus vosnesenskii OX=207650 GN=LOC117231070 PE=3 SV=1	A0A6J3JY15	81 kDa	3.2272	2.0551	2.4896	2.12	0.83814	1.5708	0	0
maleylacetoacetate isomerase OS=Bombus vosnesenskii OX=207650 GN=LOC117231068 PE=3 SV=1	A0A6J3JW20 (+1)	25 kDa	2.4204	2.0551	2.4896	3.18	0	1.5708	1.2259	0
V-type proton ATPase subunit D OS=Bombus vosnesenskii OX=207650 GN=LOC117234096 PE=3 SV=1	A0A6J3KDI5	28 kDa	1.6136	2.0551	3.3194	1.06	0	1.5708	1.2259	0
nucleolin-like OS=Bombus vosnesenskii OX=207650 GN=LOC117238201 PE=4 SV=1	A0A6J3L0D3	25 kDa	2.4204	1.0275	1.6597	1.06	3.3526	1.5708	0	0
Acylphosphatase OS=Bombus vosnesenskii OX=207650 GN=LOC117230289 PE=3 SV=1	A0A6J3JTL5	15 kDa	1.6136	0	1.6597	2.12	3.3526	1.5708	0	0
10 kDa heat shock protein, mitochondrial OS=Bombus vosnesenskii OX=207650 GN=LOC117242850 PE=3 SV=1	A0A6J3LKJ7	11 kDa	1.6136	1.0275	2.4896	0	2.5144	0	0	1.8798
Carboxylic ester hydrolase OS=Bombus vosnesenskii OX=207650 GN=LOC117231399 PE=3 SV=1	A0A6J3JXY5 (+1)	63 kDa	5.6477	0	0.82985	1.06	2.5144	0	2.4518	0
acetyl-coenzyme A synthetase OS=Bombus vosnesenskii OX=207650 GN=LOC117242149 PE=3 SV=1	A0A6J3LKQ3	77 kDa	3.2272	1.0275	0	4.2399	2.5144	0	1.2259	0
Proteasome subunit beta OS=Bombus vosnesenskii OX=207650 GN=LOC117232080 PE=3 SV=1	A0A6J3K3M2	31 kDa	1.6136	2.0551	3.3194	0	0	3.1415	0	0

Proteasome subunit beta OS=Bombus vosnesenskii OX=207650 GN=LOC117231304 PE=3 SV=1	A0A6J3JXF8	24 kDa	2.4204	3.0826	4.1493	0	0	1.5708	0	0
Fatty acyl-CoA reductase OS=Bombus vosnesenskii OX=207650 GN=LOC117241889 PE=3 SV=1	A0A6J3LH74	58 kDa	4.8408	4.1101	2.4896	0	0	3.1415	0	0
cytosolic non-specific dipeptidase OS=Bombus vosnesenskii OX=207650 GN=LOC117242103 PE=3 SV=1	A0A6J3LFN9	53 kDa	2.4204	0	0.82985	1.06	4.1907	0	0	0
ATP synthase subunit O, mitochondrial OS=Bombus vosnesenskii OX=207650 GN=LOC117236983 PE=3 SV=1	A0A6J3KSM 5	27 kDa	1.6136	4.1101	2.4896	0	0	0	0	0
Heat shock protein 83 OS=Bombus vosnesenskii OX=207650 GN=LOC117240806 PE=3 SV=1	A0A6J3LC29	83 kDa	5.6477	2.0551	0	0	0.83814	0	0	0
UPF0160 protein MYG1, mitochondrial isoform X2 OS=Bombus vosnesenskii OX=207650 GN=LOC117237639 PE=3 SV=1	A0A6J3KXK 7 (+1)	38 kDa	3.2272	1.0275	1.6597	1.06	0.83814	1.5708	2.4518	1.8798
vesicular integral-membrane protein VIP36 OS=Bombus vosnesenskii OX=207650 GN=LOC117230739 PE=4 SV=1	A0A6J3JVV6	37 kDa	0.80681	2.0551	0.82985	2.12	1.6763	4.7123	1.2259	1.8798
Glutathione synthetase OS=Bombus vosnesenskii OX=207650 GN=LOC117235755 PE=3 SV=1	A0A6J3KLC 7	56 kDa	1.6136	1.0275	0.82985	1.06	0.83814	3.1415	2.4518	1.8798
palmitoyl-protein hydrolase OS=Bombus vosnesenskii OX=207650 GN=LOC117232656 PE=3 SV=1	A0A6J3K7B9	26 kDa	2.4204	2.0551	3.3194	1.06	0.83814	1.5708	1.2259	0
Signal transducer and activator of transcription OS=Bombus vosnesenskii OX=207650	A0A6J3JV29 (+1)	88 kDa	1.6136	2.0551	0	2.12	0	3.1415	4.9036	1.8798

GN=LOC117230840 PE=3 SV=1										
Eukaryotic translation initiation factor 3 subunit B OS=Bombus vosnesenskii OX=207650 GN=LOC117242857 PE=3 SV=1	A0A6J3LJR1	81 kDa	2.4204	1.0275	3.3194	1.06	0.83814	0	1.2259	0
balbiani ring protein 3-like isoform X11 OS=Bombus vosnesenskii OX=207650 GN=LOC117242717 PE=3 SV=1	A0A6J3LJ12 (+9)	116 kDa	0.80681	1.0275	3.3194	2.12	1.6763	0	1.2259	0
UDP-glucose 4-epimerase OS=Bombus vosnesenskii OX=207650 GN=LOC117238034 PE=3 SV=1	A0A6J3KZC 2 (+1)	42 kDa	3.2272	2.0551	2.4896	1.06	0	3.1415	0	0
Dihydrolipoamide acetyltransferase component of pyruvate dehydrogenase complex OS=Bombus vosnesenskii OX=207650 GN=LOC117242247 PE=3 SV=1	A0A6J3LGH 4	50 kDa	1.6136	2.0551	2.4896	3.18	0.83814	0	0	0
CDGSH iron-sulfur domain-containing protein 2 homologue OS=Bombus vosnesenskii OX=207650 GN=LOC117230581 PE=3 SV=1	A0A6J3JV32	15 kDa	2.4204	2.0551	1.6597	1.06	0	3.1415	0	0
phospholipase A1 OS=Bombus vosnesenskii OX=207650 GN=LOC117234586 PE=3 SV=1	A0A6J3KG2 8	34 kDa	1.6136	1.0275	0.82985	0	4.1907	0	1.2259	0
TRPL translocation defect protein 14 isoform X6 OS=Bombus vosnesenskii OX=207650 GN=LOC117235482 PE=4 SV=1	A0A6J3KKF 9	55 kDa	0.80681	0	0.82985	1.06	3.3526	0	1.2259	0
kynurenine/alpha-aminoadipate aminotransferase, mitochondrial-like OS=Bombus vosnesenskii OX=207650 GN=LOC117232479 PE=4 SV=1	A0A6J3K3P8	48 kDa	3.2272	2.0551	0.82985	0	1.6763	0	1.2259	0
Beta-glucuronidase OS=Bombus vosnesenskii OX=207650	A0A6J3KEI2	77 kDa	0.80681	3.0826	4.9791	0	0	1.5708	0	0

GN=LOC117234039 PE=3 SV=1										
Coatomer subunit alpha OS=Bombus vosnesenskii OX=207650 GN=LOC117234785 PE=4 SV=1	A0A6J3KIV8 (+1)	138 kDa	1.6136	3.0826	1.6597	0	0.83814	0	0	0
Isocitrate dehydrogenase [NAD] subunit, mitochondrial OS=Bombus vosnesenskii OX=207650 GN=LOC117240153 PE=3 SV=1	A0A6J3LBB8 (+1)	39 kDa	4.034	3.0826	1.6597	0	0	1.5708	0	0
heterogeneous nuclear ribonucleoprotein Q isoform X8 OS=Bombus vosnesenskii OX=207650 GN=LOC117232048 PE=4 SV=1	A0A6J3K4C 0 (+2)	73 kDa	2.4204	0	2.4896	0	1.6763	3.1415	0	0
N-acetylglucosamine-6-sulfatase-like OS=Bombus vosnesenskii OX=207650 GN=LOC117234266 PE=4 SV=1	A0A6J3KGZ 3	56 kDa	1.6136	1.0275	3.3194	2.12	0.83814	1.5708	1.2259	1.8798
Bis(5'-nucleosyl)-tetraphosphatase [asymmetrical] OS=Bombus vosnesenskii OX=207650 GN=LOC117236196 PE=3 SV=1	A0A6J3KRT 9	16 kDa	1.6136	1.0275	0.82985	1.06	1.6763	1.5708	1.2259	1.8798
glutaryl-CoA dehydrogenase, mitochondrial OS=Bombus vosnesenskii OX=207650 GN=LOC117242259 PE=3 SV=1	A0A6J3LL89	47 kDa	3.2272	1.0275	0.82985	2.12	0.83814	0	1.2259	0
interleukin enhancer-binding factor 2 isoform X2 OS=Bombus vosnesenskii OX=207650 GN=LOC117238670 PE=4 SV=1	A0A6J3L1Z9 (+1)	42 kDa	1.6136	1.0275	3.3194	1.06	0.83814	0	1.2259	0
matrix metalloproteinase-14 isoform X1 OS=Bombus vosnesenskii OX=207650 GN=LOC117233695 PE=3 SV=1	A0A6J3KAJ7 (+3)	67 kDa	2.4204	1.0275	1.6597	0	0.83814	4.7123	3.6777	0
adipocyte plasma membrane-associated protein-like OS=Bombus vosnesenskii OX=207650	A0A6J3KV00	63 kDa	2.4204	2.0551	1.6597	2.12	0.83814	1.5708	0	0

GN=LOC117236667 PE=3 SV=1										
scoloptoxin SSD14-like isoform X3 OS=Bombus vosnesenskii OX=207650 GN=LOC117239721 PE=4 SV=1	A0A6J3L709 (+3)	73 kDa	1.6136	2.0551	2.4896	1.06	1.6763	3.1415	0	0
Signal peptidase complex subunit 2 OS=Bombus vosnesenskii OX=207650 GN=LOC117232148 PE=3 SV=1	A0A6J3K3X4	21 kDa	3.2272	3.0826	1.6597	1.06	0	0	1.2259	0
protein sidekick isoform X4 OS=Bombus vosnesenskii OX=207650 GN=LOC117239561 PE=4 SV=1	A0A6J3LB24 (+5)	246 kDa	2.4204	2.0551	3.3194	1.06	0	3.1415	0	0
protein CutA homolog OS=Bombus vosnesenskii OX=207650 GN=LOC117243128 PE=3 SV=1	A0A6J3LM45	16 kDa	1.6136	2.0551	1.6597	2.12	0	0	1.2259	0
beta-N-acetylhexosaminidase OS=Bombus vosnesenskii OX=207650 GN=LOC117233990 PE=3 SV=1	A0A6J3KFB2 (+3)	78 kDa	2.4204	2.0551	0.82985	2.12	1.6763	0	0	0
proteasome endopeptidase complex OS=Bombus vosnesenskii OX=207650 GN=LOC117241848 PE=4 SV=1	A0A6J3LFE8	25 kDa	3.2272	3.0826	3.3194	0	0	1.5708	0	0
cAMP-dependent protein kinase type I regulatory subunit isoform X1 OS=Bombus vosnesenskii OX=207650 GN=LOC117238261 PE=3 SV=1	A0A6J3KZZ1 (+2)	42 kDa	0.80681	0	0	1.06	5.867	0	1.2259	0
40S ribosomal protein S2 OS=Bombus vosnesenskii OX=207650 GN=LOC117236486 PE=3 SV=1	A0A6J3KQG7 (+2)	31 kDa	2.4204	3.0826	2.4896	0	0	0	0	0
obscurin isoform X4 OS=Bombus vosnesenskii OX=207650 GN=LOC117239206 PE=3 SV=1	A0A6J3L4G8 (+7)	633 kDa	0.80681	0	8.2985	1.06	1.6763	0	0	0
Spectrin beta chain OS=Bombus vosnesenskii	A0A6J3KCF4 (+6)	269 kDa	0.80681	1.0275	0.82985	1.06	2.5144	1.5708	1.2259	1.8798

OX=207650 GN=LOC117234064 PE=3 SV=1										
Inosine triphosphate pyrophosphatase OS=Bombus vosnesenskii OX=207650 GN=LOC117231570 PE=3 SV=1	A0A6J3JYW2	22 kDa	0.80681	3.0826	2.4896	1.06	0.83814	1.5708	1.2259	0
molybdenum cofactor biosynthesis protein 1 isoform X1 OS=Bombus vosnesenskii OX=207650 GN=LOC117236876 PE=3 SV=1	A0A6J3KSU9 (+2)	62 kDa	2.4204	1.0275	1.6597	2.12	0.83814	1.5708	0	1.8798
alpha-N-acetylglucosaminidase OS=Bombus vosnesenskii OX=207650 GN=LOC117232735 PE=4 SV=1	A0A6J3K6D3	89 kDa	1.6136	2.0551	0.82985	1.06	1.6763	0	2.4518	0
tr|A0A6J3LHV5|A0A6J3LHV5_9HYME-DECOY	?	0.38	2.0551	2.4896	1.06	0	1.2259		0	
CAAX prenyl protease OS=Bombus vosnesenskii OX=207650 GN=LOC117232659 PE=3 SV=1	A0A6J3K6G2	52 kDa	1.6136	3.0826	1.6597	2.12	0	1.5708	0	0
40S ribosomal protein S3a OS=Bombus vosnesenskii OX=207650 GN=LOC117241712 PE=3 SV=1	A0A6J3LG17	30 kDa	2.4204	2.0551	1.6597	1.06	0	3.1415	0	0
ejaculatory bulb-specific protein 3 OS=Bombus vosnesenskii OX=207650 GN=LOC117242113 PE=4 SV=1	A0A6J3LGA9	14 kDa	0.80681	0	0.82985	0	2.5144	1.5708	0	0
melanotransferrin OS=Bombus vosnesenskii OX=207650 GN=LOC117235303 PE=4 SV=1	A0A6J3KJE7	93 kDa	2.4204	2.0551	2.4896	0	0	1.5708	0	0
Trifunctional purine biosynthetic protein adenosine-3 OS=Bombus vosnesenskii OX=207650 GN=LOC117235347 PE=3 SV=1	A0A6J3KMS9	109 kDa	2.4204	0	0.82985	0	2.5144	0	2.4518	0
mitogen-activated protein kinase OS=Bombus vosnesenskii OX=207650	A0A6J3KK39	42 kDa	1.6136	0	3.3194	4.2399	0.83814	0	0	0

GN=LOC117235423 PE=4 SV=1										
NADH dehydrogenase [ubiquinone] 1 alpha subcomplex subunit 9, mitochondrial OS=Bombus vosnesenskii OX=207650 GN=LOC117242515 PE=4 SV=1	A0A6J3LIC5	46 kDa	1.6136	2.0551	2.4896	0	0	1.5708	0	0
protein THEM6 OS=Bombus vosnesenskii OX=207650 GN=LOC117236807 PE=4 SV=1	A0A6J3KV25	23 kDa	3.2272	3.0826	2.4896	0	0	1.5708	0	0
Phosphate carrier protein, mitochondrial OS=Bombus vosnesenskii OX=207650 GN=LOC117230445 PE=3 SV=1	A0A6J3JUC3 (+1)	39 kDa	4.034	2.0551	1.6597	0	0	1.5708	0	0
vitamin K-dependent protein C-like OS=Bombus vosnesenskii OX=207650 GN=LOC117231419 PE=4 SV=1	A0A6J3JY39	35 kDa	0	0	0.82985	0	2.5144	1.5708	0	1.8798
AP-2 complex subunit alpha OS=Bombus vosnesenskii OX=207650 GN=LOC117233212 PE=3 SV=1	A0A6J3K929	105 kDa	2.4204	0	1.6597	3.18	0	0	0	0
calpain-A-like isoform X1 OS=Bombus vosnesenskii OX=207650 GN=LOC117237860 PE=3 SV=1	A0A6J3KXN2 (+7)	96 kDa	1.6136	0	2.4896	0	0	0	0	0
gamma-glutamylcyclotransferase OS=Bombus vosnesenskii OX=207650 GN=LOC117243262 PE=4 SV=1	A0A6J3LLX0	20 kDa	2.4204	1.0275	0.82985	1.06	0.83814	1.5708	1.2259	0
thioredoxin domain-containing protein OS=Bombus vosnesenskii OX=207650 GN=LOC117235277 PE=4 SV=1	A0A6J3KIN8	34 kDa	2.4204	1.0275	0.82985	2.12	0.83814	1.5708	1.2259	0
uncharacterized peptidase C1-like protein F26E4.3 isoform X1 OS=Bombus vosnesenskii OX=207650 GN=LOC117232051 PE=3 SV=1	A0A6J3K1X4 (+1)	53 kDa	1.6136	2.0551	0.82985	1.06	0.83814	1.5708	1.2259	0

Probable methylthioribulose-1-phosphate dehydratase OS=Bombus vosnesenskii OX=207650 GN=LOC117236782 PE=3 SV=1	A0A6J3KS65	27 kDa	1.6136	1.0275	1.6597	3.18	0.83814	1.5708	1.2259	0
copper transport protein ATOX1 homolog OS=Bombus vosnesenskii OX=207650 GN=LOC117236882 PE=4 SV=1	A0A6J3KWA0	8 kDa	2.4204	1.0275	1.6597	2.12	1.6763	0	1.2259	0
GTP:AMP phosphotransferase, mitochondrial OS=Bombus vosnesenskii OX=207650 GN=LOC117242256 PE=3 SV=1	A0A6J3LH10	25 kDa	1.6136	1.0275	0.82985	2.12	1.6763	0	1.2259	0
glutathione hydrolase 1 proenzyme-like OS=Bombus vosnesenskii OX=207650 GN=LOC117230952 PE=4 SV=1	A0A6J3JWY5	63 kDa	1.6136	1.0275	0.82985	3.18	0	1.5708	1.2259	0
fructose-bisphosphatase OS=Bombus vosnesenskii OX=207650 GN=LOC117239089 PE=3 SV=1	A0A6J3L4S9	37 kDa	2.4204	1.0275	1.6597	1.06	1.6763	0	1.2259	0
proliferation-associated protein 2G4 OS=Bombus vosnesenskii OX=207650 GN=LOC117235774 PE=3 SV=1	A0A6J3KQ42	44 kDa	0.80681	1.0275	1.6597	1.06	3.3526	0	0	1.8798
Cytidine deaminase OS=Bombus vosnesenskii OX=207650 GN=LOC117231413 PE=3 SV=1	A0A6J3JY11	16 kDa	0.80681	2.0551	2.4896	2.12	0.83814	0	1.2259	0
oligoribonuclease-like isoform X1 OS=Bombus vosnesenskii OX=207650 GN=LOC117236508 PE=3 SV=1	A0A6J3KU81 (+1)	26 kDa	1.6136	3.0826	2.4896	2.12	0.83814	0	0	0
m7GpppX diphosphatase OS=Bombus vosnesenskii OX=207650 GN=LOC117236526 PE=3 SV=1	A0A6J3KQN7	39 kDa	1.6136	2.0551	2.4896	2.12	0	0	0	0
insulin-degrading enzyme isoform X1 OS=Bombus vosnesenskii OX=207650	A0A6J3L959 (+1)	116 kDa	4.034	2.0551	1.6597	0	0	3.1415	0	0

GN=LOC117240265 PE=3 SV=1										
troponin T isoform X1 OS=Bombus vosnesenskii OX=207650 GN=LOC117233281 PE=3 SV=1	A0A6J3K890 (+12)	52 kDa	0	1.0275	0.82985	0	4.1907	3.1415	0	0
pentatricopeptide repeat-containing protein 2, mitochondrial-like OS=Bombus vosnesenskii OX=207650 GN=LOC117243230 PE=4 SV=1	A0A6J3LLX9	46 kDa	4.8408	2.0551	0.82985	0	0	1.5708	0	0
uncharacterized protein LOC117232149 OS=Bombus vosnesenskii OX=207650 GN=LOC117232149 PE=4 SV=1	A0A6J3K406	46 kDa	0.80681	3.0826	2.4896	4.2399	0	0	1.2259	0
prohibitin-2 OS=Bombus vosnesenskii OX=207650 GN=LOC117243366 PE=3 SV=1	A0A6J3LRJ1	39 kDa	4.034	2.0551	2.4896	0	0	0	0	0
prickle planar cell polarity protein 3-A isoform X5 OS=Bombus vosnesenskii OX=207650 GN=LOC117232250 PE=4 SV=1	A0A6J3K2S0 (+8)	66 kDa	0.80681	2.0551	2.4896	0	0	0	0	0
carboxypeptidase B-like OS=Bombus vosnesenskii OX=207650 GN=LOC117243232 PE=3 SV=1	A0A6J3LMI8	48 kDa	0	3.0826	2.4896	0	0	0	3.6777	0
Proteasome subunit beta OS=Bombus vosnesenskii OX=207650 GN=LOC117243188 PE=3 SV=1	A0A6J3LNP 1	23 kDa	1.6136	2.0551	3.3194	0	0	0	0	0
fibrillin-2-like OS=Bombus vosnesenskii OX=207650 GN=LOC117232996 PE=4 SV=1	A0A6J3K6M 1	314 kDa	0	8.2202	1.6597	1.06	0	0	0	0
D-3-phosphoglycerate dehydrogenase isoform X2 OS=Bombus vosnesenskii OX=207650 GN=LOC117240657 PE=3 SV=1	A0A6J3LD11 (+1)	52 kDa	2.4204	0	1.6597	0	0	0	0	0
Thioredoxin domain-containing protein 17 OS=Bombus	A0A6J3KM5 6	15 kDa	1.6136	1.0275	1.6597	1.06	0.83814	1.5708	1.2259	1.8798

vosnesenskii OX=207650 GN=LOC117235921 PE=4 SV=1										
L-galactose dehydrogenase-like isoform X1 OS=Bombus vosnesenskii OX=207650 GN=LOC117231806 PE=4 SV=1	A0A6J3K1T8 (+2)	42 kDa	1.6136	1.0275	0.82985	1.06	1.6763	1.5708	1.2259	0
uncharacterized oxidoreductase YuxG-like OS=Bombus vosnesenskii OX=207650 GN=LOC117233668 PE=4 SV=1	A0A6J3KDH3	57 kDa	0.80681	1.0275	2.4896	1.06	1.6763	1.5708	1.2259	0
cytosolic purine 5'-nucleotidase isoform X4 OS=Bombus vosnesenskii OX=207650 GN=LOC117238620 PE=3 SV=1	A0A6J3L1Q1 (+7)	73 kDa	0.80681	1.0275	0.82985	2.12	1.6763	1.5708	0	0
cadherin-99C isoform X2 OS=Bombus vosnesenskii OX=207650 GN=LOC117230454 PE=4 SV=1	A0A6J3JUP2 (+1)	151 kDa	2.4204	2.0551	1.6597	1.06	0.83814	1.5708	0	0
protein-synthesizing GTPase OS=Bombus vosnesenskii OX=207650 GN=LOC117238512 PE=4 SV=1	A0A6J3L274	51 kDa	0.80681	1.0275	1.6597	2.12	1.6763	1.5708	0	0
Eukaryotic peptide chain release factor subunit 1 OS=Bombus vosnesenskii OX=207650 GN=LOC117242157 PE=3 SV=1	A0A6J3LII2 (+1)	49 kDa	0.80681	1.0275	1.6597	1.06	0.83814	1.5708	0	0
uncharacterized protein LOC117236272 OS=Bombus vosnesenskii OX=207650 GN=LOC117236272 PE=4 SV=1	A0A6J3KS91	34 kDa	1.6136	2.0551	1.6597	1.06	0	0	2.4518	0
acyl-CoA-binding protein homolog OS=Bombus vosnesenskii OX=207650 GN=LOC117236283 PE=4 SV=1	A0A6J3KSA8	10 kDa	1.6136	1.0275	0.82985	2.12	0.83814	0	0	0
cytochrome P450 9e2-like OS=Bombus vosnesenskii OX=207650 GN=LOC117236974 PE=3 SV=1	A0A6J3KSL5	57 kDa	2.4204	2.0551	0.82985	1.06	0	3.1415	0	0

fibrillin-2-like isoform X1 OS=Bombus vosnesenskii OX=207650 GN=LOC117240054 PE=4 SV=1	A0A6J3L981	177 kDa	1.6136	0	1.6597	0	1.6763	0	1.2259	0
laminin subunit alpha OS=Bombus vosnesenskii OX=207650 GN=LOC117242057 PE=4 SV=1	A0A6J3LHZ6	406 kDa	1.6136	1.0275	3.3194	2.12	0	0	0	0
NADH dehydrogenase [ubiquinone] 1 beta subcomplex subunit 6 OS=Bombus vosnesenskii OX=207650 GN=LOC117236517 PE=3 SV=1	A0A6J3KQ6 0	19 kDa	1.6136	1.0275	2.4896	0	0	3.1415	0	0
lysozyme OS=Bombus vosnesenskii OX=207650 GN=LOC117235558 PE=4 SV=1	A0A6J3KP02	17 kDa	0.80681	0	0.82985	0	3.3526	1.5708	0	0
mannose-1-phosphate guanylyltransferase OS=Bombus vosnesenskii OX=207650 GN=LOC117231277 PE=3 SV=1	A0A6J3JZ83	41 kDa	1.6136	1.0275	1.6597	0	0	0	1.2259	0
Glycine cleavage system P protein OS=Bombus vosnesenskii OX=207650 GN=LOC117236441 PE=3 SV=1	A0A6J3KPQ 4 (+1)	108 kDa	2.4204	1.0275	0.82985	1.06	0	0	0	0
echinoderm microtubule-associated protein-like 2 isoform X2 OS=Bombus vosnesenskii OX=207650 GN=LOC117233530 PE=3 SV=1	A0A6J3K9K3 (+10)	100 kDa	1.6136	2.0551	1.6597	0	0	0	0	0
ras-related protein Rab-2 OS=Bombus vosnesenskii OX=207650 GN=LOC117238882 PE=4 SV=1	A0A6J3L405	24 kDa	2.4204	2.0551	0	0	1.6763	0	0	0
protein transport protein Sec24C OS=Bombus vosnesenskii OX=207650 GN=LOC117232139 PE=3 SV=1	A0A6J3K292	140 kDa	2.4204	0	0.82985	0	1.6763	0	0	0
Dihydropyrimidine dehydrogenase [NADP(+)] OS=Bombus vosnesenskii	A0A6J3KEM 5	112 kDa	0	0	1.6597	1.06	1.6763	0	0	0

OX=207650 GN=LOC117234475 PE=3 SV=1										
Eukaryotic translation initiation factor 5A OS=Bombus vosnesenskii OX=207650 GN=LOC117232474 PE=3 SV=1	A0A6J3K6A2	18 kDa	0.80681	1.0275	0	0	5.0288	0	0	0
1-phosphatidylinositol 4,5-bisphosphate phosphodiesterase OS=Bombus vosnesenskii OX=207650 GN=LOC117230326 PE=4 SV=1	A0A6J3JTG5 (+1)	130 kDa	0.80681	1.0275	0.82985	1.06	0.83814	3.1415	1.2259	0
malectin-A OS=Bombus vosnesenskii OX=207650 GN=LOC117234132 PE=3 SV=1	A0A6J3KDP2	30 kDa	2.4204	1.0275	0.82985	1.06	0.83814	3.1415	0	0
Moesin/ezrin/radixin homolog 1 OS=Bombus vosnesenskii OX=207650 GN=LOC117231632 PE=4 SV=1	A0A6J3JZ97 (+4)	109 kDa	1.6136	2.0551	1.6597	1.06	0.83814	0	1.2259	0
Leucine carboxyl methyltransferase 1 OS=Bombus vosnesenskii OX=207650 GN=LOC117238210 PE=3 SV=1	A0A6J3L0N7	36 kDa	0.80681	2.0551	0.82985	2.12	1.6763	0	1.2259	1.8798
C-terminal-binding protein isoform X2 OS=Bombus vosnesenskii OX=207650 GN=LOC117234995 PE=3 SV=1	A0A6J3KH43	50 kDa	0.80681	1.0275	0.82985	3.18	1.6763	0	1.2259	0
ribose-5-phosphate isomerase OS=Bombus vosnesenskii OX=207650 GN=LOC117240140 PE=3 SV=1	A0A6J3L9B1	30 kDa	0.80681	1.0275	1.6597	1.06	1.6763	1.5708	0	0
Very-long-chain (3R)-3-hydroxyacyl-CoA dehydratase OS=Bombus vosnesenskii OX=207650 GN=LOC117230285 PE=3 SV=1	A0A6J3JTA3	26 kDa	0.80681	2.0551	1.6597	2.12	0	3.1415	0	0
calmodulin OS=Bombus vosnesenskii OX=207650 GN=LOC117241777 PE=4 SV=1	A0A6J3LEZ5	17 kDa	0.80681	1.0275	1.6597	1.06	3.3526	0	0	1.8798

furin-like protease 2 isoform X3 OS=Bombus vosnesenskii OX=207650 GN=LOC117232940 PE=3 SV=1	A0A6J3K6D9 (+2)	141 kDa	2.4204	1.0275	0.82985	2.12	0.83814	0	0	0
Coatomer subunit delta OS=Bombus vosnesenskii OX=207650 GN=LOC117231827 PE=3 SV=1	A0A6J3K207	58 kDa	1.6136	2.0551	1.6597	1.06	0.83814	0	0	0
40S ribosomal protein SA OS=Bombus vosnesenskii OX=207650 GN=LOC117243134 PE=3 SV=1	A0A6J3LNG4	34 kDa	1.6136	2.0551	1.6597	0	0	1.5708	0	0
Sodium/potassium-transporting ATPase subunit alpha OS=Bombus vosnesenskii OX=207650 GN=LOC117237222 PE=3 SV=1	A0A6J3KU22 (+7)	116 kDa	0	2.0551	2.4896	0	0.83814	1.5708	0	0
60S ribosomal protein L3 OS=Bombus vosnesenskii OX=207650 GN=LOC117243278 PE=3 SV=1	A0A6J3LP64	47 kDa	0.80681	2.0551	3.3194	0	0	1.5708	0	0
(S)-3-amino-2-methylpropionate transaminase OS=Bombus vosnesenskii OX=207650 GN=LOC117237002 PE=3 SV=1	A0A6J3KX53 (+2)	56 kDa	1.6136	1.0275	1.6597	0	0	0	2.4518	0
serine proteinase stubble-like isoform X1 OS=Bombus vosnesenskii OX=207650 GN=LOC117230823 PE=4 SV=1	A0A6J3JUZ6 (+1)	105 kDa	1.6136	2.0551	2.4896	0	0	3.1415	0	0
Aminopeptidase OS=Bombus vosnesenskii OX=207650 GN=LOC117241474 PE=3 SV=1	A0A6J3LCH3 (+3)	112 kDa	0.80681	4.1101	1.6597	0	0	0	1.2259	0
Methionine aminopeptidase OS=Bombus vosnesenskii OX=207650 GN=LOC117241610 PE=3 SV=1	A0A6J3LFA7	42 kDa	2.4204	0	3.3194	1.06	0	1.5708	0	0
Calcium-transporting ATPase OS=Bombus vosnesenskii OX=207650 GN=LOC117242425 PE=3 SV=1	A0A6J3LHD7 (+9)	138 kDa	0	0	0	1.06	2.5144	1.5708	0	0

ras-like GTP-binding protein RhoL OS=Bombus vosnesenskii OX=207650 GN=LOC117235702 PE=4 SV=1	A0A6J3KL07	22 kDa	1.6136	0	1.6597	0	0	0	0	0
nucleoplasmin-like protein isoform X1 OS=Bombus vosnesenskii OX=207650 GN=LOC117238295 PE=3 SV=1	A0A6J3L178 (+1)	20 kDa	0	0	1.6597	0	0	1.5708	0	0
Pyruvate dehydrogenase E1 component subunit alpha OS=Bombus vosnesenskii OX=207650 GN=LOC117241718 PE=4 SV=1	A0A6J3LEK4 (+1)	44 kDa	0	0	0	0	3.3526	0	0	0
non-specific serine/threonine protein kinase OS=Bombus vosnesenskii OX=207650 GN=LOC117233470 PE=3 SV=1	A0A6J3KCG8 (+1)	28 kDa	0.80681	1.0275	1.6597	1.06	0.83814	0	1.2259	0
Polypeptide N-acetylgalactosaminyltransferase OS=Bombus vosnesenskii OX=207650 GN=LOC117238410 PE=3 SV=1	A0A6J3L406	68 kDa	1.6136	2.0551	0.82985	1.06	0.83814	1.5708	0	0
ATPase H(+)-transporting accessory protein 2 isoform X3 OS=Bombus vosnesenskii OX=207650 GN=LOC117231873 PE=4 SV=1	A0A6J3K2Y5	38 kDa	0.80681	3.0826	0.82985	1.06	0	1.5708	1.2259	1.8798
acyl-coenzyme A thioesterase 13-like OS=Bombus vosnesenskii OX=207650 GN=LOC117236201 PE=3 SV=1	A0A6J3KRU9	15 kDa	0.80681	1.0275	1.6597	1.06	1.6763	0	0	1.8798
uncharacterized protein LOC117233336 OS=Bombus vosnesenskii OX=207650 GN=LOC117233336 PE=4 SV=1	A0A6J3K8M3	44 kDa	1.6136	2.0551	0.82985	1.06	0	1.5708	1.2259	0
protein-tyrosine-phosphatase OS=Bombus vosnesenskii OX=207650 GN=LOC117232714 PE=3 SV=1	A0A6J3K569 (+1)	233 kDa	2.4204	1.0275	0.82985	1.06	0.83814	0	1.2259	0
Phosphomannomutase OS=Bombus vosnesenskii	A0A6J3JXX1	30 kDa	1.6136	2.0551	0.82985	0	0.83814	1.5708	0	0

OX=207650 GN=LOC117231391 PE=3 SV=1										
twinfilin isoform X3 OS=Bombus vosnesenskii OX=207650 GN=LOC117242612 PE=3 SV=1	A0A6J3LIH0 (+3)	39 kDa	2.4204	1.0275	0.82985	1.06	0.83814	0	0	0
dnaJ homolog subfamily C member 5 isoform X1 OS=Bombus vosnesenskii OX=207650 GN=LOC117239256 PE=4 SV=1	A0A6J3L9K0 (+1)	28 kDa	1.6136	1.0275	1.6597	0	0	1.5708	2.4518	0
Transcription initiation factor IIA subunit 2 OS=Bombus vosnesenskii OX=207650 GN=LOC117231844 PE=3 SV=1	A0A6J3K0J2	13 kDa	1.6136	1.0275	2.4896	1.06	0	0	1.2259	0
circadian clock-controlled protein-like OS=Bombus vosnesenskii OX=207650 GN=LOC117234143 PE=4 SV=1	A0A6J3KDQ 7	27 kDa	1.6136	1.0275	1.6597	2.12	0	0	1.2259	0
V-type proton ATPase subunit a OS=Bombus vosnesenskii OX=207650 GN=LOC117231861 PE=3 SV=1	A0A6J3K0I1 (+3)	97 kDa	0.80681	2.0551	0.82985	0	0	3.1415	0	0
D-2-hydroxyglutarate dehydrogenase, mitochondrial isoform X1 OS=Bombus vosnesenskii OX=207650 GN=LOC117241384 PE=3 SV=1	A0A6J3LC84 (+1)	61 kDa	2.4204	1.0275	0.82985	0	0.83814	0	0	0
disco-interacting protein 2 homolog A isoform X2 OS=Bombus vosnesenskii OX=207650 GN=LOC117236238 PE=4 SV=1	A0A6J3KNR 4 (+9)	238 kDa	0.80681	0	0.82985	0	0.83814	0	1.2259	0
Glycerol-3-phosphate dehydrogenase [NAD(+)] OS=Bombus vosnesenskii OX=207650 GN=LOC117231867 PE=3 SV=1	A0A6J3K0N 7 (+1)	40 kDa	1.6136	0	1.6597	1.06	0	0	2.4518	1.8798
division abnormally delayed protein OS=Bombus vosnesenskii OX=207650	A0A6J3L896	69 kDa	2.4204	1.0275	1.6597	1.06	0	1.5708	0	0

GN=LOC117239738 PE=3 SV=1										
ovalbumin-related protein X-like isoform X2 OS=Bombus vosnesenskii OX=207650 GN=LOC117231310 PE=3 SV=1	A0A6J3JXH0 (+1)	45 kDa	0.80681	1.0275	1.6597	0	1.6763	0	0	0
Acyltransferase OS=Bombus vosnesenskii OX=207650 GN=LOC117240348 PE=3 SV=1	A0A6J3LC54	40 kDa	1.6136	4.1101	0.82985	0	0	1.5708	0	0
cytochrome P450 6k1-like OS=Bombus vosnesenskii OX=207650 GN=LOC117235447 PE=3 SV=1	A0A6J3KJM 7	58 kDa	2.4204	2.0551	1.6597	0	0	0	0	0
Fatty acyl-CoA reductase OS=Bombus vosnesenskii OX=207650 GN=LOC117239039 PE=3 SV=1	A0A6J3L6P5	60 kDa	1.6136	1.0275	2.4896	0	0	0	0	0
Dolichyl-diphosphooligosaccharide--protein glycosyltransferase subunit 1 OS=Bombus vosnesenskii OX=207650 GN=LOC117238409 PE=3 SV=1	A0A6J3L1M 5	70 kDa	1.6136	1.0275	1.6597	0	0	0	0	0
uncharacterized protein LOC117236207 OS=Bombus vosnesenskii OX=207650 GN=LOC117236207 PE=4 SV=1	A0A6J3KP49	260 kDa	1.6136	0	0	2.12	1.6763	0	3.6777	0
aquaporin-like isoform X3 OS=Bombus vosnesenskii OX=207650 GN=LOC117242641 PE=3 SV=1	A0A6J3LIN4 (+2)	28 kDa	1.6136	2.0551	1.6597	0	0	0	0	0
short-chain specific acyl-CoA dehydrogenase, mitochondrial OS=Bombus vosnesenskii OX=207650 GN=LOC117236862 PE=3 SV=1	A0A6J3KST 0	45 kDa	2.4204	0	0	2.12	0.83814	0	0	0
uncharacterized protein LOC117231796 OS=Bombus vosnesenskii OX=207650 GN=LOC117231796 PE=4 SV=1	A0A6J3K096	47 kDa	3.2272	1.0275	0.82985	0	0	0	0	0

prostatic acid phosphatase-like OS=Bombus vosnesenskii OX=207650 GN=LOC117243201 PE=4 SV=1	A0A6J3LLU4	49 kDa	0	0	0	0	3.3526	0	0	0
oxysterol-binding protein 1 isoform X6 OS=Bombus vosnesenskii OX=207650 GN=LOC117232772 PE=4 SV=1	A0A6J3K5C5 (+6)	88 kDa	0	1.0275	0.82985	1.06	0.83814	1.5708	1.2259	0
long-chain fatty acid transport protein 1-like OS=Bombus vosnesenskii OX=207650 GN=LOC117242805 PE=3 SV=1	A0A6J3LK79	73 kDa	0.80681	1.0275	0.82985	0	0	1.5708	0	1.8798
WD repeat and FYVE domain-containing protein 2 OS=Bombus vosnesenskii OX=207650 GN=LOC117230957 PE=4 SV=1	A0A6J3JVH9	46 kDa	0	0	0.82985	2.12	0.83814	1.5708	1.2259	0
S-adenosylmethionine synthase OS=Bombus vosnesenskii OX=207650 GN=LOC117242186 PE=3 SV=1	A0A6J3LH11 (+1)	44 kDa	1.6136	1.0275	1.6597	1.06	0.83814	0	0	0
vigilin OS=Bombus vosnesenskii OX=207650 GN=LOC117239631 PE=4 SV=1	A0A6J3L7L3	139 kDa	0	0	0.82985	1.06	1.6763	0	0	0
pyroglutamyl-peptidase 1 OS=Bombus vosnesenskii OX=207650 GN=LOC117236169 PE=3 SV=1	A0A6J3KNS2	23 kDa	0.80681	1.0275	1.6597	0	1.6763	0	0	0
protein Skeletor, isoforms B/C isoform X2 OS=Bombus vosnesenskii OX=207650 GN=LOC117238159 PE=4 SV=1	A0A6J3L2R5 (+1)	77 kDa	0	2.0551	1.6597	1.06	0	1.5708	0	0
Serine/threonine-protein phosphatase 2A activator OS=Bombus vosnesenskii OX=207650 GN=LOC117235894 PE=3 SV=1	A0A6J3KQW1	38 kDa	2.4204	0	1.6597	1.06	0	0	1.2259	0
Coatomer subunit beta' OS=Bombus vosnesenskii OX=207650	A0A6J3LIM4 (+1)	105 kDa	0.80681	2.0551	0.82985	1.06	0	0	0	0

GN=LOC117242638 PE=3 SV=1										
protein bark beetle OS=Bombus vosnesenskii OX=207650 GN=LOC117235781 PE=4 SV=1	A0A6J3KPG1	331 kDa	2.4204	1.0275	0.82985	0	0.83814	0	0	0
WD repeat-containing protein 61 isoform X1 OS=Bombus vosnesenskii OX=207650 GN=LOC117240893 PE=4 SV=1	A0A6J3LB17 (+1)	36 kDa	1.6136	2.0551	1.6597	0	0	0	1.2259	0
phospholipase D3-like isoform X3 OS=Bombus vosnesenskii OX=207650 GN=LOC117238488 PE=3 SV=1	A0A6J3L5I2 (+2)	63 kDa	0	2.0551	0.82985	1.06	0	1.5708	0	0
Peptidoglycan-recognition protein OS=Bombus vosnesenskii OX=207650 GN=LOC117239036 PE=3 SV=1	A0A6J3L883	21 kDa	2.4204	1.0275	0.82985	1.06	0	0	0	0
uncharacterized protein LOC117232066 OS=Bombus vosnesenskii OX=207650 GN=LOC117232066 PE=4 SV=1	A0A6J3K1Z5	95 kDa	1.6136	1.0275	1.6597	1.06	0	0	0	0
Proliferating cell nuclear antigen OS=Bombus vosnesenskii OX=207650 GN=LOC117230283 PE=3 SV=1	A0A6J3JRQ4	29 kDa	1.6136	1.0275	0.82985	0	0	1.5708	0	0
modular serine protease-like OS=Bombus vosnesenskii OX=207650 GN=LOC117241982 PE=4 SV=1	A0A6J3LJY1	71 kDa	0.80681	0	1.6597	3.18	0	0	1.2259	0
peptidyl-prolyl cis-trans isomerase FKBP62-like OS=Bombus vosnesenskii OX=207650 GN=LOC117232903 PE=4 SV=1	A0A6J3K664	33 kDa	1.6136	0	1.6597	1.06	1.6763	0	0	0
GMP reductase OS=Bombus vosnesenskii OX=207650 GN=LOC117239369 PE=3 SV=1	A0A6J3LA22	38 kDa	0.80681	1.0275	0.82985	0	0	1.5708	0	0
Alkaline phosphatase OS=Bombus vosnesenskii OX=207650	A0A6J3LMQ8	59 kDa	3.2272	1.0275	0	1.06	0	0	1.2259	0

GN=LOC117243409 PE=3 SV=1										
V-type proton ATPase subunit F OS=Bombus vosnesenskii OX=207650 GN=LOC117230942 PE=3 SV=1	A0A6J3JWV3	14 kDa	0.80681	0	0.82985	0	0.83814	0	0	0
angio-associated migratory cell protein isoform X1 OS=Bombus vosnesenskii OX=207650 GN=LOC117237439 PE=4 SV=1	A0A6J3KV86 (+1)	45 kDa	1.6136	2.0551	2.4896	0	0	0	0	0
Glutamine-dependent NAD(+) synthetase OS=Bombus vosnesenskii OX=207650 GN=LOC117238577 PE=3 SV=1	A0A6J3L2K3	84 kDa	1.6136	2.0551	1.6597	0	0	0	0	0
protein unzipped OS=Bombus vosnesenskii OX=207650 GN=LOC117235937 PE=4 SV=1	A0A6J3KR34	53 kDa	2.4204	1.0275	0.82985	0	0	0	0	0
Dystroglycan 1 OS=Bombus vosnesenskii OX=207650 GN=LOC117239203 PE=4 SV=1	A0A6J3L7I4 (+1)	145 kDa	2.4204	2.0551	1.6597	0	0	1.5708	0	0
acyl-CoA Delta(11) desaturase-like isoform X2 OS=Bombus vosnesenskii OX=207650 GN=LOC117240417 PE=3 SV=1	A0A6J3LB05 (+1)	40 kDa	2.4204	0	2.4896	0	0	1.5708	0	0
V-type proton ATPase subunit OS=Bombus vosnesenskii OX=207650 GN=LOC117233393 PE=3 SV=1	A0A6J3KBY0	40 kDa	1.6136	1.0275	1.6597	0	0	0	0	0
Obg-like ATPase 1 OS=Bombus vosnesenskii OX=207650 GN=LOC117237974 PE=3 SV=1	A0A6J3L2I8	45 kDa	0.80681	0	0	1.06	1.6763	0	0	0
GTP-binding nuclear protein OS=Bombus vosnesenskii OX=207650 GN=LOC117236382 PE=3 SV=1	A0A6J3KTJ1	25 kDa	1.6136	0	1.6597	0	0	0	0	0
phospholipase A1 OS=Bombus vosnesenskii OX=207650 GN=LOC117234583 PE=3 SV=1	A0A6J3KFG7	35 kDa	0.80681	4.1101	0	0	0	0	0	0
ADP-ribosylation factor-like protein 8B-A OS=Bombus	A0A6J3L2I9	21 kDa	0.80681	1.0275	0.82985	0	0.83814	1.5708	0	0

vosnesenskii OX=207650 GN=LOC117238565 PE=3 SV=1										
Queuine tRNA-ribosyltransferase accessory subunit 2 OS=Bombus vosnesenskii OX=207650 GN=LOC117235141 PE=3 SV=1	A0A6J3KI32 (+2)	47 kDa	0.80681	0	0.82985	2.12	0.83814	1.5708	1.2259	0
extended synaptotagmin-2 isoform X2 OS=Bombus vosnesenskii OX=207650 GN=LOC117239155 PE=4 SV=1	A0A6J3L460 (+2)	91 kDa	0.80681	1.0275	1.6597	1.06	0	0	1.2259	0
ubiquitin-conjugating enzyme E2 L3 OS=Bombus vosnesenskii OX=207650 GN=LOC117231470 PE=3 SV=1	A0A6J3JY99	18 kDa	0.80681	0	0.82985	1.06	0.83814	0	1.2259	0
Uroporphyrinogen decarboxylase OS=Bombus vosnesenskii OX=207650 GN=LOC117232472 PE=3 SV=1	A0A6J3K5D 4 (+1)	40 kDa	1.6136	1.0275	0.82985	1.06	0.83814	0	0	0
F-BAR domain only protein 2 isoform X7 OS=Bombus vosnesenskii OX=207650 GN=LOC117241230 PE=3 SV=1	A0A6J3LBX5 (+8)	103 kDa	0.80681	0	0.82985	1.06	0	1.5708	0	0
tr|A0A6J3K2P1|A0A6J3K2P1_9HYME-DECOY	?	0.79	1.0275	0.82985	0	0.68425	1.2259		0	
chymotrypsin-1-like OS=Bombus vosnesenskii OX=207650 GN=LOC117241961 PE=4 SV=1	A0A6J3LJU9	33 kDa	0	1.0275	0.82985	0	1.6763	0	2.4518	0
apoptosis-inducing factor 1, mitochondrial OS=Bombus vosnesenskii OX=207650 GN=LOC117234260 PE=3 SV=1	A0A6J3KDD 4	75 kDa	0.80681	0	1.6597	1.06	0.83814	0	0	0
3-oxoacyl-[acyl-carrier-protein] synthase OS=Bombus vosnesenskii OX=207650 GN=LOC117239692 PE=3 SV=1	A0A6J3L6V1 (+2)	47 kDa	0	1.0275	0.82985	2.12	0.83814	0	1.2259	0
Inositol-1-monophosphatase OS=Bombus vosnesenskii OX=207650	A0A6J3LGI0	30 kDa	0.80681	0	0	0	0.83814	0	1.2259	0

GN=LOC117242151 PE=3 SV=1										
probable serine hydrolase isoform X2 OS=Bombus vosnesenskii OX=207650 GN=LOC117235382 PE=4 SV=1	A0A6J3KJA1 (+4)	36 kDa	0.80681	0	2.4896	0	0	0	1.2259	1.8798
magnesium-dependent phosphatase 1-like OS=Bombus vosnesenskii OX=207650 GN=LOC117233483 PE=4 SV=1	A0A6J3KCJ4	18 kDa	1.6136	0	0	0	0.83814	1.5708	1.2259	0
Pre-mRNA-processing factor 19 OS=Bombus vosnesenskii OX=207650 GN=LOC117235501 PE=3 SV=1	A0A6J3KMQ2	55 kDa	0.80681	1.0275	0.82985	1.06	0	0	0	0
Tripeptidyl-peptidase 2 OS=Bombus vosnesenskii OX=207650 GN=LOC117243560 PE=3 SV=1	A0A6J3LNJ0	254 kDa	1.6136	1.0275	1.6597	0	0	0	0	0
protein krasavietz OS=Bombus vosnesenskii OX=207650 GN=LOC117240218 PE=3 SV=1	A0A6J3LBN5	48 kDa	1.6136	2.0551	0.82985	0	0	0	0	0
endoplasmic reticulum resident protein 44 isoform X1 OS=Bombus vosnesenskii OX=207650 GN=LOC117237961 PE=4 SV=1	A0A6J3KY53 (+1)	49 kDa	1.6136	0	0	0	1.6763	1.5708	0	0
Coatomer subunit gamma OS=Bombus vosnesenskii OX=207650 GN=LOC117241652 PE=3 SV=1	A0A6J3LDS3	96 kDa	1.6136	3.0826	0.82985	0	0	0	0	0
persulfide dioxygenase ETHE1, mitochondrial isoform X1 OS=Bombus vosnesenskii OX=207650 GN=LOC117234587 PE=4 SV=1	A0A6J3KHQ4 (+1)	31 kDa	2.4204	0	1.6597	0	0.83814	0	0	0
Poly [ADP-ribose] polymerase OS=Bombus vosnesenskii OX=207650 GN=LOC117235235 PE=3 SV=1	A0A6J3KIH5 (+1)	113 kDa	1.6136	1.0275	0.82985	0	0	0	0	0

uncharacterized protein LOC117231055 OS=Bombus vosnesenskii OX=207650 GN=LOC117231055 PE=3 SV=1	A0A6J3JW01	24 kDa	1.6136	0	1.6597	0	0	0	1.2259	0
leucine-rich repeat receptor-like protein kinase PEPR1 isoform X2 OS=Bombus vosnesenskii OX=207650 GN=LOC117243489 PE=4 SV=1	A0A6J3LN41 (+1)	99 kDa	0.80681	2.0551	1.6597	0	0	0	0	0
ubiquilin-4 OS=Bombus vosnesenskii OX=207650 GN=LOC117243530 PE=4 SV=1	A0A6J3LSA0	58 kDa	1.6136	2.0551	1.6597	1.06	0	0	0	0
Multiple inositol polyphosphate phosphatase 1 OS=Bombus vosnesenskii OX=207650 GN=LOC117243022 PE=3 SV=1	A0A6J3LLI7	55 kDa	0.80681	0	0	1.06	2.5144	0	0	0
multidrug resistance protein homolog 49 isoform X1 OS=Bombus vosnesenskii OX=207650 GN=LOC117236934 PE=4 SV=1	A0A6J3KWM2 (+1)	148 kDa	0.80681	1.0275	0.82985	0	0	0	0	0
medium-chain acyl-CoA ligase ACSF2, mitochondrial isoform X2 OS=Bombus vosnesenskii OX=207650 GN=LOC117238082 PE=3 SV=1	A0A6J3KZ09 (+1)	69 kDa	0.80681	0	0.82985	1.06	0	0	0	0
spectrin beta chain, non-erythrocytic 5 isoform X2 OS=Bombus vosnesenskii OX=207650 GN=LOC117232847 PE=3 SV=1	A0A6J3K5S5 (+3)	488 kDa	1.6136	0	0.82985	0	1.6763	0	0	0
retinol dehydrogenase 12 OS=Bombus vosnesenskii OX=207650 GN=LOC117236723 PE=3 SV=1	A0A6J3KRU1	41 kDa	1.6136	1.0275	0	0	0	0	0	0
hemocyte protein-glutamine gamma-glutamyltransferase-like OS=Bombus vosnesenskii OX=207650 GN=LOC117241966 PE=3 SV=1	A0A6J3LG18	80 kDa	0	1.0275	0.82985	1.06	0	0	0	0

lethal(2) giant larvae protein homolog 1 isoform X2 OS=Bombus vosnesenskii OX=207650 GN=LOC117243572 PE=3 SV=1	A0A6J3LNK5 (+7)	129 kDa	0	3.0826	1.6597	0	0	0	0	0
26S proteasome non-ATPase regulatory subunit 2 OS=Bombus vosnesenskii OX=207650 GN=LOC117236305 PE=3 SV=1	A0A6J3KSF1	99 kDa	3.2272	0	0.82985	0	0	0	0	0
Flavin-containing monooxygenase OS=Bombus vosnesenskii OX=207650 GN=LOC117237032 PE=3 SV=1	A0A6J3KTZ9	48 kDa	0	0	0	0	3.3526	0	0	0
omega-conotoxin-like protein 1 OS=Bombus vosnesenskii OX=207650 GN=LOC117232521 PE=4 SV=1	A0A6J3K3Y1	8 kDa	0	0	0	0	3.3526	0	0	0
neprilysin-like OS=Bombus vosnesenskii OX=207650 GN=LOC117237088 PE=3 SV=1	A0A6J3KT95	85 kDa	0.80681	0	0	0	5.0288	0	0	0
ATP synthase subunit d, mitochondrial OS=Bombus vosnesenskii OX=207650 GN=LOC117231980 PE=3 SV=1	A0A6J3K335	20 kDa	0.80681	1.0275	0.82985	1.06	0	0	0	0
uncharacterized protein LOC117238050 OS=Bombus vosnesenskii OX=207650 GN=LOC117238050 PE=3 SV=1	A0A6J3KZT2	13 kDa	1.6136	1.0275	0.82985	0	0	1.5708	0	0
UBX domain-containing protein 4 isoform X3 OS=Bombus vosnesenskii OX=207650 GN=LOC117231780 PE=4 SV=1	A0A6J3K1K4 (+2)	59 kDa	0.80681	1.0275	0.82985	2.12	0	0	1.2259	0
adrenodoxin-like protein 2, mitochondrial OS=Bombus vosnesenskii OX=207650 GN=LOC117239011 PE=4 SV=1	A0A6J3L3E5 (+20)	18 kDa	0.80681	1.0275	1.6597	1.06	0	0	0	0
extracellular serine/threonine protein CG31145 isoform X1 OS=Bombus vosnesenskii OX=207650	A0A6J3LPP3	59 kDa	0.80681	0	0.82985	1.06	0.83814	0	0	0

GN=LOC117242970 PE=3 SV=1										
ero1-like protein isoform X1 OS=Bombus vosnesenskii OX=207650 GN=LOC117237671 PE=3 SV=1	A0A6J3L113 (+1)	55 kDa	0.80681	0	1.6597	1.06	0	1.5708	0	0
long-chain fatty acid transport protein 4 isoform X1 OS=Bombus vosnesenskii OX=207650 GN=LOC117235573 PE=3 SV=1	A0A6J3KN96 (+1)	87 kDa	0.80681	2.0551	0.82985	1.06	0.83814	0	0	0
elongin-B OS=Bombus vosnesenskii OX=207650 GN=LOC117240409 PE=4 SV=1	A0A6J3LAF9	13 kDa	0.80681	1.0275	1.6597	1.06	0	0	1.2259	0
Trehalase OS=Bombus vosnesenskii OX=207650 GN=LOC117242995 PE=3 SV=1	A0A6J3LLC0	67 kDa	0.80681	2.0551	0.82985	1.06	0	0	1.2259	0
actin-related protein 2/3 complex subunit 1A-A isoform X3 OS=Bombus vosnesenskii OX=207650 GN=LOC117236184 PE=3 SV=1	A0A6J3KNI1 (+2)	35 kDa	1.6136	0	0	1.06	0	1.5708	0	0
mitochondrial amidoxime-reducing component 1 OS=Bombus vosnesenskii OX=207650 GN=LOC117236942 PE=4 SV=1	A0A6J3KVX0	38 kDa	0.80681	1.0275	0	2.12	0.83814	0	0	0
uncharacterized protein LOC117237144 isoform X2 OS=Bombus vosnesenskii OX=207650 GN=LOC117237144 PE=4 SV=1	A0A6J3KX42 (+1)	39 kDa	1.6136	1.0275	0	0	0	1.5708	1.2259	0
uncharacterized protein LOC117237352 OS=Bombus vosnesenskii OX=207650 GN=LOC117237352 PE=4 SV=1	A0A6J3KVG9	28 kDa	0.80681	0	1.6597	1.06	1.6763	0	0	0
FAS-associated factor 2 OS=Bombus vosnesenskii OX=207650 GN=LOC117237411 PE=4 SV=1	A0A6J3KZH1	51 kDa	0.80681	1.0275	1.6597	0	0	0	0	0

probable histone-binding protein Caf1 OS=Bombus vosnesenskii OX=207650 GN=LOC117235627 PE=4 SV=1	A0A6J3KPD3	48 kDa	0.80681	1.0275	0.82985	0	0	0	0	0
nuclear pore complex protein Nup214-like OS=Bombus vosnesenskii OX=207650 GN=LOC117230579 PE=4 SV=1	A0A6J3JVH6	174 kDa	0	0	1.6597	1.06	0.83814	0	0	0
Sepiapterin reductase OS=Bombus vosnesenskii OX=207650 GN=LOC117241643 PE=3 SV=1	A0A6J3LCY4	29 kDa	0	1.0275	0.82985	0	2.5144	0	0	0
ER membrane protein complex subunit 1 OS=Bombus vosnesenskii OX=207650 GN=LOC117235762 PE=3 SV=1	A0A6J3KLU2 (+1)	104 kDa	1.6136	0	0.82985	0	0	1.5708	0	0
laminin subunit beta-1 isoform X2 OS=Bombus vosnesenskii OX=207650 GN=LOC117231856 PE=4 SV=1	A0A6J3K258 (+1)	198 kDa	0.80681	1.0275	1.6597	0	0	0	0	0
hemocytin OS=Bombus vosnesenskii OX=207650 GN=LOC117242485 PE=3 SV=1	A0A6J3LMD2	440 kDa	1.6136	1.0275	0.82985	0	0	0	0	0
Serine hydroxymethyltransferase OS=Bombus vosnesenskii OX=207650 GN=LOC117231296 PE=3 SV=1	A0A6J3JZC9	58 kDa	1.6136	0	0.82985	1.06	0	0	0	0
Dolichyl-diphosphooligosaccharide--protein glycosyltransferase subunit DAD1 OS=Bombus vosnesenskii OX=207650 GN=LOC117236423 PE=3 SV=1	A0A6J3KT14	13 kDa	0.80681	1.0275	1.6597	0	0	0	0	0
constitutive coactivator of PPAR-gamma-like protein 1 homolog OS=Bombus vosnesenskii OX=207650 GN=LOC117236563 PE=3 SV=1	A0A6J3KUJ0	109 kDa	1.6136	1.0275	0	1.06	0	0	0	0
insulin-like growth factor-binding protein complex acid labile	A0A6J3KZ31	38 kDa	1.6136	0	0	0	0	1.5708	0	0

subunit OS=Bombus vosnesenskii OX=207650 GN=LOC117237995 PE=4 SV=1										
Proteasome subunit alpha type OS=Bombus vosnesenskii OX=207650 GN=LOC117240090 PE=3 SV=1	A0A6J3LB30	29 kDa	0	1.0275	0.82985	0	0	0	0	0
CD109 antigen OS=Bombus vosnesenskii OX=207650 GN=LOC117241897 PE=4 SV=1	A0A6J3LH90	186 kDa	0	2.0551	1.6597	0	0	0	0	0
26S proteasome non-ATPase regulatory subunit 1 OS=Bombus vosnesenskii OX=207650 GN=LOC117234572 PE=3 SV=1	A0A6J3KFE9	112 kDa	1.6136	0	0.82985	0	0	0	0	0
uncharacterized protein LOC117242415 OS=Bombus vosnesenskii OX=207650 GN=LOC117242415 PE=4 SV=1	A0A6J3LHR0	58 kDa	2.4204	1.0275	0.82985	0	0	0	0	0
plexin-A4 OS=Bombus vosnesenskii OX=207650 GN=LOC117237775 PE=3 SV=1	A0A6J3KYG6	216 kDa	0	0	1.6597	3.18	0	0	0	0
uncharacterized protein LOC117230536 OS=Bombus vosnesenskii OX=207650 GN=LOC117230536 PE=4 SV=1	A0A6J3JUJ1	14 kDa	0	0	0	0	2.5144	0	0	0
N-terminal acetyltransferase B complex subunit MDM20 homolog OS=Bombus vosnesenskii OX=207650 GN=LOC117243587 PE=3 SV=1	A0A6J3LNQ5	104 kDa	0	2.0551	0	0	0	0	0	0
Small ubiquitin-related modifier OS=Bombus vosnesenskii OX=207650 GN=LOC117239587 PE=3 SV=1	A0A6J3L6D7	11 kDa	0	0	0	0	2.5144	0	0	0
heat shock 70 kDa protein 4 isoform X1 OS=Bombus vosnesenskii OX=207650 GN=LOC117234768 PE=3 SV=1	A0A6J3KGB6 (+1)	98 kDa	3.2272	0	0	0	0	0	0	0

C-1-tetrahydrofolate synthase, cytoplasmic OS=Bombus vosnesenskii OX=207650 GN=LOC117234666 PE=3 SV=1	A0A6J3KGG2 (+1)	101 kDa	0	0	0	0	0	1.5708	0	0
NADH dehydrogenase [ubiquinone] 1 beta subcomplex subunit 8, mitochondrial OS=Bombus vosnesenskii OX=207650 GN=LOC117230856 PE=4 SV=1	A0A6J3JUY7	21 kDa	0	1.0275	0	0	0	0	0	0
Uridine 5'-monophosphate synthase OS=Bombus vosnesenskii OX=207650 GN=LOC117242782 PE=3 SV=1	A0A6J3LLL7 (+1)	52 kDa	1.6136	0	0	0	0.83814	1.5708	0	0
tryptase-like isoform X1 OS=Bombus vosnesenskii OX=207650 GN=LOC117230419 PE=4 SV=1	A0A6J3JUG1 (+2)	46 kDa	1.6136	0	0.82985	0	0	1.5708	0	0
40S ribosomal protein S18 OS=Bombus vosnesenskii OX=207650 GN=LOC117243033 PE=3 SV=1	A0A6J3LMZ0 (+1)	18 kDa	0.80681	2.0551	0.82985	0	0	0	0	0
sodium/calcium exchanger regulatory protein 1-like OS=Bombus vosnesenskii OX=207650 GN=LOC117234134 PE=3 SV=1	A0A6J3KG83	15 kDa	0.80681	1.0275	0	1.06	0	0	0	0
uncharacterized protein LOC117239635 OS=Bombus vosnesenskii OX=207650 GN=LOC117239635 PE=4 SV=1	A0A6J3L7L7	49 kDa	0.80681	1.0275	1.6597	1.06	0	0	0	0
Mitochondrial pyruvate carrier OS=Bombus vosnesenskii OX=207650 GN=LOC117234818 PE=3 SV=1	A0A6J3KH87 (+1)	14 kDa	1.6136	0	0.82985	0	0	1.5708	0	0
icarapin-like OS=Bombus vosnesenskii OX=207650 GN=LOC117234375 PE=4 SV=1	A0A6J3KHK7	25 kDa	1.6136	1.0275	0.82985	0	0.83814	0	0	0
glutamine--tRNA ligase OS=Bombus vosnesenskii OX=207650	A0A6J3KQX2 (+2)	89 kDa	1.6136	0	0.82985	1.06	0	0	0	0

GN=LOC117235903 PE=3 SV=1										
fatty-acid amide hydrolase 2-like isoform X1 OS=Bombus vosnesenskii OX=207650 GN=LOC117235615 PE=4 SV=1	A0A6J3KKK3 (+1)	61 kDa	0.80681	2.0551	0	0	0	1.5708	0	0
phosphatidylinositol-binding clathrin assembly protein unc-11 isoform X3 OS=Bombus vosnesenskii OX=207650 GN=LOC117242523 PE=3 SV=1	A0A6J3LHZ3 (+23)	96 kDa	0.80681	0	1.6597	1.06	0	0	1.2259	0
very low-density lipoprotein receptor-like isoform X2 OS=Bombus vosnesenskii OX=207650 GN=LOC117232607 PE=4 SV=1	A0A6J3K4C5 (+8)	101 kDa	0.80681	1.0275	0.82985	0	0	0	0	0
Glucosamine 6-phosphate N-acetyltransferase OS=Bombus vosnesenskii OX=207650 GN=LOC117232535 PE=3 SV=1	A0A6J3K412	22 kDa	0.80681	1.0275	1.6597	0	0	0	0	0
Vacuolar protein sorting-associated protein 29 OS=Bombus vosnesenskii OX=207650 GN=LOC117237479 PE=3 SV=1	A0A6J3KVG5	21 kDa	0.80681	1.0275	1.6597	0	0	0	0	0
integrin alpha-PS2 isoform X4 OS=Bombus vosnesenskii OX=207650 GN=LOC117233588 PE=3 SV=1	A0A6J3K9Y8 (+5)	162 kDa	0	1.0275	0	0	0.83814	0	0	0
exportin-1 OS=Bombus vosnesenskii OX=207650 GN=LOC117240370 PE=3 SV=1	A0A6J3LC98	123 kDa	1.6136	1.0275	0.82985	0	0	0	0	0
eukaryotic peptide chain release factor GTP-binding subunit ERF3A OS=Bombus vosnesenskii OX=207650 GN=LOC117236319 PE=3 SV=1	A0A6J3KPJ0	66 kDa	0.80681	0	0.82985	1.06	0	0	0	0
tr|A0A6J3L0T6|A0A6J3L0T6_9HYME-DECOY	?	0.34	0	0	0	0.68425	0		0	
CCAAT/enhancer-binding protein zeta isoform X3 OS=Bombus vosnesenskii	A0A6J3LDH6 (+1)	125 kDa	1.6136	1.0275	1.6597	0	0	0	0	0

OX=207650 GN=LOC117241612 PE=3 SV=1										
6-pyruvoyl tetrahydrobiopterin synthase OS=Bombus vosnesenskii OX=207650 GN=LOC117239621 PE=3 SV=1	A0A6J3LBE4	17 kDa	2.4204	0	0	1.06	0	0	0	0
protein Malvolio isoform X1 OS=Bombus vosnesenskii OX=207650 GN=LOC117243198 PE=3 SV=1	A0A6J3LLI3 (+2)	71 kDa	0.80681	0	0.82985	0	0	0	0	0
26S proteasome non-ATPase regulatory subunit 10-like OS=Bombus vosnesenskii OX=207650 GN=LOC117237296 PE=4 SV=1	A0A6J3KV72	25 kDa	0.80681	0	0	0	0	1.5708	0	0
sp|CATD_HUMAN|	sp|CATD_HUMAN|	45 kDa	1.6136	1.0275	0	0	0	0	2.4518	0
protein angel OS=Bombus vosnesenskii OX=207650 GN=LOC117239377 PE=4 SV=1	A0A6J3L661	64 kDa	0.80681	2.0551	1.6597	0	0	0	0	0
glycogen synthase kinase-3 beta-like isoform X5 OS=Bombus vosnesenskii OX=207650 GN=LOC117234091 PE=3 SV=1	A0A6J3KCM3 (+8)	50 kDa	0	0	0	1.06	1.6763	0	0	0
phosphoglucomutase-2 OS=Bombus vosnesenskii OX=207650 GN=LOC117238741 PE=3 SV=1	A0A6J3L703	69 kDa	1.6136	1.0275	1.6597	0	0	0	0	0
Prohibitin OS=Bombus vosnesenskii OX=207650 GN=LOC117231645 PE=3 SV=1	A0A6J3K0T6	30 kDa	0.80681	1.0275	0	0	0	0	0	0
uncharacterized protein LOC117235222 OS=Bombus vosnesenskii OX=207650 GN=LOC117235222 PE=4 SV=1	A0A6J3KIE1 (+1)	76 kDa	0	0	0	0	0.83814	0	3.6777	0
alpha-1,2-Mannosidase OS=Bombus vosnesenskii OX=207650 GN=LOC117232324 PE=3 SV=1	A0A6J3K4S5	65 kDa	1.6136	0	1.6597	0	0	0	0	0

methanethiol oxidase OS=Bombus vosnesenskii OX=207650 GN=LOC117243436 PE=3 SV=1	A0A6J3LNT8	53 kDa	0	0	1.6597	0	0	0	0	0
uncharacterized protein LOC117231228 OS=Bombus vosnesenskii OX=207650 GN=LOC117231228 PE=4 SV=1	A0A6J3JX31	93 kDa	2.4204	0	0	0	0.83814	0	0	0
adenosine deaminase OS=Bombus vosnesenskii OX=207650 GN=LOC117231787 PE=3 SV=1	A0A6J3K1S5	58 kDa	0	0	0	0	2.5144	0	0	0
Translin OS=Bombus vosnesenskii OX=207650 GN=LOC117243187 PE=3 SV=1	A0A6J3LQP 8	27 kDa	0	0	3.3194	0	0	0	0	0
aminopeptidase N OS=Bombus vosnesenskii OX=207650 GN=LOC117242943 PE=3 SV=1	A0A6J3LK60	112 kDa	0.80681	2.0551	0.82985	0	0	0	0	0
protein argonaute-2 isoform X1 OS=Bombus vosnesenskii OX=207650 GN=LOC117234637 PE=3 SV=1	A0A6J3KFG 6 (+3)	116 kDa	0	0	0.82985	1.06	0	0	0	0
beta-1-syntrophin isoform X2 OS=Bombus vosnesenskii OX=207650 GN=LOC117230365 PE=3 SV=1	A0A6J3JTH9 (+1)	61 kDa	0	0	0.82985	0	0.83814	0	0	0
Elongation of very long chain fatty acids protein OS=Bombus vosnesenskii OX=207650 GN=LOC117239420 PE=3 SV=1	A0A6J3L5D7	35 kDa	1.6136	0	0	0	0	1.5708	0	0
Glucosidase 2 subunit beta OS=Bombus vosnesenskii OX=207650 GN=LOC117236854 PE=4 SV=1	A0A6J3KSN 1	61 kDa	0.80681	0	0.82985	0	0	0	0	0
esterase FE4-like isoform X3 OS=Bombus vosnesenskii OX=207650 GN=LOC117237667 PE=4 SV=1	A0A6J3KXS 3	75 kDa	0.80681	0	0.82985	0	0	0	0	0
transmembrane protein 214-A OS=Bombus vosnesenskii	A0A6J3L402	77 kDa	1.6136	1.0275	0	0	0	0	0	0

OX=207650 GN=LOC117238407 PE=3 SV=1										
long-chain-fatty-acid--CoA ligase 3 isoform X3 OS=Bombus vosnesenskii OX=207650 GN=LOC117230337 PE=3 SV=1	A0A6J3JS10 (+2)	80 kDa	1.6136	0	0.82985	0	0	0	0	0
transmembrane protein 104-like isoform X2 OS=Bombus vosnesenskii OX=207650 GN=LOC117242142 PE=3 SV=1	A0A6J3LFW 3 (+4)	61 kDa	0.80681	0	0.82985	0	0	0	0	0
uncharacterized protein LOC117232587 OS=Bombus vosnesenskii OX=207650 GN=LOC117232587 PE=4 SV=1	A0A6J3K615	16 kDa	0.80681	0	1.6597	1.06	0	0	0	0
scavenger receptor class B member 1-like OS=Bombus vosnesenskii OX=207650 GN=LOC117242877 PE=3 SV=1	A0A6J3LKT0	58 kDa	1.6136	1.0275	0	0	0	0	1.2259	0
Eukaryotic translation initiation factor 3 subunit K OS=Bombus vosnesenskii OX=207650 GN=LOC117236448 PE=3 SV=1	A0A6J3KTX 2	25 kDa	1.6136	0	0.82985	0	0	0	0	0
GDP-mannose 4,6-dehydratase OS=Bombus vosnesenskii OX=207650 GN=LOC117235257 PE=3 SV=1	A0A6J3KIN2	40 kDa	1.6136	0	0	0	0.83814	0	0	0
Septin OS=Bombus vosnesenskii OX=207650 GN=LOC117230866 PE=3 SV=1	A0A6J3JWV 9	49 kDa	0	0	0.82985	0	0	1.5708	0	0
prion-like-(Q/N-rich) domain-bearing protein 25 OS=Bombus vosnesenskii OX=207650 GN=LOC117240088 PE=4 SV=1	A0A6J3LD52	40 kDa	1.6136	0	0.82985	1.06	0	0	0	0
uncharacterized protein YJR142W OS=Bombus vosnesenskii OX=207650 GN=LOC117237638 PE=4 SV=1	A0A6J3KX15	37 kDa	1.6136	0	0.82985	0	0	0	0	0
dnaJ homolog subfamily C member 3 OS=Bombus	A0A6J3KFL8	55 kDa	0.80681	1.0275	0	0	0	0	0	0

vosnesenskii OX=207650 GN=LOC117234620 PE=4 SV=1										
metal transporter CNNM4 isoform X1 OS=Bombus vosnesenskii OX=207650 GN=LOC117230873 PE=3 SV=1	A0A6J3JV05 (+1)	113 kDa	1.6136	0	0.82985	0	0	0	0	0
Copper homeostasis protein cutC homolog OS=Bombus vosnesenskii OX=207650 GN=LOC117240202 PE=3 SV=1	A0A6J3LDR5	26 kDa	0.80681	0	0	0	0	0	0	0
DNA polymerase OS=Bombus vosnesenskii OX=207650 GN=LOC117238506 PE=3 SV=1	A0A6J3L259	39 kDa	0.80681	0	0.82985	2.12	0	0	0	0
uncharacterized protein CG43427 isoform X1 OS=Bombus vosnesenskii OX=207650 GN=LOC117238977 PE=4 SV=1	A0A6J3L383 (+2)	202 kDa	0	2.0551	0	0	0	1.5708	0	0
Prostaglandin E synthase 2 OS=Bombus vosnesenskii OX=207650 GN=LOC117237143 PE=3 SV=1	A0A6J3KY29	46 kDa	1.6136	0	0	0	0.83814	0	0	0
F-actin-capping protein subunit alpha OS=Bombus vosnesenskii OX=207650 GN=LOC117239225 PE=3 SV=1	A0A6J3L5K6	33 kDa	0	0	0.82985	0	1.6763	0	0	0
Moesin/ezrin/radixin homolog 1 OS=Bombus vosnesenskii OX=207650 GN=LOC117242173 PE=4 SV=1	A0A6J3LGK9 (+2)	73 kDa	0	0	0	0	1.6763	0	0	0
Mitochondrial import inner membrane translocase subunit OS=Bombus vosnesenskii OX=207650 GN=LOC117235971 PE=3 SV=1	A0A6J3KQM1	10 kDa	0	0	0	0	1.6763	0	0	0
mitochondrial dicarboxylate carrier OS=Bombus vosnesenskii OX=207650 GN=LOC117239266 PE=3 SV=1	A0A6J3L7T4	32 kDa	1.6136	2.0551	0	0	0	0	0	0

uncharacterized protein LOC117231202 isoform X2 OS=Bombus vosnesenskii OX=207650 GN=LOC117231202 PE=4 SV=1	A0A6J3JYS0 (+1)	192 kDa	0	0	0	0	1.6763	0	0	0
cysteine desulfurase OS=Bombus vosnesenskii OX=207650 GN=LOC117231589 PE=3 SV=1	A0A6J3JYZ9 (+1)	50 kDa	1.6136	0	0	0	0	0	0	0
Integrin beta OS=Bombus vosnesenskii OX=207650 GN=LOC117231480 PE=3 SV=1	A0A6J3K0P9	93 kDa	0	1.0275	0	0	0	0	0	0
hydroxyacid oxidase 1 OS=Bombus vosnesenskii OX=207650 GN=LOC117237194 PE=3 SV=1	A0A6J3KUS 5	41 kDa	2.4204	0	0.82985	0	0	0	0	0
Serine/threonine-protein phosphatase OS=Bombus vosnesenskii OX=207650 GN=LOC117235790 PE=3 SV=1	A0A6J3KQ6 8	35 kDa	2.4204	0	0.82985	0	0	0	0	0
V-type proton ATPase subunit H OS=Bombus vosnesenskii OX=207650 GN=LOC117235695 PE=3 SV=1	A0A6J3KLJ5	56 kDa	0	0	0.82985	0	0	0	0	0
NADH-ubiquinone oxidoreductase 75 kDa subunit, mitochondrial OS=Bombus vosnesenskii OX=207650 GN=LOC117235548 PE=3 SV=1	A0A6J3KNX 9	80 kDa	0	0	0	0	0	0	0	0
muscle M-line assembly protein unc-89-like isoform X1 OS=Bombus vosnesenskii OX=207650 GN=LOC117242037 PE=4 SV=1	A0A6J3LHW 6 (+1)	230 kDa	0	0	1.6597	0	0.83814	0	0	0
Mitochondrial fission 1 protein OS=Bombus vosnesenskii OX=207650 GN=LOC117237776 PE=3 SV=1	A0A6J3KXW 1	17 kDa	0	0	0	0	0.83814	0	0	0
uncharacterized protein LOC117240514 isoform X1 OS=Bombus vosnesenskii	A0A6J3LCM 2 (+1)	47 kDa	0	0	0	0	0.83814	0	0	0

OX=207650 GN=LOC117240514 PE=4 SV=1										
serine/arginine repetitive matrix protein 2 isoform X1 OS=Bombus vosnesenskii OX=207650 GN=LOC117231749 PE=4 SV=1	A0A6J3JZQ0 (+1)	145 kDa	0	0	0	0	0.83814	0	0	0
Annexin OS=Bombus vosnesenskii OX=207650 GN=LOC117236290 PE=3 SV=1	A0A6J3KSC3 (+1)	36 kDa	0	1.0275	0	0	0	3.1415	0	0
ADP-ribose pyrophosphatase, mitochondrial OS=Bombus vosnesenskii OX=207650 GN=LOC117240012 PE=4 SV=1	A0A6J3LAR4	35 kDa	0	0	0	0	0.83814	0	0	0
ankyrin-2-like isoform X2 OS=Bombus vosnesenskii OX=207650 GN=LOC117230732 PE=3 SV=1	A0A6J3JU94 (+5)	654 kDa	0	0	0	0	1.6763	0	0	0
leukocyte elastase inhibitor OS=Bombus vosnesenskii OX=207650 GN=LOC117238287 PE=3 SV=1	A0A6J3L036	76 kDa	0	0	0	0	1.6763	0	0	0
congested-like trachea protein isoform X2 OS=Bombus vosnesenskii OX=207650 GN=LOC117243018 PE=3 SV=1	A0A6J3LKK0 (+1)	28 kDa	1.6136	0	0	0	0	0	0	0
troponin I 2-like isoform X3 OS=Bombus vosnesenskii OX=207650 GN=LOC117236763 PE=4 SV=1	A0A6J3KS09 (+2)	46 kDa	0	0	0	0	0.83814	0	0	0
Transferrin OS=Bombus vosnesenskii OX=207650 GN=LOC117230686 PE=3 SV=1	A0A6J3JU29 (+3)	81 kDa	0	0	0	0	0.83814	0	0	0
methyltransferase-like 26 OS=Bombus vosnesenskii OX=207650 GN=LOC117239091 PE=3 SV=1	A0A6J3L3S9 (+1)	25 kDa	0	0	0	0	0	0	1.2259	0
protein Mo25 isoform X1 OS=Bombus vosnesenskii OX=207650	A0A6J3KP79 (+1)	39 kDa	0	0	0	0	0	0	1.2259	0

GN=LOC117236254 PE=3 SV=1										
60S ribosomal protein L37a OS=Bombus vosnesenskii OX=207650 GN=LOC117239523 PE=3 SV=1	A0A6J3L727	10 kDa	0	1.0275	1.6597	0	0	0	0	0
NFU1 iron-sulfur cluster scaffold homolog, mitochondrial OS=Bombus vosnesenskii OX=207650 GN=LOC117235792 PE=3 SV=1	A0A6J3KLH3	31 kDa	0	0	0.82985	0	0	0	0	0
AP-2 complex subunit mu OS=Bombus vosnesenskii OX=207650 GN=LOC117231777 PE=3 SV=1	A0A6J3JZW2	50 kDa	0.80681	0	0	0	0	0	0	0
NADH dehydrogenase [ubiquinone] flavoprotein 1, mitochondrial OS=Bombus vosnesenskii OX=207650 GN=LOC117237617 PE=3 SV=1	A0A6J3KW85	52 kDa	0.80681	0	0	0	0	0	0	0
von Willebrand factor A domain-containing protein 8 isoform X1 OS=Bombus vosnesenskii OX=207650 GN=LOC117234315 PE=4 SV=1	A0A6J3KG27 (+1)	209 kDa	1.6136	1.0275	0	0	0	0	0	0
integrin-linked protein kinase homolog pat-4 OS=Bombus vosnesenskii OX=207650 GN=LOC117235061 PE=4 SV=1	A0A6J3KHM0	51 kDa	1.6136	0	0	1.06	0	0	0	0
tr|A0A6J3K8X3|A0A6J3K8X3_9HYME-DECOY	tr|A0A6J3K8X3|A0A6J3K8X3_9HYME-DECOY (+5)	?	0	0	1.6597	0	0	0	1.2259	0
integrin alpha-PS1 isoform X1 OS=Bombus vosnesenskii OX=207650 GN=LOC117241693 PE=3 SV=1	A0A6J3LEF1 (+1)	123 kDa	0	1.0275	0	0	0	0	0	0
LOW QUALITY PROTEIN: neprilysin-11-like OS=Bombus vosnesenskii OX=207650 GN=LOC117242764 PE=3 SV=1	A0A6J3LJ80	130 kDa	0.80681	0	0	0	0	0	0	0

casein kinase II subunit alpha isoform X1 OS=Bombus vosnesenskii OX=207650 GN=LOC117236292 PE=3 SV=1	A0A6J3KP22 (+1)	55 kDa	0	1.0275	0	0	0	0	0	0
cyclic AMP-dependent transcription factor ATF-6 alpha isoform X1 OS=Bombus vosnesenskii OX=207650 GN=LOC117235516 PE=4 SV=1	A0A6J3KK22 (+1)	92 kDa	1.6136	1.0275	0	0	0	0	0	0
calcium-binding mitochondrial carrier protein Aralar1 isoform X1 OS=Bombus vosnesenskii OX=207650 GN=LOC117236528 PE=3 SV=1	A0A6J3KQ80 (+5)	78 kDa	0	0	0	0	0	0	0	0
glutaminyl-peptide cyclotransferase OS=Bombus vosnesenskii OX=207650 GN=LOC117243577 PE=3 SV=1	A0A6J3LQP4	43 kDa	0	0	1.6597	0	0	0	0	0
Eukaryotic translation initiation factor 3 subunit L OS=Bombus vosnesenskii OX=207650 GN=LOC117231211 PE=3 SV=1	A0A6J3JYU1	63 kDa	2.4204	0	0	0	0	0	0	0
pregnancy zone protein-like OS=Bombus vosnesenskii OX=207650 GN=LOC117235080 PE=3 SV=1	A0A6J3KHP5	197 kDa	0	0	2.4896	0	0	0	0	0
NADH dehydrogenase [ubiquinone] 1 alpha subcomplex subunit 10, mitochondrial OS=Bombus vosnesenskii OX=207650 GN=LOC117242625 PE=3 SV=1	A0A6J3LJB0	49 kDa	0	0	0	0	0	0	0	0
sushi, von Willebrand factor type A, EGF and pentraxin domain-containing protein 1 isoform X2 OS=Bombus vosnesenskii OX=207650 GN=LOC117234975 PE=4 SV=1	A0A6J3KH15 (+1)	387 kDa	0	0	0	0	0	0	0	0
cAMP-dependent protein kinase type II regulatory subunit isoform X1 OS=Bombus vosnesenskii OX=207650	A0A6J3JS99 (+1)	44 kDa	0	0	0	0	1.6763	0	0	0

GN=LOC117230367 PE=3 SV=1										
pleckstrin homology domain-containing family F member 2 OS=Bombus vosnesenskii OX=207650 GN=LOC117238753 PE=4 SV=1	A0A6J3L5Q2	32 kDa	0	0	0	0	1.6763	0	0	0
26S proteasome non-ATPase regulatory subunit 12 OS=Bombus vosnesenskii OX=207650 GN=LOC117243043 PE=3 SV=1	A0A6J3LQ40	50 kDa	1.6136	0	0	0	0	0	0	0
LOW QUALITY PROTEIN: fibroin heavy chain-like OS=Bombus vosnesenskii OX=207650 GN=LOC117243521 PE=4 SV=1	A0A6J3LNB 2	80 kDa	0	0	0	0	0	3.1415	0	0
Alanine--tRNA ligase OS=Bombus vosnesenskii OX=207650 GN=LOC117230261 PE=3 SV=1	A0A6J3JRK3 (+1)	133 kDa	1.6136	0	0	0	0	0	0	0
GMP synthase (glutamine-hydrolyzing) OS=Bombus vosnesenskii OX=207650 GN=LOC117237941 PE=4 SV=1	A0A6J3KY25	79 kDa	0	0	0	0	1.6763	0	0	0
Neutral ceramidase OS=Bombus vosnesenskii OX=207650 GN=LOC117234945 PE=3 SV=1	A0A6J3KHN 8	80 kDa	0	0	1.6597	0	0	0	0	0
Adenosylhomocysteinase OS=Bombus vosnesenskii OX=207650 GN=LOC117237082 PE=3 SV=1	A0A6J3KXR 1 (+3)	59 kDa	0	0	0	0	0	0	0	0
acidic leucine-rich nuclear phosphoprotein 32 family member A isoform X1 OS=Bombus vosnesenskii OX=207650 GN=LOC117242949 PE=3 SV=1	A0A6J3LK76 (+1)	29 kDa	0	0	0	0	0	0	0	0
cyclin-dependent kinase 10 OS=Bombus vosnesenskii OX=207650	A0A6J3KLP5	46 kDa	0	0	0	0	0	0	0	0

GN=LOC117235729 PE=3 SV=1										
mannose-1-phosphate guanyltransferase alpha-A OS=Bombus vosnesenskii OX=207650 GN=LOC117243017 PE=4 SV=1	A0A6J3LMW2	46 kDa	0	0	0	0	0	0	0	0
Sphingomyelin phosphodiesterase OS=Bombus vosnesenskii OX=207650 GN=LOC117239126 PE=3 SV=1	A0A6J3L3Y1 (+1)	64 kDa	0	0	1.6597	0	0	0	0	0
tr|A0A6J3KHY4|A0A6J3KHY4_9HYME-DECOY	?	0.41	0	0	0	1.3685	0	0		
Phenylalanine--tRNA ligase beta subunit OS=Bombus vosnesenskii OX=207650 GN=LOC117232547 PE=3 SV=1	A0A6J3K450 (+1)	67 kDa	1.6136	0	0	0	0	0	0	0
tr|A0A6J3JZ41|A0A6J3JZ41_9HYME-DECOY	tr|A0A6J3JZ41|A0A6J3JZ41_9HYME-DECOY (+1)	?	0	0	0	0	2.12	0	0	0
uncharacterized protein LOC117236786 OS=Bombus vosnesenskii OX=207650 GN=LOC117236786 PE=4 SV=1	A0A6J3KRE9	177 kDa	0	0	0	0	0	0	0	0
tr|A0A6J3KE19|A0A6J3KE19_9HYME-DECOY	tr|A0A6J3KE19|A0A6J3KE19_9HYME-DECOY (+2)	?	0	0	0	1.6597	0	0	0	0

Supplementary Table S3. All proteins identified in captive low- and high-elevation venom samples (with four biological replicates each).

Proteins	Accession Number	Molecular Weight	High elevation Site 1	High elevation Site 2	High elevation Site 3	High elevation Site 4	Low elevation Site 1	Low elevation Site 2	Low elevation Site 3	Low elevation Site 4
transmembrane protease serine 9-like OS=Bombus vosnesenskii OX=207650 GN=LOC117231933 PE=4 SV=1	A0A6J3K2U7	61 kDa	2,593.70	2,438.30	2,370.30	2,353.10	2,321.70	1,811.70	1,958.60	2,490.90
phospholipase A2 OS=Bombus vosnesenskii OX=207650 GN=LOC117231696 PE=4 SV=1	A0A6J3K1V5	21 kDa	1,362.70	1,557.60	1,599.80	1,685.20	1,566.50	1,569.10	1,587.50	1,736.00
venom dipeptidyl peptidase 4 OS=Bombus vosnesenskii OX=207650 GN=LOC117232985 PE=4 SV=1	A0A6J3K7R6	89 kDa	1,260.80	1,197.50	1,223.40	1,142.90	1,035.90	959.15	1,205.20	1,316.20
CLIP domain-containing serine protease OS=Bombus vosnesenskii OX=207650 GN=LOC117237098 PE=3 SV=1	A0A6J3KUF5 (+1)	40 kDa	842.95	951.24	987.14	988.53	834.44	863.04	941.71	934.92
CLIP domain-containing serine protease OS=Bombus vosnesenskii OX=207650 GN=LOC117238063 PE=3 SV=1	A0A6J3L236	40 kDa	841.92	944.73	980.28	976.66	828.94	859.24	920.37	929.14
inducible metalloproteinase inhibitor protein-like OS=Bombus vosnesenskii OX=207650 GN=LOC117240279 PE=4 SV=1	A0A6J3LE39	11 kDa	771.93	1,003.30	795	1,003.40	967.65	869.7	919.44	878.11
venom acid phosphatase Acph-1-like isoform X1 OS=Bombus vosnesenskii OX=207650 GN=LOC117235967 PE=4 SV=1	A0A6J3KME8 (+1)	49 kDa	494.03	659.47	913.62	800.52	733.16	846.87	981.61	977.28
apolipophorins OS=Bombus vosnesenskii OX=207650 GN=LOC117238300 PE=4 SV=1	A0A6J3L183	375 kDa	537.26	425.18	507.78	398.78	457.95	484.33	412.87	378.4
platelet-derived growth factor subunit A isoform X1 OS=Bombus vosnesenskii OX=207650 GN=LOC117236816 PE=3 SV=1	A0A6J3KSG9	35 kDa	532.12	439.28	312.71	420.55	459.05	404.4	508.43	420.76
vascular endothelial growth factor A isoform X2	A0A6J3KV47	30 kDa	520.79	427.35	305.85	415.6	459.05	391.08	494.51	413.06

OS=Bombus vosnesenskii OX=207650 GN=LOC117236816 PE=3 SV=1										
venom protease-like OS=Bombus vosnesenskii OX=207650 GN=LOC117231932 PE=4 SV=1	A0A6J3K168	44 kDa	510.5	379.63	384.27	378	314.84	327.33	357.2	431.35
Hyaluronidase OS=Bombus vosnesenskii OX=207650 GN=LOC117242244 PE=3 SV=1	A0A6J3LGZ0	88 kDa	470.36	299.36	347.02	276.08	359.98	250.25	341.43	412.1
Transferrin OS=Bombus vosnesenskii OX=207650 GN=LOC117230525 PE=3 SV=1	A0A6J3JT60	78 kDa	381.85	260.32	248.99	252.33	267.51	200.77	233.8	146.35
melittin-like OS=Bombus vosnesenskii OX=207650 GN=LOC117232005 PE=4 SV=1	A0A6J3K401	6 kDa	159.53	167.04	177.43	209.78	288.42	154.15	163.29	185.83
Glyceraldehyde-3-phosphate dehydrogenase OS=Bombus vosnesenskii OX=207650 GN=LOC117230333 PE=3 SV=1	A0A6J3JT78	36 kDa	247.02	160.53	131.36	116.76	173.93	180.79	165.15	177.16
Bee-milk protein OS=Bombus vosnesenskii OX=207650 GN=LOC117233259 PE=3 SV=1	A0A6J3K9U6	96 kDa	285.1	148.6	119.59	207.8	168.43	81.832	116.9	166.57
sp|TRYP_PIG|	sp|TRYP_PIG|	24 kDa	154.39	127.99	133.32	124.68	134.3	127.51	131.75	133.83
venom protease-like OS=Bombus vosnesenskii OX=207650 GN=LOC117238613 PE=4 SV=1	A0A6J3L1M8	33 kDa	80.281	91.111	152.92	140.51	0	211.24	198.55	0
prosaposin OS=Bombus vosnesenskii OX=207650 GN=LOC117235656 PE=4 SV=1	A0A6J3KPJ1	104 kDa	166.74	106.3	115.67	128.64	128.8	113.23	139.17	124.21
uncharacterized protein LOC117238774 OS=Bombus vosnesenskii OX=207650 GN=LOC117238774 PE=3 SV=1	A0A6J3L3K9	100 kDa	19.556	113.89	68.619	128.64	48.437	123.7	142.88	157.91
LOW QUALITY PROTEIN: vitellogenin-like OS=Bombus vosnesenskii OX=207650	A0A6J3K186	202 kDa	106.01	111.72	60.777	99.942	67.152	125.6	89.996	47.179

GN=LOC117231930 PE=4 SV=1										
Superoxide dismutase [Cu-Zn] OS=Bombus vosnesenskii OX=207650 GN=LOC117238299 PE=3 SV=1	A0A6J3L104	16 kDa	95.719	79.18	85.284	58.382	103.48	79.929	77.007	87.618
sp|K2C1_HUMAN|	sp|K2C1_HUMAN|	66 kDa	84.398	90.026	81.363	63.329	51.74	94.202	76.079	48.142
Aconitate hydratase, mitochondrial OS=Bombus vosnesenskii OX=207650 GN=LOC117243148 PE=3 SV=1	A0A6J3LLK6	86 kDa	114.25	75.926	60.777	54.424	92.471	71.365	70.512	31.774
Fructose-bisphosphate aldolase OS=Bombus vosnesenskii OX=207650 GN=LOC117238722 PE=3 SV=1	A0A6J3L5K3	40 kDa	58.667	84.603	58.817	73.225	51.74	88.493	67.729	68.362
aldo-keto reductase family 1 member B1-like OS=Bombus vosnesenskii OX=207650 GN=LOC117240155 PE=4 SV=1	A0A6J3LBB3	36 kDa	46.316	83.518	63.718	72.235	70.454	74.22	68.657	61.622
inositol 2-dehydrogenase-like OS=Bombus vosnesenskii OX=207650 GN=LOC117242414 PE=3 SV=1	A0A6J3LI37	43 kDa	61.754	60.741	49.994	62.34	78.16	79.929	90.924	36.588
Fructose-bisphosphate aldolase OS=Bombus vosnesenskii OX=207650 GN=LOC117238722 PE=3 SV=1	A0A6J3L3C4	39 kDa	53.52	73.756	54.896	64.319	49.538	77.074	62.162	65.473
serine protease inhibitor 88Ea-like isoform X2 OS=Bombus vosnesenskii OX=207650 GN=LOC117233673 PE=3 SV=1	A0A6J3KBG4 (+1)	50 kDa	96.748	54.233	78.422	47.497	55.042	36.158	46.39	70.287
Pyruvate kinase OS=Bombus vosnesenskii OX=207650 GN=LOC117235801 PE=3 SV=1	A0A6J3KQ88	59 kDa	67.93	56.402	52.935	41.56	79.261	64.705	52.884	49.105
3-ketoacyl-CoA thiolase, mitochondrial OS=Bombus vosnesenskii OX=207650 GN=LOC117234486 PE=3 SV=1	A0A6J3KFI3	42 kDa	49.403	58.571	49.014	51.455	74.858	84.687	54.74	32.737
chitinase-like protein Idgf4 isoform X2 OS=Bombus	A0A6J3LI89	48 kDa	48.374	55.317	48.034	50.466	49.538	66.608	65.873	58.733

vosnesenskii OX=207650 GN=LOC117242559 PE=3 SV=1										
acetyl-CoA acetyltransferase, cytosolic OS=Bombus vosnesenskii OX=207650 GN=LOC117236714 PE=3 SV=1	A0A6J3KVA4	41 kDa	21.614	45.555	55.876	50.466	61.647	78.978	51.029	42.365
heat shock 70 kDa protein cognate 5 OS=Bombus vosnesenskii OX=207650 GN=LOC117238386 PE=3 SV=1	A0A6J3L1N7	75 kDa	66.9	41.217	43.132	39.581	55.042	64.705	46.39	46.216
heat shock 70 kDa protein cognate 4 OS=Bombus vosnesenskii OX=207650 GN=LOC117238264 PE=3 SV=1	A0A6J3L4B2	71 kDa	65.871	49.894	45.093	40.57	60.547	62.801	41.751	36.588
alpha-L-fucosidase OS=Bombus vosnesenskii OX=207650 GN=LOC117242104 PE=3 SV=1	A0A6J3LKH1	56 kDa	53.52	53.148	40.191	46.507	63.849	43.771	53.812	49.105
Transgelin OS=Bombus vosnesenskii OX=207650 GN=LOC117242319 PE=3 SV=1	A0A6J3LHN2	20 kDa	32.936	39.047	48.034	34.633	55.042	57.092	50.101	63.547
venom serine protease 34-like OS=Bombus vosnesenskii OX=207650 GN=LOC117238058 PE=4 SV=1	A0A6J3L320	45 kDa	38.082	48.809	55.876	62.34	52.841	31.401	29.689	55.845
ubiquitin-40S ribosomal protein S27a OS=Bombus vosnesenskii OX=207650 GN=LOC117233173 PE=3 SV=1	A0A6J3K9D4	18 kDa	54.55	48.809	35.29	39.581	55.042	44.722	39.895	42.365
transketolase-like protein 2 isoform X2 OS=Bombus vosnesenskii OX=207650 GN=LOC117237681 PE=4 SV=1	A0A6J3KWQ1	67 kDa	65.871	45.555	49.994	40.57	48.437	52.335	40.823	23.108
Phosphotransferase OS=Bombus vosnesenskii OX=207650 GN=LOC117230814 PE=3 SV=1	A0A6J3JUQ3 (+4)	51 kDa	55.579	37.963	42.152	35.623	58.345	40.916	41.751	39.476
peroxiredoxin 1-like OS=Bombus vosnesenskii OX=207650	A0A6J3L2X1	22 kDa	43.228	41.217	51.955	33.644	52.841	44.722	38.967	41.402

GN=LOC117238633 PE=3 SV=1										
sp|K1C10_HUMAN|	sp|K1C10_HUMAN|	60 kDa	61.754	44.471	52.935	29.686	40.731	51.383	35.256	26.959
slit homolog 3 protein-like OS=Bombus vosnesenskii OX=207650 GN=LOC117235722 PE=4 SV=1	A0A6J3KPW0	31 kDa	19.556	65.079	54.896	73.225	68.253	23.788	12.989	21.182
Transaldolase OS=Bombus vosnesenskii OX=207650 GN=LOC117234257 PE=3 SV=1	A0A6J3KGW6	37 kDa	28.819	43.386	34.31	39.581	35.227	54.238	44.534	38.514
ATP-citrate synthase OS=Bombus vosnesenskii OX=207650 GN=LOC117243094 PE=3 SV=1	A0A6J3LQA8	120 kDa	7.2047	36.878	50.974	41.56	48.437	62.801	32.473	32.737
tenascin-like OS=Bombus vosnesenskii OX=207650 GN=LOC117240225 PE=4 SV=1	A0A6J3LBP4	69 kDa	44.257	43.386	28.428	42.549	45.135	44.722	38.967	24.071
Peptidyl-prolyl cis-trans isomerase OS=Bombus vosnesenskii OX=207650 GN=LOC117235564 PE=3 SV=1	A0A6J3KKY3	23 kDa	37.053	41.217	40.191	39.581	33.025	39.013	38.04	41.402
Malic enzyme OS=Bombus vosnesenskii OX=207650 GN=LOC117230634 PE=3 SV=1	A0A6J3JUX4 (+2)	71 kDa	18.526	43.386	38.231	31.665	56.143	52.335	34.328	35.625
Catalase OS=Bombus vosnesenskii OX=207650 GN=LOC117233114 PE=3 SV=1	A0A6J3K7A5 (+1)	67 kDa	43.228	39.047	42.152	30.675	34.126	40.916	38.967	27.922
Glycerol-3-phosphate dehydrogenase [NAD(+)] OS=Bombus vosnesenskii OX=207650 GN=LOC117231866 PE=3 SV=1	A0A6J3K2X5	41 kDa	43.228	33.624	31.369	43.539	47.336	44.722	38.04	19.257
probable enoyl-CoA hydratase, mitochondrial OS=Bombus vosnesenskii OX=207650 GN=LOC117242339 PE=3 SV=1	A0A6J3LJE0	32 kDa	43.228	42.301	32.349	30.675	44.034	43.771	34.328	24.071
Triosephosphate isomerase OS=Bombus vosnesenskii OX=207650	A0A6J3LP61	27 kDa	45.286	32.54	36.27	27.707	49.538	40.916	35.256	28.885

GN=LOC117242845 PE=3 SV=1										
lysozyme OS=Bombus vosnesenskii OX=207650 GN=LOC117231586 PE=3 SV=1	A0A6J3K197	18 kDa	34.994	23.862	43.132	27.707	39.631	31.401	50.101	41.402
D-altritol 5-dehydrogenase-like isoform X1 OS=Bombus vosnesenskii OX=207650 GN=LOC117241762 PE=4 SV=1	A0A6J3LGD9	39 kDa	40.14	43.386	39.211	32.654	42.933	28.546	33.401	30.811
putative serine protease K12H4.7 OS=Bombus vosnesenskii OX=207650 GN=LOC117237063 PE=3 SV=1	A0A6J3KU14	56 kDa	7.2047	48.809	38.231	41.56	24.219	34.255	42.679	43.328
general odorant-binding protein 56d-like OS=Bombus vosnesenskii OX=207650 GN=LOC117239448 PE=4 SV=1	A0A6J3L6L1	15 kDa	28.819	35.794	35.29	29.686	55.042	37.11	41.751	23.108
alpha-glucosidase OS=Bombus vosnesenskii OX=207650 GN=LOC117235298 PE=4 SV=1	A0A6J3KMJ2	65 kDa	34.994	42.301	41.172	72.235	25.32	14.273	8.3501	43.328
Isocitrate dehydrogenase [NADP] OS=Bombus vosnesenskii OX=207650 GN=LOC117231466 PE=3 SV=1	A0A6J3K047 (+1)	46 kDa	46.316	32.54	29.408	31.665	31.925	40.916	34.328	29.848
venom protease-like isoform X1 OS=Bombus vosnesenskii OX=207650 GN=LOC117237097 PE=4 SV=1	A0A6J3KU65 (+1)	32 kDa	5.1462	0	50.974	45.518	0	79.929	80.718	0
Malate dehydrogenase OS=Bombus vosnesenskii OX=207650 GN=LOC117232774 PE=3 SV=1	A0A6J3K5D0	36 kDa	45.286	39.047	31.369	26.717	39.631	32.352	31.545	27.922
plancitoxin-1 isoform X1 OS=Bombus vosnesenskii OX=207650 GN=LOC117233141 PE=3 SV=1	A0A6J3KAL1 (+1)	46 kDa	30.877	30.37	30.389	36.612	39.631	32.352	35.256	33.699
enoyl-CoA hydratase OS=Bombus vosnesenskii OX=207650 GN=LOC117232959 PE=3 SV=1	A0A6J3K7P0 (+1)	80 kDa	34.994	31.455	29.408	32.654	34.126	39.965	34.328	25.034

ornithine decarboxylase 2-like OS=Bombus vosnesenskii OX=207650 GN=LOC117238735 PE=3 SV=1	A0A6J3L3E1	47 kDa	19.556	27.116	42.152	33.644	26.42	38.061	31.545	39.476
chymotrypsin inhibitor-like OS=Bombus vosnesenskii OX=207650 GN=LOC117240283 PE=4 SV=1	A0A6J3L993	8 kDa	36.023	32.54	30.389	32.654	34.126	35.207	30.617	29.848
antichymotrypsin-2-like isoform X2 OS=Bombus vosnesenskii OX=207650 GN=LOC117231437 PE=3 SV=1	A0A6J3JY50	45 kDa	23.672	26.032	36.27	30.675	37.429	34.255	41.751	21.182
sp|K22E_HUMAN|	sp|K22E_HUMAN|	66 kDa	36.023	37.963	37.251	21.769	25.32	40.916	30.617	20.22
LOW QUALITY PROTEIN: endoplasmic reticulum chaperone BiP-like OS=Bombus vosnesenskii OX=207650 GN=LOC117240895 PE=3 SV=1	A0A6J3LCD0	73 kDa	24.702	27.116	32.349	27.707	37.429	35.207	32.473	30.811
Citrate synthase OS=Bombus vosnesenskii OX=207650 GN=LOC117241831 PE=3 SV=1	A0A6J3LGS6	51 kDa	1.0292	45.555	34.31	30.675	27.521	36.158	35.256	28.885
Phosphoglycerate mutase OS=Bombus vosnesenskii OX=207650 GN=LOC117230691 PE=3 SV=1	A0A6J3JVM9	29 kDa	27.789	27.116	33.329	31.665	35.227	31.401	28.762	24.071
Glutathione peroxidase OS=Bombus vosnesenskii OX=207650 GN=LOC117238361 PE=3 SV=1	A0A6J3L1F5 (+1)	22 kDa	25.731	29.286	31.369	24.738	29.723	29.498	27.834	30.811
hydroxyacid-oxoacid transhydrogenase OS=Bombus vosnesenskii OX=207650 GN=LOC117243594 PE=3 SV=1	A0A6J3LNR3	51 kDa	11.322	40.132	27.448	25.728	39.631	35.207	31.545	21.182
Dipeptidase OS=Bombus vosnesenskii OX=207650 GN=LOC117238734 PE=3 SV=1	A0A6J3L5M3 (+1)	51 kDa	31.906	24.947	28.428	21.769	31.925	30.449	29.689	26.959
cytoplasmic aconitate hydratase-like OS=Bombus vosnesenskii OX=207650	A0A6J3JV87	99 kDa	22.643	31.455	26.467	24.738	30.824	34.255	31.545	23.108

GN=LOC117230685 PE=3 SV=1										
xanthine dehydrogenase/oxidase-like isoform X1 OS=Bombus vosnesenskii OX=207650 GN=LOC117242522 PE=3 SV=1	A0A6J3LHY8 (+1)	141 kDa	54.55	27.116	20.586	26.717	33.025	20.934	24.123	16.368
Calreticulin OS=Bombus vosnesenskii OX=207650 GN=LOC117242401 PE=3 SV=1	A0A6J3LJP3	47 kDa	9.2631	40.132	37.251	33.644	18.714	28.546	28.762	20.22
Aspartate aminotransferase OS=Bombus vosnesenskii OX=207650 GN=LOC117230654 PE=3 SV=1	A0A6J3JTX0	47 kDa	37.053	27.116	26.467	19.79	31.925	26.643	25.05	21.182
protein artichoke-like isoform X2 OS=Bombus vosnesenskii OX=207650 GN=LOC117239542 PE=4 SV=1	A0A6J3L645 (+2)	79 kDa	32.936	26.032	27.448	19.79	33.025	25.691	28.762	22.145
phosphopyruvate hydratase OS=Bombus vosnesenskii OX=207650 GN=LOC117233542 PE=3 SV=1	A0A6J3KBG5	47 kDa	47.345	28.201	22.546	20.78	34.126	22.837	25.978	13.48
uncharacterized protein LOC117239136 OS=Bombus vosnesenskii OX=207650 GN=LOC117239136 PE=4 SV=1	A0A6J3L525	37 kDa	15.439	29.286	17.645	20.78	33.025	26.643	36.184	17.331
glutamate dehydrogenase [NAD(P)(+)] OS=Bombus vosnesenskii OX=207650 GN=LOC117231037 PE=3 SV=1	A0A6J3JXC8	62 kDa	1.0292	34.709	25.487	28.696	19.815	40.916	24.123	30.811
peroxisomal multifunctional enzyme type 2 OS=Bombus vosnesenskii OX=207650 GN=LOC117231189 PE=4 SV=1	A0A6J3JWN4	80 kDa	18.526	16.27	21.566	21.769	28.622	51.383	23.195	17.331
Nucleoside diphosphate kinase OS=Bombus vosnesenskii OX=207650 GN=LOC117236192 PE=3 SV=1	A0A6J3KNU4	19 kDa	33.965	20.608	29.408	18.801	26.42	22.837	23.195	25.034
Aminopeptidase OS=Bombus vosnesenskii OX=207650	A0A6J3KHZ5	99 kDa	13.38	26.032	33.329	26.717	40.731	32.352	18.556	10.591

GN=LOC117235017 PE=3 SV=1										
sp|K1C9_HUMAN|	sp|K1C9_HUMAN|	62 kDa	31.906	30.37	17.645	19.79	19.815	38.061	27.834	7.7027
cytochrome P450 315a1, mitochondrial OS=Bombus vosnesenskii OX=207650 GN=LOC117239595 PE=3 SV=1	A0A6J3L998	61 kDa	2.0585	10.847	53.915	26.717	2.2017	46.625	38.967	7.7027
antichymotrypsin-2-like OS=Bombus vosnesenskii OX=207650 GN=LOC117231439 PE=3 SV=1	A0A6J3K0H4	44 kDa	27.789	21.693	23.527	17.811	29.723	19.982	30.617	22.145
uncharacterized protein LOC117234144 OS=Bombus vosnesenskii OX=207650 GN=LOC117234144 PE=4 SV=1	A0A6J3KF51	27 kDa	26.76	20.608	18.625	31.665	12.109	32.352	15.772	30.811
alpha-glucosidase OS=Bombus vosnesenskii OX=207650 GN=LOC117239768 PE=4 SV=1	A0A6J3L783	66 kDa	17.497	28.201	30.389	47.497	17.614	12.37	4.639	32.737
uncharacterized protein LOC117232001 OS=Bombus vosnesenskii OX=207650 GN=LOC117232001 PE=4 SV=1	A0A6J3K381	12 kDa	37.053	19.524	14.704	25.728	22.017	25.691	19.484	20.22
Protein disulfide-isomerase OS=Bombus vosnesenskii OX=207650 GN=LOC117239181 PE=3 SV=1	A0A6J3L956	56 kDa	31.906	21.693	17.645	27.707	18.714	29.498	15.772	18.294
Carboxypeptidase OS=Bombus vosnesenskii OX=207650 GN=LOC117231762 PE=3 SV=1	A0A6J3JZZ8	54 kDa	8.2339	23.862	26.467	22.759	27.521	27.595	22.267	18.294
Multifunctional fusion protein OS=Bombus vosnesenskii OX=207650 GN=LOC117232948 PE=3 SV=1	A0A6J3K829 (+1)	63 kDa	18.526	18.439	20.586	15.832	28.622	25.691	24.123	17.331
thioredoxin reductase 1, mitochondrial isoform X2 OS=Bombus vosnesenskii OX=207650 GN=LOC117234026 PE=3 SV=1	A0A6J3KFK9 (+2)	58 kDa	29.848	18.439	22.546	19.79	20.916	19.982	15.772	27.922
N-acyl-aliphatic-L-amino acid amidohydrolase OS=Bombus	A0A6J3KTI8	46 kDa	29.848	18.439	23.527	18.801	19.815	15.225	23.195	19.257

vosnesenskii OX=207650 GN=LOC117236511 PE=3 SV=1										
Superoxide dismutase [Cu-Zn] OS=Bombus vosnesenskii OX=207650 GN=LOC117232686 PE=3 SV=1	A0A6J3K7G9	18 kDa	24.702	23.862	23.527	18.801	23.118	19.982	18.556	20.22
Prolyl endopeptidase OS=Bombus vosnesenskii OX=207650 GN=LOC117239543 PE=3 SV=1	A0A6J3L704	86 kDa	23.672	20.608	24.507	16.822	27.521	16.176	16.7	25.997
peptidylglycine monooxygenase OS=Bombus vosnesenskii OX=207650 GN=LOC117231991 PE=4 SV=1	A0A6J3K367	37 kDa	16.468	20.608	23.527	22.759	13.21	23.788	22.267	22.145
alpha-glucosidase OS=Bombus vosnesenskii OX=207650 GN=LOC117233420 PE=4 SV=1	A0A6J3K922	69 kDa	22.643	17.354	25.487	29.686	25.32	13.322	4.639	28.885
actin-5C OS=Bombus vosnesenskii OX=207650 GN=LOC117242144 PE=3 SV=1	A0A6J3LGG6	42 kDa	11.322	22.778	17.645	27.707	17.614	28.546	16.7	23.108
uncharacterized protein LOC117235293 OS=Bombus vosnesenskii OX=207650 GN=LOC117235293 PE=3 SV=1	A0A6J3KJC7	128 kDa	48.374	24.947	15.684	19.79	16.513	9.5154	16.7	5.777
glycine--tRNA ligase OS=Bombus vosnesenskii OX=207650 GN=LOC117240358 PE=3 SV=1	A0A6J3L9H6	55 kDa	23.672	17.354	18.625	19.79	19.815	18.079	21.339	15.405
lambda-crystallin homolog isoform X2 OS=Bombus vosnesenskii OX=207650 GN=LOC117232137 PE=3 SV=1	A0A6J3K4R3	36 kDa	10.292	24.947	21.566	18.801	30.824	24.74	22.267	1.9257
Dipeptidyl peptidase 3 OS=Bombus vosnesenskii OX=207650 GN=LOC117231034 PE=3 SV=1	A0A6J3JVW9	90 kDa	15.439	16.27	29.408	14.843	25.32	19.031	22.267	11.554
palmitoyl-protein thioesterase 1 OS=Bombus vosnesenskii OX=207650	A0A6J3LA43	35 kDa	25.731	15.185	18.625	13.853	23.118	19.031	11.134	26.959

GN=LOC117239379 PE=3 SV=1										
hydroxysteroid dehydrogenase-like protein 2 OS=Bombus vosnesenskii OX=207650 GN=LOC117232682 PE=4 SV=1	A0A6J3K6L0	46 kDa	22.643	18.439	25.487	19.79	13.21	22.837	12.061	17.331
Superoxide dismutase OS=Bombus vosnesenskii OX=207650 GN=LOC117233036 PE=3 SV=1	A0A6J3K822	25 kDa	26.76	22.778	12.744	13.853	26.42	17.128	18.556	10.591
Malate dehydrogenase OS=Bombus vosnesenskii OX=207650 GN=LOC117232345 PE=3 SV=1	A0A6J3K3A1	36 kDa	9.2631	15.185	13.724	15.832	12.109	36.158	19.484	17.331
cytochrome c OS=Bombus vosnesenskii OX=207650 GN=LOC117239742 PE=3 SV=1	A0A6J3L760	12 kDa	22.643	13.016	17.645	13.853	24.219	15.225	23.195	15.405
defensin-1 OS=Bombus vosnesenskii OX=207650 GN=LOC117233325 PE=4 SV=1	A0A6J3K9M3	18 kDa	32.936	31.455	24.507	16.822	9.9076	8.5638	10.206	8.6656
Adenylosuccinate synthetase OS=Bombus vosnesenskii OX=207650 GN=LOC117243394 PE=3 SV=1	A0A6J3LMV0	49 kDa	16.468	14.1	16.665	15.832	23.118	21.885	18.556	15.405
very long-chain specific acyl-CoA dehydrogenase, mitochondrial OS=Bombus vosnesenskii OX=207650 GN=LOC117238208 PE=3 SV=1	A0A6J3L0E8	69 kDa	2.0585	22.778	19.606	17.811	20.916	37.11	20.411	0.96284
Thioredoxin OS=Bombus vosnesenskii OX=207650 GN=LOC117234344 PE=3 SV=1	A0A6J3KE25	12 kDa	13.38	14.1	13.724	14.843	31.925	25.691	14.845	13.48
antichymotrypsin-2-like OS=Bombus vosnesenskii OX=207650 GN=LOC117231436 PE=3 SV=1	A0A6J3K005	45 kDa	26.76	10.847	15.684	26.717	8.8068	11.418	16.7	22.145
Acyl-coenzyme A oxidase OS=Bombus vosnesenskii OX=207650 GN=LOC117230768 PE=3 SV=1	A0A6J3JUC6	77 kDa	4.117	17.354	19.606	9.8952	16.513	50.431	12.989	8.6656

aldehyde dehydrogenase (NAD(+)) OS=Bombus vosnesenskii OX=207650 GN=LOC117232657 PE=3 SV=1	A0A6J3K4P4	57 kDa	22.643	16.27	15.684	14.843	26.42	14.273	18.556	10.591
Rab GDP dissociation inhibitor OS=Bombus vosnesenskii OX=207650 GN=LOC117236932 PE=3 SV=1	A0A6J3KT72	50 kDa	15.439	11.931	19.606	21.769	20.916	15.225	16.7	14.443
4a-hydroxytetrahydrobiopterin dehydratase OS=Bombus vosnesenskii OX=207650 GN=LOC117242770 PE=3 SV=1	A0A6J3LJ85	16 kDa	20.585	16.27	13.724	13.853	19.815	18.079	18.556	17.331
Mesencephalic astrocyte-derived neurotrophic factor homolog OS=Bombus vosnesenskii OX=207650 GN=LOC117234342 PE=3 SV=1	A0A6J3KDT9	20 kDa	14.409	14.1	16.665	18.801	11.008	17.128	15.772	22.145
Probable methylmalonate-semialdehyde dehydrogenase [acylating], mitochondrial OS=Bombus vosnesenskii OX=207650 GN=LOC117233535 PE=3 SV=1	A0A6J3K9M5	56 kDa	18.526	21.693	11.763	16.822	19.815	19.031	14.845	10.591
Carboxypeptidase Q OS=Bombus vosnesenskii OX=207650 GN=LOC117230722 PE=4 SV=1	A0A6J3JU92 (+1)	64 kDa	18.526	15.185	14.704	11.874	12.109	6.6608	14.845	33.699
LOW QUALITY PROTEIN: protein Red-like OS=Bombus vosnesenskii OX=207650 GN=LOC117242386 PE=3 SV=1	A0A6J3LH70	102 kDa	16.468	9.7619	13.724	11.874	12.109	15.225	21.339	19.257
lysosomal aspartic protease OS=Bombus vosnesenskii OX=207650 GN=LOC117242419 PE=3 SV=1	A0A6J3LHR5	42 kDa	1.0292	18.439	2.9408	23.749	0	28.546	17.628	29.848
gamma-interferon-inducible lysosomal thiol reductase-like OS=Bombus vosnesenskii OX=207650 GN=LOC117234916 PE=3 SV=1	A0A6J3KGZ4	25 kDa	10.292	9.7619	14.704	15.832	16.513	13.322	22.267	19.257

translation elongation factor 2 OS=Bombus vosnesenskii OX=207650 GN=LOC117236689 PE=3 SV=1	A0A6J3KV39	95 kDa	10.292	18.439	13.724	17.811	13.21	25.691	12.989	10.591
aldehyde dehydrogenase X, mitochondrial isoform X2 OS=Bombus vosnesenskii OX=207650 GN=LOC117242272 PE=3 SV=1	A0A6J3LGM0 (+1)	53 kDa	14.409	18.439	9.8028	15.832	17.614	14.273	20.411	12.517
Hydroxymethylglutaryl-CoA synthase OS=Bombus vosnesenskii OX=207650 GN=LOC117232655 PE=3 SV=1	A0A6J3K4N3	51 kDa	0	10.847	17.645	12.864	18.714	32.352	14.845	13.48
chymotrypsin-1-like OS=Bombus vosnesenskii OX=207650 GN=LOC117231855 PE=4 SV=1	A0A6J3K0H0	28 kDa	15.439	18.439	20.586	16.822	9.9076	7.6123	1.8556	31.774
alanine transaminase OS=Bombus vosnesenskii OX=207650 GN=LOC117242549 PE=3 SV=1	A0A6J3LIK0	61 kDa	7.2047	18.439	15.684	13.853	13.21	19.031	17.628	11.554
phenoloxidase-activating factor 2-like isoform X1 OS=Bombus vosnesenskii OX=207650 GN=LOC117235288 PE=4 SV=1	A0A6J3KIR2 (+1)	47 kDa	8.2339	19.524	18.625	19.79	16.513	6.6608	16.7	13.48
folate gamma-glutamyl hydrolase OS=Bombus vosnesenskii OX=207650 GN=LOC117231476 PE=3 SV=1	A0A6J3K067	37 kDa	16.468	15.185	9.8028	14.843	16.513	13.322	17.628	13.48
Protein disulfide-isomerase OS=Bombus vosnesenskii OX=207650 GN=LOC117237128 PE=3 SV=1	A0A6J3KUB9	56 kDa	3.0877	18.439	12.744	21.769	13.21	20.934	18.556	11.554
ribonuclease Oy OS=Bombus vosnesenskii OX=207650 GN=LOC117235716 PE=3 SV=1	A0A6J3KPV6	32 kDa	7.2047	16.27	14.704	16.822	13.21	19.031	16.7	15.405
actin, muscle OS=Bombus vosnesenskii OX=207650 GN=LOC117235961 PE=3 SV=1	A0A6J3KMX3	42 kDa	0	16.27	9.8028	24.738	0	23.788	10.206	16.368

presequence protease, mitochondrial isoform X2 OS=Bombus vosnesenskii OX=207650 GN=LOC117231361 PE=4 SV=1	A0A6J3JZN8 (+1)	119 kDa	10.292	13.016	15.684	11.874	16.513	21.885	16.7	10.591
alpha-glucosidase OS=Bombus vosnesenskii OX=207650 GN=LOC117233421 PE=4 SV=1	A0A6J3KC70	66 kDa	19.556	6.5079	17.645	10.885	9.9076	11.418	23.195	16.368
Dihydrolipoyl dehydrogenase OS=Bombus vosnesenskii OX=207650 GN=LOC117241790 PE=3 SV=1	A0A6J3LGI2	54 kDa	11.322	19.524	17.645	12.864	25.32	17.128	9.2779	3.8514
protein dj-1beta-like OS=Bombus vosnesenskii OX=207650 GN=LOC117242227 PE=4 SV=1	A0A6J3LGV4	23 kDa	7.2047	16.27	18.625	13.853	11.008	14.273	17.628	16.368
neutral alpha-glucosidase AB isoform X2 OS=Bombus vosnesenskii OX=207650 GN=LOC117239666 PE=3 SV=1	A0A6J3L6Q2 (+3)	107 kDa	15.439	16.27	9.8028	9.8952	20.916	15.225	12.061	15.405
trifunctional enzyme subunit beta, mitochondrial OS=Bombus vosnesenskii OX=207650 GN=LOC117231974 PE=3 SV=1	A0A6J3K330	51 kDa	13.38	11.931	15.684	9.8952	22.017	19.982	12.989	7.7027
Beta-hexosaminidase OS=Bombus vosnesenskii OX=207650 GN=LOC117230346 PE=3 SV=1	A0A6J3JTD9	62 kDa	11.322	3.254	6.8619	16.822	29.723	6.6608	25.978	6.7399
spermine oxidase-like OS=Bombus vosnesenskii OX=207650 GN=LOC117230418 PE=4 SV=1	A0A6J3JU51	56 kDa	9.2631	11.931	14.704	12.864	17.614	16.176	17.628	9.6284
LOW QUALITY PROTEIN: fumarylacetoacetate hydrolase domain-containing protein 2A OS=Bombus vosnesenskii OX=207650 GN=LOC117240147 PE=3 SV=1	A0A6J3L8H4	38 kDa	13.38	13.016	15.684	9.8952	14.311	12.37	15.772	12.517
protein D2-like isoform X1 OS=Bombus vosnesenskii	A0A6J3JVN2 (+1)	23 kDa	15.439	11.931	10.783	8.9057	18.714	12.37	16.7	12.517

OX=207650 GN=LOC117230745 PE=4 SV=1										
Peptidyl-prolyl cis-trans isomerase OS=Bombus vosnesenskii OX=207650 GN=LOC117235053 PE=3 SV=1	A0A6J3KL98	23 kDa	5.1462	13.016	12.744	15.832	8.8068	20.934	12.989	16.368
N-acetylneuraminate lyase-like isoform X1 OS=Bombus vosnesenskii OX=207650 GN=LOC117235775 PE=3 SV=1	A0A6J3KLW3	33 kDa	19.556	8.6772	9.8028	12.864	15.412	13.322	15.772	6.7399
Acyl-coenzyme A oxidase OS=Bombus vosnesenskii OX=207650 GN=LOC117230767 PE=3 SV=1	A0A6J3JVS6	76 kDa	0	0	0	0	0	32.352	0	0
Aldose 1-epimerase OS=Bombus vosnesenskii OX=207650 GN=LOC117239793 PE=3 SV=1	A0A6J3L7B7 (+2)	41 kDa	14.409	13.016	13.724	13.853	16.513	7.6123	13.917	8.6656
antichymotrypsin-2-like OS=Bombus vosnesenskii OX=207650 GN=LOC117231371 PE=3 SV=1	A0A6J3JZC3	59 kDa	14.409	7.5926	12.744	10.885	17.614	12.37	15.772	9.6284
kynurenine--oxoglutarate transaminase 3 OS=Bombus vosnesenskii OX=207650 GN=LOC117237489 PE=4 SV=1	A0A6J3KWC1	51 kDa	0	14.1	13.724	14.843	3.3025	22.837	18.556	13.48
uncharacterized protein LOC117230889 OS=Bombus vosnesenskii OX=207650 GN=LOC117230889 PE=4 SV=1	A0A6J3JX64	53 kDa	11.322	7.5926	13.724	13.853	9.9076	19.982	11.134	12.517
Aspartate aminotransferase OS=Bombus vosnesenskii OX=207650 GN=LOC117237467 PE=3 SV=1	A0A6J3KYQ6	48 kDa	3.0877	14.1	19.606	8.9057	18.714	15.225	14.845	3.8514
ester hydrolase C11orf54 homolog OS=Bombus vosnesenskii OX=207650 GN=LOC117238785 PE=4 SV=1	A0A6J3L5T9	35 kDa	18.526	16.27	8.8225	11.874	18.714	12.37	9.2779	2.8885
isopentenyl-diphosphate Delta-isomerase OS=Bombus	A0A6J3L543	28 kDa	1.0292	3.254	2.9408	7.9162	1.1008	42.819	18.556	16.368

vosnesenskii OX=207650 GN=LOC117239147 PE=3 SV=1										
BMP-binding endothelial regulator protein-like OS=Bombus vosnesenskii OX=207650 GN=LOC117239152 PE=4 SV=1	A0A6J3L556	35 kDa	1.0292	17.354	11.763	16.822	8.8068	18.079	10.206	7.7027
Glycerol-3-phosphate dehydrogenase OS=Bombus vosnesenskii OX=207650 GN=LOC117236450 PE=3 SV=1	A0A6J3KPT4 (+2)	81 kDa	5.1462	9.7619	9.8028	9.8952	13.21	19.982	11.134	13.48
Annexin OS=Bombus vosnesenskii OX=207650 GN=LOC117239673 PE=3 SV=1	A0A6J3L7R4	36 kDa	9.2631	10.847	12.744	10.885	13.21	14.273	9.2779	13.48
NPC intracellular cholesterol transporter 2-like OS=Bombus vosnesenskii OX=207650 GN=LOC117237184 PE=3 SV=1	A0A6J3KYB3	16 kDa	10.292	6.5079	13.724	9.8952	16.513	14.273	3.7112	16.368
phosphoribosylformylglycinamidine synthase OS=Bombus vosnesenskii OX=207650 GN=LOC117241660 PE=3 SV=1	A0A6J3LI08	147 kDa	2.0585	15.185	10.783	13.853	13.21	14.273	13.917	5.777
aldo-keto reductase family 1 member B1-like OS=Bombus vosnesenskii OX=207650 GN=LOC117240154 PE=4 SV=1	A0A6J3L9M0	36 kDa	10.292	14.1	10.783	10.885	6.6051	14.273	9.2779	9.6284
NADH-cytochrome b5 reductase OS=Bombus vosnesenskii OX=207650 GN=LOC117233094 PE=3 SV=1	A0A6J3K764 (+2)	34 kDa	6.1754	16.27	10.783	8.9057	7.7059	10.467	9.2779	14.443
uncharacterized protein LOC117230545 OS=Bombus vosnesenskii OX=207650 GN=LOC117230545 PE=4 SV=1	A0A6J3JV84	49 kDa	9.2631	10.847	10.783	10.885	12.109	13.322	6.4946	9.6284
beta-mannosidase OS=Bombus vosnesenskii OX=207650 GN=LOC117239229 PE=3 SV=1	A0A6J3L9G7	184 kDa	18.526	6.5079	8.8225	9.8952	12.109	6.6608	12.061	7.7027
aldehyde dehydrogenase, mitochondrial OS=Bombus vosnesenskii OX=207650	A0A6J3L942	56 kDa	1.0292	7.5926	6.8619	10.885	5.5042	21.885	14.845	9.6284

GN=LOC117240084 PE=3 SV=1										
uncharacterized protein C05D11.1-like OS=Bombus vosnesenskii OX=207650 GN=LOC117242775 PE=4 SV=1	A0A6J3LJ97	117 kDa	11.322	13.016	8.8225	7.9162	5.5042	13.322	11.134	9.6284
Angiotensin-converting enzyme OS=Bombus vosnesenskii OX=207650 GN=LOC117235883 PE=3 SV=1	A0A6J3KQ34	74 kDa	13.38	8.6772	8.8225	8.9057	15.412	7.6123	8.3501	9.6284
xaa-Pro dipeptidase OS=Bombus vosnesenskii OX=207650 GN=LOC117231884 PE=4 SV=1	A0A6J3K308	55 kDa	16.468	9.7619	8.8225	5.9371	11.008	9.5154	9.2779	9.6284
Alpha-galactosidase OS=Bombus vosnesenskii OX=207650 GN=LOC117241661 PE=3 SV=1	A0A6J3LD28 (+1)	50 kDa	7.2047	8.6772	10.783	11.874	14.311	10.467	10.206	7.7027
S-formylglutathione hydrolase OS=Bombus vosnesenskii OX=207650 GN=LOC117231879 PE=3 SV=1	A0A6J3K2Z5	32 kDa	1.0292	13.016	10.783	9.8952	7.7059	11.418	13.917	9.6284
cytoglobin-2-like OS=Bombus vosnesenskii OX=207650 GN=LOC117235202 PE=3 SV=1	A0A6J3KIA7	19 kDa	3.0877	13.016	7.8422	8.9057	9.9076	9.5154	14.845	9.6284
lysosomal Pro-X carboxypeptidase OS=Bombus vosnesenskii OX=207650 GN=LOC117237864 PE=3 SV=1	A0A6J3L151	57 kDa	7.2047	11.931	7.8422	8.9057	6.6051	10.467	15.772	7.7027
ATP synthase subunit beta OS=Bombus vosnesenskii OX=207650 GN=LOC117243357 PE=3 SV=1	A0A6J3LME2	55 kDa	1.0292	15.185	16.665	7.9162	12.109	18.079	8.3501	0.96284
Prostaglandin reductase 1 OS=Bombus vosnesenskii OX=207650 GN=LOC117232106 PE=3 SV=1	A0A6J3K3R2 (+1)	32 kDa	5.1462	11.931	5.8817	9.8952	8.8068	11.418	12.061	9.6284
Malic enzyme OS=Bombus vosnesenskii OX=207650 GN=LOC117230727 PE=3 SV=1	A0A6J3JUA2 (+1)	70 kDa	3.0877	9.7619	10.783	8.9057	13.21	8.5638	11.134	7.7027

tetratricopeptide repeat protein 27-like isoform X2 OS=Bombus vosnesenskii OX=207650 GN=LOC117233658 PE=3 SV=1	A0A6J3KBE9 (+1)	111 kDa	10.292	4.3386	10.783	8.9057	13.21	13.322	10.206	5.777
protein FAM151B isoform X2 OS=Bombus vosnesenskii OX=207650 GN=LOC117238017 PE=3 SV=1	A0A6J3KZ70 (+1)	32 kDa	11.322	6.5079	6.8619	5.9371	13.21	10.467	9.2779	10.591
elongation factor 1-gamma OS=Bombus vosnesenskii OX=207650 GN=LOC117231394 PE=4 SV=1	A0A6J3JXX8	49 kDa	11.322	7.5926	10.783	6.9266	7.7059	15.225	8.3501	7.7027
trithorax group protein osa-like OS=Bombus vosnesenskii OX=207650 GN=LOC117242406 PE=4 SV=1	A0A6J3LLX3	79 kDa	4.117	9.7619	8.8225	9.8952	9.9076	7.6123	14.845	8.6656
Adenosylhomocysteinase OS=Bombus vosnesenskii OX=207650 GN=LOC117237425 PE=3 SV=1	A0A6J3KWE5	48 kDa	5.1462	9.7619	9.8028	2.9686	20.916	13.322	4.639	8.6656
N-sulphoglucosamine sulphohydrolase OS=Bombus vosnesenskii OX=207650 GN=LOC117231385 PE=3 SV=1	A0A6J3JXV0	59 kDa	9.2631	6.5079	13.724	8.9057	9.9076	5.7092	6.4946	10.591
uncharacterized protein LOC117232577 isoform X1 OS=Bombus vosnesenskii OX=207650 GN=LOC117232577 PE=4 SV=1	A0A6J3K5Z3 (+1)	52 kDa	10.292	8.6772	6.8619	6.9266	12.109	7.6123	8.3501	10.591
V-type proton ATPase subunit C OS=Bombus vosnesenskii OX=207650 GN=LOC117237209 PE=3 SV=1	A0A6J3KV38	45 kDa	2.0585	9.7619	6.8619	5.9371	12.109	12.37	8.3501	10.591
Putative hydroxypyruvate isomerase OS=Bombus vosnesenskii OX=207650 GN=LOC117238498 PE=3 SV=1	A0A6J3L4E6	30 kDa	6.1754	9.7619	15.684	5.9371	13.21	6.6608	6.4946	4.8142
neprilysin-2 isoform X2 OS=Bombus vosnesenskii OX=207650	A0A6J3LJI2 (+2)	91 kDa	13.38	11.931	4.9014	1.979	19.815	4.7577	5.5668	8.6656

GN=LOC117242822 PE=3 SV=1										
uncharacterized protein LOC117243154 isoform X2 OS=Bombus vosnesenskii OX=207650 GN=LOC117243154 PE=4 SV=1	A0A6J3LM77 (+1)	16 kDa	14.409	8.6772	5.8817	6.9266	7.7059	8.5638	9.2779	9.6284
farnesol dehydrogenase-like OS=Bombus vosnesenskii OX=207650 GN=LOC117238743 PE=3 SV=1	A0A6J3L2C4 (+2)	27 kDa	0	17.354	10.783	9.8952	7.7059	7.6123	10.206	5.777
Transgelin OS=Bombus vosnesenskii OX=207650 GN=LOC117240061 PE=3 SV=1	A0A6J3L916 (+1)	21 kDa	9.2631	8.6772	8.8225	7.9162	9.9076	10.467	7.4223	7.7027
glyoxalase domain-containing protein 4 OS=Bombus vosnesenskii OX=207650 GN=LOC117232230 PE=3 SV=1	A0A6J3K2N8	32 kDa	1.0292	5.4233	5.8817	9.8952	4.4034	18.079	12.061	10.591
Glucose-6-phosphate isomerase OS=Bombus vosnesenskii OX=207650 GN=LOC117234302 PE=3 SV=1	A0A6J3KDN0	63 kDa	6.1754	10.847	8.8225	4.9476	20.916	7.6123	4.639	3.8514
sodium/calcium exchanger regulatory protein 1 isoform X1 OS=Bombus vosnesenskii OX=207650 GN=LOC117236795 PE=3 SV=1	A0A6J3KUY4 (+1)	15 kDa	1.0292	8.6772	7.8422	13.853	6.6051	13.322	11.134	5.777
glutamate synthase (NADH) OS=Bombus vosnesenskii OX=207650 GN=LOC117235398 PE=3 SV=1	A0A6J3KJD9 (+1)	229 kDa	0	7.5926	3.9211	11.874	1.1008	19.031	5.5668	14.443
ecdysteroid-regulated 16 kDa protein OS=Bombus vosnesenskii OX=207650 GN=LOC117230938 PE=3 SV=1	A0A6J3JXE0	17 kDa	0	6.5079	2.9408	6.9266	2.2017	21.885	11.134	10.591
L-fuconate dehydratase OS=Bombus vosnesenskii OX=207650 GN=LOC117238197 PE=4 SV=1	A0A6J3KZI9	51 kDa	9.2631	8.6772	6.8619	6.9266	12.109	7.6123	8.3501	4.8142
Vinculin OS=Bombus vosnesenskii OX=207650	A0A6J3JTP9 (+18)	118 kDa	12.351	5.4233	10.783	3.9581	7.7059	11.418	5.5668	7.7027

GN=LOC117230649 PE=3 SV=1										
selenoprotein F OS=Bombus vosnesenskii OX=207650 GN=LOC117233169 PE=3 SV=1	A0A6J3K7M7	18 kDa	1.0292	6.5079	4.9014	11.874	3.3025	11.418	9.2779	18.294
Xylulose kinase OS=Bombus vosnesenskii OX=207650 GN=LOC117230886 PE=3 SV=1	A0A6J3JX60	59 kDa	1.0292	10.847	5.8817	5.9371	9.9076	10.467	12.989	6.7399
uncharacterized protein LOC117231108 OS=Bombus vosnesenskii OX=207650 GN=LOC117231108 PE=3 SV=1	A0A6J3JXU1	92 kDa	7.2047	11.931	8.8225	6.9266	13.21	5.7092	6.4946	3.8514
RNA-binding protein lark isoform X4 OS=Bombus vosnesenskii OX=207650 GN=LOC117241627 PE=4 SV=1	A0A6J3LCX5 (+6)	38 kDa	8.2339	5.4233	9.8028	5.9371	9.9076	10.467	6.4946	8.6656
Protein quiver OS=Bombus vosnesenskii OX=207650 GN=LOC117243557 PE=3 SV=1	A0A6J3LPG0	17 kDa	11.322	8.6772	9.8028	2.9686	9.9076	6.6608	6.4946	6.7399
Na(+)/H(+) exchange regulatory cofactor NHE-RF1 OS=Bombus vosnesenskii OX=207650 GN=LOC117237691 PE=4 SV=1	A0A6J3L077	28 kDa	13.38	6.5079	6.8619	5.9371	9.9076	3.8061	5.5668	9.6284
Eukaryotic translation initiation factor 5A OS=Bombus vosnesenskii OX=207650 GN=LOC117232474 PE=3 SV=1	A0A6J3K6A2	18 kDa	1.0292	7.5926	5.8817	9.8952	4.4034	11.418	6.4946	12.517
inorganic diphosphatase OS=Bombus vosnesenskii OX=207650 GN=LOC117240383 PE=3 SV=1	A0A6J3LAT6	38 kDa	15.439	6.5079	7.8422	3.9581	12.109	5.7092	4.639	3.8514
carnitine O-palmitoyltransferase 2, mitochondrial OS=Bombus vosnesenskii OX=207650 GN=LOC117237850 PE=3 SV=1	A0A6J3KYT6	76 kDa	1.0292	6.5079	6.8619	5.9371	8.8068	8.5638	10.206	7.7027
4-hydroxyphenylpyruvate dioxygenase OS=Bombus vosnesenskii OX=207650 GN=LOC117239655 PE=3 SV=1	A0A6J3LBH6	44 kDa	0	7.5926	9.8028	6.9266	6.6051	10.467	7.4223	10.591

Calponin OS=Bombus vosnesenskii OX=207650 GN=LOC117240277 PE=3 SV=1	A0A6J3LAE3	19 kDa	1.0292	7.5926	4.9014	7.9162	0	12.37	9.2779	15.405
4-coumarate--CoA ligase 1-like OS=Bombus vosnesenskii OX=207650 GN=LOC117231690 PE=3 SV=1	A0A6J3K1U4	65 kDa	5.1462	7.5926	5.8817	9.8952	9.9076	5.7092	7.4223	2.8885
tr|A0A6J3L998|A0A6J3L998_9HYME-DECOY	tr|A0A6J3L998|A0A6J3L998_9HYME-DECOY	?	0	1.0847	10.783	10.885	0	12.37	14.845	0.96284
Alpha-mannosidase OS=Bombus vosnesenskii OX=207650 GN=LOC117235663 PE=3 SV=1	A0A6J3KNS1 (+1)	114 kDa	5.1462	11.931	9.8028	5.9371	6.6051	0.95154	4.639	9.6284
E1 ubiquitin-activating enzyme OS=Bombus vosnesenskii OX=207650 GN=LOC117235496 PE=3 SV=1	A0A6J3KKI9	117 kDa	1.0292	5.4233	5.8817	6.9266	5.5042	11.418	12.061	7.7027
Acetyl-CoA hydrolase OS=Bombus vosnesenskii OX=207650 GN=LOC117238640 PE=3 SV=1	A0A6J3L1V1 (+1)	53 kDa	0	8.6772	11.763	7.9162	8.8068	5.7092	3.7112	9.6284
LOW QUALITY PROTEIN: titin OS=Bombus vosnesenskii OX=207650 GN=LOC117242036 PE=3 SV=1	A0A6J3LFW1	2183 kDa	17.497	3.254	2.9408	0.98952	24.219	4.7577	0.92779	2.8885
Inositol oxygenase OS=Bombus vosnesenskii OX=207650 GN=LOC117237723 PE=3 SV=1	A0A6J3KY58	35 kDa	2.0585	10.847	5.8817	6.9266	4.4034	7.6123	7.4223	7.7027
lysozyme OS=Bombus vosnesenskii OX=207650 GN=LOC117235558 PE=4 SV=1	A0A6J3KP02	17 kDa	6.1754	4.3386	2.9408	11.874	5.5042	6.6608	7.4223	7.7027
twitchin OS=Bombus vosnesenskii OX=207650 GN=LOC117237692 PE=3 SV=1	A0A6J3KY12	1038 kDa	12.351	5.4233	4.9014	1.979	13.21	4.7577	5.5668	6.7399
sp|K1C15_SHEEP|	sp|K1C15_SHEEP|	49 kDa	17.497	3.254	11.763	1.979	7.7059	4.7577	4.639	4.8142
peroxiredoxin-like OS=Bombus vosnesenskii OX=207650 GN=LOC117233967 PE=4 SV=1	A0A6J3KBU5	27 kDa	8.2339	6.5079	7.8422	4.9476	6.6051	7.6123	7.4223	4.8142

digestive cysteine proteinase 1 OS=Bombus vosnesenskii OX=207650 GN=LOC117238749 PE=3 SV=1	A0A6J3L714	62 kDa	4.117	5.4233	7.8422	4.9476	9.9076	8.5638	4.639	9.6284
furin-like protease 2 isoform X3 OS=Bombus vosnesenskii OX=207650 GN=LOC117232940 PE=3 SV=1	A0A6J3K6D9 (+2)	141 kDa	8.2339	6.5079	3.9211	5.9371	7.7059	3.8061	7.4223	7.7027
Diphosphomevalonate decarboxylase OS=Bombus vosnesenskii OX=207650 GN=LOC117236200 PE=3 SV=1	A0A6J3KP45	43 kDa	1.0292	4.3386	9.8028	6.9266	8.8068	12.37	4.639	4.8142
apoptosis-inducing factor 3-like isoform X2 OS=Bombus vosnesenskii OX=207650 GN=LOC117234983 PE=4 SV=1	A0A6J3KKW3 (+1)	64 kDa	4.117	8.6772	8.8225	6.9266	8.8068	9.5154	3.7112	1.9257
Adenylyl cyclase-associated protein OS=Bombus vosnesenskii OX=207650 GN=LOC117233624 PE=3 SV=1	A0A6J3KA49 (+2)	58 kDa	9.2631	3.254	7.8422	4.9476	6.6051	9.5154	4.639	5.777
Epoxide hydrolase OS=Bombus vosnesenskii OX=207650 GN=LOC117234154 PE=3 SV=1	A0A6J3KD28	52 kDa	5.1462	5.4233	4.9014	4.9476	7.7059	11.418	6.4946	5.777
NADPH--cytochrome P450 reductase OS=Bombus vosnesenskii OX=207650 GN=LOC117236438 PE=3 SV=1	A0A6J3KQE9	79 kDa	7.2047	6.5079	5.8817	6.9266	4.4034	8.5638	8.3501	3.8514
Pyridoxal kinase OS=Bombus vosnesenskii OX=207650 GN=LOC117234550 PE=3 SV=1	A0A6J3KFW6	33 kDa	9.2631	5.4233	7.8422	4.9476	4.4034	5.7092	5.5668	8.6656
glycerol-3-phosphate phosphatase OS=Bombus vosnesenskii OX=207650 GN=LOC117234387 PE=3 SV=1	A0A6J3KHP6	34 kDa	7.2047	7.5926	7.8422	4.9476	5.5042	3.8061	8.3501	3.8514
isochorismatase domain-containing protein 2-like OS=Bombus vosnesenskii OX=207650 GN=LOC117231714 PE=3 SV=1	A0A6J3K1Y8	22 kDa	7.2047	7.5926	6.8619	4.9476	8.8068	3.8061	4.639	5.777

tubulin polymerization-promoting protein homolog OS=Bombus vosnesenskii OX=207650 GN=LOC117240160 PE=3 SV=1	A0A6J3L8J3	25 kDa	6.1754	5.4233	5.8817	6.9266	7.7059	4.7577	5.5668	6.7399
Acylphosphatase OS=Bombus vosnesenskii OX=207650 GN=LOC117230289 PE=3 SV=1	A0A6J3JTL5	15 kDa	9.2631	3.254	4.9014	3.9581	7.7059	6.6608	5.5668	6.7399
LOW QUALITY PROTEIN: peptidoglycan recognition protein 3-like OS=Bombus vosnesenskii OX=207650 GN=LOC117233578 PE=3 SV=1	A0A6J3K9X4	46 kDa	10.292	9.7619	3.9211	4.9476	5.5042	5.7092	4.639	3.8514
peptidylprolyl isomerase OS=Bombus vosnesenskii OX=207650 GN=LOC117239367 PE=4 SV=1	A0A6J3L643 (+1)	52 kDa	3.0877	5.4233	3.9211	5.9371	3.3025	10.467	7.4223	6.7399
calexcitin-2 OS=Bombus vosnesenskii OX=207650 GN=LOC117232823 PE=4 SV=1	A0A6J3K5N6	21 kDa	6.1754	5.4233	7.8422	6.9266	6.6051	2.8546	7.4223	5.777
omega-conotoxin-like protein 1 OS=Bombus vosnesenskii OX=207650 GN=LOC117232521 PE=4 SV=1	A0A6J3K3Y1	8 kDa	4.117	4.3386	6.8619	5.9371	5.5042	6.6608	8.3501	4.8142
esterase E4-like OS=Bombus vosnesenskii OX=207650 GN=LOC117235282 PE=4 SV=1	A0A6J3KLB0	64 kDa	17.497	6.5079	4.9014	2.9686	1.1008	2.8546	4.639	3.8514
clavesin-2 OS=Bombus vosnesenskii OX=207650 GN=LOC117236343 PE=4 SV=1	A0A6J3KPW1	33 kDa	2.0585	5.4233	4.9014	6.9266	5.5042	12.37	7.4223	0.96284
protein CutA homolog OS=Bombus vosnesenskii OX=207650 GN=LOC117243128 PE=3 SV=1	A0A6J3LM45	16 kDa	3.0877	4.3386	7.8422	6.9266	4.4034	5.7092	8.3501	5.777
EH domain-containing protein 3 OS=Bombus vosnesenskii OX=207650 GN=LOC117231106 PE=4 SV=1	A0A6J3JY90	107 kDa	6.1754	3.254	4.9014	5.9371	7.7059	8.5638	3.7112	2.8885
fumarate hydratase OS=Bombus vosnesenskii	A0A6J3K5M2	55 kDa	0	9.7619	7.8422	5.9371	6.6051	7.6123	4.639	2.8885

OX=207650 GN=LOC117232291 PE=3 SV=1										
Alpha-1,4 glucan phosphorylase OS=Bombus vosnesenskii OX=207650 GN=LOC117234120 PE=3 SV=1	A0A6J3KF07 (+1)	97 kDa	1.0292	5.4233	2.9408	8.9057	0	7.6123	11.134	7.7027
succinate-semialdehyde dehydrogenase [NADP(+)] GabD OS=Bombus vosnesenskii OX=207650 GN=LOC117230218 PE=4 SV=1	A0A6J3JR58	56 kDa	7.2047	8.6772	3.9211	3.9581	5.5042	9.5154	4.639	1.9257
haloacid dehalogenase-like hydrolase domain-containing protein 2 isoform X1 OS=Bombus vosnesenskii OX=207650 GN=LOC117232284 PE=3 SV=1	A0A6J3K5K6	28 kDa	11.322	2.1693	4.9014	3.9581	6.6051	4.7577	5.5668	4.8142
peptidylprolyl isomerase OS=Bombus vosnesenskii OX=207650 GN=LOC117241957 PE=4 SV=1	A0A6J3LHL2	12 kDa	4.117	4.3386	4.9014	2.9686	6.6051	7.6123	6.4946	7.7027
dynein assembly factor 5, axonemal isoform X2 OS=Bombus vosnesenskii OX=207650 GN=LOC117239165 PE=3 SV=1	A0A6J3L583 (+1)	119 kDa	7.2047	3.254	5.8817	3.9581	5.5042	3.8061	6.4946	4.8142
Glycine cleavage system H protein OS=Bombus vosnesenskii OX=207650 GN=LOC117233162 PE=3 SV=1	A0A6J3K7L0	19 kDa	0	8.6772	4.9014	4.9476	3.3025	7.6123	7.4223	6.7399
enoyl-CoA delta isomerase 1, mitochondrial-like isoform X1 OS=Bombus vosnesenskii OX=207650 GN=LOC117233157 PE=4 SV=1	A0A6J3K9A3 (+1)	31 kDa	4.117	3.254	6.8619	5.9371	9.9076	7.6123	4.639	2.8885
Glutathione peroxidase OS=Bombus vosnesenskii OX=207650 GN=LOC117231056 PE=3 SV=1	A0A6J3JW58	23 kDa	4.117	4.3386	3.9211	4.9476	3.3025	10.467	7.4223	3.8514
low-density lipoprotein receptor-related protein 2 OS=Bombus	A0A6J3LKB7	515 kDa	5.1462	6.5079	4.9014	2.9686	4.4034	11.418	1.8556	4.8142

vosnesenskii OX=207650 GN=LOC117242508 PE=4 SV=1										
myrosinase 1 OS=Bombus vosnesenskii OX=207650 GN=LOC117233446 PE=3 SV=1	A0A6J3K967	57 kDa	2.0585	3.254	7.8422	3.9581	6.6051	10.467	5.5668	0.96284
ejaculatory bulb-specific protein 3 OS=Bombus vosnesenskii OX=207650 GN=LOC117242113 PE=4 SV=1	A0A6J3LGA9	14 kDa	2.0585	8.6772	3.9211	5.9371	3.3025	7.6123	6.4946	4.8142
10-formyltetrahydrofolate dehydrogenase OS=Bombus vosnesenskii OX=207650 GN=LOC117242354 PE=3 SV=1	A0A6J3LLN7	100 kDa	0	6.5079	0	6.9266	4.4034	5.7092	5.5668	5.777
N-acetylglucosamine-6-sulfatase-like OS=Bombus vosnesenskii OX=207650 GN=LOC117234266 PE=4 SV=1	A0A6J3KGZ3	56 kDa	12.351	4.3386	4.9014	8.9057	2.2017	1.9031	3.7112	3.8514
palmitoyl-protein hydrolase OS=Bombus vosnesenskii OX=207650 GN=LOC117234497 PE=3 SV=1	A0A6J3KIE9	24 kDa	0	4.3386	3.9211	6.9266	0	6.6608	5.5668	12.517
beta-1,3-glucan-binding protein 1-like OS=Bombus vosnesenskii OX=207650 GN=LOC117234110 PE=3 SV=1	A0A6J3KG34	54 kDa	6.1754	6.5079	5.8817	3.9581	4.4034	2.8546	4.639	5.777
Elongation factor 1-alpha OS=Bombus vosnesenskii OX=207650 GN=LOC117239077 PE=3 SV=1	A0A6J3L6U0 (+2)	50 kDa	3.0877	4.3386	4.9014	6.9266	5.5042	7.6123	3.7112	5.777
Fumarylacetoacetase OS=Bombus vosnesenskii OX=207650 GN=LOC117231648 PE=3 SV=1	A0A6J3K0Z5	46 kDa	1.0292	3.254	3.9211	2.9686	13.21	8.5638	5.5668	1.9257
enoyl-[acyl-carrier-protein] reductase, mitochondrial isoform X1 OS=Bombus vosnesenskii OX=207650 GN=LOC117241787 PE=4 SV=1	A0A6J3LIU8	41 kDa	7.2047	3.254	3.9211	2.9686	6.6051	7.6123	3.7112	4.8142
uncharacterized protein LOC117230524 OS=Bombus	A0A6J3JUH5	79 kDa	2.0585	2.1693	4.9014	4.9476	3.3025	6.6608	8.3501	7.7027

vosnesenskii OX=207650 GN=LOC117230524 PE=4 SV=1										
guanine nucleotide-binding protein G(I)/G(S)/G(T) subunit beta-1 OS=Bombus vosnesenskii OX=207650 GN=LOC117243391 PE=3 SV=1	A0A6J3LRL8	37 kDa	0	2.1693	4.9014	7.9162	1.1008	8.5638	3.7112	9.6284
ATP synthase subunit alpha OS=Bombus vosnesenskii OX=207650 GN=LOC117235069 PE=3 SV=1	A0A6J3KHN1	59 kDa	0	7.5926	3.9211	3.9581	6.6051	13.322	3.7112	0
uncharacterized oxidoreductase YjmC-like OS=Bombus vosnesenskii OX=207650 GN=LOC117243183 PE=4 SV=1	A0A6J3LMC1	43 kDa	3.0877	5.4233	6.8619	2.9686	4.4034	4.7577	7.4223	3.8514
zinc carboxypeptidase-like OS=Bombus vosnesenskii OX=207650 GN=LOC117242130 PE=3 SV=1	A0A6J3LIC7	46 kDa	2.0585	7.5926	4.9014	4.9476	4.4034	2.8546	0.92779	9.6284
2-iminobutanoate/2-iminopropanoate deaminase OS=Bombus vosnesenskii OX=207650 GN=LOC117233713 PE=3 SV=1	A0A6J3KAM0	15 kDa	4.117	5.4233	3.9211	5.9371	5.5042	6.6608	3.7112	3.8514
matrix metalloproteinase-14 isoform X1 OS=Bombus vosnesenskii OX=207650 GN=LOC117233695 PE=3 SV=1	A0A6J3KAJ7 (+3)	67 kDa	10.292	4.3386	4.9014	3.9581	2.2017	0	3.7112	8.6656
cytochrome P450 6k1-like isoform X1 OS=Bombus vosnesenskii OX=207650 GN=LOC117242889 PE=3 SV=1	A0A6J3LM90	59 kDa	1.0292	4.3386	6.8619	8.9057	4.4034	6.6608	2.7834	1.9257
uncharacterized protein LOC117239118 isoform X1 OS=Bombus vosnesenskii OX=207650 GN=LOC117239118 PE=4 SV=1	A0A6J3L3X2	40 kDa	3.0877	3.254	4.9014	1.979	6.6051	3.8061	6.4946	4.8142
glutamate decarboxylase 1 OS=Bombus vosnesenskii OX=207650	A0A6J3JTC6	55 kDa	5.1462	5.4233	3.9211	1.979	11.008	1.9031	4.639	0.96284

GN=LOC117230530 PE=3 SV=1										
testicular acid phosphatase homolog isoform X2 OS=Bombus vosnesenskii OX=207650 GN=LOC117243362 PE=4 SV=1	A0A6J3LRI5 (+1)	45 kDa	3.0877	5.4233	3.9211	2.9686	4.4034	6.6608	5.5668	2.8885
thioredoxin, mitochondrial-like OS=Bombus vosnesenskii OX=207650 GN=LOC117243081 PE=4 SV=1	A0A6J3LLT4	15 kDa	5.1462	2.1693	3.9211	4.9476	2.2017	6.6608	5.5668	3.8514
Glucosylceramidase OS=Bombus vosnesenskii OX=207650 GN=LOC117233136 PE=3 SV=1	A0A6J3K7F7 (+1)	58 kDa	2.0585	2.1693	3.9211	3.9581	5.5042	10.467	1.8556	3.8514
putative aminopeptidase-2 isoform X1 OS=Bombus vosnesenskii OX=207650 GN=LOC117233796 PE=3 SV=1	A0A6J3KAY4 (+1)	313 kDa	2.0585	2.1693	8.8225	1.979	6.6051	7.6123	4.639	0.96284
long-chain-fatty-acid--CoA ligase ACSBG2 isoform X2 OS=Bombus vosnesenskii OX=207650 GN=LOC117235086 PE=4 SV=1	A0A6J3KHT4 (+1)	77 kDa	8.2339	5.4233	3.9211	1.979	4.4034	7.6123	1.8556	0.96284
pyridoxal 5'-phosphate synthase OS=Bombus vosnesenskii OX=207650 GN=LOC117235120 PE=3 SV=1	A0A6J3KKD2	28 kDa	7.2047	3.254	3.9211	4.9476	3.3025	4.7577	2.7834	4.8142
uncharacterized protein LOC117241701 OS=Bombus vosnesenskii OX=207650 GN=LOC117241701 PE=4 SV=1	A0A6J3LEG2	17 kDa	3.0877	2.1693	7.8422	2.9686	4.4034	4.7577	1.8556	5.777
UDP-glucuronosyltransferase 2B15-like isoform X1 OS=Bombus vosnesenskii OX=207650 GN=LOC117233445 PE=4 SV=1	A0A6J3KAD6	60 kDa	2.0585	5.4233	6.8619	6.9266	4.4034	2.8546	5.5668	0.96284
uncharacterized protein LOC117233304 OS=Bombus vosnesenskii OX=207650 GN=LOC117233304 PE=4 SV=1	A0A6J3KA22	11 kDa	1.0292	8.6772	4.9014	7.9162	1.1008	3.8061	2.7834	3.8514

pyrimidine-specific ribonucleoside hydrolase RihA-like isoform X3 OS=Bombus vosnesenskii OX=207650 GN=LOC117236557 PE=3 SV=1	A0A6J3KQW3 (+2)	37 kDa	3.0877	4.3386	5.8817	2.9686	6.6051	0.95154	4.639	5.777
regulator of chromosome condensation isoform X1 OS=Bombus vosnesenskii OX=207650 GN=LOC117239731 PE=4 SV=1	A0A6J3LBW7 (+1)	48 kDa	0	2.1693	3.9211	4.9476	1.1008	5.7092	5.5668	9.6284
carbonic anhydrase 2-like OS=Bombus vosnesenskii OX=207650 GN=LOC117230637 PE=3 SV=1	A0A6J3JUX8	38 kDa	10.292	1.0847	4.9014	0	8.8068	1.9031	0	7.7027
Sulfhydryl oxidase OS=Bombus vosnesenskii OX=207650 GN=LOC117231249 PE=3 SV=1	A0A6J3JZ28	75 kDa	7.2047	3.254	2.9408	3.9581	6.6051	2.8546	2.7834	3.8514
Cell division control protein 42 homolog OS=Bombus vosnesenskii OX=207650 GN=LOC117230940 PE=3 SV=1	A0A6J3JVC5	21 kDa	3.0877	3.254	3.9211	3.9581	3.3025	5.7092	4.639	4.8142
thioredoxin-like protein 1 OS=Bombus vosnesenskii OX=207650 GN=LOC117243430 PE=4 SV=1	A0A6J3LMV1	33 kDa	1.0292	3.254	3.9211	2.9686	5.5042	7.6123	4.639	4.8142
GDP-L-fucose synthase OS=Bombus vosnesenskii OX=207650 GN=LOC117241847 PE=3 SV=1	A0A6J3LJ88	37 kDa	13.38	2.1693	3.9211	1.979	3.3025	1.9031	2.7834	3.8514
probable chitinase 2 OS=Bombus vosnesenskii OX=207650 GN=LOC117243229 PE=4 SV=1	A0A6J3LNU9	45 kDa	0	3.254	7.8422	4.9476	0	5.7092	0.92779	10.591
Transgelin OS=Bombus vosnesenskii OX=207650 GN=LOC117240275 PE=3 SV=1	A0A6J3L983	20 kDa	2.0585	3.254	4.9014	4.9476	4.4034	2.8546	3.7112	4.8142
Pyruvate carboxylase OS=Bombus vosnesenskii OX=207650 GN=LOC117233613 PE=4 SV=1	A0A6J3KA48 (+1)	132 kDa	2.0585	4.3386	2.9408	2.9686	2.2017	7.6123	5.5668	2.8885

dihydropteridine reductase OS=Bombus vosnesenskii OX=207650 GN=LOC117240234 PE=4 SV=1	A0A6J3LBR0	26 kDa	2.0585	3.254	5.8817	4.9476	4.4034	3.8061	4.639	2.8885
Succinate--CoA ligase [ADP/GDP-forming] subunit alpha, mitochondrial OS=Bombus vosnesenskii OX=207650 GN=LOC117232354 PE=3 SV=1	A0A6J3K3A9 (+1)	36 kDa	4.117	2.1693	5.8817	1.979	6.6051	4.7577	4.639	2.8885
ras-related protein Rap1 OS=Bombus vosnesenskii OX=207650 GN=LOC117232864 PE=4 SV=1	A0A6J3K6Z5	21 kDa	1.0292	4.3386	2.9408	3.9581	3.3025	7.6123	1.8556	6.7399
Succinate--CoA ligase [ADP-forming] subunit beta, mitochondrial OS=Bombus vosnesenskii OX=207650 GN=LOC117230566 PE=3 SV=1	A0A6J3JTG9	49 kDa	0	4.3386	2.9408	2.9686	0	6.6608	4.639	7.7027
modular serine protease-like OS=Bombus vosnesenskii OX=207650 GN=LOC117241982 PE=4 SV=1	A0A6J3LJY1	71 kDa	4.117	4.3386	3.9211	1.979	4.4034	4.7577	4.639	1.9257
vesicle-fusing ATPase OS=Bombus vosnesenskii OX=207650 GN=LOC117239275 PE=4 SV=1	A0A6J3L7V0	89 kDa	2.0585	5.4233	5.8817	3.9581	5.5042	4.7577	2.7834	1.9257
Peptidyl-prolyl cis-trans isomerase OS=Bombus vosnesenskii OX=207650 GN=LOC117238473 PE=3 SV=1	A0A6J3L215	18 kDa	5.1462	2.1693	4.9014	2.9686	3.3025	2.8546	6.4946	1.9257
Calcyclin-binding protein OS=Bombus vosnesenskii OX=207650 GN=LOC117230128 PE=4 SV=1	A0A6J3JQX1	26 kDa	5.1462	1.0847	1.9606	3.9581	2.2017	5.7092	4.639	4.8142
acyl-CoA-binding protein homolog OS=Bombus vosnesenskii OX=207650 GN=LOC117236283 PE=4 SV=1	A0A6J3KSA8	10 kDa	8.2339	3.254	3.9211	3.9581	3.3025	2.8546	1.8556	4.8142
Ferritin OS=Bombus vosnesenskii OX=207650	A0A6J3LPQ7	26 kDa	1.0292	3.254	8.8225	4.9476	7.7059	2.8546	0.92779	2.8885

GN=LOC117242977 PE=3 SV=1										
phosphatidylinositol transfer protein alpha isoform OS=Bombus vosnesenskii OX=207650 GN=LOC117236864 PE=4 SV=1	A0A6J3KSQ6	32 kDa	0	2.1693	2.9408	2.9686	1.1008	6.6608	6.4946	8.6656
uncharacterized protein LOC117235148 OS=Bombus vosnesenskii OX=207650 GN=LOC117235148 PE=4 SV=1	A0A6J3KI42	28 kDa	6.1754	1.0847	5.8817	7.9162	3.3025	0.95154	0	4.8142
phenoloxidase 1 OS=Bombus vosnesenskii OX=207650 GN=LOC117243143 PE=4 SV=1	A0A6J3LL92	80 kDa	4.117	1.0847	8.8225	2.9686	3.3025	5.7092	3.7112	0
delta(3,5)-Delta(2,4)-dienoyl-CoA isomerase, mitochondrial OS=Bombus vosnesenskii OX=207650 GN=LOC117237801 PE=3 SV=1	A0A6J3L0S6	34 kDa	0	3.254	0.98028	3.9581	1.1008	8.5638	3.7112	7.7027
3-hydroxyisobutyryl-CoA hydrolase, mitochondrial OS=Bombus vosnesenskii OX=207650 GN=LOC117230463 PE=4 SV=1	A0A6J3JUQ9 (+2)	43 kDa	3.0877	3.254	1.9606	1.979	3.3025	8.5638	2.7834	3.8514
dehydrogenase/reductase SDR family member 4 OS=Bombus vosnesenskii OX=207650 GN=LOC117233634 PE=4 SV=1	A0A6J3KA67	29 kDa	3.0877	3.254	4.9014	2.9686	3.3025	4.7577	4.639	2.8885
ADP-ribosylation factor GTPase-activating protein 2 isoform X2 OS=Bombus vosnesenskii OX=207650 GN=LOC117233115 PE=4 SV=1	A0A6J3KAA4	59 kDa	4.117	2.1693	4.9014	2.9686	4.4034	3.8061	2.7834	2.8885
Peptidoglycan-recognition protein OS=Bombus vosnesenskii OX=207650 GN=LOC117239036 PE=3 SV=1	A0A6J3L883	21 kDa	3.0877	5.4233	1.9606	1.979	5.5042	2.8546	3.7112	0.96284
xanthine dehydrogenase OS=Bombus vosnesenskii OX=207650 GN=LOC117243463 PE=3 SV=1	A0A6J3LRY3	150 kDa	6.1754	2.1693	2.9408	1.979	7.7059	6.6608	1.8556	0

uncharacterized protein LOC117242277 OS=Bombus vosnesenskii OX=207650 GN=LOC117242277 PE=4 SV=1	A0A6J3LHI0	28 kDa	0	4.3386	3.9211	5.9371	1.1008	4.7577	4.639	4.8142
LOW QUALITY PROTEIN: transmembrane protease serine 9-like OS=Bombus vosnesenskii OX=207650 GN=LOC117231737 PE=4 SV=1	A0A6J3K1G1	63 kDa	2.0585	1.0847	6.8619	1.979	4.4034	1.9031	0	9.6284
guanine nucleotide-binding protein subunit beta-like protein OS=Bombus vosnesenskii OX=207650 GN=LOC117235788 PE=3 SV=1	A0A6J3KLG9	36 kDa	16.468	2.1693	3.9211	0	5.5042	2.8546	0	0
Adenosine kinase OS=Bombus vosnesenskii OX=207650 GN=LOC117241673 PE=3 SV=1	A0A6J3LE00 (+2)	38 kDa	1.0292	5.4233	4.9014	1.979	3.3025	5.7092	3.7112	1.9257
venom acid phosphatase Acph-1-like isoform X1 OS=Bombus vosnesenskii OX=207650 GN=LOC117242856 PE=4 SV=1	A0A6J3LJQ5 (+1)	45 kDa	1.0292	4.3386	4.9014	1.979	5.5042	3.8061	4.639	1.9257
Coronin OS=Bombus vosnesenskii OX=207650 GN=LOC117236453 PE=3 SV=1	A0A6J3KQJ2	55 kDa	0	2.1693	3.9211	3.9581	6.6051	2.8546	1.8556	6.7399
cofilin/actin-depolymerizing factor homolog isoform X1 OS=Bombus vosnesenskii OX=207650 GN=LOC117236984 PE=3 SV=1	A0A6J3KX03	17 kDa	3.0877	4.3386	1.9606	3.9581	4.4034	2.8546	2.7834	4.8142
Mevalonate kinase OS=Bombus vosnesenskii OX=207650 GN=LOC117239257 PE=3 SV=1	A0A6J3L5P2	43 kDa	1.0292	1.0847	5.8817	2.9686	5.5042	5.7092	2.7834	1.9257
Isovaleryl-CoA dehydrogenase, mitochondrial OS=Bombus vosnesenskii OX=207650 GN=LOC117234547 PE=3 SV=1	A0A6J3KF18	47 kDa	0	3.254	3.9211	3.9581	3.3025	7.6123	3.7112	0.96284
hypoxia up-regulated protein 1 isoform X2 OS=Bombus vosnesenskii OX=207650 GN=LOC117230237 PE=3 SV=1	A0A6J3JT79 (+1)	106 kDa	0	0	4.9014	3.9581	0	10.467	4.639	1.9257

laminin subunit alpha OS=Bombus vosnesenskii OX=207650 GN=LOC117242057 PE=4 SV=1	A0A6J3LHZ6	406 kDa	7.2047	3.254	1.9606	2.9686	1.1008	1.9031	2.7834	3.8514
10 kDa heat shock protein, mitochondrial OS=Bombus vosnesenskii OX=207650 GN=LOC117242850 PE=3 SV=1	A0A6J3LKJ7	11 kDa	4.117	1.0847	3.9211	1.979	3.3025	1.9031	3.7112	3.8514
Succinate--CoA ligase [GDP-forming] subunit beta, mitochondrial OS=Bombus vosnesenskii OX=207650 GN=LOC117242998 PE=3 SV=1	A0A6J3LKT3 (+1)	185 kDa	8.2339	1.0847	1.9606	0.98952	4.4034	6.6608	1.8556	0.96284
hexamerin-like OS=Bombus vosnesenskii OX=207650 GN=LOC117236575 PE=4 SV=1	A0A6J3KR03	81 kDa	1.0292	4.3386	1.9606	3.9581	1.1008	2.8546	8.3501	0.96284
14-3-3 protein zeta isoform X1 OS=Bombus vosnesenskii OX=207650 GN=LOC117234426 PE=3 SV=1	A0A6J3KE87 (+2)	28 kDa	1.0292	4.3386	0.98028	4.9476	2.2017	7.6123	1.8556	3.8514
UV excision repair protein RAD23 OS=Bombus vosnesenskii OX=207650 GN=LOC117235163 PE=3 SV=1	A0A6J3KLV9	40 kDa	0	5.4233	4.9014	1.979	2.2017	3.8061	1.8556	4.8142
neurotrimin-like OS=Bombus vosnesenskii OX=207650 GN=LOC117234800 PE=4 SV=1	A0A6J3KIX8	55 kDa	0	1.0847	3.9211	4.9476	1.1008	3.8061	1.8556	9.6284
filamin-A isoform X1 OS=Bombus vosnesenskii OX=207650 GN=LOC117242997 PE=3 SV=1	A0A6J3LKE3 (+3)	263 kDa	3.0877	2.1693	3.9211	1.979	3.3025	3.8061	3.7112	3.8514
phosphoserine transaminase OS=Bombus vosnesenskii OX=207650 GN=LOC117232382 PE=3 SV=1	A0A6J3K4Y9	41 kDa	3.0877	3.254	4.9014	1.979	2.2017	5.7092	2.7834	0.96284
MD-2-related lipid-recognition protein-like OS=Bombus vosnesenskii OX=207650 GN=LOC117233559 PE=3 SV=1	A0A6J3K9R7	18 kDa	4.117	2.1693	1.9606	3.9581	3.3025	2.8546	3.7112	1.9257

cysteine-rich venom protein 1-like OS=Bombus vosnesenskii OX=207650 GN=LOC117237301 PE=4 SV=1	A0A6J3KVJ1	11 kDa	1.0292	3.254	3.9211	4.9476	4.4034	4.7577	2.7834	0.96284
NPC intracellular cholesterol transporter 2 homolog a-like OS=Bombus vosnesenskii OX=207650 GN=LOC117243477 PE=3 SV=1	A0A6J3LNA2	17 kDa	4.117	3.254	1.9606	1.979	4.4034	2.8546	4.639	2.8885
glyoxylate reductase/hydroxypyruvate reductase-like OS=Bombus vosnesenskii OX=207650 GN=LOC117241767 PE=3 SV=1	A0A6J3LIS1	35 kDa	0	6.5079	1.9606	4.9476	1.1008	6.6608	2.7834	1.9257
mitochondrial-processing peptidase subunit alpha OS=Bombus vosnesenskii OX=207650 GN=LOC117235550 PE=3 SV=1	A0A6J3KNY4	62 kDa	0	0	2.9408	2.9686	2.2017	4.7577	2.7834	6.7399
ubiquitin-conjugating enzyme E2-17 kDa OS=Bombus vosnesenskii OX=207650 GN=LOC117233838 PE=3 SV=1	A0A6J3KB77	17 kDa	0	2.1693	3.9211	2.9686	0	4.7577	3.7112	5.777
fasciclin-2 isoform X3 OS=Bombus vosnesenskii OX=207650 GN=LOC117233646 PE=4 SV=1	A0A6J3KA86 (+5)	100 kDa	2.0585	2.1693	4.9014	2.9686	4.4034	2.8546	2.7834	2.8885
Apolipoprotein D OS=Bombus vosnesenskii OX=207650 GN=LOC117232811 PE=3 SV=1	A0A6J3K6Q2	30 kDa	2.0585	4.3386	4.9014	1.979	1.1008	2.8546	3.7112	2.8885
titin isoform X4 OS=Bombus vosnesenskii OX=207650 GN=LOC117234474 PE=4 SV=1	A0A6J3KGZ1 (+2)	206 kDa	1.0292	1.0847	1.9606	1.979	1.1008	3.8061	4.639	6.7399
Dynein light chain OS=Bombus vosnesenskii OX=207650 GN=LOC117242439 PE=3 SV=1	A0A6J3LIA8	10 kDa	4.117	2.1693	0.98028	0.98952	3.3025	4.7577	2.7834	4.8142
CD109 antigen-like isoform X2 OS=Bombus vosnesenskii OX=207650 GN=LOC117235688 PE=3 SV=1	A0A6J3KKY8 (+3)	171 kDa	3.0877	2.1693	1.9606	3.9581	2.2017	2.8546	3.7112	4.8142

uncharacterized protein LOC117230536 OS=Bombus vosnesenskii OX=207650 GN=LOC117230536 PE=4 SV=1	A0A6J3JUJ1	14 kDa	7.2047	1.0847	3.9211	3.9581	5.5042	0	0	1.9257
vitamin K-dependent protein C-like OS=Bombus vosnesenskii OX=207650 GN=LOC117231419 PE=4 SV=1	A0A6J3JY39	35 kDa	2.0585	3.254	0.98028	2.9686	3.3025	2.8546	2.7834	3.8514
Triokinase/FMN cyclase OS=Bombus vosnesenskii OX=207650 GN=LOC117239461 PE=4 SV=1	A0A6J3L5L3	58 kDa	3.0877	5.4233	2.9408	1.979	3.3025	3.8061	2.7834	0.96284
Dihydropyrimidine dehydrogenase [NADP(+)] OS=Bombus vosnesenskii OX=207650 GN=LOC117234475 PE=3 SV=1	A0A6J3KEM5	112 kDa	2.0585	1.0847	0.98028	1.979	4.4034	4.7577	4.639	1.9257
nitrilase and fragile histidine triad fusion protein NitFhit isoform X2 OS=Bombus vosnesenskii OX=207650 GN=LOC117238515 PE=4 SV=1	A0A6J3L5P5 (+1)	32 kDa	4.117	3.254	1.9606	1.979	2.2017	4.7577	3.7112	1.9257
ATP-dependent (S)-NAD(P)H-hydrate dehydratase OS=Bombus vosnesenskii OX=207650 GN=LOC117233515 PE=3 SV=1	A0A6J3KAN5 (+1)	36 kDa	0	2.1693	0.98028	2.9686	3.3025	6.6608	3.7112	1.9257
arginine kinase OS=Bombus vosnesenskii OX=207650 GN=LOC117230705 PE=3 SV=1	A0A6J3JW35	42 kDa	7.2047	1.0847	1.9606	0	5.5042	5.7092	0.92779	0.96284
Nuclear migration protein nudC OS=Bombus vosnesenskii OX=207650 GN=LOC117232109 PE=3 SV=1	A0A6J3K3R6	39 kDa	2.0585	2.1693	2.9408	0.98952	1.1008	2.8546	3.7112	4.8142
S-methyl-5'-thioadenosine phosphorylase OS=Bombus vosnesenskii OX=207650 GN=LOC117237214 PE=3 SV=1	A0A6J3KU08	31 kDa	1.0292	2.1693	3.9211	2.9686	3.3025	0.95154	3.7112	2.8885
plastin-2 isoform X2 OS=Bombus vosnesenskii OX=207650	A0A6J3KFV5 (+1)	71 kDa	3.0877	1.0847	1.9606	2.9686	4.4034	1.9031	3.7112	3.8514

GN=LOC117234674 PE=4 SV=1										
molybdenum cofactor biosynthesis protein 1 isoform X1 OS=Bombus vosnesenskii OX=207650 GN=LOC117236876 PE=3 SV=1	A0A6J3KSU9 (+1)	62 kDa	6.1754	2.1693	3.9211	1.979	1.1008	0.95154	2.7834	2.8885
muscle LIM protein Mlp84B-like isoform X2 OS=Bombus vosnesenskii OX=207650 GN=LOC117242615 PE=4 SV=1	A0A6J3LJ87 (+1)	53 kDa	2.0585	3.254	1.9606	2.9686	4.4034	2.8546	2.7834	2.8885
basigin OS=Bombus vosnesenskii OX=207650 GN=LOC117233787 PE=4 SV=1	A0A6J3KCS7	40 kDa	4.117	2.1693	2.9408	1.979	4.4034	2.8546	1.8556	2.8885
NSFL1 cofactor p47 OS=Bombus vosnesenskii OX=207650 GN=LOC117236632 PE=4 SV=1	A0A6J3KRE3	43 kDa	1.0292	2.1693	4.9014	1.979	4.4034	4.7577	1.8556	1.9257
heterogeneous nuclear ribonucleoprotein Q isoform X6 OS=Bombus vosnesenskii OX=207650 GN=LOC117232048 PE=4 SV=1	A0A6J3K1W9 (+5)	74 kDa	2.0585	1.0847	3.9211	2.9686	2.2017	2.8546	1.8556	4.8142
Dihydrolipoamide acetyltransferase component of pyruvate dehydrogenase complex OS=Bombus vosnesenskii OX=207650 GN=LOC117233204 PE=3 SV=1	A0A6J3K8Z9	64 kDa	0	4.3386	3.9211	1.979	1.1008	3.8061	3.7112	3.8514
actin-interacting protein 1 isoform X1 OS=Bombus vosnesenskii OX=207650 GN=LOC117233927 PE=4 SV=1	A0A6J3KF08 (+1)	66 kDa	7.2047	3.254	5.8817	0	3.3025	0	0.92779	0.96284
NAD(P)H-hydrate epimerase OS=Bombus vosnesenskii OX=207650 GN=LOC117231992 PE=3 SV=1	A0A6J3K1J2	26 kDa	4.117	2.1693	2.9408	0.98952	3.3025	2.8546	2.7834	2.8885
group XV phospholipase A2-like OS=Bombus vosnesenskii OX=207650 GN=LOC117236116 PE=4 SV=1	A0A6J3KNS9	46 kDa	4.117	3.254	1.9606	0.98952	4.4034	3.8061	2.7834	0.96284

Tetraspanin OS=Bombus vosnesenskii OX=207650 GN=LOC117236013 PE=3 SV=1	A0A6J3KQU6 (+1)	26 kDa	8.2339	3.254	1.9606	1.979	3.3025	0.95154	0.92779	1.9257
Sorbitol dehydrogenase OS=Bombus vosnesenskii OX=207650 GN=LOC117233279 PE=3 SV=1	A0A6J3K9X7	38 kDa	2.0585	3.254	0.98028	2.9686	3.3025	4.7577	1.8556	0.96284
6-phosphogluconate dehydrogenase, decarboxylating OS=Bombus vosnesenskii OX=207650 GN=LOC117241845 PE=3 SV=1	A0A6J3LEY8	53 kDa	0	3.254	1.9606	2.9686	0	5.7092	2.7834	3.8514
elongation factor 1-beta' OS=Bombus vosnesenskii OX=207650 GN=LOC117235556 PE=3 SV=1	A0A6J3KKA6	25 kDa	1.0292	3.254	2.9408	3.9581	3.3025	2.8546	2.7834	0.96284
probable elongation factor 1-delta isoform X1 OS=Bombus vosnesenskii OX=207650 GN=LOC117230147 PE=3 SV=1	A0A6J3JS28 (+1)	48 kDa	3.0877	3.254	0.98028	2.9686	2.2017	1.9031	2.7834	1.9257
uncharacterized protein LOC117234454 OS=Bombus vosnesenskii OX=207650 GN=LOC117234454 PE=4 SV=1	A0A6J3KEF4	257 kDa	4.117	2.1693	1.9606	1.979	3.3025	5.7092	0.92779	0.96284
astakine-like isoform X2 OS=Bombus vosnesenskii OX=207650 GN=LOC117240206 PE=4 SV=1	A0A6J3L9P6 (+1)	14 kDa	2.0585	4.3386	3.9211	0.98952	1.1008	3.8061	2.7834	1.9257
endoplasmic reticulum resident protein 29 OS=Bombus vosnesenskii OX=207650 GN=LOC117236860 PE=4 SV=1	A0A6J3KSR8	27 kDa	4.117	1.0847	2.9408	0.98952	3.3025	2.8546	3.7112	1.9257
xaa-Pro aminopeptidase ApepP isoform X1 OS=Bombus vosnesenskii OX=207650 GN=LOC117230786 PE=3 SV=1	A0A6J3JWG5 (+1)	73 kDa	6.1754	1.0847	2.9408	0.98952	3.3025	2.8546	1.8556	1.9257
adenylate kinase isoenzyme 1 OS=Bombus vosnesenskii OX=207650 GN=LOC117236063 PE=3 SV=1	A0A6J3KN22	21 kDa	5.1462	2.1693	1.9606	0.98952	5.5042	0.95154	2.7834	1.9257

T-complex protein 1 subunit beta OS=Bombus vosnesenskii OX=207650 GN=LOC117233093 PE=3 SV=1	A0A6J3KA42	58 kDa	1.0292	0	1.9606	4.9476	2.2017	2.8546	4.639	2.8885
Cystathionine beta-synthase OS=Bombus vosnesenskii OX=207650 GN=LOC117231499 PE=3 SV=1	A0A6J3K028 (+1)	48 kDa	0	4.3386	2.9408	1.979	1.1008	5.7092	2.7834	1.9257
cytochrome b5-like OS=Bombus vosnesenskii OX=207650 GN=LOC117230358 PE=3 SV=1	A0A6J3JU52	14 kDa	0	4.3386	0.98028	2.9686	2.2017	4.7577	1.8556	3.8514
N-acetyl-D-glucosamine kinase OS=Bombus vosnesenskii OX=207650 GN=LOC117240262 PE=3 SV=1	A0A6J3LE03 (+1)	45 kDa	7.2047	1.0847	3.9211	0.98952	4.4034	1.9031	0	0
tr|A0A6J3KRA2|A0A6J3KRA2_9HYME-DECOY	tr|A0A6J3KRA2|A0A6J3KRA2_9HYME-DECOY	?	0	3.254	0.98028	2.9686	0	1.9031	4.639	1.9257
glucose-6-phosphate 1-epimerase OS=Bombus vosnesenskii OX=207650 GN=LOC117242678 PE=3 SV=1	A0A6J3LJ93 (+2)	32 kDa	0	1.0847	0.98028	2.9686	0	3.8061	4.639	5.777
Adenylosuccinate lyase OS=Bombus vosnesenskii OX=207650 GN=LOC117243150 PE=3 SV=1	A0A6J3LQJ7 (+1)	55 kDa	0	6.5079	0.98028	4.9476	0	4.7577	2.7834	0
AP complex subunit beta OS=Bombus vosnesenskii OX=207650 GN=LOC117237848 PE=3 SV=1	A0A6J3KY96 (+1)	103 kDa	5.1462	2.1693	2.9408	2.9686	1.1008	2.8546	0.92779	0.96284
uncharacterized protein LOC117230535 OS=Bombus vosnesenskii OX=207650 GN=LOC117230535 PE=4 SV=1	A0A6J3JV74	14 kDa	3.0877	2.1693	1.9606	2.9686	4.4034	1.9031	0.92779	2.8885
Cytosol aminopeptidase OS=Bombus vosnesenskii OX=207650 GN=LOC117238672 PE=3 SV=1	A0A6J3L338	55 kDa	3.0877	2.1693	1.9606	1.979	4.4034	3.8061	0	1.9257
Eukaryotic translation initiation factor 3 subunit B OS=Bombus vosnesenskii OX=207650	A0A6J3LJR1	81 kDa	3.0877	3.254	3.9211	0.98952	2.2017	3.8061	1.8556	0

GN=LOC117242857 PE=3 SV=1										
Ferritin OS=Bombus vosnesenskii OX=207650 GN=LOC117242978 PE=3 SV=1	A0A6J3LL74	25 kDa	1.0292	2.1693	4.9014	0	6.6051	2.8546	0	2.8885
Glucosylceramidase OS=Bombus vosnesenskii OX=207650 GN=LOC117233260 PE=3 SV=1	A0A6J3K860	58 kDa	0	0	2.9408	3.9581	1.1008	0.95154	0	10.591
uncharacterized peptidase C1-like protein F26E4.3 isoform X1 OS=Bombus vosnesenskii OX=207650 GN=LOC117232051 PE=3 SV=1	A0A6J3K1X4 (+1)	53 kDa	2.0585	3.254	1.9606	1.979	3.3025	1.9031	1.8556	1.9257
inositol-3-phosphate synthase OS=Bombus vosnesenskii OX=207650 GN=LOC117237148 PE=3 SV=1	A0A6J3KX50 (+1)	57 kDa	4.117	1.0847	0.98028	0.98952	5.5042	2.8546	1.8556	1.9257
Histone H2B OS=Bombus vosnesenskii OX=207650 GN=LOC117233847 PE=3 SV=1	A0A6J3KB65	14 kDa	1.0292	2.1693	1.9606	0.98952	2.2017	4.7577	2.7834	2.8885
probable medium-chain specific acyl-CoA dehydrogenase, mitochondrial OS=Bombus vosnesenskii OX=207650 GN=LOC117233161 PE=3 SV=1	A0A6J3K9A8	46 kDa	1.0292	1.0847	0.98028	0.98952	4.4034	2.8546	3.7112	1.9257
Bleomycin hydrolase OS=Bombus vosnesenskii OX=207650 GN=LOC117235680 PE=3 SV=1	A0A6J3KLG0 (+1)	52 kDa	1.0292	3.254	1.9606	1.979	4.4034	3.8061	1.8556	0
glyoxylate reductase/hydroxypyruvate reductase OS=Bombus vosnesenskii OX=207650 GN=LOC117231482 PE=3 SV=1	A0A6J3JYC3	40 kDa	0	3.254	0.98028	3.9581	2.2017	3.8061	3.7112	0.96284
Phosphotriesterase-related protein OS=Bombus vosnesenskii OX=207650 GN=LOC117234443 PE=3 SV=1	A0A6J3KEL5	40 kDa	1.0292	2.1693	2.9408	0.98952	7.7059	2.8546	1.8556	0
uncharacterized protein LOC117235222 OS=Bombus	A0A6J3KIE1	76 kDa	2.0585	1.0847	0.98028	0.98952	2.2017	0.95154	0	8.6656

vosnesenskii OX=207650 GN=LOC117235222 PE=4 SV=1										
Eukaryotic translation initiation factor 2A OS=Bombus vosnesenskii OX=207650 GN=LOC117237210 PE=3 SV=1	A0A6J3KU02	65 kDa	5.1462	0	0.98028	1.979	4.4034	3.8061	2.7834	0
neprilysin-like OS=Bombus vosnesenskii OX=207650 GN=LOC117237088 PE=3 SV=1	A0A6J3KT95	85 kDa	0	0	0	2.9686	0	0.95154	0	14.443
cytosolic non-specific dipeptidase OS=Bombus vosnesenskii OX=207650 GN=LOC117242103 PE=3 SV=1	A0A6J3LFN9	53 kDa	1.0292	1.0847	2.9408	0.98952	2.2017	2.8546	3.7112	2.8885
vanin-like protein 1 OS=Bombus vosnesenskii OX=207650 GN=LOC117238482 PE=3 SV=1	A0A6J3L230	58 kDa	3.0877	3.254	0.98028	0.98952	2.2017	1.9031	1.8556	3.8514
Acetyltransferase component of pyruvate dehydrogenase complex OS=Bombus vosnesenskii OX=207650 GN=LOC117242230 PE=3 SV=1	A0A6J3LGV9	53 kDa	1.0292	3.254	0	3.9581	1.1008	4.7577	1.8556	1.9257
cysteine-rich with EGF-like domain protein 2 OS=Bombus vosnesenskii OX=207650 GN=LOC117234786 PE=4 SV=1	A0A6J3KGE0	42 kDa	1.0292	1.0847	1.9606	3.9581	0	0.95154	3.7112	2.8885
troponin I 2-like isoform X1 OS=Bombus vosnesenskii OX=207650 GN=LOC117236763 PE=4 SV=1	A0A6J3KR96 (+7)	46 kDa	0	2.1693	1.9606	1.979	0	3.8061	1.8556	3.8514
Bee-milk protein OS=Bombus vosnesenskii OX=207650 GN=LOC117243318 PE=3 SV=1	A0A6J3LM61	48 kDa	0	1.0847	0	1.979	0	7.6123	0.92779	4.8142
probable salivary secreted peptide OS=Bombus vosnesenskii OX=207650 GN=LOC117234813 PE=4 SV=1	A0A6J3KJ03	15 kDa	0	1.0847	4.9014	0.98952	4.4034	0	0	5.777
Acylamino-acid-releasing enzyme OS=Bombus vosnesenskii OX=207650	A0A6J3LKW2 (+1)	86 kDa	2.0585	3.254	0.98028	0.98952	2.2017	2.8546	0.92779	1.9257

GN=LOC117242890 PE=3 SV=1										
arrestin domain-containing protein 17 isoform X1 OS=Bombus vosnesenskii OX=207650 GN=LOC117234305 PE=3 SV=1	A0A6J3KDV9	44 kDa	3.0877	0	0.98028	0.98952	3.3025	3.8061	2.7834	1.9257
Dolichyl-diphosphooligosaccharide--protein glycosyltransferase subunit 2 OS=Bombus vosnesenskii OX=207650 GN=LOC117234521 PE=3 SV=1	A0A6J3KHD6	73 kDa	0	3.254	1.9606	0.98952	2.2017	2.8546	3.7112	0.96284
heparanase-like OS=Bombus vosnesenskii OX=207650 GN=LOC117240191 PE=3 SV=1	A0A6J3L9X2	60 kDa	0	1.0847	1.9606	0.98952	4.4034	3.8061	3.7112	0.96284
coproporphyrinogen oxidase OS=Bombus vosnesenskii OX=207650 GN=LOC117240248 PE=3 SV=1	A0A6J3LA96	44 kDa	2.0585	2.1693	1.9606	0	4.4034	2.8546	1.8556	0
protein-synthesizing GTPase OS=Bombus vosnesenskii OX=207650 GN=LOC117238512 PE=4 SV=1	A0A6J3L274	51 kDa	3.0877	1.0847	0	0.98952	2.2017	5.7092	1.8556	0.96284
L-lactate dehydrogenase OS=Bombus vosnesenskii OX=207650 GN=LOC117235077 PE=3 SV=1	A0A6J3KHP1 (+1)	36 kDa	7.2047	0	2.9408	1.979	3.3025	0	1.8556	0
heat shock protein 60A OS=Bombus vosnesenskii OX=207650 GN=LOC117242836 PE=3 SV=1	A0A6J3LKG2	60 kDa	1.0292	0	0.98028	0.98952	1.1008	7.6123	0.92779	0.96284
uncharacterized protein LOC117235992 isoform X1 OS=Bombus vosnesenskii OX=207650 GN=LOC117235992 PE=3 SV=1	A0A6J3KMN2	96 kDa	5.1462	2.1693	0	2.9686	2.2017	0.95154	0.92779	1.9257
adrenodoxin-like protein 2, mitochondrial OS=Bombus vosnesenskii OX=207650 GN=LOC117239011 PE=4 SV=1	A0A6J3L3E5 (+14)	18 kDa	0	2.1693	1.9606	1.979	0	3.8061	3.7112	1.9257

protein CREG1 OS=Bombus vosnesenskii OX=207650 GN=LOC117241680 PE=4 SV=1	A0A6J3LED1	33 kDa	0	1.0847	0.98028	1.979	0	1.9031	1.8556	5.777
glucose dehydrogenase [FAD, quinone]-like OS=Bombus vosnesenskii OX=207650 GN=LOC117235983 PE=3 SV=1	A0A6J3KMI4	71 kDa	5.1462	0	4.9014	0	2.2017	1.9031	0.92779	0
ras-like GTP-binding protein Rho1 isoform X1 OS=Bombus vosnesenskii OX=207650 GN=LOC117239208 PE=4 SV=1	A0A6J3L5I5 (+2)	25 kDa	1.0292	2.1693	0.98028	1.979	1.1008	0.95154	1.8556	4.8142
uncharacterized protein LOC117238289 OS=Bombus vosnesenskii OX=207650 GN=LOC117238289 PE=4 SV=1	A0A6J3L041	48 kDa	1.0292	1.0847	0.98028	1.979	4.4034	1.9031	0.92779	1.9257
lysosomal alpha-glucosidase-like OS=Bombus vosnesenskii OX=207650 GN=LOC117237021 PE=3 SV=1	A0A6J3KXG1	115 kDa	1.0292	2.1693	0.98028	0	1.1008	0.95154	3.7112	1.9257
Tubulin beta chain OS=Bombus vosnesenskii OX=207650 GN=LOC117232017 PE=3 SV=1	A0A6J3K442 (+1)	50 kDa	1.0292	0	0.98028	0.98952	1.1008	6.6608	0.92779	1.9257
Glucosidase 2 subunit beta OS=Bombus vosnesenskii OX=207650 GN=LOC117236854 PE=4 SV=1	A0A6J3KSN1	61 kDa	0	3.254	1.9606	2.9686	2.2017	2.8546	1.8556	0
cytochrome P450 9e2-like OS=Bombus vosnesenskii OX=207650 GN=LOC117243321 PE=3 SV=1	A0A6J3LPB8	61 kDa	0	3.254	2.9408	1.979	1.1008	2.8546	1.8556	0
endoplasmic reticulum junction formation protein lunapark-A isoform X2 OS=Bombus vosnesenskii OX=207650 GN=LOC117237258 PE=4 SV=1	A0A6J3KU64 (+1)	67 kDa	0	1.0847	1.9606	1.979	0	5.7092	0.92779	2.8885
acetyl-coenzyme A synthetase OS=Bombus vosnesenskii OX=207650 GN=LOC117242149 PE=3 SV=1	A0A6J3LKQ3	77 kDa	0	2.1693	1.9606	0.98952	2.2017	2.8546	2.7834	0

peptidylprolyl isomerase OS=Bombus vosnesenskii OX=207650 GN=LOC117233209 PE=4 SV=1	A0A6J3K7X6 (+1)	26 kDa	0	2.1693	0.98028	1.979	0	2.8546	2.7834	2.8885
uncharacterized protein LOC117236272 OS=Bombus vosnesenskii OX=207650 GN=LOC117236272 PE=4 SV=1	A0A6J3KS91	34 kDa	1.0292	2.1693	0.98028	0.98952	3.3025	1.9031	1.8556	1.9257
RNA-binding protein squid isoform X5 OS=Bombus vosnesenskii OX=207650 GN=LOC117242918 PE=4 SV=1	A0A6J3LK38 (+7)	34 kDa	2.0585	2.1693	2.9408	1.979	0	0.95154	0.92779	2.8885
inactive selenide, water dikinase-like protein OS=Bombus vosnesenskii OX=207650 GN=LOC117242254 PE=4 SV=1	A0A6J3LHE5	45 kDa	0	1.0847	1.9606	1.979	1.1008	1.9031	3.7112	1.9257
adipocyte plasma membrane-associated protein-like OS=Bombus vosnesenskii OX=207650 GN=LOC117236667 PE=3 SV=1	A0A6J3KV00	63 kDa	5.1462	1.0847	1.9606	0.98952	2.2017	0.95154	0	0.96284
Sterol carrier protein 2 OS=Bombus vosnesenskii OX=207650 GN=LOC117236065 PE=3 SV=1	A0A6J3KNF4	59 kDa	0	2.1693	0.98028	1.979	0	5.7092	1.8556	0.96284
cadherin-99C isoform X1 OS=Bombus vosnesenskii OX=207650 GN=LOC117230454 PE=4 SV=1	A0A6J3JST9 (+1)	174 kDa	0	1.0847	0.98028	2.9686	0	2.8546	2.7834	2.8885
neurexin-4 OS=Bombus vosnesenskii OX=207650 GN=LOC117232677 PE=3 SV=1	A0A6J3K4S8	143 kDa	3.0877	1.0847	0.98028	0.98952	5.5042	2.8546	0	0
protein disulfide-isomerase OS=Bombus vosnesenskii OX=207650 GN=LOC117233733 PE=3 SV=1	A0A6J3KAQ0	47 kDa	0	2.1693	0	0.98952	0	5.7092	1.8556	2.8885
Pyruvate dehydrogenase E1 component subunit alpha OS=Bombus vosnesenskii OX=207650	A0A6J3LEK4 (+1)	44 kDa	0	4.3386	1.9606	0.98952	0	4.7577	1.8556	0

GN=LOC117241718 PE=4 SV=1										
Electron transfer flavoprotein subunit beta OS=Bombus vosnesenskii OX=207650 GN=LOC117231261 PE=3 SV=1	A0A6J3JYP7	28 kDa	8.2339	0	1.9606	0	1.1008	1.9031	0.92779	0
macro domain-containing protein CT2219-like isoform X1 OS=Bombus vosnesenskii OX=207650 GN=LOC117236147 PE=4 SV=1	A0A6J3KSC0 (+1)	30 kDa	0	0	1.9606	2.9686	0	2.8546	0	4.8142
Pyruvate dehydrogenase E1 component subunit beta OS=Bombus vosnesenskii OX=207650 GN=LOC117237770 PE=4 SV=1	A0A6J3KX72	38 kDa	0	4.3386	0	0.98952	0	3.8061	4.639	0
probable tRNA (Guanine(26)-N(2))-dimethyltransferase OS=Bombus vosnesenskii OX=207650 GN=LOC117240164 PE=3 SV=1	A0A6J3L9F3	93 kDa	1.0292	3.254	1.9606	0.98952	1.1008	1.9031	0.92779	0.96284
Beta-galactosidase OS=Bombus vosnesenskii OX=207650 GN=LOC117242768 PE=3 SV=1	A0A6J3LLJ1	73 kDa	2.0585	1.0847	1.9606	0.98952	2.2017	0.95154	2.7834	0.96284
Electron transfer flavoprotein subunit alpha OS=Bombus vosnesenskii OX=207650 GN=LOC117237998 PE=3 SV=1	A0A6J3KZ37	35 kDa	4.117	1.0847	1.9606	0.98952	1.1008	1.9031	1.8556	0
NADH dehydrogenase [ubiquinone] flavoprotein 2, mitochondrial OS=Bombus vosnesenskii OX=207650 GN=LOC117240013 PE=3 SV=1	A0A6J3L8U0	27 kDa	2.0585	1.0847	1.9606	0	1.1008	3.8061	1.8556	0.96284
glutamic acid-rich protein-like OS=Bombus vosnesenskii OX=207650 GN=LOC117238541 PE=4 SV=1	A0A6J3L2E4	87 kDa	0	1.0847	2.9408	1.979	2.2017	2.8546	0.92779	0.96284
trypsin Blo t 3-like OS=Bombus vosnesenskii OX=207650 GN=LOC117230480 PE=4 SV=1	A0A6J3JUX1	28 kDa	0	1.0847	0.98028	1.979	1.1008	0.95154	0	4.8142

Guanine deaminase OS=Bombus vosnesenskii OX=207650 GN=LOC117234830 PE=3 SV=1	A0A6J3KGM5 (+3)	48 kDa	0	1.0847	1.9606	0.98952	4.4034	2.8546	0.92779	0
gamma-glutamylcyclotransferase OS=Bombus vosnesenskii OX=207650 GN=LOC117243262 PE=4 SV=1	A0A6J3LLX0	20 kDa	2.0585	0	1.9606	0	3.3025	1.9031	0	1.9257
phospholipase A1 OS=Bombus vosnesenskii OX=207650 GN=LOC117231169 PE=3 SV=1	A0A6J3JWI4	32 kDa	0	0	2.9408	0.98952	4.4034	0.95154	0	2.8885
Succinate dehydrogenase [ubiquinone] flavoprotein subunit, mitochondrial OS=Bombus vosnesenskii OX=207650 GN=LOC117237512 PE=3 SV=1	A0A6J3KWV8	72 kDa	1.0292	0	2.9408	0.98952	0	5.7092	1.8556	0
alpha-N-acetylglucosaminidase OS=Bombus vosnesenskii OX=207650 GN=LOC117232735 PE=4 SV=1	A0A6J3K6D3	89 kDa	1.0292	1.0847	0.98028	0.98952	2.2017	1.9031	1.8556	0.96284
ectonucleoside triphosphate diphosphohydrolase 5 isoform X3 OS=Bombus vosnesenskii OX=207650 GN=LOC117240647 PE=3 SV=1	A0A6J3LB33 (+2)	52 kDa	1.0292	2.1693	0.98028	0.98952	2.2017	2.8546	0.92779	0
Regucalcin OS=Bombus vosnesenskii OX=207650 GN=LOC117236559 PE=3 SV=1	A0A6J3KR45	34 kDa	0	2.1693	1.9606	0.98952	2.2017	0.95154	2.7834	0.96284
vesicular integral-membrane protein VIP36 OS=Bombus vosnesenskii OX=207650 GN=LOC117230739 PE=4 SV=1	A0A6J3JVV6	37 kDa	3.0877	1.0847	0	0.98952	3.3025	0.95154	1.8556	0.96284
circadian clock-controlled protein-like OS=Bombus vosnesenskii OX=207650 GN=LOC117234046 PE=4 SV=1	A0A6J3KEJ0	28 kDa	2.0585	0	0.98028	0.98952	2.2017	1.9031	0.92779	2.8885
apoptosis-inducing factor 1, mitochondrial OS=Bombus vosnesenskii OX=207650	A0A6J3KDD4	75 kDa	0	1.0847	2.9408	1.979	0	1.9031	0.92779	2.8885

GN=LOC117234260 PE=3 SV=1										
UPF0160 protein MYG1, mitochondrial isoform X2 OS=Bombus vosnesenskii OX=207650 GN=LOC117237639 PE=3 SV=1	A0A6J3KXK7 (+1)	38 kDa	0	0	3.9211	0.98952	2.2017	1.9031	1.8556	0.96284
Eukaryotic translation initiation factor 2 subunit 1 OS=Bombus vosnesenskii OX=207650 GN=LOC117239492 PE=3 SV=1	A0A6J3L8Q9	38 kDa	4.117	1.0847	0.98028	0	2.2017	0.95154	0	0.96284
Ferritin OS=Bombus vosnesenskii OX=207650 GN=LOC117236772 PE=3 SV=1	A0A6J3KS72	22 kDa	1.0292	0	0.98028	1.979	4.4034	2.8546	0.92779	0
uncharacterized protein LOC117232587 OS=Bombus vosnesenskii OX=207650 GN=LOC117232587 PE=4 SV=1	A0A6J3K615	16 kDa	0	0	0.98028	1.979	5.5042	1.9031	0.92779	0
Cytochrome c oxidase subunit OS=Bombus vosnesenskii OX=207650 GN=LOC117233989 PE=3 SV=1	A0A6J3KC57	11 kDa	0	0	0	2.9686	0	2.8546	1.8556	0.96284
uncharacterized protein LOC117233786 OS=Bombus vosnesenskii OX=207650 GN=LOC117233786 PE=4 SV=1	A0A6J3KBU7	49 kDa	0	0	2.9408	0	1.1008	3.8061	1.8556	0
palmitoyl-protein hydrolase OS=Bombus vosnesenskii OX=207650 GN=LOC117232656 PE=3 SV=1	A0A6J3K7B9	26 kDa	1.0292	1.0847	0.98028	0.98952	2.2017	1.9031	1.8556	0.96284
protein draper isoform X1 OS=Bombus vosnesenskii OX=207650 GN=LOC117230808 PE=4 SV=1	A0A6J3JWP7 (+2)	110 kDa	1.0292	3.254	0.98028	0.98952	2.2017	0.95154	0	0.96284
uncharacterized protein LOC117239134 isoform X4 OS=Bombus vosnesenskii OX=207650 GN=LOC117239134 PE=4 SV=1	A0A6J3L8S6	146 kDa	1.0292	1.0847	0.98028	1.979	3.3025	0.95154	0.92779	0
prenylcysteine oxidase-like isoform X2 OS=Bombus	A0A6J3K5A7 (+1)	55 kDa	0	1.0847	0.98028	0.98952	3.3025	0.95154	0.92779	1.9257

vosnesenskii OX=207650 GN=LOC117232243 PE=3 SV=1										
tr|A0A6J3LHV5|A0A6J3LHV5_9HYME-DECOY	tr|A0A6J3LHV5|A0A6J3LHV5_9HYME-DECOY (+1)	?	0	2.1693	1.9606	0	1.1008	0	0	1.9257
twinfilin isoform X3 OS=Bombus vosnesenskii OX=207650 GN=LOC117242612 PE=3 SV=1	A0A6J3LIH0 (+3)	39 kDa	0	1.0847	1.9606	0.98952	0	1.9031	1.8556	1.9257
glucose dehydrogenase [FAD, quinone]-like isoform X2 OS=Bombus vosnesenskii OX=207650 GN=LOC117231080 PE=3 SV=1	A0A6J3JY52 (+1)	71 kDa	0	2.1693	0	0	3.3025	0.95154	2.7834	1.9257
Alpha-mannosidase OS=Bombus vosnesenskii OX=207650 GN=LOC117242330 PE=3 SV=1	A0A6J3LHP8	126 kDa	0	0	1.9606	2.9686	0	2.8546	1.8556	0
maleylacetoacetate isomerase OS=Bombus vosnesenskii OX=207650 GN=LOC117231068 PE=3 SV=1	A0A6J3JW20 (+1)	25 kDa	1.0292	1.0847	0.98028	0.98952	1.1008	1.9031	1.8556	0.96284
histidine triad nucleotide-binding protein 1 OS=Bombus vosnesenskii OX=207650 GN=LOC117231718 PE=4 SV=1	A0A6J3K193	18 kDa	2.0585	1.0847	1.9606	0.98952	2.2017	0.95154	0.92779	0
transport and Golgi organization 2 homolog OS=Bombus vosnesenskii OX=207650 GN=LOC117242023 PE=4 SV=1	A0A6J3LK30	31 kDa	1.0292	2.1693	0.98028	0.98952	3.3025	0	0.92779	0.96284
beta-N-acetylhexosaminidase OS=Bombus vosnesenskii OX=207650 GN=LOC117233990 PE=3 SV=1	A0A6J3KFB2 (+3)	78 kDa	0	1.0847	1.9606	0.98952	2.2017	0.95154	1.8556	0.96284
Glycine cleavage system P protein OS=Bombus vosnesenskii OX=207650 GN=LOC117236441 PE=3 SV=1	A0A6J3KPQ4 (+1)	108 kDa	1.0292	1.0847	0.98028	0	1.1008	1.9031	1.8556	0.96284
insulin-degrading enzyme isoform X1 OS=Bombus vosnesenskii OX=207650	A0A6J3L959 (+1)	116 kDa	1.0292	1.0847	0.98028	0	3.3025	1.9031	1.8556	0

GN=LOC117240265 PE=3 SV=1										
Mitochondrial import inner membrane translocase subunit OS=Bombus vosnesenskii OX=207650 GN=LOC117235971 PE=3 SV=1	A0A6J3KQM1	10 kDa	0	1.0847	0.98028	0.98952	0	0.95154	2.7834	0.96284
prostatic acid phosphatase-like OS=Bombus vosnesenskii OX=207650 GN=LOC117243201 PE=4 SV=1	A0A6J3LLU4	49 kDa	0	2.1693	0	0.98952	1.1008	1.9031	1.8556	1.9257
calmodulin OS=Bombus vosnesenskii OX=207650 GN=LOC117241777 PE=4 SV=1	A0A6J3LEZ5	17 kDa	3.0877	0	0	0.98952	0	0.95154	0	2.8885
ras-like protein 2 isoform X1 OS=Bombus vosnesenskii OX=207650 GN=LOC117236480 PE=4 SV=1	A0A6J3KU39	23 kDa	1.0292	1.0847	1.9606	0.98952	0	0	0	4.8142
carboxypeptidase D-like OS=Bombus vosnesenskii OX=207650 GN=LOC117234262 PE=3 SV=1	A0A6J3KFR7	190 kDa	0	1.0847	0	0.98952	0	1.9031	1.8556	2.8885
uncharacterized protein LOC117230646 OS=Bombus vosnesenskii OX=207650 GN=LOC117230646 PE=4 SV=1	A0A6J3JUZ3	26 kDa	2.0585	0	3.9211	0	2.2017	0	0	1.9257
programmed cell death protein 6 isoform X2 OS=Bombus vosnesenskii OX=207650 GN=LOC117236771 PE=4 SV=1	A0A6J3KVK6	21 kDa	2.0585	0	0.98028	0	3.3025	1.9031	0	0
phospholipase A1 OS=Bombus vosnesenskii OX=207650 GN=LOC117234586 PE=3 SV=1	A0A6J3KG28	34 kDa	0	0	0	1.979	0	0	0	7.7027
Glycogen debranching enzyme OS=Bombus vosnesenskii OX=207650 GN=LOC117232264 PE=3 SV=1	A0A6J3K5E9	183 kDa	0	0	0	0	0	8.5638	0.92779	0
serine protease inhibitor 3-like isoform X1 OS=Bombus vosnesenskii OX=207650	A0A6J3LCG0 (+1)	9 kDa	0	1.0847	0.98028	0.98952	1.1008	1.9031	0.92779	1.9257

GN=LOC117241144 PE=3 SV=1										
deaminated glutathione amidase-like isoform X1 OS=Bombus vosnesenskii OX=207650 GN=LOC117238137 PE=4 SV=1	A0A6J3KZ94	64 kDa	1.0292	1.0847	0.98028	0.98952	0	0.95154	1.8556	1.9257
Carboxylic ester hydrolase OS=Bombus vosnesenskii OX=207650 GN=LOC117235060 PE=3 SV=1	A0A6J3KK13	58 kDa	1.0292	1.0847	0.98028	0.98952	2.2017	0	1.8556	0.96284
sp|ALBU_HUMAN|	sp|ALBU_HUMAN|	69 kDa	2.0585	2.1693	0.98028	0	0	0.95154	0.92779	0.96284
acetyl-CoA acetyltransferase, mitochondrial isoform X1 OS=Bombus vosnesenskii OX=207650 GN=LOC117237979 PE=3 SV=1	A0A6J3KYZ6 (+1)	55 kDa	0	1.0847	0	0.98952	2.2017	1.9031	0.92779	0
Beta-glucuronidase OS=Bombus vosnesenskii OX=207650 GN=LOC117234039 PE=3 SV=1	A0A6J3KEI2	77 kDa	0	2.1693	0	1.979	0	1.9031	0.92779	0.96284
Calcium-transporting ATPase OS=Bombus vosnesenskii OX=207650 GN=LOC117242425 PE=3 SV=1	A0A6J3LHD7 (+9)	138 kDa	0	0	0.98028	0	3.3025	2.8546	0.92779	0.96284
ras-related protein Rab-1A OS=Bombus vosnesenskii OX=207650 GN=LOC117239216 PE=4 SV=1	A0A6J3L7L5	23 kDa	1.0292	0	0	1.979	1.1008	0	1.8556	2.8885
Cytochrome b-c1 complex subunit Rieske, mitochondrial OS=Bombus vosnesenskii OX=207650 GN=LOC117234293 PE=3 SV=1	A0A6J3KDL9 (+1)	30 kDa	4.117	1.0847	1.9606	0	0	0	0.92779	0.96284
serine hydrolase-like protein OS=Bombus vosnesenskii OX=207650 GN=LOC117233019 PE=4 SV=1	A0A6J3K6R3	34 kDa	0	0	0.98028	0.98952	0	3.8061	1.8556	0.96284
Protein quiver OS=Bombus vosnesenskii OX=207650	A0A6J3KT08	55 kDa	1.0292	0	2.9408	0	0	0	0.92779	2.8885

GN=LOC117236921 PE=3 SV=1										
Multiple inositol polyphosphate phosphatase 1 OS=Bombus vosnesenskii OX=207650 GN=LOC117243022 PE=3 SV=1	A0A6J3LLI7	55 kDa	0	0	0.98028	0	0	1.9031	0.92779	4.8142
methanethiol oxidase OS=Bombus vosnesenskii OX=207650 GN=LOC117243436 PE=3 SV=1	A0A6J3LNT8	53 kDa	0	3.254	0.98028	4.9476	0	0	0	0
ADP/ATP translocase OS=Bombus vosnesenskii OX=207650 GN=LOC117233403 PE=3 SV=1	A0A6J3KAQ1	33 kDa	2.0585	0	0	0	0	5.7092	0	0.96284
fatty acid synthase OS=Bombus vosnesenskii OX=207650 GN=LOC117234004 PE=4 SV=1	A0A6J3KC82	262 kDa	0	0	0	0	1.1008	6.6608	0	0
protein bark beetle OS=Bombus vosnesenskii OX=207650 GN=LOC117235781 PE=4 SV=1	A0A6J3KPG1	331 kDa	1.0292	1.0847	0.98028	0	1.1008	0.95154	0.92779	1.9257
scoloptoxin SSD14-like isoform X3 OS=Bombus vosnesenskii OX=207650 GN=LOC117239721 PE=4 SV=1	A0A6J3L709 (+3)	73 kDa	1.0292	1.0847	0	0.98952	1.1008	1.9031	0	0.96284
sulfide:quinone oxidoreductase, mitochondrial OS=Bombus vosnesenskii OX=207650 GN=LOC117243089 PE=4 SV=1	A0A6J3LLV0	51 kDa	1.0292	0	0.98028	0	1.1008	1.9031	0.92779	0.96284
uncharacterized protein LOC117240659 OS=Bombus vosnesenskii OX=207650 GN=LOC117240659 PE=4 SV=1	A0A6J3LFA9	27 kDa	1.0292	1.0847	0.98028	0.98952	0	0.95154	1.8556	0.96284
uncharacterized protein LOC117233336 OS=Bombus vosnesenskii OX=207650 GN=LOC117233336 PE=4 SV=1	A0A6J3K8M3	44 kDa	1.0292	0	0.98028	0.98952	0	1.9031	0.92779	0.96284
protein MEMO1 OS=Bombus vosnesenskii OX=207650 GN=LOC117231190 PE=3 SV=1	A0A6J3JYQ6	33 kDa	0	0	0.98028	0.98952	3.3025	0.95154	0.92779	0.96284

Tetraspanin OS=Bombus vosnesenskii OX=207650 GN=LOC117236900 PE=3 SV=1	A0A6J3KS54	26 kDa	1.0292	2.1693	0.98028	0.98952	0	0.95154	1.8556	0
Probable methylthioribulose-1-phosphate dehydratase OS=Bombus vosnesenskii OX=207650 GN=LOC117236782 PE=3 SV=1	A0A6J3KS65	27 kDa	0	1.0847	1.9606	0.98952	0	1.9031	1.8556	0
Regucalcin OS=Bombus vosnesenskii OX=207650 GN=LOC117236558 PE=3 SV=1	A0A6J3KQD8	37 kDa	0	0	0.98028	0.98952	0	1.9031	1.8556	1.9257
ovalbumin-related protein X-like isoform X2 OS=Bombus vosnesenskii OX=207650 GN=LOC117231310 PE=3 SV=1	A0A6J3JXH0 (+1)	45 kDa	3.0877	0	0.98028	0	2.2017	0	0.92779	0.96284
Coatomer subunit alpha OS=Bombus vosnesenskii OX=207650 GN=LOC117234785 PE=4 SV=1	A0A6J3KIV8 (+1)	138 kDa	0	2.1693	1.9606	1.979	0	0	0.92779	0.96284
leukocyte elastase inhibitor OS=Bombus vosnesenskii OX=207650 GN=LOC117238287 PE=3 SV=1	A0A6J3L036	76 kDa	4.117	1.0847	0	0.98952	1.1008	0	0	0.96284
Sugar phosphate phosphatase OS=Bombus vosnesenskii OX=207650 GN=LOC117240022 PE=3 SV=1	A0A6J3LAT7	53 kDa	0	1.0847	0	1.979	0	1.9031	0	1.9257
uncharacterized protein LOC117239581 isoform X2 OS=Bombus vosnesenskii OX=207650 GN=LOC117239581 PE=4 SV=1	A0A6J3LB71	75 kDa	0	0	0.98028	0	0	1.9031	2.7834	1.9257
mitochondrial-processing peptidase subunit beta OS=Bombus vosnesenskii OX=207650 GN=LOC117238235 PE=3 SV=1	A0A6J3KZV1	53 kDa	0	0	0	0.98952	0	4.7577	0.92779	0.96284
Glucosamine-6-phosphate isomerase OS=Bombus vosnesenskii OX=207650	A0A6J3KDJ3	32 kDa	0	1.0847	0	0.98952	0	4.7577	0	0.96284

GN=LOC117234284 PE=3 SV=1										
uncharacterized protein LOC117237750 OS=Bombus vosnesenskii OX=207650 GN=LOC117237750 PE=4 SV=1	A0A6J3L1E8	496 kDa	5.1462	1.0847	0	0	1.1008	0.95154	0	0
papilin isoform X1 OS=Bombus vosnesenskii OX=207650 GN=LOC117236618 PE=4 SV=1	A0A6J3KQN2 (+9)	320 kDa	0	2.1693	0	0	0	1.9031	0	0.96284
Profilin OS=Bombus vosnesenskii OX=207650 GN=LOC117239432 PE=3 SV=1	A0A6J3L6I3	14 kDa	0	0	3.9211	0	0	0.95154	0	2.8885
ras-related protein Rab-2 OS=Bombus vosnesenskii OX=207650 GN=LOC117238882 PE=4 SV=1	A0A6J3L405	24 kDa	0	0	0	0.98952	0	0.95154	0	5.777
phosphatidylinositol-binding clathrin assembly protein unc-11 isoform X3 OS=Bombus vosnesenskii OX=207650 GN=LOC117242523 PE=3 SV=1	A0A6J3LHZ3 (+23)	96 kDa	1.0292	0	0	1.979	0	0.95154	0.92779	0.96284
long-chain fatty acid transport protein 1-like OS=Bombus vosnesenskii OX=207650 GN=LOC117242805 PE=3 SV=1	A0A6J3LK79	73 kDa	1.0292	1.0847	0.98028	0.98952	0	0.95154	1.8556	0
oligoribonuclease-like isoform X2 OS=Bombus vosnesenskii OX=207650 GN=LOC117236508 PE=3 SV=1	A0A6J3KU90 (+1)	22 kDa	0	0	0.98028	0.98952	2.2017	0.95154	0.92779	0
Eukaryotic translation initiation factor 3 subunit I OS=Bombus vosnesenskii OX=207650 GN=LOC117235236 PE=3 SV=1	A0A6J3KL31	37 kDa	0	0	0.98028	0.98952	1.1008	0.95154	0.92779	1.9257
lamin Dm0-like isoform X1 OS=Bombus vosnesenskii OX=207650 GN=LOC117232242 PE=3 SV=1	A0A6J3K4F0 (+1)	69 kDa	2.0585	0	0.98028	0	2.2017	0.95154	0	0.96284
uncharacterized protein LOC117236114 OS=Bombus vosnesenskii OX=207650	A0A6J3KNR7	44 kDa	0	0	0.98028	0	1.1008	1.9031	0.92779	1.9257

GN=LOC117236114 PE=4 SV=1										
3-hydroxyacyl-CoA dehydrogenase type-2 OS=Bombus vosnesenskii OX=207650 GN=LOC117236101 PE=3 SV=1	A0A6J3KN53	27 kDa	0	0	1.9606	0.98952	0	1.9031	0.92779	0.96284
Golgi phosphoprotein 3 homolog sauron OS=Bombus vosnesenskii OX=207650 GN=LOC117240023 PE=3 SV=1	A0A6J3L8W4	34 kDa	0	1.0847	0	0.98952	1.1008	2.8546	0	0.96284
fibrillin-2-like isoform X1 OS=Bombus vosnesenskii OX=207650 GN=LOC117240054 PE=4 SV=1	A0A6J3L981	177 kDa	0	1.0847	0.98028	0	0	2.8546	0.92779	0.96284
extracellular serine/threonine protein CG31145 isoform X1 OS=Bombus vosnesenskii OX=207650 GN=LOC117242970 PE=3 SV=1	A0A6J3LPP3	59 kDa	1.0292	0	2.9408	0	0	0.95154	0.92779	0
zinc finger RNA-binding protein isoform X1 OS=Bombus vosnesenskii OX=207650 GN=LOC117241931 PE=4 SV=1	A0A6J3LFX9 (+1)	109 kDa	1.0292	0	0	0	2.2017	0	0.92779	1.9257
Aminopeptidase OS=Bombus vosnesenskii OX=207650 GN=LOC117241474 PE=3 SV=1	A0A6J3LCH3 (+3)	112 kDa	0	0	0.98028	0	1.1008	1.9031	1.8556	0
chymotrypsin-1-like OS=Bombus vosnesenskii OX=207650 GN=LOC117241961 PE=4 SV=1	A0A6J3LJU9	33 kDa	1.0292	0	0	0.98952	0	0	0	4.8142
glutathione hydrolase 1 proenzyme-like OS=Bombus vosnesenskii OX=207650 GN=LOC117230952 PE=4 SV=1	A0A6J3JWY5	63 kDa	1.0292	1.0847	0	0	1.1008	0.95154	1.8556	0
protein takeout-like OS=Bombus vosnesenskii OX=207650 GN=LOC117232781 PE=4 SV=1	A0A6J3K5E3	28 kDa	1.0292	0	0.98028	0.98952	0	1.9031	0	0.96284
S-(hydroxymethyl)glutathione dehydrogenase OS=Bombus	A0A6J3LEG5	41 kDa	0	1.0847	0.98028	0	1.1008	1.9031	0	0.96284

vosnesenskii OX=207650 GN=LOC117240382 PE=3 SV=1										
pyrimidodiazepine synthase-like OS=Bombus vosnesenskii OX=207650 GN=LOC117236127 PE=3 SV=1	A0A6J3KN94	28 kDa	0	1.0847	0	0	0	1.9031	1.8556	0.96284
Signal transducer and activator of transcription OS=Bombus vosnesenskii OX=207650 GN=LOC117230840 PE=3 SV=1	A0A6J3JV29 (+1)	88 kDa	2.0585	1.0847	0	0	0	1.9031	0	0.96284
Polypeptide N-acetylgalactosaminyltransferase OS=Bombus vosnesenskii OX=207650 GN=LOC117238410 PE=3 SV=1	A0A6J3L406	68 kDa	0	0	1.9606	0.98952	1.1008	0	0	1.9257
troponin T isoform X1 OS=Bombus vosnesenskii OX=207650 GN=LOC117233281 PE=3 SV=1	A0A6J3K890 (+12)	52 kDa	0	0	0	0.98952	0	1.9031	0	0.96284
TRPL translocation defect protein 14 isoform X8 OS=Bombus vosnesenskii OX=207650 GN=LOC117235482 PE=4 SV=1	A0A6J3KJU5 (+5)	54 kDa	0	0	0.98028	0	0	1.9031	0	2.8885
threonine--tRNA ligase OS=Bombus vosnesenskii OX=207650 GN=LOC117234964 PE=3 SV=1	A0A6J3KKT2 (+1)	83 kDa	0	0	0.98028	0	2.2017	1.9031	0	0
dnaJ protein homolog 1-like OS=Bombus vosnesenskii OX=207650 GN=LOC117238783 PE=4 SV=1	A0A6J3L778	39 kDa	0	0	0.98028	0	0	2.8546	0	1.9257
protein 5NUC-like OS=Bombus vosnesenskii OX=207650 GN=LOC117243346 PE=3 SV=1	A0A6J3LNA0	68 kDa	3.0877	0	0	0	2.2017	0	0.92779	0
40S ribosomal protein S3-like OS=Bombus vosnesenskii OX=207650 GN=LOC117236727 PE=3 SV=1	A0A6J3KRX5	27 kDa	1.0292	0	0.98028	0	0	3.8061	0	0

very-long-chain 3-oxoacyl-CoA reductase-like OS=Bombus vosnesenskii OX=207650 GN=LOC117233106 PE=3 SV=1	A0A6J3K7B3	36 kDa	0	0	0.98028	0	1.1008	2.8546	0	0
muscle M-line assembly protein unc-89-like isoform X1 OS=Bombus vosnesenskii OX=207650 GN=LOC117242037 PE=4 SV=1	A0A6J3LHW6 (+1)	230 kDa	3.0877	0	0	0	2.2017	0	0	0
ornithine aminotransferase OS=Bombus vosnesenskii OX=207650 GN=LOC117230575 PE=3 SV=1	A0A6J3JUN1	87 kDa	1.0292	1.0847	0	0	0	0.95154	1.8556	0
prion-like-(Q/N-rich) domain-bearing protein 25 OS=Bombus vosnesenskii OX=207650 GN=LOC117240088 PE=4 SV=1	A0A6J3LD52	40 kDa	1.0292	2.1693	0	0.98952	0	0	0.92779	0
sphingomyelin phosphodiesterase isoform X2 OS=Bombus vosnesenskii OX=207650 GN=LOC117233533 PE=3 SV=1	A0A6J3KAS5 (+2)	82 kDa	0	1.0847	0	0.98952	0	1.9031	0.92779	0
Tripeptidyl-peptidase 2 OS=Bombus vosnesenskii OX=207650 GN=LOC117243560 PE=3 SV=1	A0A6J3LNJ0	254 kDa	1.0292	1.0847	0	0	1.1008	1.9031	0	0
GILT-like protein 1 OS=Bombus vosnesenskii OX=207650 GN=LOC117241772 PE=3 SV=1	A0A6J3LEI6	27 kDa	0	0	0.98028	0	0	0.95154	0.92779	1.9257
cAMP-dependent protein kinase type I regulatory subunit isoform X2 OS=Bombus vosnesenskii OX=207650 GN=LOC117238261 PE=3 SV=1	A0A6J3L111 (+2)	36 kDa	0	1.0847	0	0.98952	0	0.95154	0	1.9257
protein phosphatase 1 regulatory subunit 7 isoform X2 OS=Bombus vosnesenskii OX=207650 GN=LOC117235652 PE=4 SV=1	A0A6J3KKS9 (+1)	33 kDa	0	0	0	0.98952	1.1008	0.95154	0	1.9257
dnaJ homolog subfamily C member 5 isoform X1	A0A6J3L9K0 (+1)	28 kDa	2.0585	0	0.98028	0	1.1008	0	0.92779	0

OS=Bombus vosnesenskii OX=207650 GN=LOC117239256 PE=4 SV=1										
tr|A0A6J3KDN0|A0A6J3KDN0_9HYME-DECOY	tr|A0A6J3KDN0|A0A6J3KDN0_9HYME-DECOY	?	0	2.1693	1.9606	0	0	0.95154	0	0
carboxypeptidase B-like OS=Bombus vosnesenskii OX=207650 GN=LOC117243232 PE=3 SV=1	A0A6J3LMI8	48 kDa	0	0	0	0	1.1008	0.95154	0	2.8885
transmembrane protein 256 homolog OS=Bombus vosnesenskii OX=207650 GN=LOC117236681 PE=3 SV=1	A0A6J3KUA9	18 kDa	0	0	0.98028	0	0	2.8546	0.92779	0
nidogen isoform X4 OS=Bombus vosnesenskii OX=207650 GN=LOC117231978 PE=4 SV=1	A0A6J3K1E9 (+1)	144 kDa	0	0	0	0.98952	1.1008	2.8546	0	0
Dihydrolipoamide acetyltransferase component of pyruvate dehydrogenase complex OS=Bombus vosnesenskii OX=207650 GN=LOC117242247 PE=3 SV=1	A0A6J3LGH4	50 kDa	0	0	0	0	0	3.8061	0	0.96284
geranylgeranyl pyrophosphate synthase OS=Bombus vosnesenskii OX=207650 GN=LOC117237044 PE=3 SV=1	A0A6J3KXJ7	36 kDa	0	0	0	0	0	4.7577	0	0
peptidylprolyl isomerase OS=Bombus vosnesenskii OX=207650 GN=LOC117240928 PE=4 SV=1	A0A6J3LG48	40 kDa	0	0	0	0	0	0.95154	1.8556	0.96284
ATP synthase subunit gamma OS=Bombus vosnesenskii OX=207650 GN=LOC117241769 PE=3 SV=1	A0A6J3LDW0	33 kDa	0	2.1693	0.98028	0	0	0.95154	0	0
Histone H4 OS=Bombus vosnesenskii OX=207650 GN=LOC117236733 PE=3 SV=1	A0A6J3KVC8	11 kDa	2.0585	0	0	0	1.1008	0.95154	0	0
Nicotinate phosphoribosyltransferase	A0A6J3JRB7 (+1)	68 kDa	0	0	1.9606	0	1.1008	0	0	0

OS=Bombus vosnesenskii OX=207650 GN=LOC117230233 PE=3 SV=1										
Glutamine synthetase OS=Bombus vosnesenskii OX=207650 GN=LOC117231877 PE=3 SV=1	A0A6J3K0Q0	41 kDa	0	0	0.98028	0	0	2.8546	0	0
protein transport protein Sec24C OS=Bombus vosnesenskii OX=207650 GN=LOC117232139 PE=3 SV=1	A0A6J3K292	140 kDa	0	0	0	0.98952	0	2.8546	0	0
Fatty acyl-CoA reductase OS=Bombus vosnesenskii OX=207650 GN=LOC117240703 PE=3 SV=1	A0A6J3LBA6	58 kDa	0	0	0	0	0	2.8546	0	0
Alpha-amylase OS=Bombus vosnesenskii OX=207650 GN=LOC117233531 PE=3 SV=1	A0A6J3KCR0	56 kDa	0	0	0	0	0	0	0	3.8514
Obg-like ATPase 1 OS=Bombus vosnesenskii OX=207650 GN=LOC117237974 PE=3 SV=1	A0A6J3L2I8 (+1)	45 kDa	0	0	0	0	0	1.9031	0	0.96284
Aminomethyltransferase OS=Bombus vosnesenskii OX=207650 GN=LOC117232872 PE=3 SV=1	A0A6J3K7L4	54 kDa	2.0585	0	0	0	1.1008	0	0	0
Mediator of RNA polymerase II transcription subunit 20 OS=Bombus vosnesenskii OX=207650 GN=LOC117238802 PE=3 SV=1	A0A6J3L2P0 (+1)	55 kDa	0	0	1.9606	0	0	0.95154	0	0
Mannosyl-oligosaccharide glucosidase OS=Bombus vosnesenskii OX=207650 GN=LOC117238616 PE=3 SV=1	A0A6J3L2Q2 (+2)	93 kDa	0	0	0	0	0	0.95154	1.8556	0
glucose dehydrogenase [FAD, quinone]-like OS=Bombus vosnesenskii OX=207650 GN=LOC117237603 PE=3 SV=1	A0A6J3KW60	69 kDa	0	0	1.9606	0	1.1008	0	0	0
dimethylargininase OS=Bombus vosnesenskii OX=207650	A0A6J3KN42 (+1)	32 kDa	0	0	0	0	0	0.95154	0	1.9257

GN=LOC117236004 PE=3 SV=1										
UDP-glucose:glycoprotein glucosyltransferase isoform X4 OS=Bombus vosnesenskii OX=207650 GN=LOC117234455 PE=3 SV=1	A0A6J3KEF9 (+3)	174 kDa	0	0	0.98028	0	0	1.9031	0	0
NADPH:adrenodoxin oxidoreductase, mitochondrial OS=Bombus vosnesenskii OX=207650 GN=LOC117235515 PE=3 SV=1	A0A6J3KKM1	51 kDa	0	0	0.98028	0	0	1.9031	0	0
Glutathione synthetase OS=Bombus vosnesenskii OX=207650 GN=LOC117235755 PE=3 SV=1	A0A6J3KLC7	56 kDa	0	0	0.98028	0	0	1.9031	0	0
Actin-related protein 2/3 complex subunit 5 OS=Bombus vosnesenskii OX=207650 GN=LOC117235940 PE=3 SV=1	A0A6J3KM89	17 kDa	0	0	0.98028	0	0	1.9031	0	0
spectrin alpha chain isoform X1 OS=Bombus vosnesenskii OX=207650 GN=LOC117230437 PE=3 SV=1	A0A6J3JSP6 (+1)	280 kDa	0	2.1693	0	0	0	0.95154	0	0
protein argonaute-2 isoform X1 OS=Bombus vosnesenskii OX=207650 GN=LOC117234637 PE=3 SV=1	A0A6J3KFG6 (+3)	116 kDa	0	0	0	0	0	0	0.92779	1.9257
WD repeat-containing protein 61 isoform X1 OS=Bombus vosnesenskii OX=207650 GN=LOC117240893 PE=4 SV=1	A0A6J3LB17 (+1)	36 kDa	0	0	0	0.98952	0	1.9031	0	0
protein crumbs isoform X2 OS=Bombus vosnesenskii OX=207650 GN=LOC117235346 PE=4 SV=1	A0A6J3KJ11 (+3)	244 kDa	2.0585	0	0	0	0	0	0	0.96284
tr|A0A6J3L4Y3|A0A6J3L4Y3_9HYME-DECOY	tr|A0A6J3L4Y3|A0A6J3L4Y3_9HYME-DECOY	?	0	0	0	0.98952	0	0	0	1.9257
LOW QUALITY PROTEIN: fasciclin-3 OS=Bombus vosnesenskii OX=207650	A0A6J3LDU2	64 kDa	0	0	0	0	0	1.9031	0	0

GN=LOC117241615 PE=4 SV=1										
Delta-1-pyrroline-5-carboxylate synthase OS=Bombus vosnesenskii OX=207650 GN=LOC117236322 PE=3 SV=1	A0A6J3KSI1 (+1)	84 kDa	0	0	0	0	0	2.8546	0	0
Cytochrome c oxidase subunit 4 OS=Bombus vosnesenskii OX=207650 GN=LOC117233324 PE=3 SV=1	A0A6J3K8G3	23 kDa	0	0	0	0	0	2.8546	0	0
Phosphoglycerate kinase OS=Bombus vosnesenskii OX=207650 GN=LOC117239799 PE=3 SV=1	A0A6J3L8H2	45 kDa	0	0	0	0	0	1.9031	0	0
aminopeptidase N OS=Bombus vosnesenskii OX=207650 GN=LOC117242943 PE=3 SV=1	A0A6J3LK60	112 kDa	0	0	0	0	0	1.9031	0	0
talin-1 isoform X3 OS=Bombus vosnesenskii OX=207650 GN=LOC117233591 PE=4 SV=1	A0A6J3KA08 (+4)	264 kDa	0	0	0	0	0	1.9031	0	0
RNA helicase OS=Bombus vosnesenskii OX=207650 GN=LOC117236593 PE=3 SV=1	A0A6J3KQI8	48 kDa	0	0	0	0	0	1.9031	0	0
40S ribosomal protein S3a OS=Bombus vosnesenskii OX=207650 GN=LOC117241712 PE=3 SV=1	A0A6J3LG17	30 kDa	0	0	0	0	0	1.9031	0	0
Endoribonuclease OS=Bombus vosnesenskii OX=207650 GN=LOC117230272 PE=3 SV=1	A0A6J3JRJ6 (+1)	68 kDa	0	0	0	0	0	0	0	1.9257
protein seele OS=Bombus vosnesenskii OX=207650 GN=LOC117234859 PE=3 SV=1	A0A6J3KGP8	23 kDa	0	0	0	0	0	0	0	1.9257
Tubulin alpha chain OS=Bombus vosnesenskii OX=207650 GN=LOC117241679 PE=3 SV=1	A0A6J3LI83 (+1)	50 kDa	0	0	0	0	0	1.9031	0	0
cytochrome b-c1 complex subunit 2, mitochondrial OS=Bombus vosnesenskii	A0A6J3K1E4	47 kDa	0	0	0	0	0	1.9031	0	0

OX=207650 GN=LOC117231957 PE=4 SV=1										
dnaJ homolog subfamily C member 3 OS=Bombus vosnesenskii OX=207650 GN=LOC117234620 PE=4 SV=1	A0A6J3KFL8	55 kDa	0	0	0	0	0	1.9031	0	0
uncharacterized protein LOC117233521 OS=Bombus vosnesenskii OX=207650 GN=LOC117233521 PE=3 SV=1	A0A6J3KAP7 (+2)	117 kDa	0	0	1.9606	0	0	0	0	0

Supplementary Table S4. Homology-based annotation of query sequences using Uniprot ToxProt database

Protein ID	sseqid	pident	E-value	Cov_min	Prediction	sp_prob	cs
transmembrane_protease_serine_9_like	sp|Q7M4I3|SP4_BOMPE	83.5	2.64E-139	0.42	Other	0.000282	
phospholipase_A2	sp|Q7M4I6|PA2_BOMPE	89	2.99E-98	0.7555555556	SP(Sec/SPI)	0.997944	CS pos: 21-22. VHA-FQ. Pr: 0.9867
venom_dipeptidyl_peptidase_4	sp|B2D0J4|VDPP4_APIME	76	0	0.9858611825	SP(Sec/SPI)	0.831621	CS pos: 24-25. VAG-KS. Pr: 0.6202
CLIP_domain_containing_serine_protease	sp|B5U2W0|VSP_BOMIG	69.1	9.25E-198	0.9890710383	Other	0.030719	
inducible_metalloproteinase_inhibitor_protein_like	sp|P0DRJ0|TILM_BOMIG	86.9	1.88E-62	0.9428571429	SP(Sec/SPI)	0.999763	CS pos: 21-22. TSA-AR. Pr: 0.6326
venom_acid_phosphatase_Acph_1_like_isoform_X1	sp|B2D0J4|VDPP4_APIME	76	0	0.9858611825	Other	0.020461	
venom_protease_like	sp|B2D0J4|VDPP4_APIME	76	0	0.9858611825	Other	0.001054	

Hyaluronidase	sp|Q08169|HUGA_APIME	78.5	1.11E-214	0.5	SP(Sec/SPI)	0.886146	CS pos: 17-18. IDA-FF. Pr: 0.8146
Transferrin	sp|K9IMD0|TRLF_DESRO	28.2	1.60E-58	1.028248588	SP(Sec/SPI)	0.968728	CS pos: 25-26. AHS-SE. Pr: 0.6756
melittin_like	sp|P0DXZ4|TX1A_XYLAR	50	7.98E-07	0.9285714286	SP(Sec/SPI)	0.991445	CS pos: 20-21. ITA-VP. Pr: 0.6716
venom_protease_like_isoform_x1	sp|B2D0J4|VDPP4_APIME	76	0	0.9858611825	Other	0.002836	
serine_protease_inhibitor_88Ea_like_isoform_X2	sp|Q9GP13|IRIS_IXORI	29.6	8.29E-44	0.90625	SP(Sec/SPI)	0.99069	CS pos: 21-22. VSA-QC. Pr: 0.7430
venom_serine_protease_34_like	sp|B2D0J4|VDPP4_APIME	76	0	0.9858611825	SP(Sec/SPI)	0.870691	CS pos: 33-34. SNA-AN. Pr: 0.6913
apolipophorins	NA	NA	NA	NA	SP(Sec/SPI)	0.996521	CS pos: 24-25. TES-AS. Pr: 0.4693
CLIP_domain_containing_serine_protease_2	sp|B5U2W0|VSP_BOMIG	69.1	9.25E-198	0.9890710383	Other	0.030719	
Catalase	NA	NA	NA	NA	Other	0.004214	
uncharacterized_protein_LOC117238774	sp|B1P1J3|CYT_CHIGU	33.3	5.18E-09	0.1052036199	SP(Sec/SPI)	0.945979	CS pos: 24-25. VSS-GP. Pr: 0.7864
Thioredoxin	NA	NA	NA	NA	Other	0.001377	
Thioredoxin_like	NA	NA	NA	NA	Other	0.00327	
putative_serine_protease_K12H4.7	NA	NA	NA	NA	SP(Sec/SPI)	0.801134	CS pos: 19-20. SIS-WE. Pr: 0.5637
Phosphotransferase	NA	NA	NA	NA	Other	0.000253	
Aminopeptidase	NA	NA	NA	NA	Other	0.000388	
Peptidyl_prolyl_cis_trans_isomerase	NA	NA	NA	NA	SP(Sec/SPI)	0.99928	CS pos: 20-21. SNA-DD. Pr: 0.8685
plancitoxin_1_isoform_X1	sp|Q75WF2|DNA2_ACAPL	34.6	8.80E-75	0.9330024814	Other	0.002423	
peroxiredoxin_1_like	sp|P0CV91|PRDX4_CROAT	85	1.15E-16	0.2051282051	Other	0.001235	

Dipeptidyl_peptidase_3	NA	NA	NA	NA	Other	0.00075	
ester_hydrolase_C11orf54_homolog	NA	NA	NA	NA	Other	0.00057	
arginine_kinase	NA	NA	NA	NA	Other	0.00067	
Carboxypeptidase	sp|C9WMM5|VCP_APIME	81.4	1.04E-302	1	SP(Sec/SPI)	0.9796	CS pos: 19-20. ARG-FT. Pr: 0.7179
sodium_calcium_exchanger_regulatory_protein_1_isoform_X1	NA	NA	NA	NA	Other	0.000922	
peptidylprolyl_isomerase	NA	NA	NA	NA	Other	0.003039	
peroxiredoxin_like	sp|P0CV91|PRDX4_CROAT	85	1.15E-16	0.2051282051	Other	0.017316	
digestive_cysteine_proteinase_1	NA	NA	NA	NA	SP(Sec/SPI)	0.989531	CS pos: 20-21. IEA-VK. Pr: 0.7905
putative_aminopeptidase_2_isoform_X1	NA	NA	NA	NA	Other	0.485548	
venom_protease_like_isoform_X1	sp|B2D0J4|VDPP4_APIME	76	0	0.9858611825	Other	0.00546	
Carboxypeptidase_Q	sp|C9WMM5|VCP_APIME	81.4	1.04E-302	1	Other	0.002106	
thioredoxin_like_protein_1	NA	NA	NA	NA	Other	0.00327	
Dipeptidase	NA	NA	NA	NA	Other	0.009176	
peroxiredoxin_like	sp|P0CV91|PRDX4_CROAT	85	1.15E-16	0.2051282051	Other	0.017316	
omega_conotoxin_like_protein_1	sp|H9KQJ7|OCLP1_APIME	69.3	7.95E-36	1	SP(Sec/SPI)	0.9995	CS pos: 20-21. ITA VP. Pr: 0.6716
esterase_E4_like	sp|A0A8B0RBM2|ACES_BOMIG	31.5	8.83E-83	0.9240282686	SP(Sec/SPI)	0.527397	CS pos: 30-31. CTA-HP. Pr: 0.2868
matrix_metalloproteinase_14_isoform_X1	NA	NA	NA	NA	SP(Sec/SPI)	0.891226	CS pos: 30-31. VKS-EE. Pr: 0.7540

protein_id Bombus venom protein name (from your FASTA).
sseqid UniProt ID of the best toxin hit found by DIAMOND (subject sequence ID).
pident Percent amino acid identity between Bombus protein and UniProt toxin.

evalue	Statistical significance of the alignment (lower = stronger homology).
cov_min	Minimum sequence coverage between Bombus protein and toxin homolog.
prediction	SignalP-5.0 prediction: SP(Sec/SPI) = secreted signal peptide; OTHER = no signal peptide.
sp_prob	Probability that the protein contains a signal peptide (0ñ1).
cs	Signal peptide cleavage site position and motif (if predicted).

Supplementary Table S5. Putative venom toxins identified in wild and captive *B. vosnesenskii* workers in low- and high-elevation sites with 4 replicates each.

Protein	Accession	Wild population (n = 4/ group)							Captive population (n = 4/ group)				
		Low	High	log2FC	FC	*p*	FDR	Low	High	log2FC	FC	*p*	FDR
**bombolitin-like**	A0A6J3K401	210.9	90.9	-1.498	0.35	**5.32e-05**	**0.00984**	197.9	178.4	-0.058	0.96	0.785	0.964
**Transferrin**	A0A6J3JT60	180.4	101.3	-1.236	0.42	**0.00373**	0.172	212.1	285.9	0.490	1.40	**0.0302**	0.933
**phospholipase A_2**	A0A6J3K1V5	1644.6	1338.6	-0.594	0.66	**0.0227**	0.351	1614.8	1551.3	-0.003	1.00	0.98	0.98
**venom protease-like**	A0A6J3K168	291.6	206.3	-0.728	0.60	**0.0276**	0.351	357.7	413.1	0.268	1.20	0.156	0.933
**Hyaluronidase**	A0A6J3LGZ0	514.0	398.5	-0.605	0.66	**0.0277**	0.351	340.9	348.2	0.084	1.06	0.709	0.933
**CLIP domain-containing serine protease**	A0A6J3KUF5 (+1)	627.0	483.5	-0.632	0.65	**0.0309**	0.351	893.5	942.5	0.134	1.10	0.314	0.933
**Bee-milk protein**	A0A6J3K9U6	91.7	43.4	-1.155	0.45	**0.0462**	0.351	133.4	190.3	0.551	1.47	0.124	0.933
**peroxiredoxin-like**	A0A6J3KBU5	10.1	6.8	-0.822	0.57	**0.0467**	0.351	6.6	6.9	—	—	—	—
**Dipeptidyl peptidase 3**	A0A6J3JVW9	11.2	20.7	0.845	1.80	**0.0475**	0.351	19.5	19.0	-0.024	0.98	0.933	0.972
**transmembrane protease serine 9-like**	A0A6J3K2U7	1498.1	1155.9	-0.610	0.65	0.0588	0.375	2145.7	2438.8	0.254	1.19	0.0785	0.933
**peroxiredoxin 1-like**	A0A6J3L2X1	34.0	23.6	-0.692	0.62	0.0701	0.392	44.5	42.5	-0.016	0.99	0.919	0.972
**venom dipeptidyl peptidase 4**	A0A6J3K7R6	1325.8	1058.1	-0.552	0.68	0.0702	0.392	1129.1	1206.2	0.167	1.12	0.278	0.933
**uncharacterized protein LOC117238774**	A0A6J3L3K9	49.7	35.4	-0.561	0.68	0.15	0.45	118.2	82.7	-0.603	0.66	0.356	0.933

**venom acid phosphatase Acph-1-like isoform X1**	A0A6J3KME8 (+1)	750.7	681.7	-0.392	0.76	0.183	0.484	884.7	716.9	-0.272	0.83	0.249	0.933
**arginine kinase**	A0A6J3JW35	6.5	13.5	0.582	1.50	0.226	0.503	3.3	2.6	—	—	—	—
**ester hydrolase C11orf54 homolog**	A0A6J3L5T9	15.9	14.4	-0.316	0.80	0.28	0.548	10.8	13.9	0.470	1.39	0.32	0.933
**Phosphotransferase**	A0A6J3JUQ3 (+4)	31.9	30.9	-0.266	0.83	0.293	0.557	45.1	42.8	-0.012	0.99	0.944	0.972
**Aminopeptidase**	A0A6J3KHZ5	23.0	32.6	0.283	1.22	0.338	0.607	25.6	24.9	0.030	1.02	0.941	0.972
**inducible metalloproteinase inhibitor protein-like**	A0A6J3LE39	157.3	29.5	-1.084	0.47	0.342	0.608	908.7	893.4	0.020	1.01	0.878	0.972
**apolipophorins**	A0A6J3L183	546.8	748.8	0.219	1.16	0.358	0.625	433.4	467.2	0.160	1.12	0.231	0.933
**putative serine protease K12H4.7**	A0A6J3KU14	35.4	37.2	-0.183	0.88	0.394	0.651	36.1	34.0	-0.301	0.81	0.601	0.933
**plancitoxin-1 isoform X1**	A0A6J3K9T1 (+1)	28.5	28.7	-0.226	0.86	0.404	0.662	—	—	—	—	—	—
**thioredoxin**	A0A6J3KE25	48.2	51.2	-0.112	0.93	0.612	0.784	21.5	14.0	-0.483	0.72	0.121	0.933
**Dipeptidase**	A0A6J3L5M3	16.4	21.8	0.110	1.08	0.615	0.784	29.8	26.8	-0.103	0.93	0.506	0.933
**venom serine protease 34-like**	A0A6J3L320	64.4	80.1	0.095	1.07	0.758	0.882	42.4	51.3	0.361	1.28	0.219	0.933
**Catalase**	A0A6J3K8Z3 (+1)	31.7	38.5	0.053	1.04	0.822	0.923	—	—	—	—	—	—
**Carboxypeptidase**	A0A6J3JZZ8	10.2	11.2	-0.053	0.96	0.86	0.931	23.9	20.3	-0.312	0.81	0.394	0.933
**Peptidyl-prolyl cis-trans isomerase**	A0A6J3KL98	25.5	30.3	0.022	1.02	0.924	0.967	14.8	11.7	-0.323	0.80	0.464	0.933
**serine protease inhibitor 88Ea-like isoform X2**	A0A6J3KBG4 (+1)	4.3	2.7	—	—	—	—	52.0	69.2	0.442	1.36	0.159	0.933
**CLIP domain-containing serine protease-2**	A0A6J3L236	—	—	—	—	—	—	884.4	935.9	0.139	1.10	0.29	0.933
**Carboxypeptidase Q**	A0A6J3JU92 (+1)	8.6	6.1	—	—	—	—	16.8	15.1	0.165	1.12	0.713	0.933
**venom protease-like isoform X1**	A0A6J3KU65 (+1)	7.4	5.9	—	—	—	—	40.2	25.4	0.402	1.32	0.862	0.972
**digestive cysteine proteinase 1**	A0A6J3L714	5.0	6.7	—	—	—	—	8.2	5.6	—	—	—	—
**esterase E4-like**	A0A6J3KLB0	5.7	5.0	—	—	—	—	3.1	8.0	—	—	—	—
**matrix metalloproteinase-14 isoform X1**	A0A6J3KAJ7 (+3)	2.3	1.3	—	—	—	—	3.6	5.9	—	—	—	—

**omega-conotoxin-like protein 1**	A0A6J3K3Y1	0.8	0.0	—	—	—	—	6.3	5.3	—	—	—	—
**peptidylprolyl isomerase**	A0A6J3LHL2	7.3	7.0	—	—	—	—	7.1	4.1	—	—	—	—
**putative aminopeptidase-2 isoform X1**	A0A6J3KAY4 (+1)	3.0	6.7	—	—	—	—	5.0	3.8	—	—	—	—
**sodium/calcium exchanger regulatory protein 1 isoform X1**	A0A6J3KUY4	5.5	10.0	—	—	—	—	9.2	7.9	—	—	—	—
**thioredoxin-like protein 1**	A0A6J3LMV1	2.8	5.6	—	—	—	—	5.6	2.8	—	—	—	—

Significant differences in toxin abundance between populations are indicated in bold (t-test, $p < 0.05$, FDR < 0.05). Mean raw spectral counts at low and high elevation (n = 4/group). Differential-abundance statistics were obtained using limma-voom. Log2 fold-change (log2FC) represents high- relative to low-elevation. Low and high columns are arithmetic means of raw spectral counts across the 4 replicates. False Discovery Rate (FDR) values are Benjamini-Hochberg adjusted.
